# Supplementary material for: Changes in soft drinks purchased by British households associated with the UK soft drinks industry levy: a controlled interrupted time series analysis
Source: BMJ Open. 2023 Dec 4;13(12):e077059. doi: 10.1136/bmjopen-2023-077059 (PMC10711915; doi:10.1136/bmjopen-2023-077059)
Supplement: Supplementary data [file bmjopen-2023-077059supp001.pdf]

Supplementary material A

STROBE Statement—checklist of items that should be included in reports of observational studies

	Item No	Recommendation	Section and Paragraph No
**Title and abstract**	1	(*a*) Indicate the study's design with a commonly used term in the title or the abstract	Title.
		(*b*) Provide in the abstract an informative and balanced summary of what was done and what was found	Abstract: main outcome measures; results; conclusions.
**Introduction**			
Background/rationale	2	Explain the scientific background and rationale for the investigation being reported	Introduction: paragraphs 1-3.
Objectives	3	State specific objectives, including any prespecified hypotheses	Introduction: paragraph 3-4.
**Methods**			
Study design	4	Present key elements of study design early in the paper	Methods: paragraph 1.
Setting	5	Describe the setting, locations, and relevant dates, including periods of recruitment, exposure, follow-up, and data collection	Methods: data source; overall analysis strategy; Figure 1.
Participants	6	(*a*) *Cohort study*—Give the eligibility criteria, and the sources and methods of selection of participants. Describe methods of follow-up<br>*Case-control study*—Give the eligibility criteria, and the sources and methods of case ascertainment and control selection. Give the rationale for the choice of cases and controls<br>*Cross-sectional study*—Give the eligibility criteria, and the sources and methods of selection of participants	Methods: data source, product categories – drinks, confectionery and toiletries; control group; outcome measures.
		(*b*) *Cohort study*—For matched studies, give matching criteria and number of exposed and unexposed<br>*Case-control study*—For matched studies, give matching criteria and the number of controls per case	NA.
Variables	7	Clearly define all outcomes, exposures, predictors, potential confounders, and effect	Outcome measures; overall analysis strategy; primary analysis: category specific analyses; secondary analysis: all soft drinks categories combined, irrespective of levy

		modifiers. Give diagnostic criteria, if applicable	eligibility; sensitivity analysis: excluding small manufacturers; sensitivity analysis: ITS without a control category; supplementary material B.
Data sources/ measurement	8*	For each variable of interest, give sources of data and details of methods of assessment (measurement). Describe comparability of assessment methods if there is more than one group	Data source; overall analysis strategy; primary analysis: category specific analyses; secondary analysis: all soft drinks categories combined, irrespective of levy eligibility; sensitivity analysis: excluding small manufacturers; sensitivity analysis: ITS without a control category; supplementary material B.
Bias	9	Describe any efforts to address potential sources of bias	Product categories: drinks confectionery and toiletries; control group; sensitivity analysis: excluding small manufacturers; sensitivity analysis: ITS without a control category.
Study size	10	Explain how the study size was arrived at	Data source.
Quantitative variables	11	Explain how quantitative variables were handled in the analyses. If applicable, describe which groupings were chosen and why	Overall analysis strategy; primary analysis: category specific analyses; secondary analysis: all soft drinks categories combined, irrespective of levy eligibility; Box 1.
Statistical methods	12	(*a*) Describe all statistical methods, including those used to control for confounding	Control group; sensitivity analysis: ITS without a control category; Supplementary material A.
		(*b*) Describe any methods used to examine subgroups and interactions	Overall analysis strategy; primary analysis: category specific analyses; secondary analysis: all soft drinks categories combined, irrespective of levy eligibility.
		(*c*) Explain how missing data were addressed	Methods: data source
		(*d*) *Cohort study*—If applicable, explain how loss to follow-up was addressed<br>*Case-control study*—If applicable, explain how matching of cases and controls was addressed<br>*Cross-sectional study*—If applicable, describe analytical methods taking account of sampling strategy	Methods: data source.

Results

Participants	13*	(a) Report numbers of individuals at each stage of study—eg numbers potentially eligible, examined for eligibility, confirmed eligible, included in the study, completing follow-up, and analysed	Results: paragraph 1.
		(b) Give reasons for non-participation at each stage	Data source.
		(c) Consider use of a flow diagram	-
Descriptive data	14*	(a) Give characteristics of study participants (eg demographic, clinical,	Results: paragraph 1; Supplementary material C.

		social) and information on exposures and potential confounders	
		(b) Indicate number of participants with missing data for each variable of interest	Results: paragraph 1.
		(c) *Cohort study*—Summarise follow-up time (eg, average and total amount)	NA.
Outcome data	15*	*Cohort study*—Report numbers of outcome events or summary measures over time	NA.
		Case-control study—Report numbers in each exposure category, or summary measures of exposure	NA.
		Cross-sectional study—Report numbers of outcome events or summary measures	Results: paragraph 2; Table 1.
Main results	16	(*a*) Give unadjusted estimates and, if applicable, confounder-adjusted estimates and their precision (eg, 95% confidence interval). Make clear which confounders were adjusted for and why they were included	Primary analysis: category specific results; high tier drinks; low tier drinks; no levy drinks; levy exempt drinks and confectionery; Figures 2-3; Tables 2-3; supplementary material D.
		(*b*) Report category boundaries when continuous variables were categorized	Introduction - paragraph 3; product categories: drinks, confectionery and toiletries; Box 1.
		(*c*) If relevant, consider translating estimates of relative risk into absolute risk for a meaningful time period	Tables 2-3.
Other analyses	17	Report other analyses done—eg analyses of subgroups and interactions, and sensitivity analyses	Sensitivity analysis: excluding small manufacturers; sensitivity analysis: no control category; supplementary tables 4a-b; supplementary material G.
**Discussion**			
Key results	18	Summarise key results with reference to study objectives	Summary of main findings.
Limitations	19	Discuss limitations of the study, taking into account sources of potential bias or imprecision. Discuss both direction and magnitude of any potential bias	Key strengths and limitations.
Interpretation	20	Give a cautious overall interpretation of results considering objectives, limitations, multiplicity of analyses, results from similar studies, and other relevant evidence	Interpretation of findings - paragraphs 1-3.
Generalisability	21	Discuss the generalisability (external validity) of the study results	Interpretation of findings - paragraphs 4.
**Other information**			
Funding	22	Give the source of funding and the role of the funders for the present study and, if applicable, for the original study on which the present article is based	Funding.

*Give information separately for cases and controls in case-control studies and, if applicable, for exposed and unexposed groups in cohort and cross-sectional studies.

Note: An Explanation and Elaboration article discusses each checklist item and gives methodological background and published examples of transparent reporting. The STROBE checklist is best used in conjunction with this article (freely available on the Web sites of PLoS Medicine at http://www.plosmedicine.org/, Annals of Internal Medicine at http://www.annals.org/, and Epidemiology at http://www.epidem.com/). Information on the STROBE Initiative is available at www.strobe-statement.org.

Supplementary material B

The model specification is given by:

$Y_t = \beta_0 + \beta_1 T + \beta_2 A_t + \beta_3 A_t\, T + \beta_4 A_t\, Z + \beta_5 A_t\, TZ + \beta_6 I_t + \beta_7 I_t\, T + \beta_8 I_t\, Z + \beta_9 I_t\, TZ + e_t$

Y	*Average volume of (or purchased sugar in) drink or confectionery per household per week at week t (t=1,….,264)*
T	*Weeks since the start of the study; 1,…,264*
A_t	*0 if t prior to announcement, 1 if t on or after announcement*
I_t	*0 if t prior to implementation, 1 if t on or after implementation*
Z	*Control category (toiletries) = 0, drink or confectionery category = 1*
e_t	*$N(0,\sigma^2)$ representing the residual variance of the model*

Dummy indicator variables determined to be statistically significant ($p<0.05$) were included for the intervention group as appropriate representing: interaction terms restart at 0 at the point of the interventions; the increase in purchases seen throughout December in the weeks before Christmas; the fall in purchases in the weeks immediately after Christmas; and the increase in confectionery purchases seen at Easter, for toiletries these were set to 0. To adjust for temperature-related trends in drink consumption the average UK monthly temperature was included in the intervention group with the average study period temperature used for toiletries.[5] Quadratic functions of announcement trends were included where they improved model fit - assessed using likelihood ratio tests. Stationary was examined using augmented Dickey-Fuller tests.[41] Autocorrelation between preceding time points was examined using autocorrelation and partial-autocorrelation plots. An appropriate autocorrelation structure was determined and then compared to alternative models using likelihood ratio tests. Visual inspection of the data suggested no additional benefit would be gained from including polynomial terms.

Supplementary material C

Supplementary Table 1: Demographic characteristics of Kantar Worldpanel take-home panel households from March 2014 – March 2018 (weighted)

	Level	Kantar Worldpanel (%)	UK population (%)
Children in household[1]	No	65.3	71.5
	Yes	34.6	28.5
Social grade of chief income earner[2]	AB: Higher and intermediate managerial	19.3	27
	C1: Junior managerial	34.1	28
	C2: Skilled manual workers	16.4	20
	D: Semi and unskilled-manual workers	12.4	15
	E: lowest grade workers	7.9	10
Total household income (£ per annum)[3]	0-9,999	6.4	..
	10,000-19,999	18.9	..
	20,000-29,999	17.2	..
	30,000-39,999	13.1	....
	40,000-49,999	9.0	..
	50,000-59,999	5.5	..
	60,000-69,999	2.9	..
	70,000+	4.1	..
	Refused to answer	14.4	..
	Mean (£)	..	35,697
	Median (£)	..	28,947
Highest qualification of chief income earner[4]	Higher than School leaving qualifications taken at ~18 years (e.g. A-Levels)	38.3	43.8
	School leaving qualifications taken at ~18 years (e.g. A-Levels)	12.3	22.4
	School leaving qualifications taken at ~16 years (e.g. GCSE)	20.6	18.7
	Other (including no qualifications and unknown)	16.2	15.1

[1]Average of households with dependent children from 2014-2018; [2]UK population figures from 2016; [3]No directly comparable figures available from ONS, mean and medians are averaged over 2014-2019; [4]UK population figures from 2014

Supplementary material D

Supplementary Figure 1a. Observed and modelled volume (ml) of drinks exempt from the Soft Drinks Industry Levy, subcategories of low levy drinks and bottled water purchased per household per week, March 2014- March 2019 (weighted)

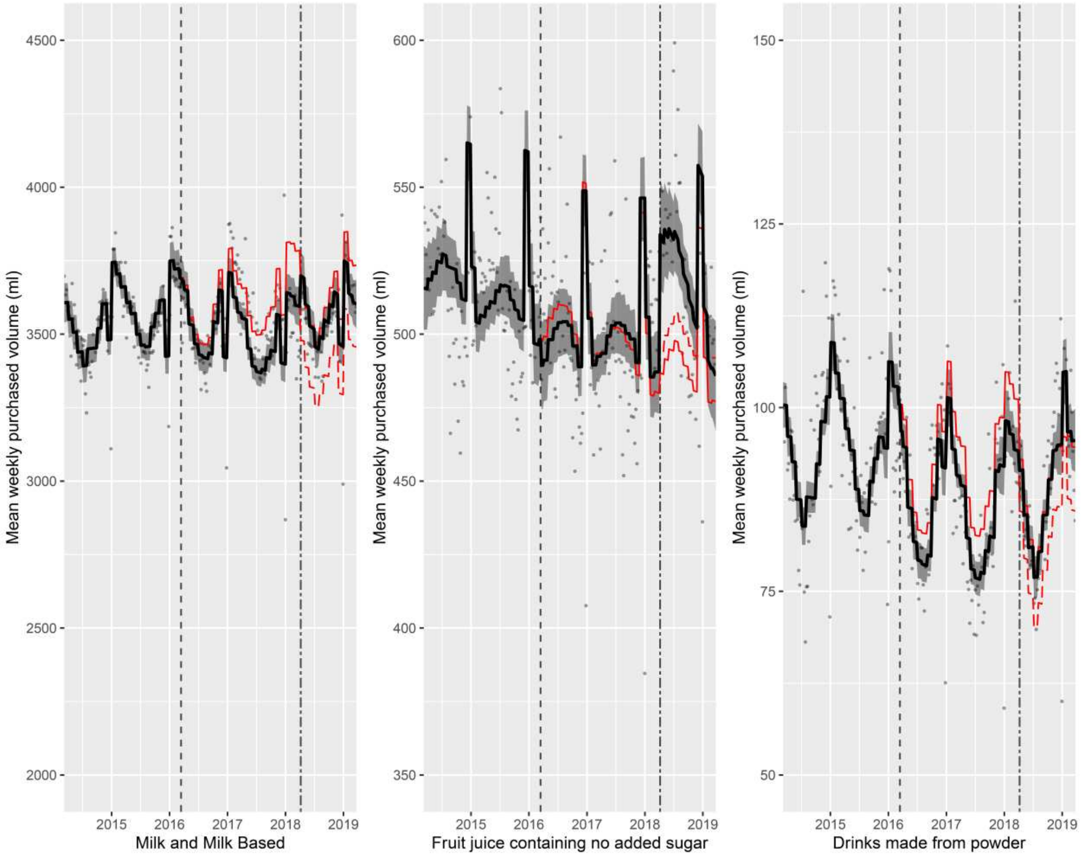

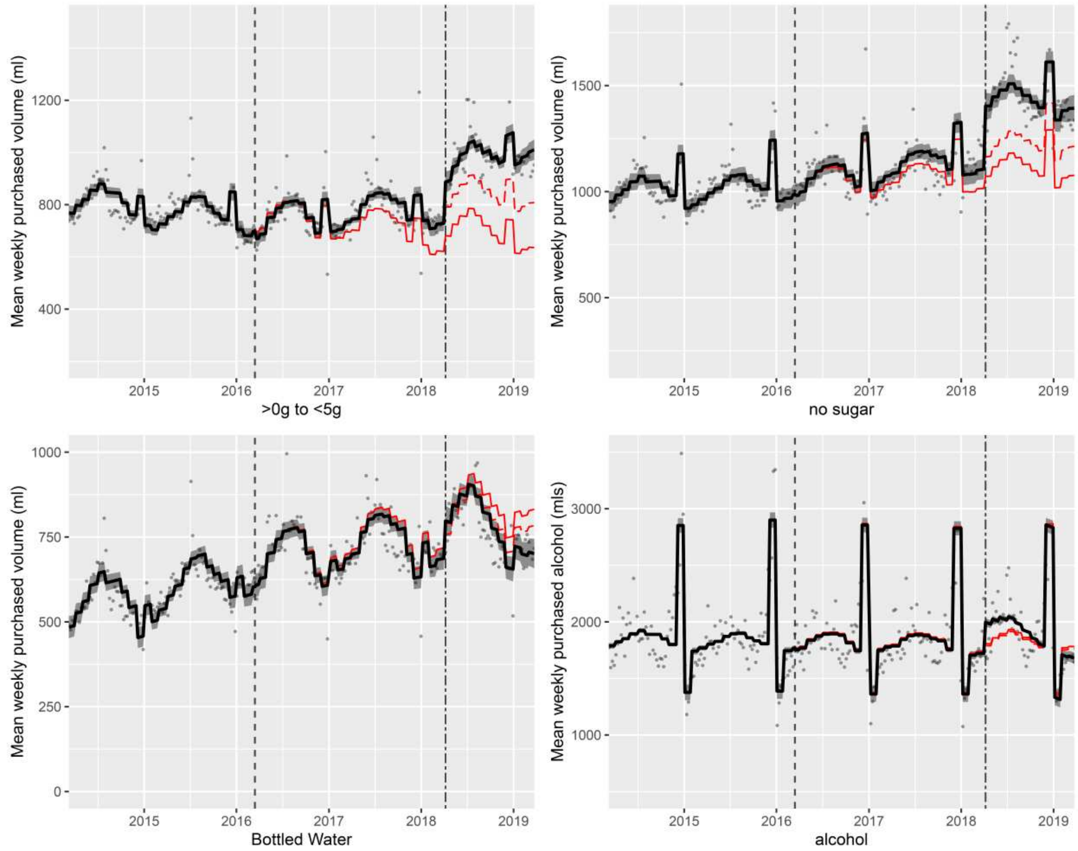

Notes. Points are observed data, black lines (with shadows) are modelled data (and 95% confidence intervals); red lines indicate counterfactuals had the announcement (red solid line) and implementation (red dashed line) not happened; the first dashed vertical line indicates the point of announcement; the second dashed vertical line indicates the point of implementation; The Y-axis varies in scale between panels to maximise the resolution of figures; modelled purchases include averaged effects for seasonality and the impact of December and January (Christmas period). The control category of toiletries is shown in Figure 3.

Supplementary Figure 1b. Observed and modelled amount of sugar in no levy drinks containing sugar (low sugar) and drinks exempt from the Soft Drinks Industry Levy purchased per household per week, March 2014- March 2019 (weighted)

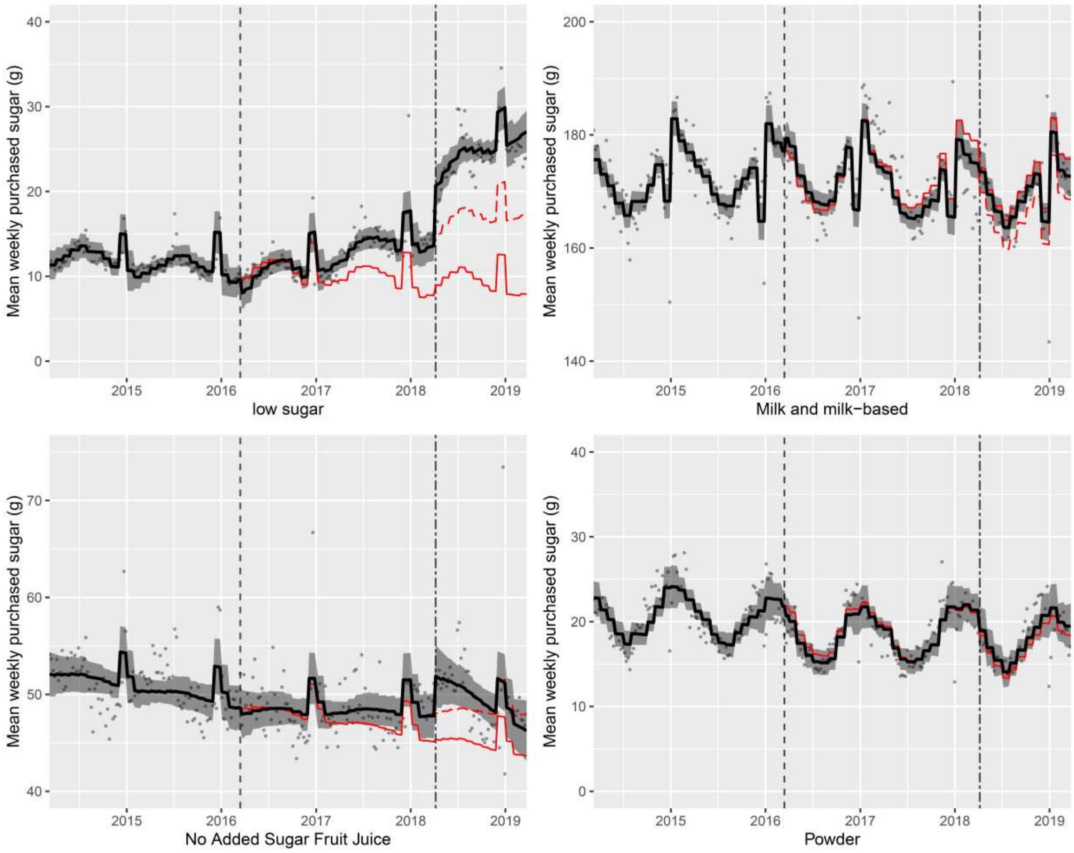

Notes. Points are observed data, black lines (with shadows) are modelled data (and 95% confidence intervals); red lines indicate counterfactuals had the announcement (red solid line) and implementation (red dashed line) not happened; the first dashed vertical line indicates the point of announcement; the second dashed vertical line indicates the point of implementation; The Y-axis varies in scale between panels to maximise the resolution of figures; modelled purchases include averaged effects for seasonality and the impact of December and January (Christmas period). The control category of toiletries is shown in Figure 3.

Supplementary Table 2a: Modelled level and trend changes in volume of drinks (ml) and confectionery (g) purchased per household per week (95% CI) in relation to the UK SDIL, March 2014 - March 2019 (weighted)

	Analysis 1: pre-post announcement (March 2014 – March 2018)		Analysis 2: pre-post implementation (March 2016 – March 2019)		Analysis 3: pre-post announcement & implementation (March 2014 – March 2019)	
	Level Change	Trend Change	Level Change	Trend Change	Level Change	Trend Change
Levy liable drinks						
High tier (≥8g sugar per 100ml)	44.0 (-7.7, 95.7)	-0.1 (-0.9, 0.7)	**-117.2 (-183.3, -51.1)**	1.0 (-0.9, 2.9)	-73.2 (-157.1, 10.7)	0.9 (-1.2, 3.0)
Low tier (≥5g - <8g sugar per 100ml)	-0.11 (-22.4, 22.1)	**-0.6 (-1.0, -0.3)**	-26.3 (-53.6, 1.04)	-0.81 (-1.6, 0.01)	-26.4 (-61.7, 8.8)	**-1.5 (-2.4, -0.6)**
No levy (<5g sugar per 100ml)	-28.2 (-149.4, 92.9)	**2.0 (0.04, 3.9)**	**372.0 (217.6, 526.4)**	**0.52 (-3.9, 4.9)**	**343.8 (147.5, 540.0)**	2.5 (-2.3, 7.3)
>0g to <5g sugar per 100ml	-27.1 (-82.6, 28.5)	**1.3 (0.4, 2.2)**	**88.1 (17.0, 159.3)**	**2.31 (0.3, 4.3)**	61.0 (-29.2, 151.4)	**3.6 (1.4, 5.8)**
0g sugar per 100ml	-7.37 (-93.9, 79.2)	0.9 (-0.6, 2.4)	**231.0 (125.2, 336.8)**	-0.99 (-4.3, 2.3)	**223.6 (87.0, 360.3)**	-0.08 (-3.7, 3.5)
Bottled water	6.86 (-53.4, 67.2)	-0.4 (-1.4, 0.6)	36.6 (-38.8, 112.0)	-2.24 (-4.5, 0.005)	43.5 (-53.1, 140.0)	**-2.6 (-5.1, -0.2)**
Levy exempt drinks						
Alcoholic drinks	-10.8 (-48.6, 27.0)	-0.07 (-0.5, 0.3)	**208.0 (137.9, 278.1)**	**-5.7 (-7.8, -3.6)**	**197.2 (117.6, 276.8)**	**-5.8 (-7.9, -3.7)**
Milk and milk based drinks*	-6.61 (-105.8, 92.6)	-1.7 (-4.0, 0.5)	**210.7 (98.7, 322.7)**	-1.2 (-5.3, 2.9)	**204.1 (54.4, 353.7)**	-2.9 (-7.6, 1.8)
No added sugar fruit juices	-8.72 (-34.3, 16.9)	0.1 (-0.3, 0.6)	30.5 (-0.9, 61.9)	-0.7 (-1.6, 0.3)	21.78 (-18.8, 62.3)	-0.5 (-1.6, 0.5)
Drinks sold as powders (g)	-1.79 (-7.8, 4.18)	-0.06 (-0.2, 0.03)	0.10 (-7.7, 7.9)	**0.3 (0.04, 0.5)**	-1.69 (-11.6, 8.17)	0.2 (-0.04, 0.4)
Other categories						
Confectionery (g)	-17.0 (-88.0, 54.0)	0.07 (-1.3, 1.5)	-77.9 (-163.9, 8.19)	2.4 (-0.6, 5.4)	**-98.9 (-206.4, -16.7)**	2.5 (-0.8, 5.8)

Bold indicates a significant difference at the 95% confidence interval level.
*Milk comprises drinks in the following categories: semi-skimmed; specific low fat % milk (e.g. 1% fat milk); whole milk; buttermilk; modified milk; other milk; other non-cows milk; rice drink; soya milk. Skimmed milk is excluded from all analysis in this paper due to missing data.

Supplementary Table 2b: Modelled level and trend changes in sugar in drinks and confectionery (g) purchased per household per week (95% CI) in relation to the UK SDIL, March 2014 - March 2019 (weighted)

	Analysis 1: pre-post announcement (March 2014 – March 2018)		Analysis 2: pre-post implementation (March 2016 – March 2019)		Analysis 3: pre-post announcement & implementation (March 2014 – March 2019)	
	Level Change	Trend Change	Level Change	Trend Change	Level Change	Trend Change
Levy liable drinks						
High tier (≥8g sugar per 100ml)	**8.9 (6.8, 10.9)**	**-0.05 (-0.08, -0.03)**	**-11.5 (-15.3, -7.7)**	**-0.1 (-0.2, -0.02)**	-2.6 (-6.9, 1.6)	**-0.2 (-0.3, -0.07)**
Low tier (≥5g - <8g sugar per 100ml)	1.7 (-1.6, 5.1)	**-0.07 (-0.1, -0.02)**	**-6.2 (-10.6, -1.8)**	0.09 (-0.03, 0.2)	-4.5 (-10.0, 1.1)	0.02 (-0.1, 0.2)
No levy (<5g sugar per 100ml)	0.2 (-3.1, 3.5)	0.04 (-0.01, 0.09)	0.8 (-3.6, 5.3)	**0.2 (0.1, 0.4)**	1.0 (-4.5, 6.6)	**0.3 (0.2, 0.4)**
>0g to <5g sugar per 100ml	0.2 (-3.1, 3.5)	0.04 (-0.01, 0.09)	0.8 (-3.6, 5.3)	**0.2 (0.1, 0.4)**	1.0 (-4.5, 6.6)	**0.3 (0.2, 0.4)**
Levy exempt drinks						
Milk and milk based drinks*	4.1 (-0.1, 9.1)	**-0.09 (-0.2, -0.01)**	-2.0 (-8.6, 4.6)	**0.2 (0.01, 0.4)**	2.1 (-6.2, 10.4)	0.1 (-0.09, 0.3)
No added sugar fruit juices	1.0 (-3.4, 5.4)	0.001 (-0.06, 0.06)	-1.6 (-7.3, 4.1)	0.07 (-0.09, 0.2)	-0.6 (-7.8, 6.6)	0.07 (-0.1, 0.2)
Drinks sold as powders (g	0.6 (-3.3, 4.5)	-0.02 (-0.08, 0.04)	-5.1 (-0.004, 10.2)	**0.2 (0.03, 0.3)**	-4.5 (-10.9, 1.9)	**0.2 (0.001, 0.3)**
Other categories						
Confectionery (g)	-8.6 (-49.7, 32.6)	0.03 (-0.8, 0.9)	-49.4 (-99.2, 0.4)	1.5 (-0.2, 3.2)	**-58.0 (-122.6, -6.7)**	1.6 (-0.4, 3.5)

Bold indicates a significant difference at the 95% confidence interval level.

*Milk comprises drinks in the following categories: semi-skimmed; specific low fat % milk (e.g. 1% fat milk); whole milk; buttermilk; modified milk; other milk; other non-cows milk; rice drink; soya milk. Skimmed milk is excluded from all analysis in this paper due to missing data.

Supplementary material E

Secondary analysis: all drinks categories combined irrespective of levy eligibility

Supplementary Table 3a: Level and trend changes in volume of, and sugar in, all soft drinks (ml) and confectionery (g) purchased per household per week (95% CI) in relation to the UK SDIL, March 2014 - March 2019 (weighted)

	Analysis 1: pre-post announcement (March 2014 – March 2018)		Analysis 2: pre-post implementation (March 2016 – March 2019)		Analysis 3: pre-post announcement & implementation (March 2014 – March 2019)	
	Level Change	Trend Change	Level Change	Trend Change	Level Change	Trend Change
All soft drinks - volume						
Higher tier (≥8g sugar per 100ml)	22.8 (-37.5, 83.1)	0.5 (-0.5, 1.5)	-76.8 (-154.0 ,0.4)	-2.2 (-4.4, 0.01)	-54.0 (-152.0, 44.0)	-1.7 (-4.1, 0.7)
Lower tier (≥5g - <8g sugar per 100ml)	19.0 (-52.2, 90.2)	**-3.2 (-5.9, -0.5)**	27.9 (-47.8, 103.6)	-4.4 (-8.9, 0.02)	46.9 (-57.0, 150.8)	**-7.6 (-12.9, -2.4)**
Drinks containing <5g sugar per 100ml)	-152.1 (-348.3, 44.1)	2.6 (-0.7, 5.8)	**435.7 (187.7, 683.7)**	1.1 (-6.0, 8.3)	283.6 (-32.6, 599.8)	3.7 (-4.2, 11.5)
Levy liable drinks combined	15.2 (-72.9, 103.3)	0.6 (-0.8, 2.0)	-39.7 (-152.7, 73.4)	0.01 (-3.2, 3.2)	-24.5 (-167.8, 118.9)	0.6 (-2.9, 4.1)
All soft drinks excluding milk*	12.9 (-205.3, 231.0)	1.0 (-2.6, 4.5)	**347.6 (70.1, 625.0)**	-5.2 (-13.1, 2.7)	**360.5 (7.47, 713.4)**	-4.2(-12.9, 4.5)
All soft drinks combined	33.9 (-189.0, 256.7)	-0.2 (-3.8, 3.4)	**453.0 (170.1, 735.9)**	-5.1 (-13.2, 3.0)	**486.9 (126.7, 847.0)**	-5.4 (-14.2, 3.5)
All soft drinks – sugar						
Higher tier (≥8g sugar per 100ml)	4.1 (-2.6, 10.8)	0.04 (-0.07, 1.1)	**-13.4 (-22.2, -4.7)**	-0.1 (-0.4, 0.1)	-9.3 (-20.3, 1.7)	-0.08 (-0.3, 0.2)
Lower tier (≥5g - <8g sugar per 100ml)	**11.0 (5.6, 16.3)**	**-0.2 (-0.3, -0.1)**	-2.7 (-9.67, 4.3)	-0.01 (-0.2,0.2)	8.3 (-0.5, 17.1)	**-0.2 (-0.4, -0.01)**
Drinks containing <5g sugar per 100ml)	-6.3 (-12.2, -0.4)	**1.0 (0.9, 1.0)**	-0.7 (-8.30, 7.0)	**0.4 (0.2, 0.6)**	-7.0 (-16.6, 2.7)	**1.4 (1.1, 1.6)**
Levy liable drinks combined	5.0 (-1.8, 11.8)	-0.003 (-0.1, 0.1)	**-13.1 (-22.0, -4.2)**	0.02 (-0.2, 0.3)	-8.1(-19.3, 3.1)	0.02 (-0.3, 0.3)
All soft drinks excluding milk*	3.1 (-4.6, 10.8)	0.04 (-0.08, 0.2)	-9.3 (-19.2, 0.7)	-0.09 (-0.4, 0.2)	-6.2 (-18.8, 6.4)	-0.05 (-0.4, 0.3)
All soft drinks combined	5.3 (-2.6, 13.2)	-0.02 (-0.1, 0.1)	-5.7 (-15.9, 4.5)	-0.08 (-0.4, 0.2)	-0.4 (-13.2, 12.5)	-0.1 (-0.4, 0.2)

Bold indicates a significant difference at the 95% confidence interval level. The levy liable drinks category is a combination of high tier, low tier and no levy drinks.

*Milk comprises drinks in the following categories: semi-skimmed; specific low fat % milk (e.g. 1% fat milk); whole milk; buttermilk; modified milk; other milk; other non-cows milk; rice drink; soya milk. Skimmed milk is excluded from all analysis in this paper due to missing data.

Supplementary Table 3b: Absolute and relative change in volume of, and sugar in, all soft drinks and confectionery (g) purchased per household (95% CI) per week in relation to the UK SDIL, March 2014- March 2019 (weighted)

	Analysis 1: pre-post announcement (March 2014 – March 2018)		Analysis 2: pre-post implementation (March 2016 – March 2019)		Analysis 3: pre-post announcement & implementation (March 2014 – March 2019)	
	Absolute change (ml or g)	Relative change (%)	Absolute change (ml or g)	Relative change (%)	Absolute change (ml or g)	Relative change (%)
All soft drinks - volume						
High tier (≥8g sugar per 100ml)	**76.6 (45.6, 107.7)**	**7.5 (4.5, 10.6)**	**-190.9 (-233.5, -148.3)**	**-19.4 (-23.7, -15.1)**	**-85.6 (-128.2, -43.0)**	**-9.8 (-14.6, -4.9)**
Low tier (≥5g - <8g sugar per 100ml)	316.7 (-241,7, -391.7)	**-28.0 (-21.3, -34.6)**	**-205.2 (-295.9, -114.5)**	**-26.2 (-37.8, -14.6)**	**-693.6 (-784.3, -602.9)**	**-54.5 (-61.7, -47.4)**
Drinks containing <5g sugar per 100ml)	**114.3 (12.4, 216.2)**	**2.2 (0.2, 4.1)**	**506.7 (367.3, 646.1)**	**9.0 (6.5, 11.4)**	**760.2 (620.8, 899.6)**	**14.1 (11.5, 16.6)**
Levy liable drinks combined	**76.5 (31.1, 121.8)**	**6.0 (2.5, 9.6)**	-42.7 (-104.9, 19.6)	-3.3(-8.1, 1.5)	**66.3 (4.1, 128.6)**	**5.6 (0.3, 10.8)**
All soft drinks excluding milk*	**116.1 (3.3, 229.0)**	**3.3 (0.09, 6.4)**	79.6 (-74.8, 234.1)	2.1 (-2.0, 6.1)	**250.1 (95.7, 404.5)**	**6.8 (2.6, 11.0)**
All soft drinks combined	11.8 (-103.7, 127.3)	0.2 (-1.4, 1.7)	**187.8 (29.7, 345.9)**	**2.6 (0.4, 4.7)**	**188.8 (30.7, 346.9)**	**2.6 (0.4, 4.7)**
All soft drinks – sugar						
High tier (≥8g sugar per 100ml)	**9.3 (5.9, 12.8)**	**7.6 (4.8, 10.4)**	**-22.9 (-27.8, -18.1)**	**-19.4 (-23.4, -15.3)**	**-9.99 (-14.8, -5.18)**	**-9.5 (-14.0, -4.9)**
Low tier (≥5g - <8g sugar per 100ml)	**-9.7 (-12.5, -6.9)**	**-18.6 (-23.9, -13.3)**	**-7.2 (-11.0, -3.4)**	**-19.4 (-29.7, -9.1)**	**-26.7 (-30.5, -22.9)**	**-47.1 (-53.9, -40.4)**
Drinks containing <5g sugar per 100ml)	**5.1 (2.1, 8.1)**	**3.6 (1.5, 5.8)**	**16.7 (12.5, 20.9)**	**11.2 (8.4, 14.0)**	**28.6 (24.4, 32.8)**	**20.9 (17.9, 23.9)**
Levy liable drinks combined	**6.1 (2.6, 9.6)**	**8.6 (3.6, 13.5)**	**-15.5 (-20.4, -10.7)**	**-22.7 (-29.8, -15.6)**	**-8.0 (-12.9, -3.2)**	**-13.2 (-21.1, -5.2)**
All soft drinks excluding milk*	**8.9 (5.0, 12.8)**	**6.5 (3.6, 9.4)**	**-17.2 (-22.7, -11.8)**	**-12.7 (-16.7, -8.67)**	**-4.5 (-10.0, 1.0)**	**-3.7 (-8.1, 0.8)**
All soft drinks combined	**4.6 (0.5, 8.6)**	**1.4 (0.2, 2.7)**	**-12.9 (-18.5, -7.4)**	**-4.3 (-6.1, -2.4)**	**-8.0 (-13.6, -2.4)**	**-2.7 (-4.5, -0.8)**

Bold indicates a significant difference at the 95% confidence interval level. The levy liable drinks category is a combination of high tier, low tier and no levy drinks.

*Milk comprises drinks in the following categories: semi-skimmed; specific low fat % milk (e.g. 1% fat milk); whole milk; buttermilk; modified milk; other milk; other non-cows milk; rice drink; soya milk. Skimmed milk is excluded from all analysis in this paper due to missing data.

Supplementary material F

Sensitivity analysis: excluding small manufacturers

Supplementary table 4a: Modelled level and trend changes in volume of, and sugar in, drinks (ml) purchased per household per week (95% CI) in relation to the UK SDIL, March 2014 - March 2019; excluding small manufacturers (weighted)

	Analysis 1: pre-post announcement (March 2014 – March 2018)		Analysis 2: pre-post implementation (March 2016 – March 2019)		Analysis 3: pre-post announcement & implementation (March 2014 – March 2019)	
	Level Change	Trend Change	Level Change	Trend Change	Level Change	Trend Change
Volume						
High levy tier (≥8g sugar per 100ml)						
All Manufacturers	44.0 (-7.7, 95.7)	-0.1 (-0.9, 0.7)	**-117.2 (-183.3, -51.1)**	1.0 (-0.9, 2.9)	-73.2 (-157.1, 10.7)	0.9 (-1.2, 3.0)
Excluding Manufacturers with <1M Litres	41.5 (-9.7, 92.7)	-0.09 (-0.9, 0.7)	**-111.9 (-177.3, -46.6)**	-1.1 (-2.9, 0.8)	-70.8 (-153.4, 12.6)	-1.2 (-3.2, 0.9)
Excluding Manufacturers with <0.5M Litres	41.5 (-9.7, 92.7)	-0.09 (-0.9, 0.7)	**-111.9 (-177.3, -46.6)**	-1.1 (-2.9, 0.8)	-70.8 (-153.4, 12.6)	-1.2 (-3.2, 0.9)
Low levy tier (≥5g - <8g sugar per 100ml)						
All Manufacturers	-0.1 (-22.4, 22.1)	**-0.6 (-1.0, -0.3)**	-26.3 (-53.6, 1.0)	-0.8 (-1.6, 0.01)	-26.4 (-61.7, 8.8)	**-1.5 (-2.4, -0.6)**
Excluding Manufacturers with <1M Litres	-2.1 (-24.0, 19.9)_	**-0.7 (-1.0, -0.3)**	-20.9 (-47.5, 5.7)	**-0.9 (-1.8, -0.1)**	-23.0 (-57.5, 11.6)	**-1.6 (-2.5, -0.7)**
Excluding Manufacturers with <0.5M Litres	-2.1 (-24.0, 19.6)_	**-0.7 (-1.0, -0.3)**	-20.7 (-47.3, 5.9)	**-0.9 (-1.8, -0.1)**	-22.9 (-57.2, 11.5)	**-1.6 (-2.5, -0.7)**
Sugar						
High tier (≥8g sugar per 100ml)						
All Manufacturers	**8.9 (6.8, 10.9)**	**-0.05 (-0.08, -0.03)**	**-11.5 (-15.3, -7.7)**	**-0.1 (-0.2, -0.02)**	-2.6 (-6.9, 1.6)	**-0.2 (-0.3, -0.07)**
Excluding Manufacturers with <1M Litres	4.5 (-1.3, 10.2)	-0.03 (-0.1, 0.06)	**-13.5 (-20.8, -6.1)**	-0.07 (-0.3, 0.1)	-9.0 (-18.3, 0.4)	-0.1 (-0.3, 0.1)
Excluding Manufacturers with <0.5M Litres	4.5 (-1.3, 10.2)	-0.03 (-0.1, 0.06)	**-13.5 (-20.8, -6.1)**	-0.07 (-0.3, 0.1)	-9.0 (-18.3, 0.4)	-0.1 (-0.3, 0.1)
Low tier (≥5g - <8g sugar per 100ml)						
All Manufacturers	1.7 (-1.6, 5.1)	**-0.07 (-0.1, -0.02)**	**-6.2 (-10.6, -1.8)**	0.09 (-0.03, 0.2)	-4.5 (-10.0, 1.1)	0.02 (-0.1, 0.2)
Excluding Manufacturers with <1M Litres	-0.4 (-2.1, 1.3)	**-0.07 (-0.1, -0.04)**	**-2.5 (-4.6, -0.4)**	0.002 (-0.06, 0.06)	**-3.0 (-5.7, -0.2)**	-0.007 (-0.1, 0.0001)
Excluding Manufacturers with <0.5M Litres	-0.5 (-2.1, 1.2)	**-0.07 (-0.1, -0.04)**	**-2.5 (-4.7, -0.4)**	0.003 (-0.06, 0.06)	**-3.0 (-5.7, -0.2)**	-0.007 (-0.1, 0.0001)

Bold indicates a significant difference at the 95% confidence interval level.

Supplementary Table 4b: Modelled absolute and relative change in volume of, and sugar in, all drinks (ml) purchased per household (95% CI) per week in relation to the UK SDIL, March 2014- March 2019; excluding small manufacturers (weighted)

	Analysis 1: pre-post announcement (March 2014 – March 2018)		Analysis 2: pre-post implementation (March 2016 – March 2019)		Analysis 3: pre-post announcement & implementation (March 2014 – March 2019)	
	Absolute change (ml)	Relative change (%)	Absolute change (ml)	Relative change (%)	Absolute change (ml)	Relative change (%)
Volume						
High tier (≥8g sugar per 100ml)						
All Manufacturers	**34.7 (8.1, 61.4)**	**7.3 (1.7, 12.9)**	**-171.6 (-208.1, -135.1)**	**-42.5 (-51.6, -33.5)**	**-140.8 (-177.3, -104.3)**	**-37.8 (-47.6, -28.0)**
Excluding Manufacturers with <1M Litres	**35.3 (8.9, 61.6)**	**7.5 (1.9, 13.1)**	**-168.1 (-204.2, -131.9)**	**-42.6 (-51.7, -33.4)**	**-136.2 (-172.3, -100.1)**	**-37.6 (-47.5, -27.6)**
Excluding Manufacturers with <0.5M Litres	**35.3 (8.9, 61.6)**	**7.5 (1.9, 13.1)**	**-168.1 (-204.2, -131.9)**	**-42.6 (-51.7, -33.4)**	**-136.2 (-172.3, -100.1)**	**-37.6 (-47.5, -27.6)**
Low tier (≥5g - <8g sugar per 100ml)						
All Manufacturers	**-65.7 (-77.5, -53.8)**	**-37.1 (-43.7, -30.4)**	**-71.8 (-87.8, -55.8)**	**-71.8 (-87.8, -55.8)**	**-170.5 (-186.5, -154.5)**	**-85.8 (-93.9, -77.8)**
Excluding Manufacturers with <1M Litres	**-66.4 (-78.2, -54.6)**	**-37.6 (-44.3, -30.9)**	**-71.2 (-87.1, -55.3)**	**-72.5 (-88.7, -56.3)**	**-171.1 (-187.0, -155.2)**	**-86.4 (-94.4, -78.4)**
Excluding Manufacturers with <0.5M Litres	**-66.5 (-78.2, -54.7)**	**-37.6 (-44.2, -31.0)**	**-71.3 (-87.1, -55.5)**	**-72.4 (-88.5, -56.3)**	**-171.2 (-187.1, -155.4)**	**-86.3 (-94.3, -78.3)**
Sugar						
High tier (≥8g sugar per 100ml)						
All Manufacturers	**5.5 (3.8, 7.3)**	**10.8 (7.4, 14.1)**	**-21.2 (-23.8, -18.5)**	**-49.3 (-55.4, -43.1)**	**-16.2 (-18.8, -13.5)**	**-42.6 (-49.6, -35.6)**
Excluding Manufacturers with <1M Litres	**4.7 (1.7, 7.7)**	**9.3 (3.4, 15.1)**	**-19.3 (-23.4, -15.2)**	**-44.3 (-53.6, -35.0)**	**-14.6 (-18.7, -10.6)**	**-37.6 (-48.1, -27.1)**
Excluding Manufacturers with <0.5M Litres	**4.7 (1.7, 7.7)**	**9.3 (3.4, 15.1)**	**-19.3 (-23.4, -15.2)**	**-44.3 (-53.6, -35.0)**	**-14.6 (-18.7, -10.6)**	**-37.6 (-48.1, -27.1)**
Low tier (≥5g - <8g sugar per 100ml)						
All Manufacturers	**-4.3 (-6.1, -2.6)**	**-37.5 (-52.5,-22.5)**	**-5.0 (-7.4, -2.6)**	**-75.8 (-112.7, -38.9)**	**-11.5 (-13.9, -9.07)**	**-87.8 (-106.4, -69.2)**
Excluding Manufacturers with <1M Litres	**-4.7 (-5.5, -3.8)**	**-39.1 (-46.5, -31.7)**	**-4.8 (-6.0, -3.6)**	**-73.0 (-91.3, -54.7)**	**-11.8 (-13.0, -10.6)**	**-86.9 (-95.8, -78.1)**
Excluding Manufacturers with <0.5M Litres	**-4.7 (-5.6, -3.8)**	**-39.1 (-46.5, -31.8)**	**-4.8 (-6.0, -3.6)**	**-72.9 (-91.1, -54.6)**	**-11.8 (-13.0, -10.6)**	**-86.9 (-95.7, -78.0)**

Bold indicates a significant difference at the 95% confidence interval level.

Supplementary Figure 2a. Observed and modelled volume (ml) in all soft drinks combined purchased per household per week, March 2014- March 2019 (weighted)

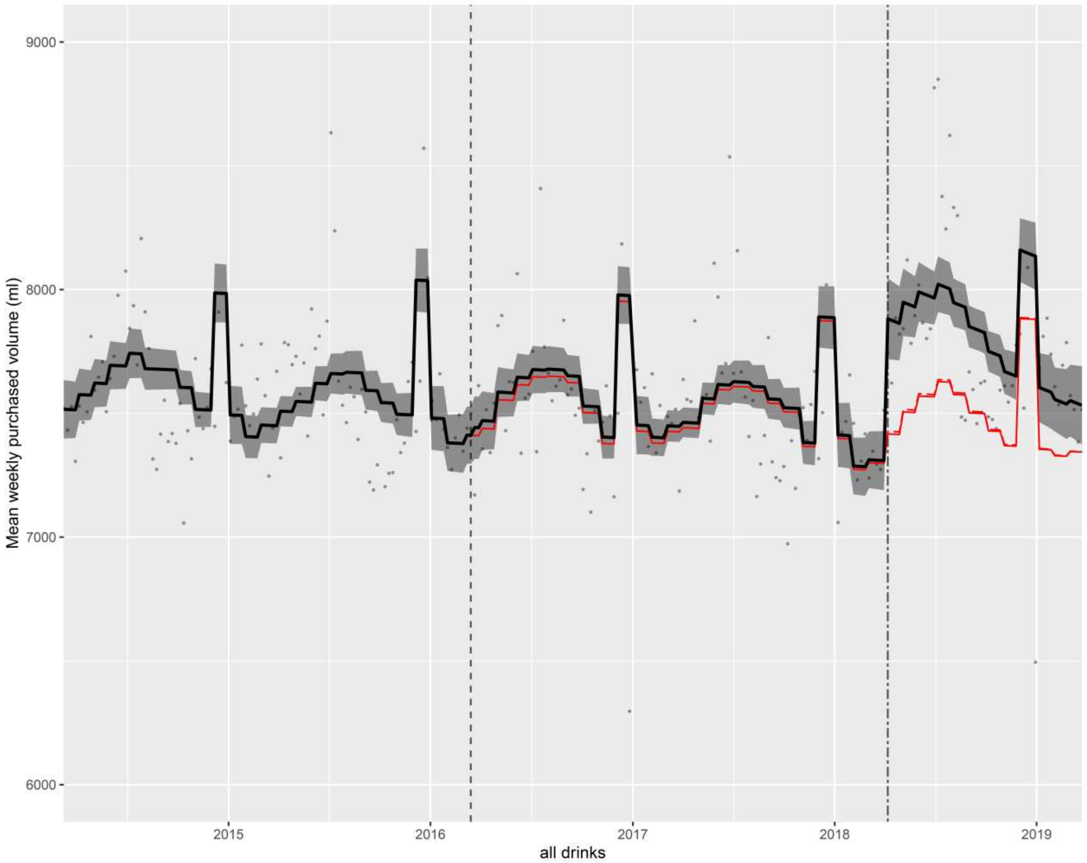

Notes. Points are observed data, black lines (with shadows) are modelled data (with 95% confidence intervals); red lines indicate the counterfactuals had the announcement (red solid line) and implementation (red dashed line) not happened; the first dashed vertical line indicates the point of announcement; the second dashed vertical line indicates the point of implementation; modelled purchases include averaged effects for seasonality and the impact of December and January (the Christmas period). The control category of toiletries is shown in Fig 3.

Supplementary Figure 2b. Observed and modelled amount of sugar (g) in all soft drinks combined purchased per household per week, March 2014- March 2019 (weighted)

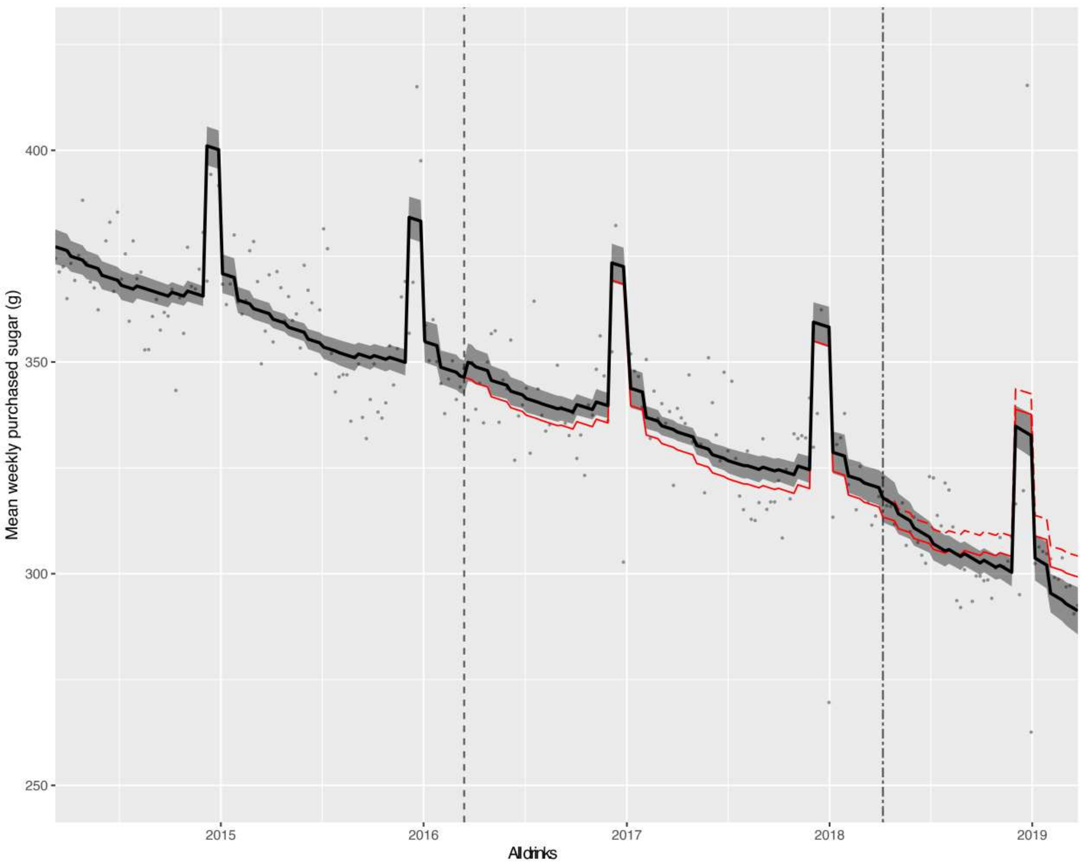

Notes. Points are observed data, black lines (with shadows) are modelled data (with 95% confidence intervals); red lines indicate the counterfactuals had the announcement (red solid line) and implementation (red dashed line) not happened; the first dashed vertical line indicates the point of announcement; the second dashed vertical line indicates the point of implementation; modelled purchases include averaged effects for seasonality and the impact of December and January (the Christmas period). The control category of toiletries is shown in Fig 3.

Supplementary material G

Removing the control category led to wider confidence intervals in a small number of cases such that absolute and relative changes in volume were not significantly different from the pre-implementation counterfactuals for no levy drinks and the pre-announcement and post implementation counterfactual for drinks containing 0g of sugar per 100ml. Significantly lower volumes of purchased volumes of powdered drinks were seen following the announcement in the controlled analysis unlike in the uncontrolled analysis. In the uncontrolled analysis absolute and relative differences in the amount of sugar in milk based drinks were significantly different from the pre-implementation counterfactual but not significantly different when examining the impact of the SDIL overall. Additionally the amount of sugar in confectionery was not significantly different from the pre-announcement counterfactual in the uncontrolled ITS analysis unlike in the controlled analysis.

Supplementary Table 5a: Level and trend changes in volume of drinks (ml) and confectionery (g) purchased per household per week (95% CI) in relation to the UK SDIL, March 2014 - March 2019 without toiletries as a control condition (weighted)

	Analysis 1: pre-post announcement (March 2014 – March 2018)		Analysis 2: pre-post implementation (March 2016 – March 2019)		Analysis 3: pre-post announcement & implementation (March 2014 – March 2019)	
	Level Change	Trend Change	Level Change	Trend Change	Level Change	Trend Change
Levy liable drinks						
High tier (≥8g sugar per 100ml)	42.3 (-9.4, 94.0)	-0.07 (-0.9, 0.8)	**-111.2 (-177.1, -45.3)**	-1.2 (-3.0, 0.7)	-68.9 (-152.7, 14.9)	-1.3 (-3.3, 0.8)
Low tier (≥5g - <8g sugar per 100ml)	-2.2 (-24.1, 19.8)	**-0.6 (-1.0, -0.2)**	-18.9 (-45.8, 8.0)	-1.0 (1.9, -0.2)	-21.1 (-55.75, 13.6)	**-1.7 (-2.6, -0.7)**
No levy (<5g sugar per 100ml)	-29.8 (-150.9, 91.3)	**2.0 (0.08, 4.0)**	**378.1 (223.7, 532.5)**	**0.3 (0.5, 2.0)**	**348.3 (152.0, 544.6)**	2.4 (-2.4, 7.2)
>0g to <5g sugar per 100ml	-27.9 (-78.3, 22.5)	**1.3 (0.5, 2.0)**	**98.0 (32.7, 163.3)**	**2.1 (0.3, 3.9)**	70.1 (-12.4, 152.6)	**3.4 (1.4, 5.4)**
0g sugar per 100ml	-2.0 (-75.5, 71.5)	0.8 (-0.4, 2.0)	**278.9 (185.6, 372.2)**	-1.7 (-4.4, 1.0)	**276.9 (158.2, 395.7)**	-0.9 (-3.9, 2.0)
Bottled water	9.9 (-49.1, 68.9)	-0.4 (-1.4, 0.6)	53.5 (-20.8, 127.8)	-2.6 (-4.7, 0.4)	63.4 (-31.5, 158.2)	**-2.9 (-5.3, -0.6)**
Levy exempt drinks						
Alcoholic drinks	-13.3 (-51.0, 24.4)	-0.03 (-0.4, 0.4)	**212.7 (142.5, 282.9)**	**-5.8 (-7.9, -3.8)**	**199.4 (119.8, 279.1)**	**-5.9 (-8.0, -3.8)**
Milk and milk based drinks*	26.0 (-35.7, 87.7)	**-1.1 (-2.0, -0.09)**	75.7 (-4.5, 155.9)	0.6 (-1.6, 2.8)	**101.7 (0.52, 202.9)**	-0.5 (-2.9, 2.0)
No added sugar fruit juices	-9.9 (-35.0, 15.2)	0.2 (-0.3, 0.6)	**40.9 (10.1, 71.7)**	-0.9 (-1.9, 0.02)	31.0 (-8.73, 70.7)	-0.8 (-1.8, 0.3)
Drinks sold as powders (g)	-3.4 (-9.01, 2.20)	-0.03 (-0.1, 0.06)	5.66 (-1.6, 12.9)	0.07 (-0.1, 0.3)	2.25 (-6.90, 11.4)	0.04 (-0.2, 0.3)
Other categories						
Confectionery (g)	-20.5 (-90.8, 49.8)	0.7 (-1.3, 1.5)	-70.6 (-155.8, 14.6)	**2.2 (0.7, 3.7)**	-91.1 (-201.5, 19.3)	2.3 (-2.2, 6.8)

Bold indicates a significant difference at the 95% confidence interval level.
*Milk comprises drinks in the following categories: semi-skimmed; specific low fat % milk (e.g. 1% fat milk); whole milk; buttermilk; modified milk; other milk; other non-cows milk; rice drink; soya milk. Skimmed milk is excluded from all analysis in this paper due to missing data.

Supplementary Table 5b: Level and trend changes in sugar in drinks and confectionery (g) purchased per household per week (95% CI) in relation to the UK SDIL, March 2014 - March 2019 without toiletries as a control condition (weighted)

	Analysis 1: pre-post announcement (March 2014 – March 2018)		Analysis 2: pre-post implementation (March 2016 – March 2019)		Analysis 3: pre-post announcement & implementation (March 2014 – March 2019)	
	Level Change	Trend Change	Level Change	Trend Change	Level Change	Trend Change
Levy liable drinks						
High tier (≥8g sugar per 100ml)	4.8 (-1.0, 10.6)	-0.008 (-0.1, 0.09)	**-12.1 (-19.5, -4.8)**	-0.2 (-0.4, 0.06)	-7.3 (-16.6, 2.0)	-0.2 (-0.4, 0.07)
Low tier (≥5g - <8g sugar per 100ml)	-0.2 (-1.7, 1.2)	**-0.4 (-0.5, -0.4)**	-1.2 (-2.9, 0.6)	**-0.07 (-0.1, -0.01)**	-1.4 (-3.7, 0.9)	**-0.5 (-0.6, -0.5)**
No levy (<5g sugar per 100ml)	1.7 (-0.2, 3.3)	**0.07 (0.04, 0.1)**	**5.2 (3.2,7.2)**	**0.08 (0.02, 0.1)**	**6.9 (4.4, 9.5)**	**0.2 (0.09, 0.2)**
>0g to <5g sugar per 100ml	1.7 (-0.2, 3.3)	**0.07 (0.04, 0.1)**	**5.2 (3.2,7.2)**	**0.08 (0.02, 0.1)**	**6.9 (4.4, 9.5)**	**0.2 (0.09, 0.2)**
Levy exempt drinks						
Milk and milk based drinks*	2.4 (-0.6, 5.3)	**-0.06 (-0.1, -0.02)**	3.7 (-0.1, 7.5)	0.01 (-0.10, 0.1)	**6.1 (1.3, 10.9)**	-0.05 (-0.2, 0.06)
No added sugar fruit juices	-1.1 (-3.5, 1.4)	0.03 (-0.03, 0.07)	**3.3 (0.3, 6.3)**	**-0.10 (-0.2, -0.01)**	2.3 (-1.6, 6.1)	-0.07 (-0.2, 0.03)
Drinks sold as powders	-1.0 (-3.1, 1.1)	0.01 (-0.02, 0.05)	0.4 (-2.2, 3.0)	-0.01 (-0.1, 0.07)	-0.6 (-3.9, 2.7)	0.001 (-0.09, 0.09)
Other categories						
Confectionery	-11.7 (-51.5, 28.1)	0.05 (-0.8, 0.9)	-42.6 (-90.8, 5.6)	1.3 (-0.4, 3.0)	-54.3 (-116.8, 8.2)	1.3 (-0.5, 3.2)

Bold indicates a significant difference at the 95% confidence interval level.
*Milk comprises drinks in the following categories: semi-skimmed; specific low fat % milk (e.g. 1% fat milk); whole milk; buttermilk; modified milk; other milk; other non-cows milk; rice drink; soya milk. Skimmed milk is excluded from all analysis in this paper due to missing data.

Supplementary Table 5c: Absolute and relative changes in volume of drinks (ml) and confectionery (g) purchased per household per week (95% CI) in relation to the UK SDIL, March 2014 - March 2019 without toiletries as a control condition (weighted)

	Analysis 1: pre-post announcement (March 2014 – March 2018)		Analysis 2: pre-post implementation (March 2016 – March 2019)		Analysis 3: pre-post announcement & implementation (March 2014 – March 2019)	
	Absolute change (ml or g)	Relative change (%)	Absolute change (ml or g)	Relative change (%)	Absolute change (ml or g)	Relative change (%)
Levy liable drinks						
High tier (≥8g sugar per 100ml)	34.4 (-4.5, 73.3)	7.3 (-1.0, 15.6)	**-171.6 (-223.1, -120.0)**	**-42.5 (-55.3, -29.7)**	**-140.9 (-192.5, -89.4)**	**-37.8 (-51.6, -24.0)**
Low tier (≥5g - <8g sugar per 100ml)	**-68.3 (-85.7,-50.9)**	**-38.2 (-47.9, -28.4)**	**-71.4 (-94.1, -48.7)**	**-71.5 (-94.2, -48.8)**	**-171.3 (-193.9, -148.6)**	**-85.8 (-97.1, -74.4)**
No levy (<5g sugar per 100ml)	**187.1 (95.7, 278.5)**	**11.5 (5.9, 17.1)**	**395.0 (273.9, 516.0)**	**19.8 (13.7, 25.9)**	**685.5 (564.4, 806.5)**	**40.2 (33.1, 47.3)**
>0g to <5g sugar per 100ml	**106.4 (68.7, 144.2)**	**17.2 (11.1, 23.3)**	**205.9 (155.5, 256.3)**	**25.6 (19.3, 31.8)**	**374.6 (326.0, 426.8)**	**59.3 (51.4, 67.2)**
0g sugar per 100ml	**80.7 (25.1, 136.4)**	**8.0 (2.5, 13.5)**	**191.9 (118.2, 265.5)**	**16.1 (9.9, 22.3)**	**312.0 (238.4, 385.6)**	**29.1 (22.3, 36.0)**
Bottled water	**-31.3 (-76.3, 13.7)**	**-4.4 (-10.6, 1.91)**	**-76.9 (-136.6, -17.2)**	**-9.89 (-17.6, -2.2)**	**-127.8 (-187.6, -68.1)**	**-15.4 (-22.6, -8.2)**
Levy exempt drinks						
Alcoholic drinks	-16.6 (-62.2, 28.9)	1.0 (-3.6, 1.7)	**-84.9 (-155.8, -14.0)**	**-4.81 (-8.8, -0.8)**	**-103.1 (-174.0, -32.2)**	**-5.8 (-9.8, -1.8)**
Milk and milk based drinks*	**-85.9 (-39.6, 132.2)**	**-2.3 (-3.6, -1.1)**	**106.4 (44.5, 168.3)**	**3.03 (1.3, 4.8)**	-32.8 (-94.6, 29.1)	-0.9 (-2.6, 0.8)
No added sugar fruit juices	5.9 (-13.9, 25.8)	1.2 (-2.9, 5.4)	-6.56 (-32.7, 19.6)	-1.33 (-6.6, 4.0)	6.95 (-19.2, 33.1)	1.5 (-4.0, 6.9)
Drinks sold as powders (g)	**-6.8 (-11.0, -2.57)**	**-6.8 (-10.9, -2.6)**	**9.3 (3.7, 14.9)**	**10.8 (4.3, 17.4)**	0.89 (-4.7, 6.5)	1.0 (-5.0, 6.9)
Other categories						
Confectionery (g)	-9.5 (-72.7, 53.7)	-2.3 (-17.7, 13.0)	40.6 (-42.1, 123.2)	11.9 (-12.3, 36.0)	36.3 (-46.4, 118.9)	10.5 (-13.4, 34.3)

Bold indicates a significant difference at the 95% confidence interval level.
*Milk comprises drinks in the following categories: semi-skimmed; specific low fat % milk (e.g. 1% fat milk); whole milk; buttermilk; modified milk; other milk; other non-cows milk; rice drink; soya milk. Skimmed milk is excluded from all analysis in this paper due to missing data.

Supplementary Table 5d: Absolute and relative changes in sugar in drinks and confectionery (g) purchased per household per week (95% CI) in relation to the UK SDIL, March 2014 - March 2019 without toiletries as a control condition (weighted)

	Analysis 1: pre-post announcement (March 2014 – March 2018)		Analysis 2: pre-post implementation (March 2016 – March 2019)		Analysis 3: pre-post announcement & implementation (March 2014 – March 2019)	
	Level Change	Trend Change	Level Change	Trend Change	Level Change	Trend Change
Levy liable drinks						
High tier (≥8g sugar per 100ml)	3.9 (-0.4, 8.3)	7.6 (-0.78, 15.9)	**-19.6 (-25.3, -13.8)**	**-43.8 (-56.6, -30.9)**	**-16.1 (-21.8, -10.3)**	**-39.0 (-52.9, -25.0)**
Low tier (≥5g - <8g sugar per 100ml)	**-4.9 (-6.1, -3.8)**	**-40.3 (-49.6, -30.9)**	**-4.7 (-6.2, -3.2)**	**-70.9 (-93.3, -48.4)**	**-11.9 (-13.4, -10.4)**	**-86.0 (-96.8, -75.2)**
No levy (<5g sugar per 100ml)	**6.1 (4.9, 7.3)**	**77.7 (62.6, 92.8)**	**9.3 (7.8, 10.9)**	**52.5 (43.6, 61.4)**	**19.2 (17.6, 20.8)**	**240.9 (221.0, 260.8)**
>0g to <5g sugar per 100ml						
Levy exempt drinks						
Milk and milk based drinks*	**-4.2 (-6.4, -2.0)**	**-2.3 (-3.6, -1.1)**	**4.1 (1.1, 7.0)**	**2.4 (0.7, 4.2)**	**-3.2 (-6.5,-0.3)**	**-1.8 (-3.5, -0.1)**
No added sugar fruit juices	**2.6 (0.7, 4.6)**	**5.9 (1.5, 10.2)**	-1.8 (-4.3, 0.8)	-3.7 (-9.1, 1.6)	**2.6 (0.04, 5.2)**	**5.9 (0.08, 11.8)**
Drinks sold as powders (g)	0.4 (-1.2, 2.1)	2.1 (-5.9, 10.0)	-0.3 (-2.4, 1.9)	-1.3 (-12.5, 10.0)	0.9 (-1.3,3.0)	4.7 (-7.2, 16.7)
Other categories						
Confectionery (g)	-5.9 (-42.0, 30.1)	-2.6 (-18.1, 13.0)	23.4 (-24.0, 70.7)	12.1 (-12.4, 36.7)	20.2 (-27.1, 67.6)	10.3 (-13.9, 34.5)

Bold indicates a significant difference at the 95% confidence interval level.
*Milk comprises drinks in the following categories: semi-skimmed; specific low fat % milk (e.g. 1% fat milk); whole milk; buttermilk; modified milk; other milk; other non-cows milk; rice drink; soya milk. Skimmed milk is excluded from all analysis in this paper due to missing data.
