# Supplementary material for: Changes in soft drinks purchased by British households associated with the UK soft drinks industry levy: a controlled interrupted time series analysis
Source: BMJ Open. 2023 Dec 4;13(12):e077059. doi: 10.1136/bmjopen-2023-077059 (PMC10711915; doi:10.1136/bmjopen-2023-077059)
Supplement: Author's manuscript [file bmjopen-2023-077059.draft_revisions.pdf]

BMJ Open

This article is a corrected version of [Pell 2021 published in The BMJ], which has now been retracted. The previous versions of the manuscript along with the* peer reer review *of Pell 2021, which informed the publication of the* BMJ Open *article, are available here https://www.bmj.com/content/372/bmj.n254/peer-review.

BMJ Open is committed to open peer review. As part of this commitment we make the peer review history of every article we publish publicly available.

When an article is published we post the peer reviewers' comments and the authors' responses online. We also post the versions of the paper that were used during peer review. These are the versions that the peer review comments apply to.

The versions of the paper that follow are the versions that were submitted during the peer review process. They are not the versions of record or the final published versions. They should not be cited or distributed as the published version of this manuscript.

BMJ Open is an open access journal and the full, final, typeset and author-corrected version of record of the manuscript is available on our site with no access controls, subscription charges or pay-per-view fees (http://bmjopen.bmj.com).

If you have any questions on BMJ Open's open peer review process please email info.bmjopen@bmj.com

BMJ Open

Changes in soft drinks purchased by British households associated with the UK soft drinks industry levy: controlled interrupted time series analysis

Journal:	*BMJ Open*
Manuscript ID	bmjopen-2023-077059
Article Type:	Original research
Date Submitted by the Author:	26-Jun-2023
Complete List of Authors:	Rogers , Nina ; MRC Epidemiology Unit<br>Pell, David; MRC Epidemiology Unit<br>Mytton, Oliver; MRC Epidemiology Unit; University College London Great Ormond Street Institute of Child Health<br>Penney, Tarra; York University - Keele Campus<br>Briggs, Adam; University of Oxford, Nuffield Department of Population Health; University of Warwick Warwick Medical School, Institute for Health Policy and Clinical Practice<br>Cummins, Steven; London School of Hygiene & Tropical Medicine, Department of Social and Environmental Health Research<br>Penn-Jones, Catrin; MRC Epidemiology Unit<br>Rayner, Mike; University of Oxford, Nuffield Department of Population Health; NIHR Oxford Biomedical Research Centre<br>Rutter, Harry; University of Bath Department of Social and Policy Sciences<br>Scarborough, Peter; Centre on Population Approaches for Non-Communicable Disease Prevention, Nuffield Department of Population Health; NIHR Oxford Biomedical Research Centre<br>Sharp, Stephen; MRC Epidemiology Unit<br>Smith, Richard ; University of Exeter,<br>White, Martin; MRC Epidemiology Unit<br>Adams, J; MRC Epidemiology Unit
Keywords:	Obesity, PUBLIC HEALTH, NUTRITION & DIETETICS

SCHOLARONE™
Manuscripts

I, the Submitting Author has the right to grant and does grant on behalf of all authors of the Work (as defined in the below author licence), an exclusive licence and/or a non-exclusive licence for contributions from authors who are: i) UK Crown employees; ii) where BMJ has agreed a CC-BY licence shall apply, and/or iii) in accordance with the terms applicable for US Federal Government officers or employees acting as part of their official duties; on a worldwide, perpetual, irrevocable, royalty-free basis to BMJ Publishing Group Ltd ("BMJ") its licensees and where the relevant Journal is co-owned by BMJ to the co-owners of the Journal, to publish the Work in this journal and any other BMJ products and to exploit all rights, as set out in our licence.

The Submitting Author accepts and understands that any supply made under these terms is made by BMJ to the Submitting Author unless you are acting as an employee on behalf of your employer or a postgraduate student of an affiliated institution which is paying any applicable article publishing charge ("APC") for Open Access articles. Where the Submitting Author wishes to make the Work available on an Open Access basis (and intends to pay the relevant APC), the terms of reuse of such Open Access shall be governed by a Creative Commons licence – details of these licences and which Creative Commons licence will apply to this Work are set out in our licence referred to above.

Other than as permitted in any relevant BMJ Author's Self Archiving Policies, I confirm this Work has not been accepted for publication elsewhere, is not being considered for publication elsewhere and does not duplicate material already published. I confirm all authors consent to publication of this Work and authorise the granting of this licence.

For peer review only

Changes in soft drinks purchased by British households associated with the UK soft drinks industry levy: controlled interrupted time series analysis

Nina T Rogers, research associate[1]

David Pell, research associate[1(when this work was conducted)]

Oliver Mytton, clinical lecturer[1(when this work was conducted), 9(currently)]

Tarra L Penney, assistant professor[1,2]

Adam Briggs, honorary associate clinical professor[3,4]

Steven Cummins, professor[5]

Catrin Jones, research associate[1]

Mike Rayner, professor[3,6]

Harry Rutter, professor[7]

Peter Scarborough, professor[3,6]

Stephen J Sharp, senior statistician[1]

Richard D Smith, professor[8]

Martin White, professor[1]

Jean Adams, professor [1]

[1]MRC Epidemiology Unit, University of Cambridge School of Clinical Medicine, Institute of Metabolic Science, Cambridge CB2 0QQ, UK

[2]Faculty of Health, School of Global Health, York University, Toronto, Canada

[3]Nuffield Department of Population Health, University of Oxford, Oxford, UK

[4]Warwick Medical School, University of Warwick, Division of Health Sciences, Coventry, UK

[5]Population Health Innovation Lab, Department of Public Health, Environment and Society, London School of Hygiene and Tropical Medicine, London, UK

[6]National Institute of Health Research Oxford Biomedical Research Centre, Oxford University Hospitals NHS Foundation Trust, Headington, Oxford, UK

[7]Department of Social and Policy Sciences, University of Bath, Bath, UK

[8]College of Medicine and Health, University of Exeter, Exeter, UK

[9]Great Ormond Street Institute of Child Health, University College London, London, UK

Correspondence to: J Adams jma79@medschl.cam.ac.uk (or @jeanmadams on Twitter)

For peer review only

For peer review only - http://bmjopen.bmj.com/site/about/guidelines.xhtml

Abstract

Objective To determine changes in household purchases of drinks one year after implementation of the UK soft drinks industry levy (SDIL).
Design Controlled interrupted time series analysis.
Participants Households reporting their purchasing to a market research company (average weekly n=22,091), March 2014-March 2019.
Intervention A two tiered tax levied on manufacturers of soft drinks, announced in March 2016 and implemented in April 2018. Drinks with ≥8g sugar/100mL (high tier) are taxed at £0.24/L, drinks with ≥5 to <8g sugar/100mL (low tier) are taxed at £0.18/L.
Main outcome measures Absolute and relative differences in the volume of, and amount of sugar in, soft drinks categories, all soft drinks combined, alcohol, and confectionery purchased per household per week one year after implementation.
Results In March 2019, compared with the counterfactual, purchased volume of high tier drinks decreased by 140.8mL (95% confidence interval 104.3-177.3mL) per household per week, equivalent to 37.8% (47.6-28.0%), and sugar purchased in these drinks decreased by 16.2g (13.5-18.8g), or 42.6% (35.6-49.6%). Purchases of low tier drinks decreased by 170.5mL (154.5-186.5mL) or 85.8% (77.8-93.9%), with an 11.5g (9.1-13.9g) reduction in sugar in these drinks, equivalent to 87.8% (69.2-106.4%). When all soft drinks were combined irrespective of levy tier or eligibility, the volume of drinks purchased increased by 188.8mL (30.7-346.9mL) per household per week, or 2.6% (0.4-4.7%), but sugar decreased by 8.0g (2.4-13.6g), or 2.7% (0.8-4.5%). Purchases of confectionery and alcoholic drinks did not increase.
Conclusions Compared with trends before the SDIL was announced, one year after implementation, the volume of soft drinks purchased increased by 189mL, or 2.6% per household per week. The amount of sugar in those drinks was 8g, or 2.7%, lower per household per week. The SDIL might benefit both public health and industry.
Trial registration ISRCTN18042742.

Strengths and limitations

- we used a large, nationally representative dataset, included a control category, and explored changes in two potential substitute categories (alcohol and confectionery)
- we only included purchases brought into homes
- we did not assess changes in other categories beyond soft drinks, alcohol, and confectionery
- the estimate of effect size in interrupted time series analyses is based on a modelled counterfactual that might be inaccurate
- attribution of effects in interrupted time series analyses is vulnerable to time varying confounding including co-interventions

For peer review only

For peer review only - http://bmjopen.bmj.com/site/about/guidelines.xhtml

Introduction

High consumption of sugar sweetened beverages (SSBs) is associated with increased risk of dental caries, obesity, type 2 diabetes, and cardiovascular disease.[1-3] The World Health Organization recommends the use of SSB taxes to reduce consumption.[4] A systematic review of studies published to June 2018 suggests that SSB taxes lead to decreases in the sales, purchasing and consumption of taxed drinks.[5] More recent findings support this conclusion.[6-10] Although price is one important mediator of these changes,[11-16] other potential mechanisms include reformulation of products to reduce sugar concentration, smaller portion sizes, and increases in the perception of SSBs being harmful to health associated with them being grouped with other taxed products such as alcohol and tobacco.[17] Furthermore, any public health benefits of reduced SSB consumption associated with SSB taxes might be negated by increased consumption of substitutes such as confectionery and alcohol.[18-20]

The UK soft drinks industry levy (SDIL) was one of the first taxes on SSBs explicitly designed to incentivise manufacturers of SSBs to reduce sugar content.[21 22] This is reflected in three design features. Firstly, the SDIL is levied on manufacturers, importers, and bottlers rather than on consumers. Secondly, the levy includes two tiers: £0.24/L for drinks containing ≥8 g total sugar per 100 mL, and £0.18/L for drinks containing ≥5 g and <8 g total sugar per 100 mL. Thirdly, the SDIL was intentionally announced in 2016, two years before implementation in 2018, to allow manufacturers time to adjust. The SDIL also provides exemptions (see box 1).[23]

Box 1 Glossary of terms

Soft drinks industry levy (SDIL)—a tiered tax on manufacturers of sugar sweetened beverages

Levy exempt drinks—drinks exempt from the SDIL irrespective of sugar content; that is, drinks containing >75% milk, drinks containing >1.2% alcohol, and drinks sold as alcohol replacements, drinks sold as powders, 100% fruit juices, and drinks sold by manufacturers selling less than one million litres of drinks not exempt for other reasons each year

High tier drinks—drinks that are not levy exempt and contain ≥8 g of sugar per 100 mL

Low tier drinks—drinks that are not levy exempt and contain ≥5 g to <8 g of sugar per 100 mL

No levy drinks—drinks that are not levy exempt but contain <5 g of sugar per 100 mL; we subdivided this category into drinks containing >0 g to <5 g of sugar per 100 mL, drinks containing 0 g of sugar per 100 mL, and bottled water

Levy liable drinks—drinks that are not levy exempt drinks; that is, the sum of high tier drinks, low tier drinks, and no levy drinks

Soft drinks—any drink not containing alcohol

Confectionery—products in the sugar confectionery and chocolate confectionery categories

Toiletries—products in the shampoo, hair conditioner, and liquid soap categories

For peer review only - http://bmjopen.bmj.com/site/about/guidelines.xhtml

Two before and after analyses have shown reductions of around 30% in sales weighted sugar concentration of levy eligible drinks in the UK from before the announcement of the SDIL on 16 March 2016 to after implementation on 6 April 2018.[24 25] However, background trends in purchases of sugary drinks are not stable, with decreases reported over several years.[26] This makes it difficult to attribute before and after decreases in sugary drinks purchases to the SDIL. An interrupted time series analysis found that the announcement and implementation of the SDIL were together associated with a 34 percentage point reduction in the proportion of levy liable drinks with >5 g total sugar per 100 mL, indicating substantial reformulation of the market.[16] Changes in prices across the UK soft drink market were also reported, although it was difficult to discern clear patterns in these, with some levied categories increasing and others decreasing in price. In a controlled interrupted time series analysis including data up to the point of SDIL implementation, we found that the SDIL announcement was associated with changes in both the volume of, and sugar purchased in, drinks in many categories.[27] Overall we found no change in total volume of purchases of all soft drinks combined, but a small increase in sugar purchased from soft drinks of 5.3g per household per week, or 1.7%.

We determined whether household purchases of drinks and confectionery had changed one year after implementation of the SDIL.

Methods

Here we extend our previous analyses[27] to study changes in the volume of, and amount of sugar in, household purchases of drinks in each levy tier, exempt drinks categories (including alcoholic drinks), and confectionery from two years before the announcement of the SDIL to one year after its implementation (March 2014 to March 2019). As before, we used controlled interrupted time series methods, with toiletries included as a control category.[27] We compared observed changes associated with the announcement and implementation of the SDIL to the counterfactual scenarios in which the announcement and implementation did not take place. Including a full two years of data before the announcement enables us to estimate pre-intervention trends and project these forward as counterfactual scenarios. The protocol is published elsewhere[28] and the study was registered. This study is reported in accordance with the strengthening the reporting of observational studies in epidemiology (STROBE) guideline (see supplementary material A).

For peer review only - http://bmjopen.bmj.com/site/about/guidelines.xhtml

Data source

We used data from a panel of households reporting their purchasing on a weekly basis to a market research company (Kantar Worldpanel; KWP). Participating households are asked to record all food and drink purchases brought into the home (including those ordered online and delivered) through barcodes scanners and manual report. Purchasing information is uploaded weekly, where it is linked to nutritional data collected by KWP field workers on a rolling basis. Households record their personal characteristics and receive gift vouchers worth about £100 ($122; €112) annually—equivalent to 0.3% of median UK annual household income after tax in 2019 (£29 600).[29]

KWP samples households from across Great Britain (GB) using proprietary methods, aiming to achieve a sample that is demographically representative of GB households. It excludes households that record fewer than six purchases weekly along with those whose adjusted weekly spend is lower than an undisclosed minimum. KWP applies proprietary weights to purchases to adjust for these exclusions and maintain the representativeness of the panel. We used these weights throughout.

The main data cleaning that occurred before analysis involved assigning products and product groups in the KWP dataset to SDIL relevant groups. This was done based on KWP assigned product groups, product names, and nutritional content. In previous work we found some evidence of error, but not bias, in the sugar concentration reported by KWP compared with information provided on manufacturers' websites.[27]

Product categories: drinks, confectionery, and toiletries

Purchased drinks that were levy liable were divided into high tier, low tier, or no levy based on sugar content (see box 1 for definitions). No levy drinks were additionally disaggregated, as described in box 1.

As the SDIL might have led to substitution to other drinks categories, we also examined purchasing of levy exempt drinks in several categories: milk based drinks (comprising milk, milk alternatives such as soya drinks, and yoghurt based juices and drinks), alcoholic drinks (comprising both alcoholic and alcohol replacement drinks), no added sugar fruit juices, and drinks sold as powder (eg, tea, coffee, hot chocolate). Other exempt categories (infant formulas and drinks sold for medical purposes) were excluded.

We also hypothesised that the SDIL might lead to substitution from sugary drinks to other high sugar categories. To investigate this, we used sugar and chocolate confectionery purchases (referred to as confectionery).

For peer review only - http://bmjopen.bmj.com/site/about/guidelines.xhtml

For peer review only

Control group

To control for background trends in household purchases we used purchases of shampoo, hair conditioner, and liquid soap (ie, toiletries). Toiletries meet the proposed criteria for a controlled interrupted time series: they are robust to seasonality and may have similar purchase volumes by households regardless of socioeconomic position or other potential confounders.[30]

Outcome measures

Most evaluations of SSB taxes focus on volume of drinks purchased. However, the SDIL's focus on reformulation makes the sugar purchased in drinks of additional public health interest. Thus, the outcome measures of interest were mean volume purchased per household per week in each of the drink categories and grams per household per week of confectionery; and mean sugar purchased per household per week from each of the drink categories and confectionery. Data were aggregated at the weekly level and analysed as a time series.

Overall analysis strategy

Previous evidence indicates that reformulation occurred after the announcement of the SDIL and price changes after implementation.[16] As such, we hypothesised the SDIL might act as two linked interventions: the announcement on 16 March 2016 and implementation on 6 April 2018.[17] Thus, our analysis strategy involved three separate comparisons that isolate the announcement and implementation of the SDIL and then examine the combined effect (fig 1). In the first analysis we isolated the announcement of the SDIL. Here we compared anticipatory effects on purchasing two years after the announcement to the counterfactual estimated from purchasing in the two years before the announcement. This replicates and updates our previous analysis[27] as we anticipate that the stabilising effect of including additional post-announcement data likely reduces error. In the second analysis, we isolated the implementation of the SDIL. Here we compared purchasing one year after implementation to the counterfactual estimated from purchasing in the four years before implementation. In the third analysis we considered both the announcement and the implementation and we compared purchasing one year after implementation (ie three years after announcement) to the counterfactual estimated from purchasing in the two years before the announcement.

Throughout, we used the proprietary weights provided by KWP.

For peer review only - http://bmjopen.bmj.com/site/about/guidelines.xhtml

Primary analysis: category specific analyses

For each of the three analyses we developed separate controlled interrupted time series models for volume and sugar purchased from each levy liable and levy exempt drinks category and confectionery (fig 1). Supplementary material B provides the full model specification.

We present absolute and relative differences between observed purchasing and counterfactual scenarios in the final week of each observation period, with standard errors used to calculate 95% confidence intervals for the relative difference obtained using the delta method.[31]

Secondary analysis: all soft drinks categories combined, irrespective of levy eligibility

Levy exempt drinks include drinks that might contain comparable amounts of sugar to levy liable products. To examine the extent to which the SDIL impacted on the purchased volume of, and amount of sugar in, soft drinks, regardless of SDIL liability, we carried out controlled interrupted time series analysis, combining purchases of all soft drinks irrespective of sugar content (ie, high tier, low tier, no levy, milk and milk based drinks, no added sugar fruit juice, and drinks sold as powders), levy liable drinks irrespective of sugar content (ie, high tier, low tier, and no levy drinks), and according to sugar content based on levy tiers irrespective of levy eligibility (ie, all soft drinks with ≥8 g of sugar per 100 mL, all soft drinks with ≥5 g to <8 g of sugar per 100 mL, and all soft drinks with <5 g of sugar per 100 mL).

Sensitivity analysis: excluding small manufacturers

The SDIL exempts drinks from manufacturers and producers who sell less than one million litres of levy liable drinks annually. As we were unable to obtain a list of exempt manufacturers, our main analyses include all manufacturers. We conducted sensitivity analyses to examine the effect of excluding manufacturers who we estimated to be small. The total purchase volume was summed by manufacturer by year across the five years in the KWP dataset, and a mean purchase volume per year for each manufacturer was calculated. In the first sensitivity analysis, we excluded manufacturers with a mean of less than one million litres purchased per year. Acknowledging KWP data excludes purchases not brought home, we repeated these analyses excluding manufacturers with mean annual purchased volumes of <0.5 million litres in KWP. We were unable to access accurate estimates of the proportion of all drinks purchases brought home. This value reflects an arbitrary, but we think conservative, estimate of the minimum proportion of drinks brought home.

For peer review only - http://bmjopen.bmj.com/site/about/guidelines.xhtml
For peer review only

Sensitivity analysis: interrupted time series without a control category

Toiletries were chosen as a control condition a priori to account for background trends in household purchases. It is, however, possible that a more appropriate control exists. As we only have access to data on purchasing of the categories described here (confectionery, drinks, toiletries), we were not able to examine alternative potential control categories. To examine the effect of the decision to use toiletries as the control category, we performed an additional sensitivity analysis with no control condition.

Changes to protocol

We made several changes to the published protocol.[28] KWP provided additional data that allowed us to increase the precision of our estimates. Specifically, we were able to increase the pre-announcement study period from 104 to 107 weeks and reduce the unit of analysis from purchases every four weeks to purchases every week. We originally intended to include purchases not brought home. We excluded these purchases, however, as these data were not available before mid-2015, meaning that robust pre-announcement trends could not be estimated. Although we originally intended to combine all no levy drinks, we present these disaggregated into those with >0 g and <5 g of sugar per 100 mL, 0 g of sugar per 100 mL, and bottled water, as trends for these different categories are noticeably different. Our original intention to explore potential disparities across socioeconomic groups will be pursued in future work.

Patient and public involvement

The steering group for the wider SDIL evaluation includes two lay members and meets twice a year. Patients and the public were not involved in developing the research question, the outcome measures, the design, or the conduct of the work reported here. The steering group has regularly contributed ideas for routes to dissemination.

Results

About 31 million purchases of drinks, confectionery, and toiletries from March 2014 to March 2019 were included from a mean of 22 091 households each week. The characteristics of included households remained consistent over the study period, and after weighting they largely reflected households in 2014-19 in the UK (see supplementary table 1).

Table 1 summarises households' weekly purchased volumes of, and amounts of sugar in, drinks and other categories over the study period. Substantial reductions in volume of, and sugar in, purchases of SDIL liable drinks were observed in the high and low tiers over time.

For peer review only - http://bmjopen.bmj.com/site/about/guidelines.xhtml
For peer review only

These reductions were accompanied by a smaller increase in volume of no levy drinks purchased, but proportionally much greater increases in sugar purchased in these drinks.

Primary analysis: category specific results

Results of the controlled interrupted time series analyses of purchased volume of, and sugar in, levy liable drinks and confectionery are shown in figure 2 (volume) and figure 3 (sugar). Absolute and relative changes are summarised in tables 2 and 3. Supplementary tables 2a and b show level and trend changes from these models. Supplementary figures 1a and b show similar figures and data for subcategories of no levy drinks and exempt categories.

High tier drinks

The trend in purchased volume of, and sugar in, high tier drinks continued downwards throughout the study period. The announcement of the SDIL was associated with an increase in purchased volume of (34.7ml (95% confidence intervals 8.1 to 61.4ml, or 7.3% (1.7 to 12.9%)), and sugar in (5.5g (3.8 to 7.2), or 10.8% (7.4 to 14.1%)), these drinks. In contrast, the implementation of the SDIL was associated with a reduction in purchased volume of, and sugar in, these drinks. The volume of high tier drinks purchased was 171.6 mL (135.1 to 208.1mL) per household per week, or 42.5% (33.5% to 51.6%), lower in March 2019 compared with the counterfactual estimated from pre-implementation trends. The reductions associated with implementation outweighed the increases associated with announcement, such that the intervention as a whole was associated with a decrease in purchased volume of 140.8ml (104.3 to 177.3ml) per household per week or 37.8% (28.0 to 47.6%) and sugar of 16.2 g (13.5 to 18.8g) per household per week or 42.6% (35.6% to 49.6%) from these drinks.

Low tier drinks

Purchased volume of, and sugar in, low tier drinks gradually increased before the announcement of SDIL. The announcement was associated with a reversal of this trend. There were reductions in purchased volume of, and sugar in, low tier drinks associated with announcement, implementation and the whole intervention. Compared with the counterfactual estimated from pre-announcement trends, in March 2019 the volume of purchased low tier drinks per household per week decreased by 170.5 mL (154.5 to 186.5 mL), or 85.8% (77.8 to 93.9%); and sugar purchased in these drinks decreased by 11.5 g (9.1 to 13.9g) per household per week, or 87.8% (69.2 to 106.4%).

For peer review only - http://bmjopen.bmj.com/site/about/guidelines.xhtml

No levy drinks

Before the announcement of the SDIL there was a gradual upward trend in volume of purchased no levy drinks but a gradual downward trend in purchased sugar. Announcement, implementation and the whole intervention were associated with increases in volume of no levy drinks purchased as well as sugar purchased from those drinks. Overall, purchased volume of no levy drinks in March 2019 was 685.5 mL (599.8 to 771.1mL) higher, equivalent to 40.2% (35.2% to 45.2%) increase compared with the counterfactual of pre-announcement trends. Equivalent figures for sugar purchased from no levy drinks were a 19.2g (16.7 to 21.6g) per household per week, equivalent to 242.8% (211.9 to 273.7%), increase.

Changes in purchased volume of subcategories within the no levy drinks group were not uniform and the overall increase in both volume and sugar purchased in this category was driven by low and no sugar drinks, rather than bottled water. The implementation, but not the announcement, of the SDIL were associated with significant decreases in bottled water purchased which led to an overall decrease in volume of bottled water purchased as a result of the whole intervention of 130.5ml (88.8 to 174.1ml) per household per week, or 15.7% (10.4 to 20.9%). In contrast, the implementation and the announcement of the SDIL were associated with increases in volume of purchased drinks with no sugar and with >0 to <5 g total sugar per 100 mL, and increases of sugar in drinks with >0 to<5g sugar per 100ml.

Levy exempt drinks and confectionery

Overall, the combined announcement and implementation of the SDIL were associated with decreases in purchased volume of alcoholic and milk and milk-based drinks, but no change in sugar purchased from levy exempt categories or from confectionery. Compared with the counterfactual of pre-announcement trends, in March 2019 volume of alcoholic drinks purchased decreased by 103.1ml (53.0 to 153.3ml) per household per week, equivalent to a 5.8% (3.0 to 8.6%) reduction; and volume of milk and milk based drinks purchased decreased by 132.8ml (51.7 to 213.9ml), equivalent to a 3.6% (1.4 to 5.7%) reduction.

Secondary analysis: all soft drinks categories combined

Supplementary table 3a and supplementary figure 2 summarise the results of the controlled interrupted time series analyses of the associated effects of the SDIL on purchased volume of, and sugar from, all soft drinks categories combined, irrespective of levy eligibility. Supplementary table 3b summarises absolute and relative changes in volume of, and sugar in, all soft drinks and confectionery purchased.

For peer review only - http://bmjopen.bmj.com/site/about/guidelines.xhtml

Overall, compared with the counterfactual estimated from pre-announcement trends, a small increase was observed in volume of all soft drinks purchased in March 2019 of 188.8ml (30.7 to 346.9ml) per household per week, equivalent to a 2.6% (0.4 to 4.7%) increase. A reduction was, however, found in sugar purchased in all soft drinks (including exempt drinks) combined of 8.0g per household per week (2.4 to 13.6g), equivalent to 2.7% (0.8 to 4.5%).

Sensitivity analyses

Excluding manufacturers of levy liable products with less than one million and less than 500 000 litres of purchased drinks annually in our dataset was associated with small changes in the magnitude of estimated coefficients, but with no change in the direction or statistical significance of any findings (supplementary tables 4a to b).

In general, removing the control category led to minor changes in effect estimates but wider confidence intervals (see supplementary material G).

Discussion

Taking account of pre-existing pre-announcement trends, this study found that one year after implementation of the SDIL, sugar purchased from soft drinks that were taken home decreased by 8.0 g per household per week (or 2.7%), whilst volume increased by 188.8 mL per household per week (or 2.6%). Assuming a mean UK household size of 2.4 people,[32] this is equivalent to a reduction in sugar from SSBs of 3.3 g per person per week and an increase in volume of 79 mL per person per week, or equivalent to the replacement of 66 mL of a drink with 5 g sugar per 100 mL per person per week with 145 mL of a sugar-free alternative. A modelling study conducted before implementation of the SDIL found that if the levy achieved reformulation it could be expected to lead to a decrease in sugar consumption from SSBs (from all sources, not just for consumption at home) of 7-38 g per person per week and that this would be associated with a reduction in the number of obese individuals in the UK of 0.2-0.9% and a reduction in incidence cases of type 2 diabetes of -2.0 to 31.1 per 1000 person years.[34] The reduction in sugar from SSBs we report one year after implementation of the SDIL is around half of these lower effect estimates.

Strengths and weaknesses of this study

In this study we used a large, nationally representative dataset, included a control category, and explored changes in two potential substitute categories (alcohol and confectionery).

We only included purchases brought into homes. Although KWP also collects data on other purchases, this smaller panel was established in mid-2015 and so was unsuitable for our

For peer review only - http://bmjopen.bmj.com/site/about/guidelines.xhtml

analyses because robust pre-announcement trends could not be estimated. KWP data are collected at the household level and do not take account of waste or differential sharing within households. Nevertheless, the data provide a reasonable estimate of consumption.[33] We did not assess changes in other categories beyond soft drinks, alcohol, and confectionery.

The estimate of effect size in interrupted time series analyses is based on a modelled counterfactual that might be inaccurate. For example, the strong downward trend in higher tier drinks before the announcement of SDIL might not have continued. Attribution of effects in interrupted time series analyses is vulnerable to time varying confounding including co-interventions. The SDIL is part of a wider sugar reduction strategy, although this has been found to have achieved minimal changes beyond those attributable to the SDIL.[24]

The personal characteristics of the panel remained similar over the study period, and proprietary weightings were used to account for non-consumers and to adjust for variations in panel composition. Households participating in KWP are slightly more likely to be from lower social grades and to have no qualifications compared with UK households generally. This might reflect the relative value placed on the small rewards for participation by different households and could limit the generalisability of our findings. If households from lower socioeconomic backgrounds are more likely to change purchasing as a result of the SDIL, then we could have marginally overestimated the effect of the SDIL. However, while we previously found that the price of soft drinks in the UK did change after implementation of the SDIL, no clear pattern was found, with the price of some groups of drinks increasing and others decreasing.[16] We previously found no systematic differences between the sugar content of drinks reported in KWP data and contemporaneous values listed on supermarket websites.[27]

Comparison with other work

Our finding that the SDIL was associated with a reduction in purchased sugar from all soft drinks is consistent with previous analyses that focused on the SDIL.[24,25] Although our estimate of the reduction in sugar consumption from all soft drinks associated with the levy (2.7%) is less than that estimated by others (29%)[24] this previous estimate did not take account of pre-existing trends which we have demonstrated were on a steep downward trajectory for high tier drinks.

We found that the reduction in purchased sugar from all soft drinks alongside a 2.6% increase in volume of all soft drinks purchased. This is consistent with previously reported reductions in the sugar concentration of drinks associated with the SDIL.[16] However, the

For peer review only

For peer review only - http://bmjopen.bmj.com/site/about/guidelines.xhtml

estimated effect size is below the range of reformulation scenarios modelled before implementation (ie, a reduction of 17 to 90 g of sugar per household per week).[34] This difference may be, at least partly, attributable to our focus on drinks taken home versus the modelling study's focus on all drinks.

Evaluations of other SSB taxes have revealed a consistent trend of reductions in purchasing of taxed drinks and no change in purchasing of untaxed drinks.[5] We found similar with both volume of, and sugar in, high and low tier drinks decreasing overall. However, these reductions in volume of taxed drinks were more than offset by increases in volume of no levy drinks purchased. Despite some increases in sugar purchased in no levy drinks, these did not offset decreases in sugar purchased from high and low tier drinks. The SDIL is relatively unique in being explicitly designed to encourage reformulation and there is evidence that substantial reformulation occurred.[16] We are not able to determine from our findings whether the changes we report are due to changes in consumer preference, formulation, or both.

Meaning of the study and implications for policymakers

Our main findings are that the SDIL was associated with a reduction in purchased sugar from all soft drinks with evidence of an increase in the total volume of soft drinks purchased. Given the reformulation associated with the SDIL already documented,[16] it is probable that the changes we report were driven by reductions in the sugar concentration of available drinks, alongside consumers switching to and, indeed increasing consumption of, lower sugar alternatives. Despite the overall reduction we found in sugar purchased in soft drinks, the average amount of sugar purchased in drinks in the no levy group paradoxically increased after implementation of the SDIL, with many drinks that previously had sugar concentrations above the levy threshold now having them just below the threshold. This seems to reflect manufacturers reformulating to target thresholds. Lowering the threshold sugar concentration at which drinks become eligible for the SDIL even further could potentially lead to greater overall reductions in sugar concentrations and sugar purchased in soft drinks, as could extension of the SDIL to milk based drinks and other currently exempt categories that sometimes contain high levels of sugar.

Nevertheless, the overall reduction in sugar with an increase in volume we report here might represent a valuable benefit for public health with potential associated benefit to the food industry. The SDIL has also been found to have had no long term negative effects on the

For peer review only - http://bmjopen.bmj.com/site/about/guidelines.xhtml
For peer review only

share value or turnover of UK soft drinks companies,[35 36] suggesting that, contrary to industry predictions, public health can gain without negatively affecting the soft drinks sector.

We note a marked pre-implementation decline in purchasing of high levy tier drinks. It is possible that this was, at least in part, driven by concern from industry about a possible SSB tax, leading to some pre-announcement reformulation; alongside growing consumer awareness of, and concerns about, the health impacts of SSBs.[37] Although it is uncertain if this trend would have continued in the absence of the SDIL, it is likely to be beneficial for health.

Reassuringly, we did not observe any increase in purchasing of potentially harmful substitutes (ie, alcohol and confectionery) associated with the SDIL, which could have partially or wholly offset any public health gains from the SDIL. However, we did not study the SDIL's effect on purchases of other food groups or on overall diet.

In contrast with previous findings from Mexico and Barbados,[6 38] we did not observe an increase in purchased bottled water associated with the SDIL. Indeed purchases of bottled water decreased significantly during the study period (by 130.5 mL per household per week, or 15.7%). Although we cannot rule out an effect of the SDIL on bottled water purchases, we cannot think of a plausible pathway through which it achieved reductions in purchased bottled water. Instead, this reduction might be due to coincident increases in concern about single use plastic that have been attributed, in the UK, to the broadcast of the nature documentary series *Blue Planet 2* in October-December 2017.[39] It is not clear if a similar "Blue Planet effect" has occurred in other countries. Unlike for many other soft drinks, a like-for-like substitution is available for bottled water in countries such as the UK—that is, filling reusable water bottles with tap water. Several UK retailers have reported substantial growth in sales of reusable water bottles since 2018.[40] Given that tap water is freely available, it is difficult to study changes in its consumption directly.

Unanswered questions and future research

Future work should seek to understand the longer term effects of the SDIL on purchasing and consumption of soft drinks as well as total diet, and health outcomes. Differential effects of the SDIL on all these outcomes across population groups (eg, by socioeconomic position and in households with vs without children) should also be explored to determine whether the SDIL contributes to narrowing inequalities in health. The changes in purchasing we report here could be used as an input to health impact modelling to estimate the effect of changes on population prevalence of obesity, diabetes, and other chronic conditions. It is likely that the

For peer review only

For peer review only - http://bmjopen.bmj.com/site/about/guidelines.xhtml

reformulation that has occurred in response to the SDIL[16] reflects substantial increases in the use of artificial sweeteners in the UK soft drinks market. Given public mistrust of artificial sweeteners,[37] the effect of the SDIL on consumption of these should also be explored.

Conclusion

One year after implementation of the SDIL, purchased sugar in soft drinks decreased by around 8 g per household per week (or 2.7%) with an increase in the volume of purchased soft drinks of 189 mL per household per week (or 2.6%). This tiered tax aiming to stimulate industry to remove sugar from soft drinks might represent a benefit for public health (by reducing sugar purchased from soft drinks without substitution to confectionery and alcohol) and also to the soft drinks industry (by total volume of soft drinks purchased).

For peer review only

For peer review only - http://bmjopen.bmj.com/site/about/guidelines.xhtml

Figure legends

Fig 1 Schematic of overall analysis strategy. Solid lines=observed data; dashed lines=counterfactual estimated from previous observed data

Fig 2 Observed and modelled volume (mls) of drinks liable to the Soft Drinks Industry Levy (SDIL), and weight of confectionery (g) purchased per household per week, March 2014 to March 2019 (weighted). Points are observed data for drinks/ confectionery; black lines (with shadows) show modelled data (and 95% confidence intervals); red lines indicate the counterfactuals had the announcement (red solid line) and implementation (red dashed line) not happened; the first dashed vertical line indicates the announcement of SDIL; the second dashed vertical line indicates the implementation of SDIL; the Y axis varies in scale between panels to maximise the resolution of figures; modelled purchases include averaged effects for seasonality and the impact of December and January (Christmas period), and, for confectionery, Easter; the control category of toiletries is shown in Fig 3

Fig 3 Observed and modelled amount of sugar (g) in drinks liable to the Soft Drinks Industry Levy and confectionery purchased per household per week, March 2014 to March 2019 (weighted). Points are observed data for drinks/ confectionery and toiletries; black lines (with shadows) show modelled data (and 95% confidence intervals); red lines indicate the counterfactuals had the announcement (red solid line) and implementation (red dashed line) not happened; the first dashed vertical line indicates the announcement of SDIL; the second dashed vertical line indicates the implementation of SDIL; the Y axis varies in scale between panels to maximise the resolution of figures; modelled purchases include averaged effects for seasonality and the impact of December and January (Christmas period), and, for confectionery, Easter

For peer review only

For peer review only - http://bmjopen.bmj.com/site/about/guidelines.xhtml

Contributors

MW, SC, MR, RS, HR, JA, PS, OM, AB, and TLP conceived the study and defined the analytical strategy. NR, JA, OM, TLP, and SS performed statistical analyses and provided preliminary interpretation of findings. NR, JA, OM, and TLP drafted the manuscript. All authors critically interpreted the results, revised the manuscript, provided relevant intellectual input, and read and approved the final manuscript. NR and JA had primary responsibility for the final content. JA will act as guarantor. The corresponding author attests that all listed authors meet authorship criteria and that no others meeting the criteria have been omitted.

Funding

NR, OM, MW, and JA were funded by the Centre for Diet and Activity Research (CEDAR), a UK Clinical Research Collaboration (UKCRC) Public Health Research Centre of Excellence Funding from the British Heart Foundation, Cancer Research UK, Economic and Social Research Council, Medical Research Council, National Institute for Health Research (NIHR), and Wellcome Trust, under the auspices of the UKCRC. This project was funded by the NIHR Public Health Research programme (grant Nos 16/49/01 and 16/130/01). The work was also supported by the Medical Research Council (grant Nos MC_UU_12015/6 and MC_UU_00006/7). The views expressed are those of the authors and not necessarily those of the National Health Service, the NIHR, or the Department of Health and Social Care, UK. The funders had no role in study design, data collection and analysis, decision to publish, or preparation of the manuscript. DP had full access to all the data in the study and had final responsibility for the decision to submit for publication.

Competing interests

All authors have completed the ICMJE uniform disclosure form at www.icmje.org/coi_disclosure.pdf (available on request from the corresponding author) and declare: MW was director of the National Institute for Health Research Public Health Research Funding programme when this work was conducted, and OM was on secondment at the UK Department of Health and Social Care when this work was conducted and previously worked with Public Health England; no support from any organisation for the submitted work other than that described above; no financial relationships with any organisations that might have an interest in the submitted work in the previous three years; and no other relationships or activities that could appear to have influenced the submitted work.

Ethical approval

Not required for secondary data analysis of anonymised data.

Data sharing

The statistical code for the analyses are available from https://github.com/MRC-Epid/SDILEvaluation. Kantar Worldpanel data are not publicly available but can be purchased from Kantar Worldpanel (http://www.kantarworldpanel.com). The authors are not legally permitted to share the data used for this study but interested parties can contact Kantar WorldPanel representative Sean Cannon (Sean.Cannon@kantar.com) to inquire about accessing this proprietary data.

The lead author affirms that the manuscript is an honest, accurate, and transparent account of the study being reported; that no important aspects of the study have been omitted; and that any discrepancies from the study as planned (and, if relevant, registered) have been explained.

For peer review only - http://bmjopen.bmj.com/site/about/guidelines.xhtml

Dissemination to participants and related patient and public communities

This work was presented at the 2020 annual scientific meeting of the Society of Social Medicine. We will issue a press release on this work and engage with media outlets as relevant. We will summarise our findings in a Twitter thread. A lay summary of this paper will be prepared in advance of publication and shared on the Medical Research Council Epidemiology Unit and Centre for Diet and Activity Research websites. We will share this summary with our networks of public health practitioners and policymakers through our social media accounts and regular e-newsletter. A lay summary of the findings of the wider project of which this is part will be made available on the National Institute for Health Research website.

For peer review only

For peer review only - http://bmjopen.bmj.com/site/about/guidelines.xhtml

References

<jrn>1 Moynihan PJ, Kelly SAM. Effect on caries of restricting sugars intake: systematic review to inform WHO guidelines. *J Dent Res* 2014;93:8-18. PubMed doi:10.1177/0022034513508954</jrn>

<jrn>2 Imamura F, O'Connor L, Ye Z, et al. Consumption of sugar sweetened beverages, artificially sweetened beverages, and fruit juice and incidence of type 2 diabetes: systematic review, meta-analysis, and estimation of population attributable fraction. *BMJ* 2015;351:h3576. PubMed doi:10.1136/bmj.h3576</jrn>

<jrn>3 Xi B, Huang Y, Reilly KH, et al. Sugar-sweetened beverages and risk of hypertension and CVD: a dose-response meta-analysis. *Br J Nutr* 2015;113:709-17. PubMed doi:10.1017/S0007114514004383</jrn>

<bok>4 World Health Organization. *Tackling NCDs: "Best Buys" and Other Recommended Interventions for the Prevention and Control of Noncommunicable Diseases.* WHO, 2017.</bok>

<jrn>5 Teng AM, Jones AC, Mizdrak A, Signal L, Genç M, Wilson N. Impact of sugar-sweetened beverage taxes on purchases and dietary intake: Systematic review and meta-analysis. *Obes Rev* 2019;20:1187-204. PubMed doi:10.1111/obr.12868</jrn>

<jrn>6 Alvarado M, Unwin N, Sharp SJ, et al. Assessing the impact of the Barbados sugar-sweetened beverage tax on beverage sales: an observational study. *Int J Behav Nutr Phys Act* 2019;16:13. PubMed doi:10.1186/s12966-019-0776-7</jrn>

<jrn>7 Roberto CA, Lawman HG, LeVasseur MT, et al. Association of a Beverage Tax on Sugar-Sweetened and Artificially Sweetened Beverages With Changes in Beverage Prices and Sales at Chain Retailers in a Large Urban Setting. *JAMA* 2019;321:1799-810 doi:10.1001/jama.2019.4249.</jrn>

<jrn>8 Royo-Bordonada MA, Fernández-Escobar C, Simón L, Sanz-Barbero B, Padilla J. Impact of an excise tax on the consumption of sugar-sweetened beverages in young people living in poorer neighbourhoods of Catalonia, Spain: a difference in differences study. *BMC Public Health* 2019;19:1553. PubMed doi:10.1186/s12889-019-7908-5</jrn>

<jrn>9 Cawley J, Frisvold D, Hill A, Jones D. The impact of the Philadelphia beverage tax on purchases and consumption by adults and children. *J Health Econ* 2019;67:102225. PubMed doi:10.1016/j.jhealeco.2019.102225</jrn>

<jrn>10 Lee MM, Falbe J, Schillinger D, Basu S, McCulloch CE, Madsen KA. Sugar-Sweetened Beverage Consumption 3 Years After the Berkeley, California, Sugar-Sweetened Beverage Tax. *Am J Public Health* 2019;109:637-9. PubMed doi:10.2105/AJPH.2019.304971</jrn>

<jrn>11 Mytton OT, Eyles H, Ogilvie D. Evaluating the Health Impacts of Food and Beverage Taxes. *Curr Obes Rep* 2014;3:432-9. PubMed doi:10.1007/s13679-014-0123-x</jrn>

<jrn>12 Falbe J, Rojas N, Grummon AH, Madsen KA. Higher Retail Prices of Sugar-Sweetened Beverages 3 Months After Implementation of an Excise Tax in Berkeley, California. *Am J Public Health* 2015;105:2194-201. PubMed doi:10.2105/AJPH.2015.302881</jrn>

<jrn>13 Alvarado M, Kostova D, Suhrcke M, et al; Barbados SSB Tax Evaluation Group. Trends in beverage prices following the introduction of a tax on sugar-sweetened beverages in Barbados. *Prev Med* 2017;105S:S23-5. PubMed doi:10.1016/j.ypmed.2017.07.013</jrn>

For peer review only - http://bmjopen.bmj.com/site/about/guidelines.xhtml

<jrn>14 Cawley J, Willage B, Frisvold D. Pass-through of a tax on sugar-sweetened beverages at the philadelphia international airport. *JAMA* 2018;319:305-6. PubMed doi:10.1001/jama.2017.16903</jrn>

<jrn>15 Stacey N, Mudara C, Ng SW, van Walbeek C, Hofman K, Edoka I. Sugar-based beverage taxes and beverage prices: Evidence from South Africa's Health Promotion Levy. *Soc Sci Med* 2019;238:112465. PubMed doi:10.1016/j.socscimed.2019.112465</jrn>

<jrn>16 Scarborough P, Adhikari V, Harrington RA, et al. Impact of the announcement and implementation of the UK Soft Drinks Industry Levy on sugar content, price, product size and number of available soft drinks in the UK, 2015-19: A controlled interrupted time series analysis. *PLoS Med* 2020;17:e1003025. PubMed doi:10.1371/journal.pmed.1003025</jrn>

<bok>17 Penney T, Adams J, Briggs A, et al. *Theorising how the UK Soft Drinks Industry Levy could impact population level diet and health: Development of a multi-sectoral systems map.* International Society for Behavioural Nutrition and Physical Activity, 2017.</bok>

<jrn>18 Quirmbach D, Cornelsen L, Jebb SA, Marteau T, Smith R. Effect of increasing the price of sugar-sweetened beverages on alcoholic beverage purchases: an economic analysis of sales data. *J Epidemiol Community Health* 2018;72:324-30. PubMed doi:10.1136/jech-2017-209791</jrn>

<jrn>19 Smith RD, Cornelsen L, Quirmbach D, Jebb SA, Marteau TM. Are sweet snacks more sensitive to price increases than sugar-sweetened beverages: analysis of British food purchase data. *BMJ Open* 2018;8:e019788. PubMed doi:10.1136/bmjopen-2017-019788</jrn>

<jrn>20 Scheelbeek PFD, Cornelsen L, Marteau TM, Jebb SA, Smith RD. Potential impact on prevalence of obesity in the UK of a 20% price increase in high sugar snacks: modelling study. *BMJ* 2019;366:l4786. PubMed doi:10.1136/bmj.l4786</jrn>

<eref>21 HM Revenue & Customs. Soft Drinks Industry Levy 2016. https://www.gov.uk/government/publications/soft-drinks-industry-levy/soft-drinks-industry-levy.</eref>

<bok>22 Gauke D. *Budget 2016.* HM Treasury, 2016.</bok>

<eref>23 HM Revenue & Customs. Check if your drink is liable for the Soft Drinks Industry Levy. Secondary Check if your drink is liable for the Soft Drinks Industry Levy 2018. https://www.gov.uk/guidance/check-if-your-drink-is-liable-for-the-soft-drinks-industry-levy.</eref>

<bok>24 Public Health England. *Sugar reduction: Report on progress between 2015 and 2018.* Public Health England, 2019.</bok>

<jrn>25 Bandy LK, Scarborough P, Harrington RA, Rayner M, Jebb SA. Reductions in sugar sales from soft drinks in the UK from 2015 to 2018. *BMC Med* 2020;18:20. PubMed doi:10.1186/s12916-019-1477-4</jrn>

<bok>26 British Soft Drinks Association. *2018 Annual Report.* British Soft Drinks Association, 2019.</bok>

<jrn>27 Pell D, Penney TL, Mytton O, et al. Anticipatory changes in British household purchases of soft drinks associated with the announcement of the Soft Drinks Industry Levy: A controlled interrupted time series analysis. *PLoS Med* 2020;17:e1003269. PubMed doi:10.1371/journal.pmed.1003269</jrn>

<bok>28 White M, Cummins S, Rayner M, et al. *Evaluation of the health impacts of the UK Treasury Soft Drinks Industry Levy (SDIL).* NIHR Public Health Research Programme, 2017.</bok>

For peer review only - http://bmjopen.bmj.com/site/about/guidelines.xhtml

<bok>29 Webber D, O'Neill J. Average household income, UK: financial year ending 2019; Estimates of median and mean disposable income for people in the UK for the financial year ending 2019. 2020.</bok>

<jrn>30 Lopez Bernal J, Cummins S, Gasparrini A. The use of controls in interrupted time series studies of public health interventions. *Int J Epidemiol* 2018;47:2082-93. PubMed doi:10.1093/ije/dyy135</jrn>

<jrn>31 Oehlert GW. A note on the Delta Method. *Am Stat* 1992;46:27-9. </jrn>

<unknown>32. Knipe E. Families and households in the UK: 2017; Trends in living arrangements including families (with and without dependent children), people living alone and people in shared accommodation, broken down by size and type of household. 2017.</unknown> **[Author: Please provide additional details for this reference. A url would help]**

<bok>33 Smith K, Griffith R, O'Connell M. *Measuring the quality of people's diets: a comparison of intake and purchase data. Econometrics and IO of food and nutrition.* Toulouse School of Economics, 2012.</bok>

<jrn>34 Briggs ADM, Mytton OT, Kehlbacher A, et al. Health impact assessment of the UK soft drinks industry levy: a comparative risk assessment modelling study. *Lancet Public Health* 2016;2:e15-22. PubMed doi:10.1016/S2468-2667(16)30037-8</jrn>

<jrn>35 Law C, Cornelsen L, Adams J, et al. The impact of UK soft drinks industry levy on manufacturers' domestic turnover. *Econ Hum Biol* 2020;37:100866. PubMed doi:10.1016/j.ehb.2020.100866</jrn>

<jrn>36 Law C, Cornelsen L, Adams J, et al. An analysis of the stock market reaction to the announcements of the UK Soft Drinks Industry Levy. *Econ Hum Biol* 2020;38:100834. PubMed doi:10.1016/j.ehb.2019.100834</jrn>

<jrn>37 Pell D, Penney T, Hammond D, Vanderlee L, White M, Adams J. Support for, and perceived effectiveness of, the UK soft drinks industry levy among UK adults: cross-sectional analysis of the International Food Policy Study. *BMJ Open* 2019;9:e026698. PubMed doi:10.1136/bmjopen-2018-026698</jrn>

<jrn>38 Colchero MA, Popkin BM, Rivera JA, Ng SW. Beverage purchases from stores in Mexico under the excise tax on sugar sweetened beverages: observational study. *BMJ* 2016;352:h6704. PubMed doi:10.1136/bmj.h6704</jrn>

<bok>39 Males J, Van Aelst P. Did the Blue Planet set the Agenda for Plastic Pollution? An Explorative Study on the Influence of a Documentary on the Public, Media and Political Agendas. Environmental Communication, 2020:1-15.</bok>

<eref>40 O'Mahony A. The rise and fall: bottled water category report 2020. Secondary The rise and fall: bottled water category report 2020 2020. https://www.thegrocer.co.uk/category-reports/the-rise-and-fall-bottled-water-category-report-2020/603079.article</eref>

For peer review only - http://bmjopen.bmj.com/site/about/guidelines.xhtml

Table 1 Mean volume of, and amount of sugar in, purchased drinks and confectionery per household per week in relation to the UK soft drinks industry levy, March 2014 to March 2019 (weighted)

	Mean (SD) volume (mL) per household/week			Mean (SD) amount of sugar (g) per household/week		
	**Pre-announcement: Mar 2014-Mar 2016**	**Post-announcement: Mar 2016-Mar 2018**	**Post-implementation: Apr 2018-Mar 2019**	**Pre-announcement: Mar 2014-Mar 2016**	**Post-announcement: Mar 2016-Mar 2018**	**Post-implementation: Apr 2018-Mar 2019**
**All drinks**	7595 (295)	7547 (466)	7826 (540)	364 (17)	337(24)	307(19)
**Levy liable drinks (sugar/100 mL)**						
High tier (≥8 g)	880 (128)	680 (136)	363 (76)	98(14)	76(15)	40(9)
Low tier (≥5 g to <8 g	155 (32)	147 (37)	75 (32)	10(2)	10(2)	5(2)
No levy (<5 g):	1811 (169)	1876 (216)	2448 (321)	12(2)	12(3)	25(5)
>0 g to <5 g	785 (78)	989 (139)	768 (92)	12(2)	12(3)	25(5)
0 g	1027 (104)	1108 (132)	1459 (190)	0 (0)	0 (0)	0 (0)
Bottled water	591(72)	714(90)	786 (138)	0 (0)	0 (0)	0 (0)
**Levy exempt drinks**						
Alcoholic drinks	1874 (380)	1872 (456)	1948 (467)	.	.	.
Milk and milk based drinks:	3546 (137)	3540 (155)	3542 (148)	172 (7)	172(8)	170(7)
Fruit juices with no added sugar	516.6(29)	502(44)	520(47)	51(3)	49(4)	50(5)
Drinks sold as powders (g)	95(12)	88(11)	90(11)	21(3)	19(3)	18(3)
Confectionery (g)	308 (91)	303 (92)	318 (100)	173 (51)	170 (52)	178 (57)
Toiletries	123 (8)	120 (8)	121 (9)	.	.	.

Sugar from alcoholic drinks is not included here as many alcoholic drinks contain sugar but the product label does not provide the amount.

For peer review only - http://bmjopen.bmj.com/site/about/guidelines.xhtml

Table 2 Absolute and relative change in volume of drinks (mL) and confectionery (g) purchased per household per week in relation to the UK soft drinks industry Levy, March 2014 to March 2019 (weighted)

	**Analysis 1: pre-post announcement (Mar 2014-Mar 2018)**		**Analysis 2: pre-post implementation (Mar 2016-Mar 2019)**		**Analysis 3: pre-post announcement and implementation (Mar 2014-Mar 2019)**	
	**Absolute change (mL or g)**	**Relative change (%)**	**Absolute change (mL or g)**	**Relative change (%)**	**Absolute change (mL or g)**	**Relative change (%)**
**All drinks**	**11.8 (-103.7, 127.3)**	0.16 (-1.42, 1.74)	**187.8 (29.7, 345.9)**	**2.56 (0.40, 4.71)**	**188.8 (30.7, 346.9)**	**2.6 (0.4, 4.7)**
**Levy liable drinks (sugar/100 mL)**						
High tier (≥8 g)	**34.7 (8.06, 61.4)**	**7.27 (1.69, 12.9)**	**-171.6 (-208.1,-135.1)**	**-42.5 (-51.6, -33.5)**	**-140.8 (-177.3, -104.3)**	**-37.8 (-47.6, -28.0)**
Low tier (≥5 g to <8 g)*	**-65.7 (-77.5, -53.8)**	**-37.1 (-43.7, -30.4)**	**-71.8 (-87.8, -55.8)**	**-71.8 (-87.8, -55.8)**	**-170.5 (-186.5, -154.5)**	**-85.8 (-93.9, -77.8)**
No levy (<5 g):	**181.0 (118.4, 243.5)**	**11.1 (7.26, 14.9)**	**395.0 (309.4. 480.7)**	**19.8 (15.5, 24.1)**	**685.5 (599.8, 771.1)**	**40.2 (35.2, 45.2)**
>0 g to <5 g	**103.8 (75.2, 132.5)**	**16.7 (12.1, 21.3)**	**202.0 (162.7, 241.2)**	**25.0 (20.1, 29.9)**	**374.6 (335.4, 413.9)**	**59.0 (52.8, 65.1)**
0 g	**87.8 (41.1, 134.5)**	**8.66 (4.05, 13.3)**	**178.9 (115.6, 242.3)**	**14.7 (9.52, 20.0)**	**316.1 (252.7, 379.4)**	**29.4 (23.5, 35.3)**
Bottled water	30.3 (-62.0, 1.4)	4.24 (-8.7, 0.2)	**82.1 (-125.7, -38.4)**	**-10.5 (-16.1, -4.9)**	**-130.5 (-174.1, -88.8)**	**-15.7 (-20.9, -10.4)**
**Levy exempt drinks**						
Alcoholic drinks	-16.5 (-48.5, 15.4)	0.95 (-2.79, 0.89)	**-84.9 (-135.1, -34.7)**	**-4.81 (-7.66, -1.97)**	**-103.1 (-153.3, -53.0)**	**-5.8 (-8.60, -2.97)**
Milk and milk based drinks	**-185.5 (-249.7, -121.4)**	**-4.9 (-6.60, -3.20)**	**145.5 (64.4, 226.6)**	**4.21 (1.86, 6.56)**	**-132.8 (-213.9, -51.7)**	**-3.56 (-5.73, -1.38)**
No added sugar fruit juices	6.8 (-6.9, 20.5)	1.4 (-1.4, 4.3)	-6.2 (-24.8, 12.5)	-1.26 (-6.1, 2.5)	8.7 (-9.9, 27.3)	1.82 (-2.1, 5.7)
Drinks sold as powders (g)	**-6.9 (-10.0, -3.8)**	**-6.8 (-9.9, -3.8)**	**9.6 (5.3, 13.9)**	**11.2 (6.2, 16.2)**	0.9 (-3.3, 5.2)	1.0 (-3.5, 5.5)
Confectionery (g)	-10.1 (-53.9, 33.8)	-2.4 (-13.1, 8.2)	39.8 (-19.0, 98.6)	11.6 (-5.5, 28.8)	35.3 (94.1, -23.5)	10.2 (-6.8, 27.1)

Bold indicates significant difference at 95% confidence interval level

For peer review only

For peer review only - http://bmjopen.bmj.com/site/about/guidelines.xhtml

Table 3 Absolute and relative change in sugar in drinks and confectionery (g) purchased per household (95% CI) per week in relation to the UK SDIL, March 2014- March 2019 (weighted)

	**Analysis 1: pre-post announcement (Mar 2014-Mar 2018)**		**Analysis 2: pre-post implementation (Mar 2016-Mar 2019)**		**Analysis 3: pre-post announcement and implementation (Mar 2014-Mar 2019)**	
	**Absolute change (g)**	**Relative change (%)**	**Absolute change (g)**	**Relative change (%)**	**Absolute change (g)**	**Relative change (%)**
**All drinks**	**4.6 (0.5, 8.6)**	**1.4 (0.2, 2.7)**	**-12.9 (-18.5, -7.4)**	**-4.3 (-6.1, -2.4)**	**-8.0 (-13.6, -2.4)**	**-2.7 (-4.5, -0.8)**
**Levy liable drinks (sugar/100 mL)**						
High tier (≥8 g)	**5.5 (3.8, 7.2)**	**10.8 (7.4, 14.1)**	**-21.2 (-23.8, -18.5)**	**-49.3 (-55.4, -43.1)**	**-16.2 (-18.8, -13.5)**	**-42.6 (-49.6, -35.6)**
Low tier (≥5 g to <8 g)	**-4.3 (-6.1, -2.6)**	**-37.5 (-52.5, -22.5)**	**-5.0 (-7.4, -2.6)**	**-75.8 (-112.7, -38.9)**	**-11.5 (-13.9, -9.1)**	**-87.8 (-106.4, -69.2)**
No levy (<5 g)†	**5.7 (3.9, 7.4)**	**72.6 (50.3, 94.9)**	**9.7 (7.3,12.1)**	**56.0 (41.9, 70.0)**	**19.2 (16.7, 21.6)**	**242.8 (211.9, 273.7)**
>0 g to <5 g sugar per 100 mL†	**5.7 (3.9, 7.4)**	**72.6 (50.3, 94.9)**	**9.7 (7.3,12.1)**	**56.0 (41.9, 70.0)**	**19.2 (16.7, 21.6)**	**242.8 (211.9, 273.7)**
**Levy exempt drinks**						
Milk and milk based drinks	**-3.9 (-6.5, -1.3)**	**-2.2 (-3.6, -0.7)**	**4.1 (0.5, 7.7)**	**2.4 (0.3, 4.6)**	-3.1 (-6.7, 0.5)	-1.8 (-3.8, 0.3)
No added sugar fruit juices	**2.6 (0.3, 4.8)**	**5.7 (0.7, 10.7)**	-1.7 (-4.8, 1.5)	-3.5 (-10.0, 3.0)	2.6 (-0.5, 5.7)	5.9 (-1.2, 13.1)
Drinks sold as powders (g)	0.3 (-1.6, 2.2)	1.6 (-7.5, 10.6)	-0.04 (-2.7, 2.6)	-0.2 (-13.9, 13.5)	1.1 (-1.6, 3.7)	5.7 (-8.8, 20.2)
Confectionery (g)	-6.6 (-32.0, 18.9)	-2.8 (-13.8, 8.14)	22.1 (-12.0, 56.1)	11.4 (-6.2, 29.1)	18.4 (-15.7, 52.4)	9.3 (-8.0, 26.7)

The counterfactual for low tier drinks crossed 0 mL shortly before the end of the study period thus predicting negative purchases; therefore the non-counterfactual estimate at the end of the study period was compared with the final week during which the counterfactual was a positive number.
*Significant difference at 95% confidence interval level.

For peer review only - http://bmjopen.bmj.com/site/about/guidelines.xhtml

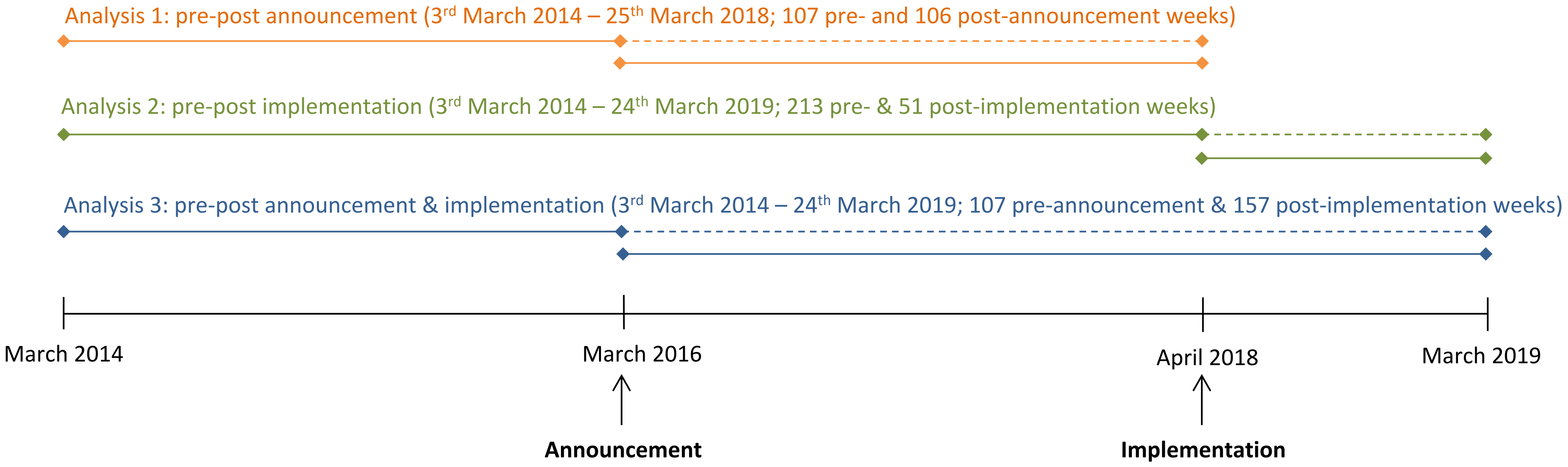

Figure 1: Schematic of overall analysis strategy

For peer review only - http://bmjopen.bmj.com/site/about/guidelines.xhtml

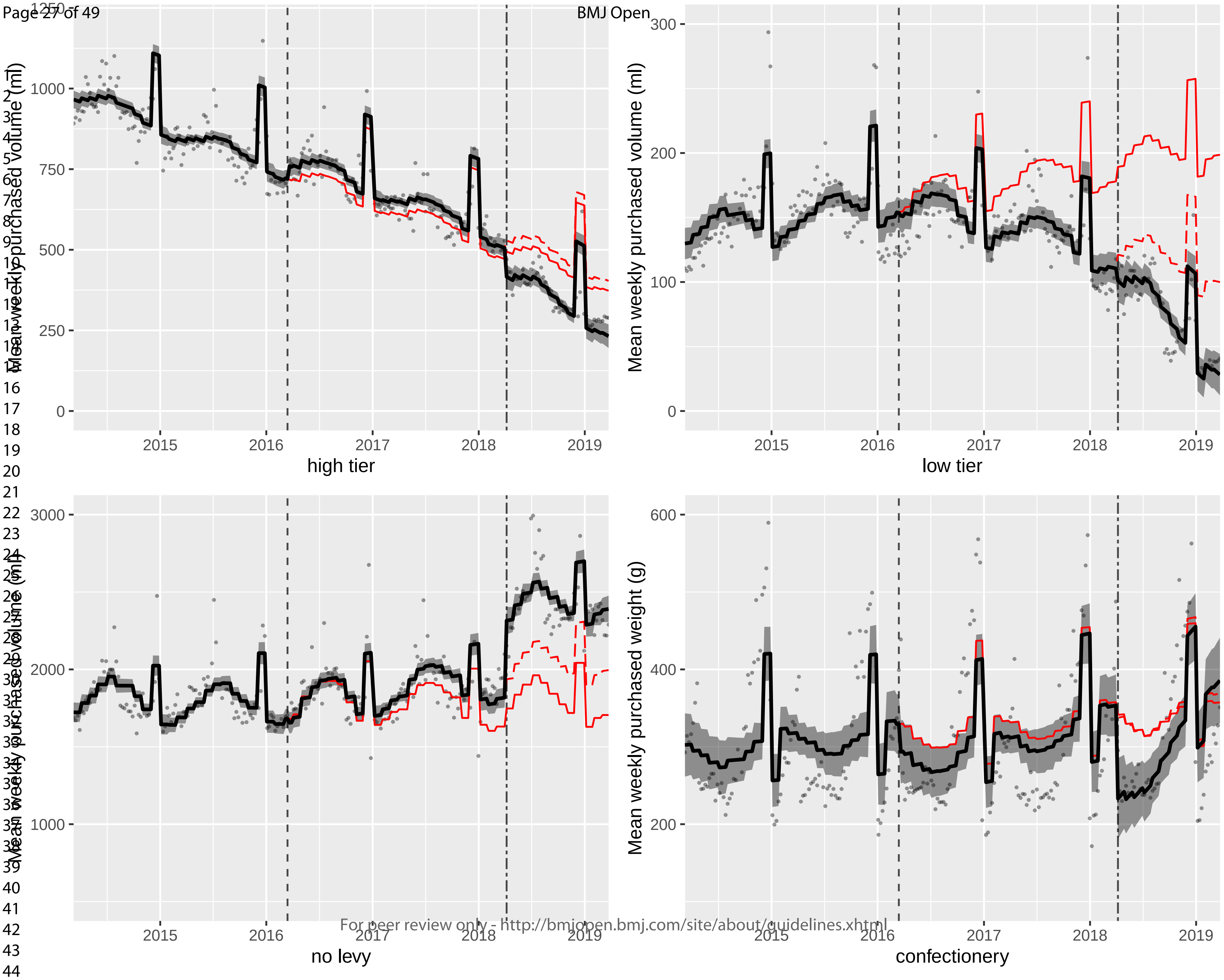
Mean weekly purchased volume (ml)
1250
1000
750
500
250
0
2015
2016
2017
2018
2019
high tier
Mean weekly purchased volume (ml)
300
200
100
0
low tier
Mean weekly purchased volume (ml)
3000
2000
1000
no levy
Mean weekly purchased weight (g)
600
400
200
confectionery

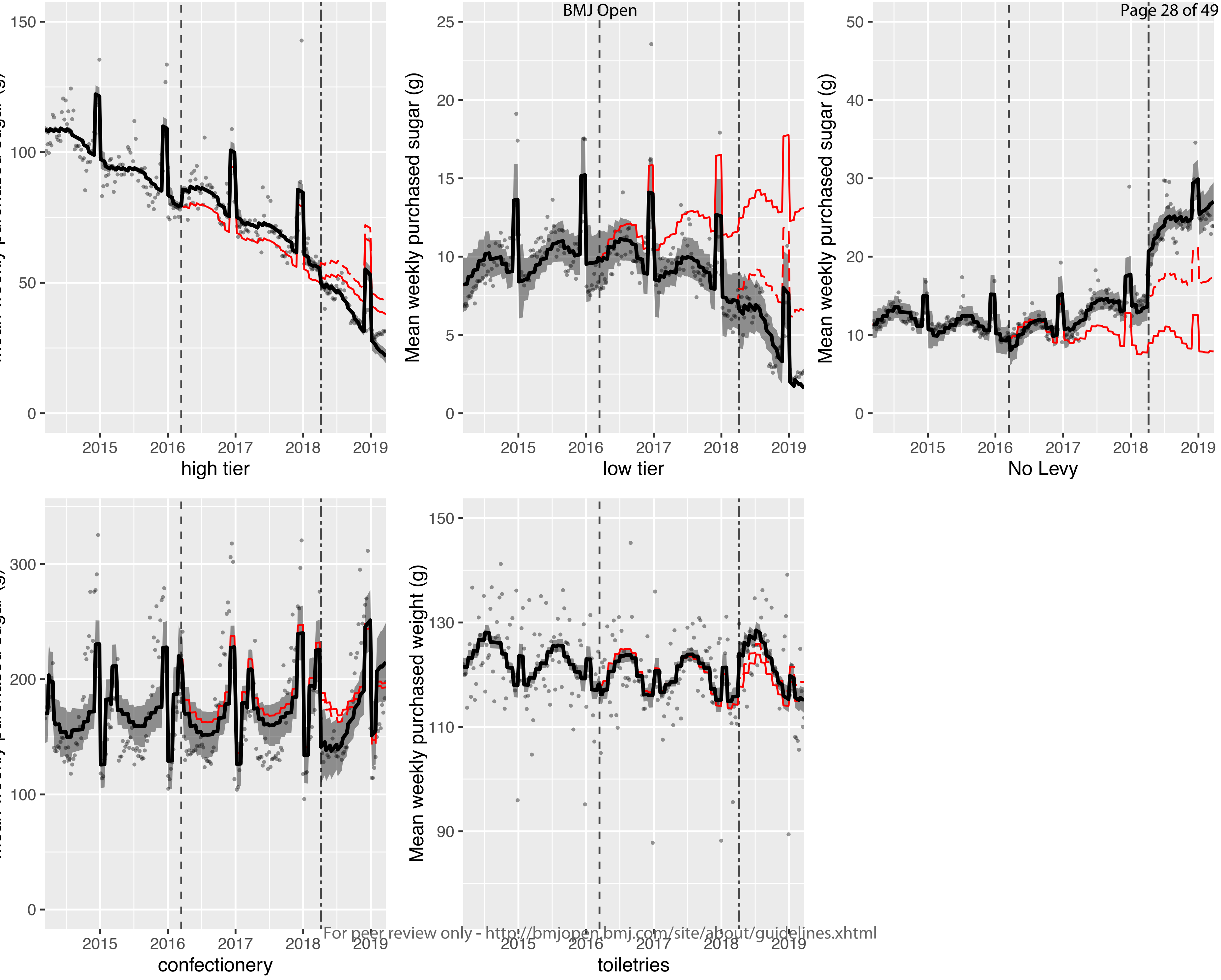
Mean weekly purchased sugar (g)
high tier
low tier
No Levy
confectionery
Mean weekly purchased weight (g)
toiletries
2015
2016
2017
2018
2019

Supplementary material A

STROBE Statement—checklist of items that should be included in reports of observational studies

	Item No	Recommendation	Section and Paragraph No
**Title and abstract**	1	(*a*) Indicate the study's design with a commonly used term in the title or the abstract	Title.
		(*b*) Provide in the abstract an informative and balanced summary of what was done and what was found	Abstract: main outcome measures; results; conclusions.
**Introduction**			
Background/rationale	2	Explain the scientific background and rationale for the investigation being reported	Introduction: paragraphs 1-3.
Objectives	3	State specific objectives, including any prespecified hypotheses	Introduction: paragraph 3-4.
**Methods**			
Study design	4	Present key elements of study design early in the paper	Methods: paragraph 1.
Setting	5	Describe the setting, locations, and relevant dates, including periods of recruitment, exposure, follow-up, and data collection	Methods: data source; overall analysis strategy; Figure 1.
Participants	6	(*a*) *Cohort study*—Give the eligibility criteria, and the sources and methods of selection of participants. Describe methods of follow-up *Case-control study*—Give the eligibility criteria, and the sources and methods of case ascertainment and control selection. Give the rationale for the choice of cases and controls *Cross-sectional study*—Give the eligibility criteria, and the sources and methods of selection of participants	Methods: data source, product categories – drinks, confectionery and toiletries; control group; outcome measures.
		(*b*) *Cohort study*—For matched studies, give matching criteria and number of exposed and unexposed *Case-control study*—For matched studies, give matching criteria and the number of controls per case	NA.
Variables	7	Clearly define all outcomes, exposures, predictors, potential confounders, and effect	Outcome measures; overall analysis strategy; primary analysis: category specific analyses; secondary analysis: all soft drinks categories combined, irrespective of levy

For peer review only

		modifiers. Give diagnostic criteria, if applicable	eligibility; sensitivity analysis: excluding small manufacturers; sensitivity analysis: ITS without a control category; supplementary material B.
Data sources/ measurement	8*	For each variable of interest, give sources of data and details of methods of assessment (measurement). Describe comparability of assessment methods if there is more than one group	Data source; overall analysis strategy; primary analysis: category specific analyses; secondary analysis: all soft drinks categories combined, irrespective of levy eligibility; sensitivity analysis: excluding small manufacturers; sensitivity analysis: ITS without a control category; supplementary material B.
Bias	9	Describe any efforts to address potential sources of bias	Product categories: drinks confectionery and toiletries; control group; sensitivity analysis: excluding small manufacturers; sensitivity analysis: ITS without a control category.
Study size	10	Explain how the study size was arrived at	Data source.
Quantitative variables	11	Explain how quantitative variables were handled in the analyses. If applicable, describe which groupings were chosen and why	Overall analysis strategy; primary analysis: category specific analyses; secondary analysis: all soft drinks categories combined, irrespective of levy eligibility; Box 1.
Statistical methods	12	(*a*) Describe all statistical methods, including those used to control for confounding	Control group; sensitivity analysis: ITS without a control category; Supplementary material A.
		(*b*) Describe any methods used to examine subgroups and interactions	Overall analysis strategy; primary analysis: category specific analyses; secondary analysis: all soft drinks categories combined, irrespective of levy eligibility.
		(*c*) Explain how missing data were addressed	Methods: data source
		(*d*) *Cohort study*—If applicable, explain how loss to follow-up was addressed<br>*Case-control study*—If applicable, explain how matching of cases and controls was addressed<br>*Cross-sectional study*—If applicable, describe analytical methods taking account of sampling strategy	Methods: data source.

Results

Participants	13*	(a) Report numbers of individuals at each stage of study—eg numbers potentially eligible, examined for eligibility, confirmed eligible, included in the study, completing follow-up, and analysed	Results: paragraph 1.
		(b) Give reasons for non-participation at each stage	Data source.
		(c) Consider use of a flow diagram	-
Descriptive data	14*	(a) Give characteristics of study participants (eg demographic, clinical,	Results: paragraph 1; Supplementary material C.

For peer review only

		social) and information on exposures and potential confounders	
		(b) Indicate number of participants with missing data for each variable of interest	Results: paragraph 1.
		(c) *Cohort study*—Summarise follow-up time (eg, average and total amount)	NA.
Outcome data	15*	*Cohort study*—Report numbers of outcome events or summary measures over time	NA.
		Case-control study—Report numbers in each exposure category, or summary measures of exposure	NA.
		Cross-sectional study—Report numbers of outcome events or summary measures	Results: paragraph 2; Table 1.
Main results	16	(*a*) Give unadjusted estimates and, if applicable, confounder-adjusted estimates and their precision (eg, 95% confidence interval). Make clear which confounders were adjusted for and why they were included	Primary analysis: category specific results; high tier drinks; low tier drinks; no levy drinks; levy exempt drinks and confectionery; Figures 2-3; Tables 2-3; supplementary material D.
		(*b*) Report category boundaries when continuous variables were categorized	Introduction - paragraph 3; product categories: drinks, confectionery and toiletries; Box 1.
		(*c*) If relevant, consider translating estimates of relative risk into absolute risk for a meaningful time period	Tables 2-3.
Other analyses	17	Report other analyses done—eg analyses of subgroups and interactions, and sensitivity analyses	Sensitivity analysis: excluding small manufacturers; sensitivity analysis: no control category; supplementary tables 4a-b; supplementary material G.
**Discussion**			
Key results	18	Summarise key results with reference to study objectives	Summary of main findings.
Limitations	19	Discuss limitations of the study, taking into account sources of potential bias or imprecision. Discuss both direction and magnitude of any potential bias	Key strengths and limitations.
Interpretation	20	Give a cautious overall interpretation of results considering objectives, limitations, multiplicity of analyses, results from similar studies, and other relevant evidence	Interpretation of findings - paragraphs 1-3.
Generalisability	21	Discuss the generalisability (external validity) of the study results	Interpretation of findings - paragraphs 4.
**Other information**			
Funding	22	Give the source of funding and the role of the funders for the present study and, if applicable, for the original study on which the present article is based	Funding.

*Give information separately for cases and controls in case-control studies and, if applicable, for exposed and unexposed groups in cohort and cross-sectional studies.

For peer review only

Note: An Explanation and Elaboration article discusses each checklist item and gives methodological background and published examples of transparent reporting. The STROBE checklist is best used in conjunction with this article (freely available on the Web sites of PLoS Medicine at http://www.plosmedicine.org/, Annals of Internal Medicine at http://www.annals.org/, and Epidemiology at http://www.epidem.com/). Information on the STROBE Initiative is available at www.strobe-statement.org.

For peer review only

Supplementary material B

The model specification is given by:

$$Y_t = \beta_0 + \beta_1 T + \beta_2 A_t + \beta_3 A_t\, T + \beta_4 A_t\, Z + \beta_5 A_t\, TZ + \beta_6 I_t + \beta_7 I_t\, T + \beta_8 I_t\, Z + \beta_9 I_t\, TZ + e_t$$

Y	*Average volume of (or purchased sugar in) drink or confectionery per household per week at week t (t=1,….,264)*
T	*Weeks since the start of the study; 1,…,264*
A_t	*0 if t prior to announcement, 1 if t on or after announcement*
I_t	*0 if t prior to implementation, 1 if t on or after implementation*
Z	*Control category (toiletries) = 0, drink or confectionery category = 1*
e_t	$N(0,\sigma^2)$ *representing the residual variance of the model*

Dummy indicator variables determined to be statistically significant ($p<0.05$) were included for the intervention group as appropriate representing: interaction terms restart at 0 at the point of the interventions; the increase in purchases seen throughout December in the weeks before Christmas; the fall in purchases in the weeks immediately after Christmas; and the increase in confectionery purchases seen at Easter, for toiletries these were set to 0. To adjust for temperature-related trends in drink consumption the average UK monthly temperature was included in the intervention group with the average study period temperature used for toiletries.[5] Quadratic functions of announcement trends were included where they improved model fit - assessed using likelihood ratio tests. Stationary was examined using augmented Dickey-Fuller tests.[41] Autocorrelation between preceding time points was examined using autocorrelation and partial-autocorrelation plots. An appropriate autocorrelation structure was determined and then compared to alternative models using likelihood ratio tests. Visual inspection of the data suggested no additional benefit would be gained from including polynomial terms.

For peer review only

Supplementary material C

Supplementary Table 1: Demographic characteristics of Kantar Worldpanel take-home panel households from March 2014 – March 2018 (weighted)

	Level	Kantar Worldpanel (%)	UK population (%)
Children in household[1]	No	65.3	71.5
	Yes	34.6	28.5
Social grade of chief income earner[2]	AB: Higher and intermediate managerial	19.3	27
	C1: Junior managerial	34.1	28
	C2: Skilled manual workers	16.4	20
	D: Semi and unskilled-manual workers	12.4	15
	E: lowest grade workers	7.9	10
Total household income (£ per annum)[3]	0-9,999	6.4	..
	10,000-19,999	18.9	..
	20,000-29,999	17.2	..
	30,000-39,999	13.1	....
	40,000-49,999	9.0	..
	50,000-59,999	5.5	..
	60,000-69,999	2.9	..
	70,000+	4.1	..
	Refused to answer	14.4	..
	Mean (£)	..	35,697
	Median (£)	..	28,947
Highest qualification of chief income earner[4]	Higher than School leaving qualifications taken at ~18 years (e.g. A-Levels)	38.3	43.8
	School leaving qualifications taken at ~18 years (e.g. A-Levels)	12.3	22.4
	School leaving qualifications taken at ~16 years (e.g. GCSE)	20.6	18.7
	Other (including no qualifications and unknown)	16.2	15.1

[1]Average of households with dependent children from 2014-2018; [2]UK population figures from 2016; [3]No directly comparable figures available from ONS, mean and medians are averaged over 2014-2019; [4]UK population figures from 2014

For peer review only

Supplementary material D

Supplementary Figure 1a. Observed and modelled volume (ml) of drinks exempt from the Soft Drinks Industry Levy purchased per household per week, March 2014- March 2019 (weighted)

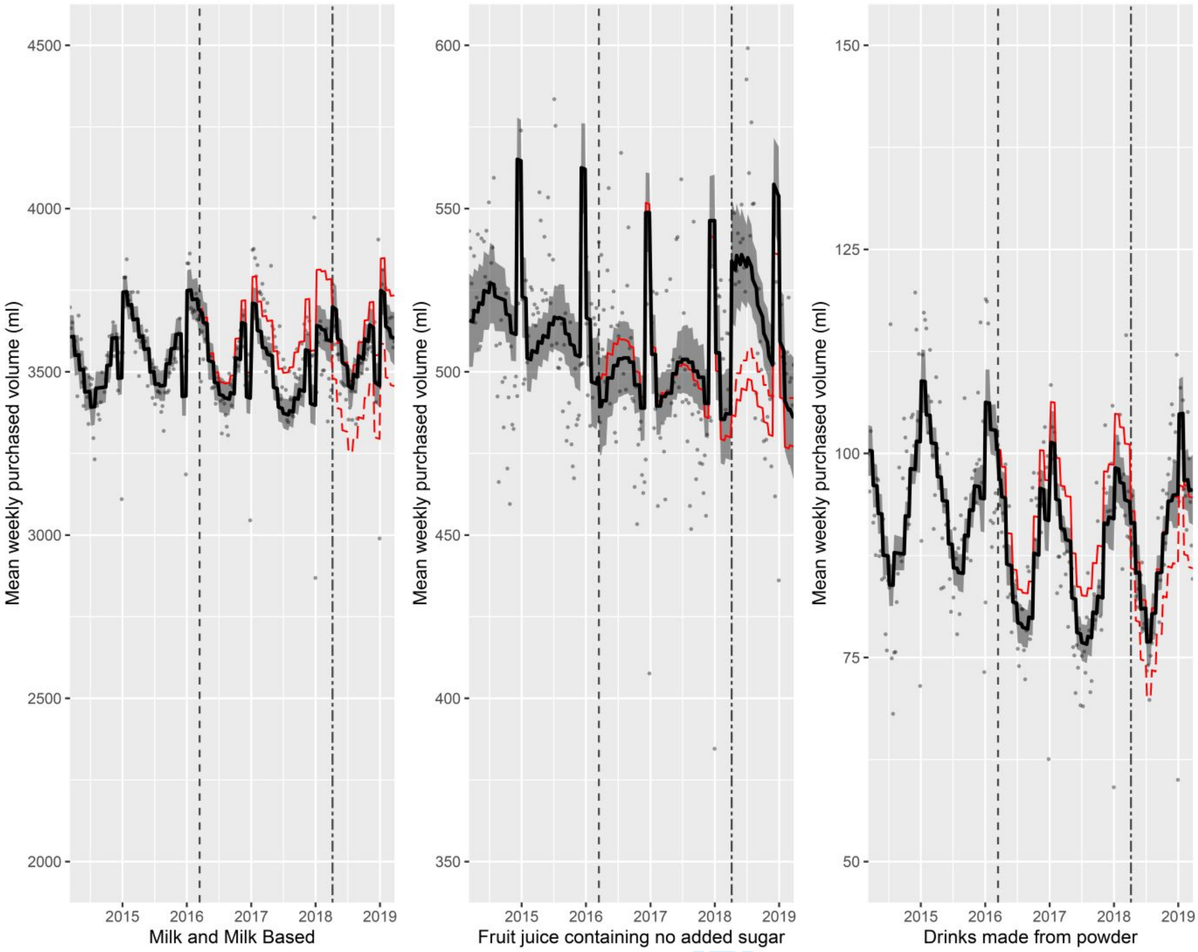

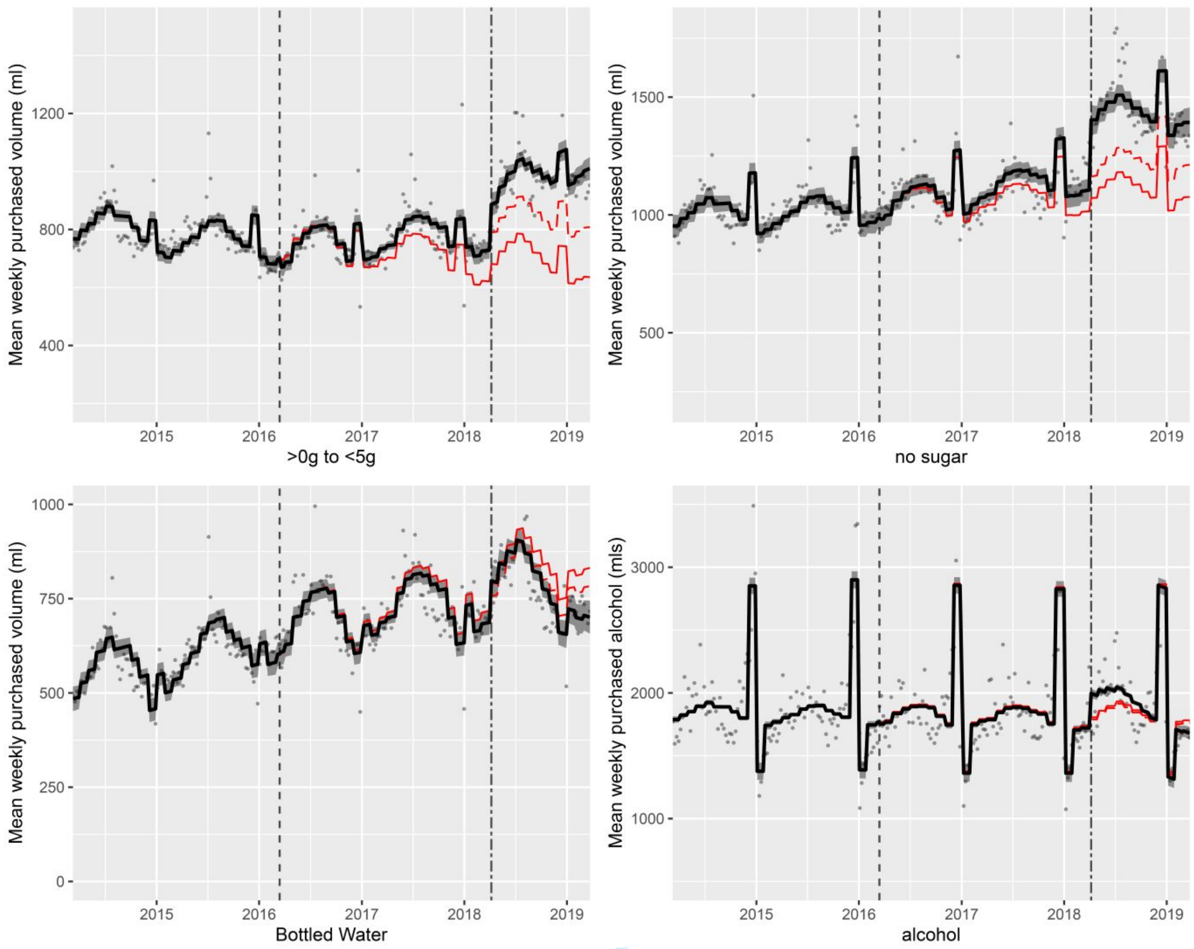

Notes. Points are observed data, black lines (with shadows) are modelled data (and 95% confidence intervals); red lines indicate counterfactuals had the announcement (red solid line) and implementation (red dashed line) not happened; the first dashed vertical line indicates the point of announcement; the second dashed vertical line indicates the point of implementation; The Y-axis varies in scale between panels to maximise the resolution of figures; modelled purchases include averaged effects for seasonality and the impact of December and January (Christmas period). The control category of toiletries is shown in Figure 3.

view only

Supplementary Figure 1b. Observed and modelled amount of sugar in drinks exempt from the Soft Drinks Industry Levy purchased per household per week, March 2014- March 2019 (weighted)

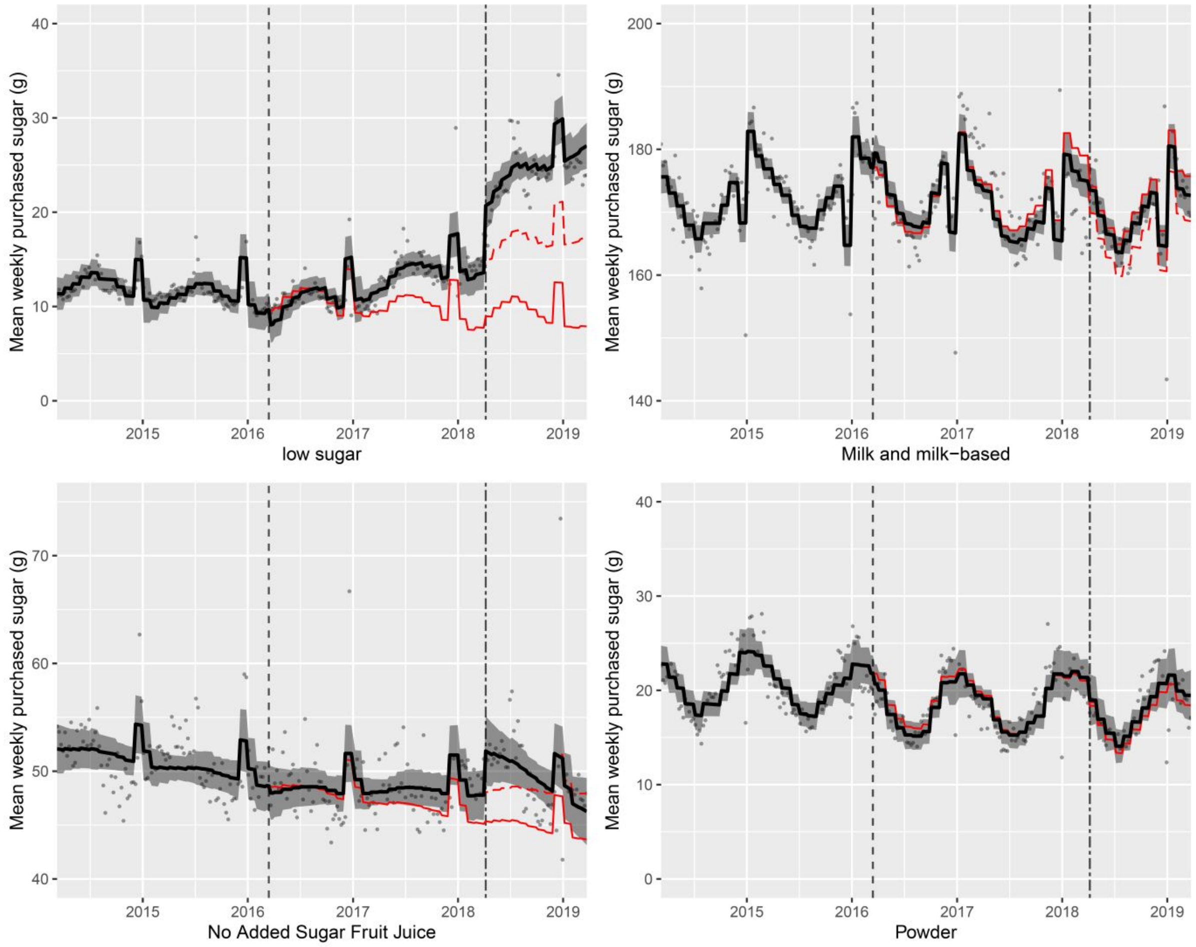

Notes. Points are observed data, black lines (with shadows) are modelled data (and 95% confidence intervals); red lines indicate counterfactuals had the announcement (red solid line) and implementation (red dashed line) not happened; the first dashed vertical line indicates the point of announcement; the second dashed vertical line indicates the point of implementation; The Y-axis varies in scale between panels to maximise the resolution of figures; modelled purchases include averaged effects for seasonality and the impact of December and January (Christmas period). The control category of toiletries is shown in Figure 3.

Supplementary Table 2a: Modelled level and trend changes in volume of drinks (ml) and confectionery (g) purchased per household per week (95% CI) in relation to the UK SDIL, March 2014 - March 2019 (weighted)

	Analysis 1: pre-post announcement (March 2014 – March 2018)		Analysis 2: pre-post implementation (March 2016 – March 2019)		Analysis 3: pre-post announcement & implementation (March 2014 – March 2019)	
	Level Change	Trend Change	Level Change	Trend Change	Level Change	Trend Change
Levy liable drinks						
High tier (≥8g sugar per 100ml)	44.0 (-7.7, 95.7)	-0.1 (-0.9, 0.7)	**-117.2 (-183.3, -51.1)**	1.0 (-0.9, 2.9)	-73.2 (-157.1, 10.7)	0.9 (-1.2, 3.0)
Low tier (≥5g - <8g sugar per 100ml)	-0.11 (-22.4, 22.1)	**-0.6 (-1.0, -0.3)**	-26.3 (-53.6, 1.04)	-0.81 (-1.6, 0.01)	-26.4 (-61.7, 8.8)	**-1.5 (-2.4, -0.6)**
No levy (<5g sugar per 100ml)	-28.2 (-149.4, 92.9)	**2.0 (0.04, 3.9)**	**372.0 (217.6, 526.4)**	**0.52 (-3.9, 4.9)**	**343.8 (147.5, 540.0)**	2.5 (-2.3, 7.3)
>0g to <5g sugar per 100ml	-27.1 (-82.6, 28.5)	**1.3 (0.4, 2.2)**	**88.1 (17.0, 159.3)**	**2.31 (0.3, 4.3)**	61.0 (-29.2, 151.4)	**3.6 (1.4, 5.8)**
0g sugar per 100ml	-7.37 (-93.9, 79.2)	0.9 (-0.6, 2.4)	**231.0 (125.2, 336.8)**	-0.99 (-4.3, 2.3)	**223.6 (87.0, 360.3)**	-0.08 (-3.7, 3.5)
Bottled water	6.86 (-53.4, 67.2)	-0.4 (-1.4, 0.6)	36.6 (-38.8, 112.0)	-2.24 (-4.5, 0.005)	43.5 (-53.1, 140.0)	**-2.6 (-5.1, -0.2)**
Levy exempt drinks						
Alcoholic drinks	-10.8 (-48.6, 27.0)	-0.07 (-0.5, 0.3)	**208.0 (137.9, 278.1)**	**-5.7 (-7.8, -3.6)**	**197.2 (117.6, 276.8)**	**-5.8 (-7.9, -3.7)**
Milk and milk based drinks[1]	-6.61 (-105.8, 92.6)	-1.7 (-4.0, 0.5)	**210.7 (98.7, 322.7)**	-1.2 (-5.3, 2.9)	**204.1 (54.4, 353.7)**	-2.9 (-7.6, 1.8)
No added sugar fruit juices	-8.72 (-34.3, 16.9)	0.1 (-0.3, 0.6)	30.5 (-0.9, 61.9)	-0.7 (-1.6, 0.3)	21.78 (-18.8, 62.3)	-0.5 (-1.6, 0.5)
Drinks sold as powders (g)	-1.79 (-7.8, 4.18)	-0.06 (-0.2, 0.03)	0.10 (-7.7, 7.9)	**0.3 (0.04, 0.5)**	-1.69 (-11.6, 8.17)	0.2 (-0.04, 0.4)
Confectionery (g)	-17.0 (-88.0, 54.0)	0.07 (-1.3, 1.5)	-77.9 (-163.9, 8.19)	2.4 (-0.6, 5.4)	**-98.9 (-206.4, -16.7)**	2.5 (-0.8, 5.8)

Notes. Bold indicates a significant difference at the 95% confidence interval level. [1]Milk comprises drinks in the following categories: semi-skimmed; specific low fat % milk (e.g. 1% fat milk); whole milk; buttermilk; modified milk; other milk; other non-cows milk; rice drink; soya milk. Skimmed milk is excluded from all analysis in this paper due to missing data.

For peer review only

Supplementary Table 2b: Modelled level and trend changes in sugar in drinks (ml) and confectionery (g) purchased per household per week (95% CI) in relation to the UK SDIL, March 2014 - March 2019 (weighted)

	Analysis 1: pre-post announcement (March 2014 – March 2018)		Analysis 2: pre-post implementation (March 2016 – March 2019)		Analysis 3: pre-post announcement & implementation (March 2014 – March 2019)	
	Level Change	Trend Change	Level Change	Trend Change	Level Change	Trend Change
Levy liable drinks						
High tier (≥8g sugar per 100ml)	**8.9 (6.8, 10.9)**	**-0.05 (-0.08, -0.03)**	**-11.5 (-15.3, -7.7)**	**-0.1 (-0.2, -0.02)**	-2.6 (-6.9, 1.6)	**-0.2 (-0.3, -0.07)**
Low tier (≥5g - <8g sugar per 100ml)	1.7 (-1.6, 5.1)	**-0.07 (-0.1, -0.02)**	**-6.2 (-10.6, -1.8)**	0.09 (-0.03, 0.2)	-4.5 (-10.0, 1.1)	0.02 (-0.1, 0.2)
No levy (<5g sugar per 100ml)	0.2 (-3.1, 3.5)	0.04 (-0.01, 0.09)	0.8 (-3.6, 5.3)	**0.2 (0.1, 0.4)**	1.0 (-4.5, 6.6)	**0.3 (0.2, 0.4)**
>0g to <5g sugar per 100ml	0.2 (-3.1, 3.5)	0.04 (-0.01, 0.09)	0.8 (-3.6, 5.3)	**0.2 (0.1, 0.4)**	1.0 (-4.5, 6.6)	**0.3 (0.2, 0.4)**
Levy exempt drinks						
Milk and milk based drinks[1]	4.1 (-0.1, 9.1)	**-0.09 (-0.2, -0.01)**	-2.0 (-8.6, 4.6)	**0.2 (0.01, 0.4)**	2.1 (-6.2, 10.4)	0.1 (-0.09, 0.3)
No added sugar fruit juices	1.0 (-3.4, 5.4)	0.001 (-0.06, 0.06)	-1.6 (-7.3, 4.1)	0.07 (-0.09, 0.2)	-0.6 (-7.8, 6.6)	0.07 (-0.1, 0.2)
Drinks sold as powders (g)	0.6 (-3.3, 4.5)	-0.02 (-0.08, 0.04)	-5.1 (-0.004, 10.2)	**0.2 (0.03, 0.3)**	-4.5 (-10.9, 1.9)	**0.2 (0.001, 0.3)**
Confectionery (g)	-8.6 (-49.7, 32.6)	0.03 (-0.8, 0.9)	-49.4 (-99.2, 0.4)	1.5 (-0.2, 3.2)	**-58.0 (-122.6, -6.7)**	1.6 (-0.4, 3.5)

Notes. Bold indicates a significant difference at the 95% confidence interval level. [1]Milk comprises drinks in the following categories: semi-skimmed; specific low fat % milk (e.g. 1% fat milk); whole milk; buttermilk; modified milk; other milk; other non-cows milk; rice drink; soya milk. Skimmed milk is excluded from all analysis in this paper due to missing data.

For peer review only

Supplementary material E

Secondary analysis: all drinks categories combined irrespective of levy eligibility

Supplementary Table 3a: Level and trend changes in volume of, and sugar in, all drinks (ml) and confectionery (g) purchased per household per week (95% CI) in relation to the UK SDIL, March 2014 - March 2019 (weighted)

	Analysis 1: pre-post announcement (March 2014 – March 2018)		Analysis 2: pre-post implementation (March 2016 – March 2019)		Analysis 3: pre-post announcement & implementation (March 2014 – March 2019)	
	Level Change	Trend Change	Level Change	Trend Change	Level Change	Trend Change
All drinks - volume						
Higher tier (≥8g sugar per 100ml)	22.8 (-37.5, 83.1)	0.5 (-0.5, 1.5)	-76.8 (-154.0 ,0.4)	-2.2 (-4.4, 0.01)	-54.0 (-152.0, 44.0)	-1.7 (-4.1, 0.7)
Lower tier (≥5g - <8g sugar per 100ml)	19.0 (-52.2, 90.2)	**-3.2 (-5.9, -0.5)**	27.9 (-47.8, 103.6)	-4.4 (-8.9, 0.02)	46.9 (-57.0, 150.8)	**-7.6 (-12.9, -2.4)**
Drinks containing <5g sugar per 100ml)	-152.1 (-348.3, 44.1)	2.6 (-0.7, 5.8)	**435.7 (187.7, 683.7)**	1.1 (-6.0, 8.3)	283.6 (-32.6, 599.8)	3.7 (-4.2, 11.5)
Levy liable drinks combined	15.2 (-72.9, 103.3)	0.6 (-0.8, 2.0)	-39.7 (-152.7, 73.4)	0.01 (-3.2, 3.2)	-24.5 (-167.8, 118.9)	0.6 (-2.9, 4.1)
All drinks excluding milk[1]	12.9 (-205.3, 231.0)	1.0 (-2.6, 4.5)	**347.6 (70.1, 625.0)**	-5.2 (-13.1, 2.7)	**360.5 (7.47, 713.4)**	-4.2(-12.9, 4.5)
All drinks combined	33.9 (-189.0, 256.7)	-0.2 (-3.8, 3.4)	**453.0 (170.1, 735.9)**	-5.1 (-13.2, 3.0)	**486.9 (126.7, 847.0)**	-5.4 (-14.2, 3.5)
All drinks – sugar						
Higher tier (≥8g sugar per 100ml)	4.1 (-2.6, 10.8)	0.04 (-0.07, 1.1)	**-13.4 (-22.2, -4.7)**	-0.1 (-0.4, 0.1)	-9.3 (-20.3, 1.7)	-0.08 (-0.3, 0.2)
Lower tier (≥5g - <8g sugar per 100ml)	**11.0 (5.6, 16.3)**	**-0.2 (-0.3, -0.1)**	-2.7 (-9.67, 4.3)	-0.01 (-0.2,0.2)	8.3 (-0.5, 17.1)	**-0.2 (-0.4, -0.01)**
Drinks containing <5g sugar per 100ml)	-6.3 (-12.2, -0.4)	**1.0 (0.9, 1.0)**	-0.7 (-8.30, 7.0)	**0.4 (0.2, 0.6)**	-7.0 (-16.6, 2.7)	**1.4 (1.1, 1.6)**
Levy liable drinks combined	5.0 (-1.8, 11.8)	-0.003 (-0.1, 0.1)	**-13.1 (-22.0, -4.2)**	0.02 (-0.2, 0.3)	-8.1(-19.3, 3.1)	0.02 (-0.3, 0.3)
All drinks excluding milk[1]	3.1 (-4.6, 10.8)	0.04 (-0.08, 0.2)	-9.3 (-19.2, 0.7)	-0.09 (-0.4, 0.2)	-6.2 (-18.8, 6.4)	-0.05 (-0.4, 0.3)
All drinks combined	5.3 (-2.6, 13.2)	-0.02 (-0.1, 0.1)	-5.7 (-15.9, 4.5)	-0.08 (-0.4, 0.2)	-0.4 (-13.2, 12.5)	-0.1 (-0.4, 0.2)

Notes. Bold indicates a significant difference at the 95% confidence interval level. The levy liable drinks category is a combination of high tier, low tier and no levy drinks. [1]Milk comprises drinks in the following categories: semi-skimmed; specific low fat % milk (e.g. 1% fat milk); whole milk; buttermilk; modified milk; other milk; other non-cows milk; rice drink; soya milk. Skimmed milk is excluded from all analysis in this paper due to missing data.

For peer review only

Supplementary Table 3b: Absolute and relative change in volume of, and sugar in, all drinks and confectionery (g) purchased per household (95% CI) per week in relation to the UK SDIL, March 2014- March 2019 (weighted)

	Analysis 1: pre-post announcement (March 2014 – March 2018)		Analysis 2: pre-post implementation (March 2016 – March 2019)		Analysis 3: pre-post announcement & implementation (March 2014 – March 2019)	
	Absolute change (ml or g)	Relative change (%)	Absolute change (ml or g)	Relative change (%)	Absolute change (ml or g)	Relative change (%)
All drinks - volume						
High tier (≥8g sugar per 100ml)	**76.6 (45.6, 107.7)**	**7.5 (4.5, 10.6)**	**-190.9 (-233.5, -148.3)**	**-19.4 (-23.7, -15.1)**	**-85.6 (-128.2, -43.0)**	**-9.8 (-14.6, -4.9)**
Low tier (≥5g - <8g sugar per 100ml)	316.7 (-241,7, -391.7)	**-28.0 (-21.3, -34.6)**	**-205.2 (-295.9, -114.5)**	**-26.2 (-37.8, -14.6)**	**-693.6 (-784.3, -602.9)**	**-54.5 (-61.7, -47.4)**
Drinks containing <5g sugar per 100ml)	**114.3 (12.4, 216.2)**	**2.2 (0.2, 4.1)**	**506.7 (367.3, 646.1)**	**9.0 (6.5, 11.4)**	**760.2 (620.8, 899.6)**	**14.1 (11.5, 16.6)**
Levy liable drinks combined	**76.5 (31.1, 121.8)**	**6.0 (2.5, 9.6)**	-42.7 (-104.9, 19.6)	-3.3(-8.1, 1.5)	**66.3 (4.1, 128.6)**	**5.6 (0.3, 10.8)**
All drinks excluding milk[1]	**116.1 (3.3, 229.0)**	**3.3 (0.09, 6.4)**	79.6 (-74.8, 234.1)	2.1 (-2.0, 6.1)	**250.1 (95.7, 404.5)**	**6.8 (2.6, 11.0)**
All drinks combined	11.8 (-103.7, 127.3)	0.2 (-1.4, 1.7)	**187.8 (29.7, 345.9)**	**2.6 (0.4, 4.7)**	**188.8 (30.7, 346.9)**	**2.6 (0.4, 4.7)**
All drinks – sugar						
High tier (≥8g sugar per 100ml)	**9.3 (5.9, 12.8)**	**7.6 (4.8, 10.4)**	**-22.9 (-27.8, -18.1)**	**-19.4 (-23.4, -15.3)**	**-9.99 (-14.8, -5.18)**	**-9.5 (-14.0, -4.9)**
Low tier (≥5g - <8g sugar per 100ml)	**-9.7 (-12.5, -6.9)**	**-18.6 (-23.9, -13.3)**	**-7.2 (-11.0, -3.4)**	**-19.4 (-29.7, -9.1)**	**-26.7 (-30.5, -22.9)**	**-47.1 (-53.9, -40.4)**
Drinks containing <5g sugar per 100ml)	**5.1 (2.1, 8.1)**	**3.6 (1.5, 5.8)**	**16.7 (12.5, 20.9)**	**11.2 (8.4, 14.0)**	**28.6 (24.4, 32.8)**	**20.9 (17.9, 23.9)**
Levy liable drinks combined	**6.1 (2.6, 9.6)**	**8.6 (3.6, 13.5)**	**-15.5 (-20.4, -10.7)**	**-22.7 (-29.8, -15.6)**	**-8.0 (-12.9, -3.2)**	**-13.2 (-21.1, -5.2)**
All drinks excluding milk[1]	**8.9 (5.0, 12.8)**	**6.5 (3.6, 9.4)**	**-17.2 (-22.7, -11.8)**	**-12.7 (-16.7, -8.67)**	**-4.5 (-10.0, 1.0)**	**-3.7 (-8.1, 0.8)**
All drinks combined	**4.6 (0.5, 8.6)**	**1.4 (0.2, 2.7)**	**-12.9 (-18.5, -7.4)**	**-4.3 (-6.1, -2.4)**	**-8.0 (-13.6, -2.4)**	**-2.7 (-4.5, -0.8)**

Notes. Bold indicates a significant difference at the 95% confidence interval level. The levy liable drinks category is a combination of high tier, low tier and no levy drinks. [1]Milk comprises drinks in the following categories: semi-skimmed; specific low fat % milk (e.g. 1% fat milk); whole milk; buttermilk; modified milk; other milk; other non-cows milk; rice drink; soya milk. Skimmed milk is excluded from all analysis in this paper due to missing data.

For peer review only

Supplementary material F

Sensitivity analysis: excluding small manufacturers

Supplementary table 4a: Modelled level and trend changes in volume of, and sugar in, drinks (ml) purchased per household per week (95% CI) in relation to the UK SDIL, March 2014 - March 2019; excluding small manufacturers (weighted)

	Analysis 1: pre-post announcement (March 2014 – March 2018)		Analysis 2: pre-post implementation (March 2016 – March 2019)		Analysis 3: pre-post announcement & implementation (March 2014 – March 2019)	
	Level Change	Trend Change	Level Change	Trend Change	Level Change	Trend Change
Volume						
High levy tier (≥8g sugar per 100ml)						
All Manufacturers	44.0 (-7.7, 95.7)	-0.1 (-0.9, 0.7)	**-117.2 (-183.3, -51.1)**	1.0 (-0.9, 2.9)	-73.2 (-157.1, 10.7)	0.9 (-1.2, 3.0)
Excluding Manufacturers with <1M Litres	41.5 (-9.7, 92.7)	-0.09 (-0.9, 0.7)	**-111.9 (-177.3, -46.6)**	-1.1 (-2.9, 0.8)	-70.8 (-153.4, 12.6)	-1.2 (-3.2, 0.9)
Excluding Manufacturers with <0.5M Litres	41.5 (-9.7, 92.7)	-0.09 (-0.9, 0.7)	**-111.9 (-177.3, -46.6)**	-1.1 (-2.9, 0.8)	-70.8 (-153.4, 12.6)	-1.2 (-3.2, 0.9)
Low levy tier (≥5g - <8g sugar per 100ml)						
All Manufacturers	-0.1 (-22.4, 22.1)	**-0.6 (-1.0, -0.3)**	-26.3 (-53.6, 1.0)	-0.8 (-1.6, 0.01)	-26.4 (-61.7, 8.8)	**-1.5 (-2.4, -0.6)**
Excluding Manufacturers with <1M Litres	-2.1 (-24.0, 19.9)_	**-0.7 (-1.0, -0.3)**	-20.9 (-47.5, 5.7)	**-0.9 (-1.8, -0.1)**	-23.0 (-57.5, 11.6)	**-1.6 (-2.5, -0.7)**
Excluding Manufacturers with <0.5M Litres	-2.1 (-24.0, 19.6)_	**-0.7 (-1.0, -0.3)**	-20.7 (-47.3, 5.9)	**-0.9 (-1.8, -0.1)**	-22.9 (-57.2, 11.5)	**-1.6 (-2.5, -0.7)**
Sugar						
High tier (≥8g sugar per 100ml)						
All Manufacturers	**8.9 (6.8, 10.9)**	**-0.05 (-0.08, -0.03)**	**-11.5 (-15.3, -7.7)**	**-0.1 (-0.2, -0.02)**	-2.6 (-6.9, 1.6)	**-0.2 (-0.3, -0.07)**
Excluding Manufacturers with <1M Litres	4.5 (-1.3, 10.2)	-0.03 (-0.1, 0.06)	**-13.5 (-20.8, -6.1)**	-0.07 (-0.3, 0.1)	-9.0 (-18.3, 0.4)	-0.1 (-0.3, 0.1)
Excluding Manufacturers with <0.5M Litres	4.5 (-1.3, 10.2)	-0.03 (-0.1, 0.06)	**-13.5 (-20.8, -6.1)**	-0.07 (-0.3, 0.1)	-9.0 (-18.3, 0.4)	-0.1 (-0.3, 0.1)
Low tier (≥5g - <8g sugar per 100ml)						
All Manufacturers	1.7 (-1.6, 5.1)	**-0.07 (-0.1, -0.02)**	**-6.2 (-10.6, -1.8)**	0.09 (-0.03, 0.2)	-4.5 (-10.0, 1.1)	0.02 (-0.1, 0.2)
Excluding Manufacturers with <1M Litres	-0.4 (-2.1, 1.3)	**-0.07 (-0.1, -0.04)**	**-2.5 (-4.6, -0.4)**	0.002 (-0.06, 0.06)	**-3.0 (-5.7, -0.2)**	-0.007 (-0.1, 0.0001)
Excluding Manufacturers with <0.5M Litres	-0.5 (-2.1, 1.2)	**-0.07 (-0.1, -0.04)**	**-2.5 (-4.7, -0.4)**	0.003 (-0.06, 0.06)	**-3.0 (-5.7, -0.2)**	-0.007 (-0.1, 0.0001)

For peer review only

Notes. Bold indicates a significant difference at the 95% confidence interval level.

Supplementary Table 4b: Modelled absolute and relative change in volume of, and sugar in, all drinks (ml) purchased per household (95% CI) per week in relation to the UK SDIL, March 2014- March 2019; excluding small manufacturers (weighted)

	Analysis 1: pre-post announcement (March 2014 – March 2018)		Analysis 2: pre-post implementation (March 2016 – March 2019)		Analysis 3: pre-post announcement & implementation (March 2014 – March 2019)	
	Absolute change (ml)	Relative change (%)	Absolute change (ml)	Relative change (%)	Absolute change (ml)	Relative change (%)
Volume						
High tier (≥8g sugar per 100ml)						
All Manufacturers	**34.7 (8.1, 61.4)**	**7.3 (1.7, 12.9)**	**-171.6 (-208.1, -135.1)**	**-42.5 (-51.6, -33.5)**	**-140.8 (-177.3, -104.3)**	**-37.8 (-47.6, -28.0)**
Excluding Manufacturers with <1M Litres	**35.3 (8.9, 61.6)**	**7.5 (1.9, 13.1)**	**-168.1 (-204.2, -131.9)**	**-42.6 (-51.7, -33.4)**	**-136.2 (-172.3, -100.1)**	**-37.6 (-47.5, -27.6)**
Excluding Manufacturers with <0.5M Litres	**35.3 (8.9, 61.6)**	**7.5 (1.9, 13.1)**	**-168.1 (-204.2, -131.9)**	**-42.6 (-51.7, -33.4)**	**-136.2 (-172.3, -100.1)**	**-37.6 (-47.5, -27.6)**
Low tier (≥5g - <8g sugar per 100ml)						
All Manufacturers	**-65.7 (-77.5, -53.8)**	**-37.1 (-43.7, -30.4)**	**-71.8 (-87.8, -55.8)**	**-71.8 (-87.8, -55.8)**	**-170.5 (-186.5, -154.5)**	**-85.8 (-93.9, -77.8)**
Excluding Manufacturers with <1M Litres	**-66.4 (-78.2, -54.6)**	**-37.6 (-44.3, -30.9)**	**-71.2 (-87.1, -55.3)**	**-72.5 (-88.7, -56.3)**	**-171.1 (-187.0, -155.2)**	**-86.4 (-94.4, -78.4)**
Excluding Manufacturers with <0.5M Litres	**-66.5 (-78.2, -54.7)**	**-37.6 (-44.2, -31.0)**	**-71.3 (-87.1, -55.5)**	**-72.4 (-88.5, -56.3)**	**-171.2 (-187.1, -155.4)**	**-86.3 (-94.3, -78.3)**
Sugar						
High tier (≥8g sugar per 100ml)						
All Manufacturers	**5.5 (3.8, 7.3)**	**10.8 (7.4, 14.1)**	**-21.2 (-23.8, -18.5)**	**-49.3 (-55.4, -43.1)**	**-16.2 (-18.8, -13.5)**	**-42.6 (-49.6, -35.6)**
Excluding Manufacturers with <1M Litres	**4.7 (1.7, 7.7)**	**9.3 (3.4, 15.1)**	**-19.3 (-23.4, -15.2)**	**-44.3 (-53.6, -35.0)**	**-14.6 (-18.7, -10.6)**	**-37.6 (-48.1, -27.1)**
Excluding Manufacturers with <0.5M Litres	**4.7 (1.7, 7.7)**	**9.3 (3.4, 15.1)**	**-19.3 (-23.4, -15.2)**	**-44.3 (-53.6, -35.0)**	**-14.6 (-18.7, -10.6)**	**-37.6 (-48.1, -27.1)**
Low tier (≥5g - <8g sugar per 100ml)						
All Manufacturers	**-4.3 (-6.1, -2.6)**	**-37.5 (-52.5,-22.5)**	**-5.0 (-7.4, -2.6)**	**-75.8 (-112.7, -38.9)**	**-11.5 (-13.9, -9.07)**	**-87.8 (-106.4, -69.2)**
Excluding Manufacturers with <1M Litres	**-4.7 (-5.5, -3.8)**	**-39.1 (-46.5, -31.7)**	**-4.8 (-6.0, -3.6)**	**-73.0 (-91.3, -54.7)**	**-11.8 (-13.0, -10.6)**	**-86.9 (-95.8, -78.1)**
Excluding Manufacturers with <0.5M Litres	**-4.7 (-5.6, -3.8)**	**-39.1 (-46.5, -31.8)**	**-4.8 (-6.0, -3.6)**	**-72.9 (-91.1, -54.6)**	**-11.8 (-13.0, -10.6)**	**-86.9 (-95.7, -78.0)**

Notes. Bold indicates a significant difference at the 95% confidence interval level.

For peer review only

Supplementary Figure 2. Observed and modelled volume (ml) and amount of sugar (g) in all drinks combined purchased per household per week, March 2014- March 2019 (weighted)

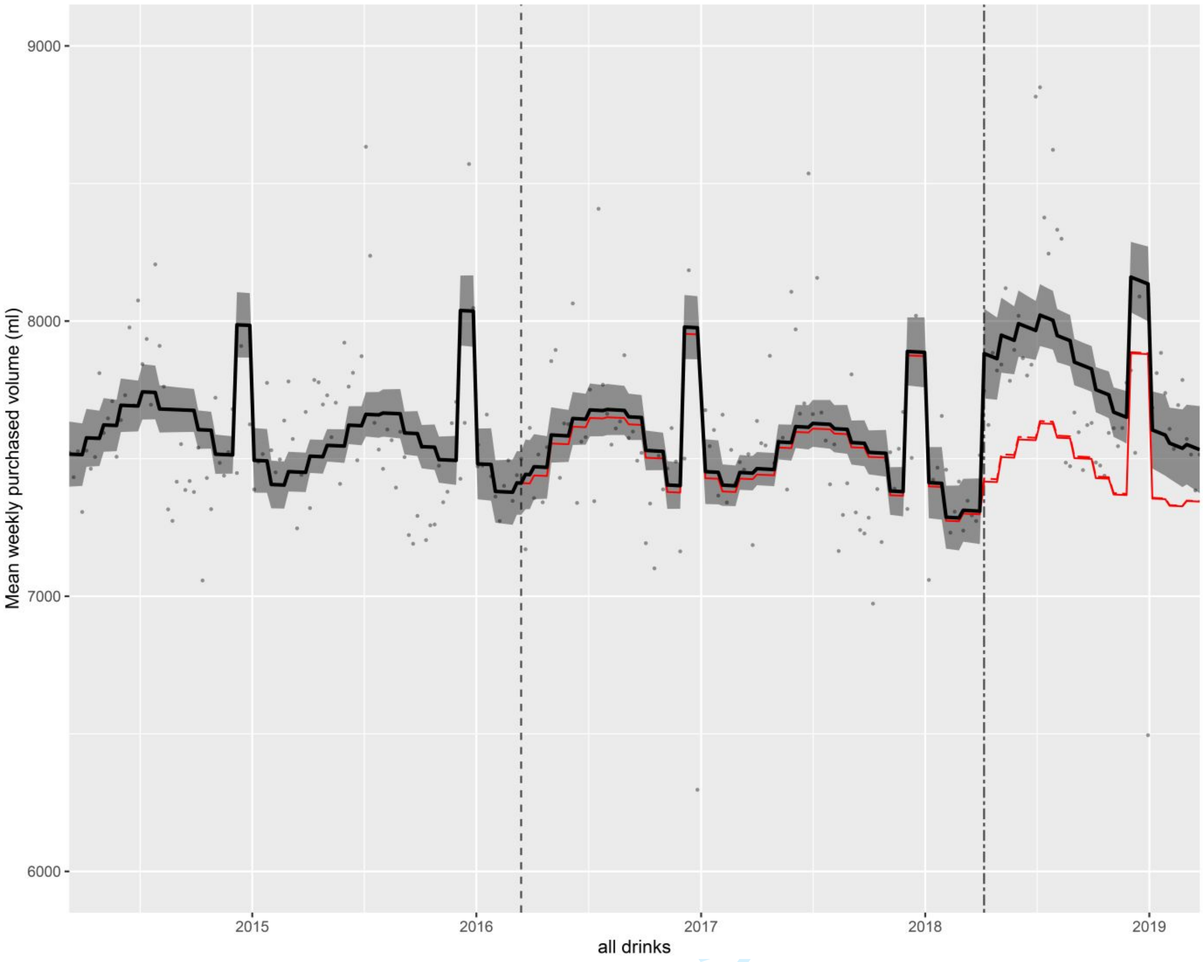

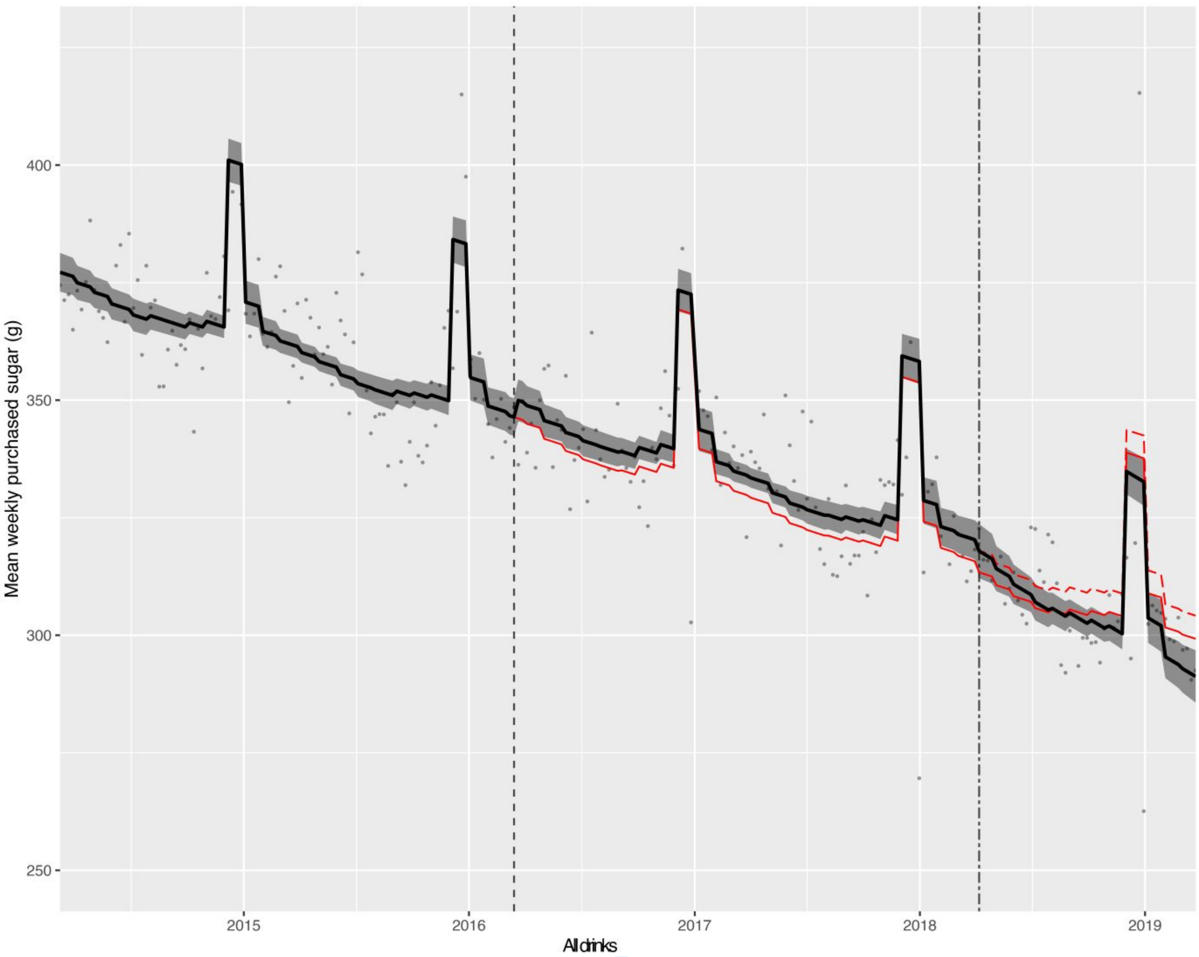

Notes. Points are observed data, black lines (with shadows) are modelled data (with 95% confidence intervals); red lines indicate the counterfactuals had the announcement (red solid line) and implementation (red dashed line) not happened; the first dashed vertical line indicates the point of announcement; the second dashed vertical line indicates the point of implementation; The Y-axis varies in scale between panels to maximise the resolution of figures; modelled purchases include averaged effects for seasonality and the impact of December and January (the Christmas period). The control category of toiletries is shown in Fig 3.

Supplementary material G

Removing the control category led to wider confidence intervals in a small number of cases such that absolute and relative changes in volume were not significantly different from the pre-implementation counterfactuals for no levy drinks and the pre-announcement and post implementation counterfactual for drinks containing 0g of sugar per 100ml. Significantly lower volumes of purchased volumes of powdered drinks were seen following the announcement in the controlled analysis unlike in the uncontrolled analysis. In the uncontrolled analysis absolute and relative differences in the amount of sugar in milk based drinks were significantly different from the pre-implementation counterfactual but not significantly different when examining the impact of the SDIL overall. Additionally the amount of sugar in confectionery was not significantly different from the pre-announcement counterfactual in the uncontrolled ITS analysis unlike in the controlled analysis.

For peer review only

Supplementary Table 5a: Level and trend changes in volume of drinks (ml) and confectionery (g) purchased per household per week (95% CI) in relation to the UK SDIL, March 2014 - March 2019 without toiletries as a control condition (weighted)

	Analysis 1: pre-post announcement (March 2014 – March 2018)		Analysis 2: pre-post implementation (March 2016 – March 2019)		Analysis 3: pre-post announcement & implementation (March 2014 – March 2019)	
	Level Change	Trend Change	Level Change	Trend Change	Level Change	Trend Change
Levy liable drinks						
High tier (≥8g sugar per 100ml)	42.3 (-9.4, 94.0)	-0.07 (-0.9, 0.8)	**-111.2 (-177.1, -45.3)**	-1.2 (-3.0, 0.7)	-68.9 (-152.7, 14.9)	-1.3 (-3.3, 0.8)
Low tier (≥5g - <8g sugar per 100ml)	-2.2 (-24.1, 19.8)	**-0.6 (-1.0, -0.2)**	-18.9 (-45.8, 8.0)	-1.0 (1.9, -0.2)	-21.1 (-55.75, 13.6)	**-1.7 (-2.6, -0.7)**
No levy (<5g sugar per 100ml)	-29.8 (-150.9, 91.3)	**2.0 (0.08, 4.0)**	**378.1 (223.7, 532.5)**	**0.3 (0.5, 2.0)**	**348.3 (152.0, 544.6)**	2.4 (-2.4, 7.2)
>0g to <5g sugar per 100ml	-27.9 (-78.3, 22.5)	**1.3 (0.5, 2.0)**	**98.0 (32.7, 163.3)**	**2.1 (0.3, 3.9)**	70.1 (-12.4, 152.6)	**3.4 (1.4, 5.4)**
0g sugar per 100ml	-2.0 (-75.5, 71.5)	0.8 (-0.4, 2.0)	**278.9 (185.6, 372.2)**	-1.7 (-4.4, 1.0)	**276.9 (158.2, 395.7)**	-0.9 (-3.9, 2.0)
Bottled water	9.9 (-49.1, 68.9)	-0.4 (-1.4, 0.6)	53.5 (-20.8, 127.8)	-2.6 (-4.7, 0.4)	63.4 (-31.5, 158.2)	**-2.9 (-5.3, -0.6)**
Levy exempt drinks						
Alcoholic drinks	-13.3 (-51.0, 24.4)	-0.03 (-0.4, 0.4)	**212.7 (142.5, 282.9)**	**-5.8 (-7.9, -3.8)**	**199.4 (119.8, 279.1)**	**-5.9 (-8.0, -3.8)**
Milk and milk based drinks[1]	26.0 (-35.7, 87.7)	**-1.1 (-2.0, -0.09)**	75.7 (-4.5, 155.9)	0.6 (-1.6, 2.8)	**101.7 (0.52, 202.9)**	-0.5 (-2.9, 2.0)
No added sugar fruit juices	-9.9 (-35.0, 15.2)	0.2 (-0.3, 0.6)	**40.9 (10.1, 71.7)**	-0.9 (-1.9, 0.02)	31.0 (-8.73, 70.7)	-0.8 (-1.8, 0.3)
Drinks sold as powders (g)	-3.4 (-9.01, 2.20)	-0.03 (-0.1, 0.06)	5.66 (-1.6, 12.9)	0.07 (-0.1, 0.3)	2.25 (-6.90, 11.4)	0.04 (-0.2, 0.3)
Confectionery (g)	-20.5 (-90.8, 49.8)	0.7 (-1.3, 1.5)	-70.6 (-155.8, 14.6)	**2.2 (0.7, 3.7)**	-91.1 (-201.5, 19.3)	2.3 (-2.2, 6.8)

Notes.Bold indicates a significant difference at the 95% confidence interval level. [1]Milk comprises drinks in the following categories: semi-skimmed; specific low fat % milk (e.g. 1% fat milk); whole milk; buttermilk; modified milk; other milk; other non-cows milk; rice drink; soya milk. Skimmed milk is excluded from all analysis in this paper due to missing data.

For peer review only

Supplementary Table 5b: Level and trend changes in sugar in drinks (ml) and confectionery (g) purchased per household per week (95% CI) in relation to the UK SDIL, March 2014 - March 2019 without toiletries as a control condition (weighted)

	Analysis 1: pre-post announcement (March 2014 – March 2018)		Analysis 2: pre-post implementation (March 2016 – March 2019)		Analysis 3: pre-post announcement & implementation (March 2014 – March 2019)	
	Level Change	Trend Change	Level Change	Trend Change	Level Change	Trend Change
Levy liable drinks						
High tier (≥8g sugar per 100ml)	4.8 (-1.0, 10.6)	-0.008 (-0.1, 0.09)	**-12.1 (-19.5, -4.8)**	-0.2 (-0.4, 0.06)	-7.3 (-16.6, 2.0)	-0.2 (-0.4, 0.07)
Low tier (≥5g - <8g sugar per 100ml)	-0.2 (-1.7, 1.2)	**-0.4 (-0.5, -0.4)**	-1.2 (-2.9, 0.6)	**-0.07 (-0.1, -0.01)**	-1.4 (-3.7, 0.9)	**-0.5 (-0.6, -0.5)**
No levy (<5g sugar per 100ml)	1.7 (-0.2, 3.3)	**0.07 (0.04, 0.1)**	**5.2 (3.2,7.2)**	**0.08 (0.02, 0.1)**	**6.9 (4.4, 9.5)**	**0.2 (0.09, 0.2)**
>0g to <5g sugar per 100ml	1.7 (-0.2, 3.3)	**0.07 (0.04, 0.1)**	**5.2 (3.2,7.2)**	**0.08 (0.02, 0.1)**	**6.9 (4.4, 9.5)**	**0.2 (0.09, 0.2)**
Levy exempt drinks						
Milk and milk based drinks[1]	2.4 (-0.6, 5.3)	**-0.06 (-0.1, -0.02)**	3.7 (-0.1, 7.5)	0.01 (-0.10, 0.1)	**6.1 (1.3, 10.9)**	-0.05 (-0.2, 0.06)
No added sugar fruit juices	-1.1 (-3.5, 1.4)	0.03 (-0.03, 0.07)	**3.3 (0.3, 6.3)**	**-0.10 (-0.2, -0.01)**	2.3 (-1.6, 6.1)	-0.07 (-0.2, 0.03)
Drinks sold as powders (g)	-1.0 (-3.1, 1.1)	0.01 (-0.02, 0.05)	0.4 (-2.2, 3.0)	-0.01 (-0.1, 0.07)	-0.6 (-3.9, 2.7)	0.001 (-0.09, 0.09)
Confectionery (g)	-11.7 (-51.5, 28.1)	0.05 (-0.8, 0.9)	-42.6 (-90.8, 5.6)	1.3 (-0.4, 3.0)	-54.3 (-116.8, 8.2)	1.3 (-0.5, 3.2)

Notes. Bold indicates a significant difference at the 95% confidence interval level. [1]Milk comprises drinks in the following categories: semi-skimmed; specific low fat % milk (e.g. 1% fat milk); whole milk; buttermilk; modified milk; other milk; other non-cows milk; rice drink; soya milk. Skimmed milk is excluded from all analysis in this paper due to missing data.

For peer review only

Supplementary Table 5c: Absolute and relative changes in volume of drinks (ml) and confectionery (g) purchased per household per week (95% CI) in relation to the UK SDIL, March 2014 - March 2019 without toiletries as a control condition (weighted)

	Analysis 1: pre-post announcement (March 2014 – March 2018)		Analysis 2: pre-post implementation (March 2016 – March 2019)		Analysis 3: pre-post announcement & implementation (March 2014 – March 2019)	
	Absolute change (ml or g)	Relative change (%)	Absolute change (ml or g)	Relative change (%)	Absolute change (ml or g)	Relative change (%)
Levy liable drinks						
High tier (≥8g sugar per 100ml)	34.4 (-4.5, 73.3)	7.3 (-1.0, 15.6)	**-171.6 (-223.1, -120.0)**	**-42.5 (-55.3, -29.7)**	**-140.9 (-192.5, -89.4)**	**-37.8 (-51.6, -24.0)**
Low tier (≥5g - <8g sugar per 100ml)	**-68.3 (-85.7,-50.9)**	**-38.2 (-47.9, -28.4)**	**-71.4 (-94.1, -48.7)**	**-71.5 (-94.2, -48.8)**	**-171.3 (-193.9, -148.6)**	**-85.8 (-97.1, -74.4)**
No levy (<5g sugar per 100ml)	**187.1 (95.7, 278.5)**	**11.5 (5.9, 17.1)**	**395.0 (273.9, 516.0)**	**19.8 (13.7, 25.9)**	**685.5 (564.4, 806.5)**	**40.2 (33.1, 47.3)**
>0g to <5g sugar per 100ml	**106.4 (68.7, 144.2)**	**17.2 (11.1, 23.3)**	**205.9 (155.5, 256.3)**	**25.6 (19.3, 31.8)**	**374.6 (326.0, 426.8)**	**59.3 (51.4, 67.2)**
0g sugar per 100ml	**80.7 (25.1, 136.4)**	**8.0 (2.5, 13.5)**	**191.9 (118.2, 265.5)**	**16.1 (9.9, 22.3)**	**312.0 (238.4, 385.6)**	**29.1 (22.3, 36.0)**
Bottled water	**-31.3 (-76.3, 13.7)**	**-4.4 (-10.6, 1.91)**	**-76.9 (-136.6, -17.2)**	**-9.89 (-17.6, -2.2)**	**-127.8 (-187.6, -68.1)**	**-15.4 (-22.6, -8.2)**
Levy exempt drinks						
Alcoholic drinks	-16.6 (-62.2, 28.9)	1.0 (-3.6, 1.7)	**-84.9 (-155.8, -14.0)**	**-4.81 (-8.8, -0.8)**	**-103.1 (-174.0, -32.2)**	**-5.8 (-9.8, -1.8)**
Milk and milk based drinks[1]	**-85.9 (-39.6, 132.2)**	**-2.3 (-3.6, -1.1)**	**106.4 (44.5, 168.3)**	**3.03 (1.3, 4.8)**	-32.8 (-94.6, 29.1)	-0.9 (-2.6, 0.8)
No added sugar fruit juices	5.9 (-13.9, 25.8)	1.2 (-2.9, 5.4)	-6.56 (-32.7, 19.6)	-1.33 (-6.6, 4.0)	6.95 (-19.2, 33.1)	1.5 (-4.0, 6.9)
Drinks sold as powders (g)	**-6.8 (-11.0, -2.57)**	**-6.8 (-10.9, -2.6)**	**9.3 (3.7, 14.9)**	**10.8 (4.3, 17.4)**	0.89 (-4.7, 6.5)	1.0 (-5.0, 6.9)
Confectionery (g)	-9.5 (-72.7, 53.7)	-2.3 (-17.7, 13.0)	40.6 (-42.1, 123.2)	11.9 (-12.3, 36.0)	36.3 (-46.4, 118.9)	10.5 (-13.4, 34.3)

Notes. Bold indicates a significant difference at the 95% confidence interval level. [1]Milk comprises drinks in the following categories: semi-skimmed; specific low fat % milk (e.g. 1% fat milk); whole milk; buttermilk; modified milk; other milk; other non-cows milk; rice drink; soya milk. Skimmed milk is excluded from all analysis in this paper due to missing data.

For peer review only

Supplementary Table 5d: Absolute and relative changes in sugar in drinks (ml) and confectionery (g) purchased per household per week (95% CI) in relation to the UK SDIL, March 2014 - March 2019 without toiletries as a control condition (weighted)

	Analysis 1: pre-post announcement (March 2014 – March 2018)		Analysis 2: pre-post implementation (March 2016 – March 2019)		Analysis 3: pre-post announcement & implementation (March 2014 – March 2019)	
	Level Change	Trend Change	Level Change	Trend Change	Level Change	Trend Change
Levy liable drinks						
High tier (≥8g sugar per 100ml)	3.9 (-0.4, 8.3)	7.6 (-0.78, 15.9)	**-19.6 (-25.3, -13.8)**	**-43.8 (-56.6, -30.9)**	**-16.1 (-21.8, -10.3)**	**-39.0 (-52.9, -25.0)**
Low tier (≥5g - <8g sugar per 100ml)	**-4.9 (-6.1, -3.8)**	**-40.3 (-49.6, -30.9)**	**-4.7 (-6.2, -3.2)**	**-70.9 (-93.3, -48.4)**	**-11.9 (-13.4, -10.4)**	**-86.0 (-96.8, -75.2)**
No levy (<5g sugar per 100ml)	**6.1 (4.9, 7.3)**	**77.7 (62.6, 92.8)**	**9.3 (7.8, 10.9)**	**52.5 (43.6, 61.4)**	**19.2 (17.6, 20.8)**	**240.9 (221.0, 260.8)**
>0g to <5g sugar per 100ml						
Levy exempt drinks						
Milk and milk based drinks[1]	**-4.2 (-6.4, -2.0)**	**-2.3 (-3.6, -1.1)**	**4.1 (1.1, 7.0)**	**2.4 (0.7, 4.2)**	**-3.2 (-6.5,-0.3)**	**-1.8 (-3.5, -0.1)**
No added sugar fruit juices	**2.6 (0.7, 4.6)**	**5.9 (1.5, 10.2)**	-1.8 (-4.3, 0.8)	-3.7 (-9.1, 1.6)	**2.6 (0.04, 5.2)**	**5.9 (0.08, 11.8)**
Drinks sold as powders (g)	0.4 (-1.2, 2.1)	2.1 (-5.9, 10.0)	-0.3 (-2.4, 1.9)	-1.3 (-12.5, 10.0)	0.9 (-1.3,3.0)	4.7 (-7.2, 16.7)
Confectionery (g)	-5.9 (-42.0, 30.1)	-2.6 (-18.1, 13.0)	23.4 (-24.0, 70.7)	12.1 (-12.4, 36.7)	20.2 (-27.1, 67.6)	10.3 (-13.9, 34.5)

Notes.[†]Trend[2], Bold indicates a significant difference at the 95% confidence interval level. [1]Milk comprises drinks in the following categories: semi-skimmed; specific low fat % milk (e.g. 1% fat milk); whole milk; buttermilk; modified milk; other milk; other non-cows milk; rice drink; soya milk. Skimmed milk is excluded from all analysis in this paper due to missing data.

For peer review only

BMJ Open

Changes in soft drinks purchased by British households associated with the UK soft drinks industry levy: a controlled interrupted time series analysis

Journal:	*BMJ Open*
Manuscript ID	bmjopen-2023-077059.R1
Article Type:	Original research
Date Submitted by the Author:	13-Oct-2023
Complete List of Authors:	Rogers , Nina ; MRC Epidemiology Unit<br>Pell, David; MRC Epidemiology Unit<br>Mytton, Oliver; MRC Epidemiology Unit; University College London Great Ormond Street Institute of Child Health<br>Penney, Tarra; York University - Keele Campus<br>Briggs, Adam; University of Oxford, Nuffield Department of Population Health; University of Warwick Warwick Medical School, Institute for Health Policy and Clinical Practice<br>Cummins, Steven; London School of Hygiene & Tropical Medicine, Department of Social and Environmental Health Research<br>Penn-Jones, Catrin; MRC Epidemiology Unit<br>Rayner, Mike; University of Oxford, Nuffield Department of Population Health; NIHR Oxford Biomedical Research Centre<br>Rutter, Harry; University of Bath Department of Social and Policy Sciences<br>Scarborough, Peter; Centre on Population Approaches for Non-Communicable Disease Prevention, Nuffield Department of Population Health; NIHR Oxford Biomedical Research Centre<br>Sharp, Stephen; MRC Epidemiology Unit<br>Smith, Richard ; University of Exeter,<br>White, Martin; MRC Epidemiology Unit<br>Adams, J; MRC Epidemiology Unit
**Primary Subject Heading**:	Public health
Secondary Subject Heading:	Health policy
Keywords:	Obesity, PUBLIC HEALTH, NUTRITION & DIETETICS

SCHOLARONE™
Manuscripts

I, the Submitting Author has the right to grant and does grant on behalf of all authors of the Work (as defined in the below author licence), an exclusive licence and/or a non-exclusive licence for contributions from authors who are: i) UK Crown employees; ii) where BMJ has agreed a CC-BY licence shall apply, and/or iii) in accordance with the terms applicable for US Federal Government officers or employees acting as part of their official duties; on a worldwide, perpetual, irrevocable, royalty-free basis to BMJ Publishing Group Ltd ("BMJ") its licensees and where the relevant Journal is co-owned by BMJ to the co-owners of the Journal, to publish the Work in this journal and any other BMJ products and to exploit all rights, as set out in our licence.

The Submitting Author accepts and understands that any supply made under these terms is made by BMJ to the Submitting Author unless you are acting as an employee on behalf of your employer or a postgraduate student of an affiliated institution which is paying any applicable article publishing charge ("APC") for Open Access articles. Where the Submitting Author wishes to make the Work available on an Open Access basis (and intends to pay the relevant APC), the terms of reuse of such Open Access shall be governed by a Creative Commons licence – details of these licences and which Creative Commons licence will apply to this Work are set out in our licence referred to above.

Other than as permitted in any relevant BMJ Author's Self Archiving Policies, I confirm this Work has not been accepted for publication elsewhere, is not being considered for publication elsewhere and does not duplicate material already published. I confirm all authors consent to publication of this Work and authorise the granting of this licence.

For peer review only

Changes in soft drinks purchased by British households associated with the UK soft drinks industry levy: a controlled interrupted time series analysis

Nina T Rogers, research associate[1]

David Pell, research associate[1(when this work was conducted)]

Oliver Mytton, clinical lecturer[1(when this work was conducted), 9(currently)]

Tarra L Penney, assistant professor[1,2]

Adam Briggs, honorary associate clinical professor[3,4]

Steven Cummins, professor[5]

Catrin Jones, research associate[1]

Mike Rayner, professor[3,6]

Harry Rutter, professor[7]

Peter Scarborough, professor[3,6]

Stephen J Sharp, senior statistician[1]

Richard D Smith, professor[8]

Martin White, professor[1]

Jean Adams, professor [1]

[1]MRC Epidemiology Unit, University of Cambridge School of Clinical Medicine, Institute of Metabolic Science, Cambridge CB2 0QQ, UK

[2]Faculty of Health, School of Global Health, York University, Toronto, Canada

[3]Nuffield Department of Population Health, University of Oxford, Oxford, UK

[4]Warwick Medical School, University of Warwick, Division of Health Sciences, Coventry, UK

[5]Population Health Innovation Lab, Department of Public Health, Environment and Society, London School of Hygiene and Tropical Medicine, London, UK

[6]National Institute of Health Research Oxford Biomedical Research Centre, Oxford University Hospitals NHS Foundation Trust, Headington, Oxford, UK

[7]Department of Social and Policy Sciences, University of Bath, Bath, UK

[8]College of Medicine and Health, University of Exeter, Exeter, UK

[9]Great Ormond Street Institute of Child Health, University College London, London, UK

Correspondence to:
Jean Adams
jma79@medschl.cam.ac.uk
(@jeanmadams on Twitter)

For peer review only - http://bmjopen.bmj.com/site/about/guidelines.xhtml
For peer review only

Abstract

Objective To determine changes in household purchases of drinks one year after implementation of UK soft drinks industry levy (SDIL).
Design Controlled interrupted time series.
Participants Households reporting their purchasing to market research company (average weekly n=22,091), March 2014 to March 2019.
Intervention A two tiered tax levied on soft drinks manufacturers, announced in March 2016 and implemented in April 2018. Drinks with ≥8g sugar/100mL (high tier) are taxed at £0.24/L, drinks with ≥5 to <8g sugar/100mL (low tier) are taxed at £0.18/L.
Main outcome measures Absolute and relative differences in the volume of, and amount of sugar in, soft drinks categories, all soft drinks combined, alcohol, and confectionery purchased per household per week one year after implementation.
Results In March 2019, compared with the counterfactual, purchased volume of high tier drinks decreased by 140.8mL (95% confidence interval 104.3-177.3mL) per household per week, equivalent to 37.8% (47.6-28.0%), and sugar purchased in these drinks decreased by 16.2g (13.5-18.8g), or 42.6% (35.6-49.6%). Purchases of low tier drinks decreased by 170.5mL (154.5-186.5mL) or 85.8% (77.8-93.9%), with an 11.5g (9.1-13.9g) reduction in sugar in these drinks, equivalent to 87.8% (69.2-106.4%). When all soft drinks were combined irrespective of levy tier or eligibility, the volume of drinks purchased increased by 188.8mL (30.7-346.9mL) per household per week, or 2.6% (0.4-4.7%), but sugar decreased by 8.0g (2.4-13.6g), or 2.7% (0.8-4.5%). Purchases of confectionery and alcoholic drinks did not increase.
Conclusions Compared with trends before the SDIL was announced, one year after implementation, volume of soft drinks purchased increased by 189mL, or 2.6% per household per week. The amount of sugar in those drinks was 8g, or 2.7%, lower per household per week. The SDIL might benefit both public health and industry. Further studies should determine whether and how apparently small effect sizes translate into health outcomes.
Study registration ISRCTN18042742.

Strengths and limitations of this study

- We used a large, nationally representative dataset, included a control category, and explored changes in two potential substitute categories (alcohol and confectionery).
- We only included purchases brought into homes.
- We did not assess changes in other categories beyond soft drinks, alcohol, and confectionery.
- The estimate of effect size in interrupted time series analyses is based on a modelled counterfactual that might be inaccurate.
- Attribution of effects in interrupted time series analyses is vulnerable to time varying confounding such as co-interventions.

For peer review only - http://bmjopen.bmj.com/site/about/guidelines.xhtml

Introduction

High consumption of sugar sweetened beverages (SSBs) is associated with increased risk of dental caries, obesity, type 2 diabetes, and cardiovascular disease.[1-3] The World Health Organization recommends the use of SSB taxes to reduce consumption.[4] A systematic review of studies published to June 2018 suggests that SSB taxes lead to decreases in the sales, purchasing and consumption of taxed drinks.[5] More recent findings support this conclusion.[6-10] Although price is one important mediator of these changes,[11-16] other potential mechanisms include reformulation of products to reduce sugar concentration, smaller portion sizes, and increases in the perception of SSBs being harmful to health associated with them being grouped with other taxed products such as alcohol and tobacco.[17] Furthermore, any public health benefits of reduced SSB consumption associated with SSB taxes might be negated by increased consumption of substitutes such as confectionery and alcohol.[18-20]

The UK soft drinks industry levy (SDIL) was one of the first taxes on SSBs explicitly designed to incentivise manufacturers of SSBs to reduce sugar content.[21 22] This is reflected in three design features. Firstly, the SDIL is levied on manufacturers, importers, and bottlers rather than on consumers. Secondly, the levy includes two tiers: £0.24/L for drinks containing ≥8 g total sugar per 100 mL, and £0.18/L for drinks containing ≥5 g and <8 g total sugar per 100 mL. Thirdly, the SDIL was intentionally announced in 2016, two years before implementation in 2018, to allow manufacturers time to adjust. The SDIL also provides exemptions (see Box 1).[23]

Box 1. Glossary of terms

Soft drinks industry levy (SDIL)—a tiered tax on manufacturers of sugar sweetened beverages

Levy exempt drinks—drinks exempt from the SDIL irrespective of sugar content; that is, drinks containing >75% milk, drinks containing >1.2% alcohol, and drinks sold as alcohol replacements, drinks sold as powders, 100% fruit juices, and drinks sold by manufacturers selling less than one million litres of drinks not exempt for other reasons each year

High tier drinks—drinks that are not levy exempt and contain ≥8 g of sugar per 100 mL

Low tier drinks—drinks that are not levy exempt and contain ≥5 g to <8 g of sugar per 100 mL

No levy drinks—drinks that are not levy exempt but contain <5 g of sugar per 100 mL; we subdivided this category into drinks containing >0 g to <5 g of sugar per 100 mL, drinks containing 0 g of sugar per 100 mL, and bottled water

Levy liable drinks—drinks that are not levy exempt drinks; that is, the sum of high tier drinks, low tier drinks, and no levy drinks

Soft drinks—any drink not containing alcohol

Confectionery—products in the sugar confectionery and chocolate confectionery categories

For peer review only - http://bmjopen.bmj.com/site/about/guidelines.xhtml

Toiletries—products in the shampoo, hair conditioner, and liquid soap categories

Two before and after analyses have shown reductions of around 30% in sales weighted sugar concentration of levy eligible drinks in the UK from before the announcement of the SDIL on 16 March 2016 to after implementation on 6 April 2018.[24 25] However, background trends in purchases of sugary drinks are not stable, with decreases reported over several years.[26] This makes it difficult to attribute before and after decreases in sugary drinks purchases to the SDIL. An interrupted time series analysis found that the announcement and implementation of the SDIL were together associated with a 34 percentage point reduction in the proportion of levy liable drinks with >5 g total sugar per 100 mL, indicating substantial reformulation of the market.[15] Changes in prices across the UK soft drink market were also reported, although it was difficult to discern clear patterns in these, with some levied categories increasing and others decreasing in price. In a controlled interrupted time series analysis including data up to the point of SDIL implementation, we found that the SDIL announcement was associated with changes in both the volume of, and sugar purchased in, drinks in many categories.[27] Overall we found no change in total volume of purchases of all soft drinks combined, but a small increase in sugar purchased from soft drinks of 5.3g per household per week, or 1.7%.

In this paper our aim was to determine whether household purchases of drinks and confectionery had changed one year after implementation of the SDIL.

Methods

Here we extend our previous analyses[27] to study changes in the volume of, and amount of sugar in, household purchases of drinks in each levy tier, exempt drinks categories (including alcoholic drinks), and confectionery from two years before the announcement of the SDIL to one year after its implementation (March 2014 to March 2019). As before, we used controlled interrupted time series methods, with toiletries included as a control category.[27] We compared observed changes associated with the announcement and implementation of the SDIL to the counterfactual scenarios in which the announcement and implementation did not take place. Including a full two years of data before the announcement enables us to estimate pre-intervention trends and project these forward as counterfactual scenarios. The protocol is published elsewhere[28] and the study was registered. This study is reported in accordance with the strengthening the reporting of observational studies in epidemiology (STROBE) guideline (see Supplementary material A).

For peer review only - http://bmjopen.bmj.com/site/about/guidelines.xhtml

Data source

We used data from a panel of households reporting their purchasing on a weekly basis to a market research company (Kantar Worldpanel; KWP). Participating households are asked to record all food and drink purchases brought into the home (including those ordered online and delivered) through barcodes scanners and manual report. Purchasing information is uploaded weekly, where it is linked to nutritional data collected by KWP field workers on a rolling basis. Households record their personal characteristics and receive gift vouchers worth about £100 ($122; €112) annually—equivalent to 0.3% of median UK annual household income after tax in 2019 (£29 600).[29]

KWP samples households from across Great Britain (GB) using proprietary methods, aiming to achieve a sample that is demographically representative of GB households. It excludes households that record fewer than six purchases weekly along with those whose adjusted weekly spend is lower than an undisclosed minimum. KWP applies proprietary weights to purchases to adjust for these exclusions and maintain the representativeness of the panel. We used these weights throughout.

The main data cleaning that occurred before analysis involved assigning products and product groups in the KWP dataset to SDIL relevant groups. This was done based on KWP assigned product groups, product names, and nutritional content. In previous work we found some evidence of error, but not bias, in the sugar concentration reported by KWP compared with information provided on manufacturers' websites.[27]

Product categories: drinks, confectionery, and toiletries

Purchased drinks that were levy liable were divided into high tier, low tier, or no levy based on sugar content (see box 1 for definitions). No levy drinks were additionally disaggregated, as described in box 1.

As the SDIL might have led to substitution to other drinks categories, we also examined purchasing of levy exempt drinks in several categories: milk based drinks (comprising milk, milk alternatives such as soya drinks, and yoghurt based juices and drinks), alcoholic drinks (comprising both alcoholic and alcohol replacement drinks), no added sugar fruit juices, and drinks sold as powder (eg, tea, coffee, hot chocolate). Other exempt categories (infant formulas and drinks sold for medical purposes) were excluded.

We also hypothesised that the SDIL might lead to substitution from sugary drinks to other high sugar categories. To investigate this, we used sugar and chocolate confectionery purchases (referred to as confectionery).

For peer review only - http://bmjopen.bmj.com/site/about/guidelines.xhtml

For peer review only

Control group

To control for background trends in household purchases we used purchases of shampoo, hair conditioner, and liquid soap (ie, toiletries). Toiletries meet the proposed criteria for a controlled interrupted time series: they are robust to seasonality and may have similar purchase volumes by households regardless of socioeconomic position or other potential confounders.[30]

Outcome measures

Most evaluations of SSB taxes focus on volume of drinks purchased. However, the SDIL's focus on reformulation makes the sugar purchased in drinks of additional public health interest. Thus, the outcome measures of interest were mean volume purchased per household per week in each of the drink categories and grams per household per week of confectionery; and mean sugar purchased per household per week from each of the drink categories and confectionery. Data were aggregated at the weekly level and analysed as a time series.

Overall analysis strategy

Previous evidence indicates that reformulation occurred after the announcement of the SDIL and price changes after implementation.[15] As such, we hypothesised the SDIL might act as two linked interventions: the announcement on 16 March 2016 and implementation on 6 April 2018.[17] Thus, our analysis strategy involved three separate comparisons that isolate the announcement and implementation of the SDIL and then examine the combined effect (Figure 1). In the first analysis we isolated the announcement of the SDIL. Here we compared anticipatory effects on purchasing two years after the announcement to the counterfactual estimated from purchasing in the two years before the announcement. This replicates and updates our previous analysis[27] as we anticipate that the stabilising effect of including additional post-announcement data likely reduces error. In the second analysis, we isolated the implementation of the SDIL. Here we compared purchasing one year after implementation to the counterfactual estimated from purchasing in the four years before implementation. In the third analysis we considered both the announcement and the implementation and we compared purchasing one year after implementation (ie three years after announcement) to the counterfactual estimated from purchasing in the two years before the announcement.

Throughout, we used the proprietary weights provided by KWP.

Primary analysis: category specific analyses

For peer review only - http://bmjopen.bmj.com/site/about/guidelines.xhtml
For peer review only

For each of the three analyses we developed separate controlled interrupted time series models for volume and sugar purchased from each levy liable and levy exempt drinks category and confectionery (Figure 1). Supplementary material B provides the full model specification.

We present absolute and relative differences between observed purchasing and counterfactual scenarios in the final week of each observation period, with standard errors used to calculate 95% confidence intervals for the relative difference obtained using the delta method.[31]

Secondary analysis: all soft drinks categories combined, irrespective of levy eligibility

Levy exempt drinks include drinks that might contain comparable amounts of sugar to levy liable products. To examine the extent to which the SDIL impacted on the purchased volume of, and amount of sugar in, soft drinks, regardless of SDIL liability, we carried out controlled interrupted time series analysis, combining purchases of all soft drinks irrespective of sugar content (ie, high tier, low tier, no levy, milk and milk based drinks, no added sugar fruit juice, and drinks sold as powders), levy liable drinks irrespective of sugar content (ie, high tier, low tier, and no levy drinks), and according to sugar content based on levy tiers irrespective of levy eligibility (ie, all soft drinks with ≥8 g of sugar per 100 mL, all soft drinks with ≥5 g to <8 g of sugar per 100 mL, and all soft drinks with <5 g of sugar per 100 mL).

Sensitivity analysis: excluding small manufacturers

The SDIL exempts drinks from manufacturers and producers who sell less than one million litres of levy liable drinks annually. As we were unable to obtain a list of exempt manufacturers, our main analyses include all manufacturers. We conducted sensitivity analyses to examine the effect of excluding manufacturers who we estimated to be small. The total purchase volume was summed by manufacturer by year across the five years in the KWP dataset, and a mean purchase volume per year for each manufacturer was calculated. In the first sensitivity analysis, we excluded manufacturers with a mean of less than one million litres purchased per year. Acknowledging KWP data excludes purchases not brought home, we repeated these analyses excluding manufacturers with mean annual purchased volumes of <0.5 million litres in KWP. We were unable to access accurate estimates of the proportion of all drinks purchases brought home. This value reflects an arbitrary, but we think conservative, estimate of the minimum proportion of drinks brought home.

Sensitivity analysis: interrupted time series without a control category

For peer review only - http://bmjopen.bmj.com/site/about/guidelines.xhtml

Toiletries were chosen as a control condition a priori to account for background trends in household purchases. It is, however, possible that a more appropriate control exists. As we only have access to data on purchasing of the categories described here (confectionery, drinks, toiletries), we were not able to examine alternative potential control categories. To examine the effect of the decision to use toiletries as the control category, we performed an additional sensitivity analysis with no control condition.

Changes to the protocol

We made several changes to the published protocol.[28] KWP provided additional data that allowed us to increase the precision of our estimates. Specifically, we were able to increase the pre-announcement study period from 104 to 107 weeks and reduce the unit of analysis from purchases every four weeks to purchases every week. We originally intended to include purchases not brought home. We excluded these purchases, however, as these data were not available before mid-2015, meaning that robust pre-announcement trends could not be estimated. Although we originally intended to combine all no levy drinks, we present these disaggregated into those with >0 g and <5 g of sugar per 100 mL, 0 g of sugar per 100 mL, and bottled water, as trends for these different categories are noticeably different. Our original intention to explore potential disparities across socioeconomic groups will be pursued in future work.

Patient and public involvement

The steering group for the wider SDIL evaluation includes two lay members and meets twice a year. Patients and the public were not involved in developing the research question, the outcome measures, the design, or the conduct of the work reported here. The steering group has regularly contributed ideas for routes to dissemination.

Correction of Pell et al (2021)

This paper is a corrected version of Pell et al (2021),[32], now retracted, which was originally published in the *BMJ*. The analysis presented in the original Pell et al (2021) paper included an incorrect weighting variable. This variable was incorrectly calculated as the inverse of what it should have been. The variable was also redundant to the analysis as it replicated a component of a second weighting variable also included (the "proprietary weights provided by KWP" mentioned above). The current corrected version replicates the original analysis without this redundant and incorrectly calculated weighting variable. The second, correct, weighting variable (the "proprietary weights provided by KWP" mentioned above) remains included. The authors identified the error themselves and alerted the journal and readers.[33]

For peer review only - http://bmjopen.bmj.com/site/about/guidelines.xhtml

Results

About 31 million purchases of drinks, confectionery, and toiletries from March 2014 to March 2019 were included from a mean of 22 091 households each week. The characteristics of included households remained consistent over the study period, and after weighting they largely reflected households in 2014-19 in the UK (see Supplementary table 1).

Table 1 summarises households' weekly purchased volumes of, and amounts of sugar in, drinks and other categories over the study period. Substantial reductions in volume of, and sugar in, purchases of SDIL liable drinks were observed in the high and low tiers over time. These reductions were accompanied by a smaller increase in volume of no levy drinks purchased, but proportionally much greater increases in sugar purchased in these drinks.

Primary analysis: category specific results

Results of the controlled interrupted time series analyses of purchased volume of, and sugar in, levy liable drinks and confectionery are shown in Figure 2 (volume) and Figure 3 (sugar). Absolute and relative changes are summarised in Tables 2 and 3. Supplementary tables 2a and b show level and trend changes from these models. Supplementary figures 1a and b show similar figures and data for subcategories of no levy drinks and exempt categories.

High tier drinks

The trend in purchased volume of, and sugar in, high tier drinks continued downwards throughout the study period. The announcement of the SDIL was associated with an increase in purchased volume of (34.7ml (95% confidence intervals 8.1 to 61.4ml, or 7.3% (1.7 to 12.9%)), and sugar in (5.5g (3.8 to 7.2), or 10.8% (7.4 to 14.1%)), these drinks. In contrast, the implementation of the SDIL was associated with a reduction in purchased volume of, and sugar in, these drinks. The volume of high tier drinks purchased was 171.6 mL (135.1 to 208.1mL) per household per week, or 42.5% (33.5% to 51.6%), lower in March 2019 compared with the counterfactual estimated from pre-implementation trends. The reductions associated with implementation outweighed the increases associated with announcement, such that the intervention as a whole was associated with a decrease in purchased volume of 140.8ml (104.3 to 177.3ml) per household per week or 37.8% (28.0 to 47.6%) and sugar of 16.2 g (13.5 to 18.8g) per household per week or 42.6% (35.6% to 49.6%) from these drinks.

Low tier drinks

Purchased volume of, and sugar in, low tier drinks gradually increased before the announcement of SDIL. The announcement was associated with a reversal of this trend.

For peer review only - http://bmjopen.bmj.com/site/about/guidelines.xhtml

There were reductions in purchased volume of, and sugar in, low tier drinks associated with announcement, implementation and the whole intervention. Compared with the counterfactual estimated from pre-announcement trends, in March 2019 the volume of purchased low tier drinks per household per week decreased by 170.5 mL (154.5 to 186.5 mL), or 85.8% (77.8 to 93.9%); and sugar purchased in these drinks decreased by 11.5 g (9.1 to 13.9g) per household per week, or 87.8% (69.2 to 106.4%).

No levy drinks

Before the announcement of the SDIL there was a gradual upward trend in volume of purchased no levy drinks but a gradual downward trend in purchased sugar. Announcement, implementation and the whole intervention were associated with increases in volume of no levy drinks purchased as well as sugar purchased from those drinks. Overall, purchased volume of no levy drinks in March 2019 was 685.5 mL (599.8 to 771.1mL) higher, equivalent to 40.2% (35.2% to 45.2%) increase compared with the counterfactual of pre-announcement trends. Equivalent figures for sugar purchased from no levy drinks were a 19.2g (16.7 to 21.6g) per household per week, equivalent to 242.8% (211.9 to 273.7%), increase.

Changes in purchased volume of subcategories within the no levy drinks group were not uniform and the overall increase in both volume and sugar purchased in this category was driven by low and no sugar drinks, rather than bottled water. The implementation, but not the announcement, of the SDIL were associated with significant decreases in bottled water purchased which led to an overall decrease in volume of bottled water purchased as a result of the whole intervention of 130.5ml (88.8 to 174.1ml) per household per week, or 15.7% (10.4 to 20.9%). In contrast, the implementation and the announcement of the SDIL were associated with increases in volume of purchased drinks with no sugar and with >0 to <5 g total sugar per 100 mL, and increases of sugar in drinks with >0 to<5g sugar per 100ml.

Levy exempt drinks and confectionery

Overall, the combined announcement and implementation of the SDIL were associated with decreases in purchased volume of alcoholic and milk and milk-based drinks, but no change in sugar purchased from levy exempt categories or from confectionery. Compared with the counterfactual of pre-announcement trends, in March 2019 volume of alcoholic drinks purchased decreased by 103.1ml (53.0 to 153.3ml) per household per week, equivalent to a 5.8% (3.0 to 8.6%) reduction; and volume of milk and milk based drinks purchased decreased by 132.8ml (51.7 to 213.9ml), equivalent to a 3.6% (1.4 to 5.7%) reduction.

For peer review only - http://bmjopen.bmj.com/site/about/guidelines.xhtml

Secondary analysis: all soft drinks categories combined

Supplementary table 3a and Supplementary figure 2 summarise the results of the controlled interrupted time series analyses of the associated effects of the SDIL on purchased volume of, and sugar from, all soft drinks categories combined, irrespective of levy eligibility. Supplementary table 3b summarises absolute and relative changes in volume of, and sugar in, all soft drinks and confectionery purchased.

Overall, compared with the counterfactual estimated from pre-announcement trends, a small increase was observed in volume of all soft drinks purchased in March 2019 of 188.8ml (30.7 to 346.9ml) per household per week, equivalent to a 2.6% (0.4 to 4.7%) increase. A reduction was, however, found in sugar purchased in all soft drinks (including exempt drinks) combined of 8.0g per household per week (2.4 to 13.6g), equivalent to 2.7% (0.8 to 4.5%).

Sensitivity analyses

Excluding manufacturers of levy liable products with less than one million and less than 500 000 litres of purchased drinks annually in our dataset was associated with small changes in the magnitude of estimated coefficients, but with no change in the direction or statistical significance of any findings (Supplementary tables 4a to b).

In general, removing the control category led to minor changes in effect estimates but wider confidence intervals (see Supplementary material G).

Discussion

Taking account of pre-existing pre-announcement trends, this study found that one year after implementation of the SDIL, sugar purchased from soft drinks that were taken home decreased by 8.0 g per household per week (or 2.7%), whilst volume increased by 188.8 mL per household per week (or 2.6%). Assuming a mean UK household size of 2.4 people,[34] this is equivalent to a reduction in sugar from SSBs of 3.3 g per person per week and an increase in volume of 79 mL per person per week, or equivalent to the replacement of 66 mL of a drink with 5 g sugar per 100 mL per person per week with 145 mL of a sugar-free alternative. A modelling study conducted before implementation of the SDIL found that if the levy achieved reformulation it could be expected to lead to a decrease in sugar consumption from SSBs (from all sources, not just for consumption at home) of 7-38 g per person per week and that this would be associated with a reduction in the number of obese individuals in the UK of 0.2-0.9% and a reduction in incidence cases of type 2 diabetes of -2.0 to 31.1 per 1000 person years.[35] The reduction in sugar from SSBs we report one year after implementation of the SDIL is around half of these lower effect estimates.

For peer review only - http://bmjopen.bmj.com/site/about/guidelines.xhtml

Strengths and weaknesses of this study

In this study we used a large, nationally representative dataset, included a control category, and explored changes in two potential substitute categories (alcohol and confectionery).

We only included purchases brought into homes. Although KWP also collects data on other purchases, this smaller panel was established in mid-2015 and so was unsuitable for our analyses because robust pre-announcement trends could not be estimated. KWP data are collected at the household level and do not take account of waste or differential sharing within households. Nevertheless, the data provide a reasonable estimate of consumption.[36] We did not assess changes in other categories beyond soft drinks, alcohol, and confectionery.

The estimate of effect size in interrupted time series analyses is based on a modelled counterfactual that might be inaccurate. For example, the strong downward trend in higher tier drinks before the announcement of SDIL might not have continued. Attribution of effects in interrupted time series analyses is vulnerable to time varying confounding including co-interventions. The SDIL is part of a wider sugar reduction strategy, although this has been found to have achieved minimal changes beyond those attributable to the SDIL.[25]

The personal characteristics of the panel remained similar over the study period, and proprietary weightings were used to account for non-consumers and to adjust for variations in panel composition. Households participating in KWP are slightly more likely to be from lower social grades and to have no qualifications compared with UK households generally. This might reflect the relative value placed on the small rewards for participation by different households and could limit the generalisability of our findings. If households from lower socioeconomic backgrounds are more likely to change purchasing as a result of the SDIL, then we could have marginally overestimated the effect of the SDIL. However, while we previously found that the price of soft drinks in the UK did change after implementation of the SDIL, no clear pattern was found, with the price of some groups of drinks increasing and others decreasing.[15] We previously found no systematic differences between the sugar content of drinks reported in KWP data and contemporaneous values listed on supermarket websites.[27]

Comparison with other work

Our finding that the SDIL was associated with a reduction in purchased sugar from all soft drinks is consistent with previous analyses that focused on the SDIL.[24 25] Although our estimate of the reduction in sugar consumption from all soft drinks associated with the levy (2.7%) is less than that estimated by others (29%)[25] this previous estimate did not take

For peer review only

For peer review only - http://bmjopen.bmj.com/site/about/guidelines.xhtml

account of pre-existing trends which we have demonstrated were on a steep downward trajectory for high tier drinks.

We found that the reduction in purchased sugar from all soft drinks alongside a 2.6% increase in volume of all soft drinks purchased. This is consistent with previously reported reductions in the sugar concentration of drinks associated with the SDIL.[15] However, the estimated effect size is below the range of reformulation scenarios modelled before implementation (ie, a reduction of 17 to 90 g of sugar per household per week).[35] This difference may be, at least partly, attributable to our focus on drinks taken home versus the modelling study's focus on all drinks. Furthermore, the modelling was based on pre-implementation best and worst case scenarios of changes in formulation, price and SSB market share whilst our analysis was based on observed data.

Evaluations of other SSB taxes have revealed a consistent trend of reductions in purchasing of taxed drinks and no change in purchasing of untaxed drinks.[5] We found similar with both volume of, and sugar in, high and low tier drinks decreasing overall. However, these reductions in volume of taxed drinks were more than offset by increases in volume of no levy drinks purchased. Despite some increases in sugar purchased in no levy drinks, these did not offset decreases in sugar purchased from high and low tier drinks. The SDIL is relatively unique in being explicitly designed to encourage reformulation and there is evidence that substantial reformulation occurred.[15] We are not able to determine from our findings whether the changes we report are due to changes in consumer preference, formulation, or both.

Meaning of the study and implications for policymakers

Our main findings are that the SDIL was associated with a reduction in purchased sugar from all soft drinks with evidence of an increase in the total volume of soft drinks purchased. Given the reformulation associated with the SDIL already documented,[15] it is probable that the changes we report were driven by reductions in the sugar concentration of available drinks, alongside consumers switching to and, indeed increasing consumption of, lower sugar alternatives. Despite the overall reduction we found in sugar purchased in soft drinks, the average amount of sugar purchased in drinks in the no levy group paradoxically increased after implementation of the SDIL, with many drinks that previously had sugar concentrations above the levy threshold now having them just below the threshold. This seems to reflect manufacturers reformulating to target thresholds. Lowering the threshold sugar concentration at which drinks become eligible for the SDIL even further could potentially lead to greater

For peer review only - http://bmjopen.bmj.com/site/about/guidelines.xhtml

overall reductions in sugar concentrations and sugar purchased in soft drinks, as could extension of the SDIL to milk based drinks and other currently exempt categories that sometimes contain high levels of sugar.

Nevertheless, the overall reduction in sugar with an increase in volume we report here might represent a valuable benefit for public health with potential associated benefit to the food industry. The SDIL has also been found to have had no long term negative effects on the share value or turnover of UK soft drinks companies,[37 38] suggesting that, contrary to industry predictions, public health can gain without negatively affecting the soft drinks sector.

We note a marked pre-implementation decline in purchasing of high levy tier drinks. It is possible that this was, at least in part, driven by concern from industry about a possible SSB tax, leading to some pre-announcement reformulation; alongside growing consumer awareness of, and concerns about, the health impacts of SSBs.[39] Although it is uncertain if this trend would have continued in the absence of the SDIL, it is likely to be beneficial for health.

Reassuringly, we did not observe any increase in purchasing of potentially harmful substitutes (ie, alcohol and confectionery) associated with the SDIL, which could have partially or wholly offset any public health gains from the SDIL. However, we did not study the SDIL's effect on purchases of other food groups or on overall diet.

In contrast with previous findings from Mexico and Barbados,[6 40] we did not observe an increase in purchased bottled water associated with the SDIL. Indeed purchases of bottled water decreased significantly during the study period (by 130.5 mL per household per week, or 15.7%). Although we cannot rule out an effect of the SDIL on bottled water purchases, we cannot think of a plausible pathway through which it achieved reductions in purchased bottled water. Instead, this reduction might be due to coincident increases in concern about single use plastic that have been attributed, in the UK, to the broadcast of the nature documentary series *Blue Planet 2* in October-December 2017.[41] It is not clear if a similar "Blue Planet effect" has occurred in other countries. Unlike for many other soft drinks, a like-for-like substitution is available for bottled water in countries such as the UK—that is, filling reusable water bottles with tap water. Several UK retailers have reported substantial growth in sales of reusable water bottles since 2018.[42] Given that tap water is freely available, it is difficult to study changes in its consumption directly.

Unanswered questions and future research

For peer review only - http://bmjopen.bmj.com/site/about/guidelines.xhtml
For peer review only

Future work should seek to understand the longer term effects of the SDIL on purchasing and consumption of soft drinks as well as total diet, and health outcomes. Differential effects of the SDIL on all these outcomes across population groups (eg, by socioeconomic position and in households with vs without children) should also be explored to determine whether the SDIL contributes to narrowing inequalities in health. The changes in purchasing we report here could be used as an input to health impact modelling to estimate the effect of changes on population prevalence of obesity, diabetes, and other chronic conditions to determine how apparently small changes in consumption at the household level translate into health benefits. It is likely that the reformulation that has occurred in response to the SDIL[15] reflects substantial increases in the use of artificial sweeteners in the UK soft drinks market. Given public mistrust of artificial sweeteners[39] and the recent advice from WHO that artificial sweeteners should not be used to reduce the risk of non-communicable diseases,[43] the effect of the SDIL on consumption of these should also be explored.

Conclusion

One year after implementation of the SDIL, purchased sugar in soft drinks decreased by around 8 g per household per week (or 2.7%) with an increase in the volume of purchased soft drinks of 189 mL per household per week (or 2.6%). This tiered tax aiming to stimulate industry to remove sugar from soft drinks might represent a benefit for public health (by reducing sugar purchased from soft drinks without substitution to confectionery and alcohol) and also to the soft drinks industry (by total volume of soft drinks purchased). Further studies are required to determine whether and how these apparently small effect sizes translate into health outcomes.

For peer review only - http://bmjopen.bmj.com/site/about/guidelines.xhtml

Contributors

DP, MW, SC, MR, RS, HR, JA, PS, OM, AB, and TLP conceived the study and defined the analytical strategy. DP, NR, JA, OM, TLP, and SS performed statistical analyses and provided preliminary interpretation of findings. DP, NR, JA, OM, and TLP drafted the manuscript. All authors critically interpreted the results, revised the manuscript, provided relevant intellectual input, and read and approved the final manuscript. NR and JA had primary responsibility for the final content. JA will act as guarantor. The corresponding author attests that all listed authors meet authorship criteria and that no others meeting the criteria have been omitted.

Funding

NR, OM, MW, and JA were funded by the Centre for Diet and Activity Research (CEDAR), a UK Clinical Research Collaboration (UKCRC) Public Health Research Centre of Excellence Funding from the British Heart Foundation, Cancer Research UK, Economic and Social Research Council, Medical Research Council, National Institute for Health Research (NIHR), and Wellcome Trust, under the auspices of the UKCRC. This project was funded by the NIHR Public Health Research programme (grant Nos 16/49/01 and 16/130/01), and these grants supported purchase of the data used in this study. The work was also supported by the Medical Research Council (grant Nos MC_UU_12015/6 and MC_UU_00006/7). The views expressed are those of the authors and not necessarily those of the National Health Service, the NIHR, or the Department of Health and Social Care, UK. The funders had no role in study design, data collection and analysis, decision to publish, or preparation of the manuscript. NR and JA had full access to all the data in the study. All authors had final responsibility for the decision to submit for publication. For the purpose of Open Access, the author has applied a Creative Commons Attribution (CC BY) licence to any Author Accepted Manuscript version arising.

Competing interests

All authors have completed the ICMJE uniform disclosure form at www.icmje.org/coi_disclosure.pdf (available on request from the corresponding author) and declare: MW was director of the National Institute for Health Research Public Health Research Funding programme when this work was conducted, and OM was on secondment at the UK Department of Health and Social Care when this work was conducted and previously worked with Public Health England; no support from any organisation for the submitted work other than that described above; no financial relationships with any organisations that might have an interest in the submitted work in the previous three years; and no other relationships or activities that could appear to have influenced the submitted work.

Ethical approval

Not required for secondary data analysis of anonymised data.

Data availability statement

The statistical code for the analyses are available from https://github.com/MRC-Epid/SDILEvaluation. Kantar Worldpanel data are not publicly available but can be purchased from Kantar Worldpanel (http://www.kantarworldpanel.com). The authors are not legally permitted to share the data used for this study but interested parties can contact Kantar WorldPanel representative Sean Cannon (Sean.Cannon@kantar.com) to inquire about accessing this proprietary data.

The lead author affirms that the manuscript is an honest, accurate, and transparent account of the study being reported; that no important aspects of the study have been omitted;

For peer review only

For peer review only - http://bmjopen.bmj.com/site/about/guidelines.xhtml

and that any discrepancies from the study as planned (and, if relevant, registered) have been explained.

For peer review only

For peer review only - http://bmjopen.bmj.com/site/about/guidelines.xhtml

References

1. Imamura F, O'Connor L, Ye Z, et al. Consumption of sugar sweetened beverages, artificially sweetened beverages, and fruit juice and incidence of type 2 diabetes: Systematic review, meta-analysis, and estimation of population attributable fraction. BMJ 2015;**351** doi: 10.1136/bmj.h3576[published Online First: Epub Date]|.

2. Moynihan PJ, Kelly SAM. Effect on caries of restricting sugars intake: Systematic review to inform WHO guidelines. Journal of Dental Research 2014;**93**(1):8-18 doi: 10.1177/0022034513508954[published Online First: Epub Date]|.

3. Xi B, Huang Y, Reilly KH, et al. Sugar-sweetened beverages and risk of hypertension and CVD: A dose-response meta-analysis. British Journal of Nutrition 2015;**113**(5):709-17 doi: 10.1017/S0007114514004383[published Online First: Epub Date]|.

4. World Health Organization. Best buys' and Other Recommended Interventions for the Prevention and Control of Noncommunicable Diseases. Geneva: WHO, 2017.

5. Teng AM, Jones AC, Mizdrak A, Signal L, Genç M, Wilson N. Impact of sugar-sweetened beverage taxes on purchases and dietary intake: Systematic review and meta-analysis. Obesity Reviews 2019;**20**(9):1187-204 doi: 10.1111/obr.12868[published Online First: Epub Date]|.

6. Alvarado M, Unwin N, Sharp SJ, et al. Assessing the impact of the Barbados sugar-sweetened beverage tax on beverage sales: An observational study. International Journal of Behavioral Nutrition and Physical Activity 2019;**16**(1) doi: 10.1186/s12966-019-0776-7[published Online First: Epub Date]|.

7. Cawley J, Frisvold D, Hill A, Jones D. The impact of the Philadelphia beverage tax on purchases and consumption by adults and children. Journal of Health Economics 2019;**67** doi: 10.1016/j.jhealeco.2019.102225[published Online First: Epub Date]|.

8. Lee MM, Falbe J, Schillinger D, Basu S, McCulloch CE, Madsen KA. Sugar-sweetened beverage consumption 3 years after the Berkeley, California, sugar-sweetened beverage tax. American Journal of Public Health 2019;**109**(4):637-39 doi: 10.2105/AJPH.2019.304971[published Online First: Epub Date]|.

9. Roberto CA, Lawman HG, Levasseur MT, et al. Association of a Beverage Tax on Sugar-Sweetened and Artificially Sweetened Beverages with Changes in Beverage Prices and Sales at Chain Retailers in a Large Urban Setting. JAMA - Journal of the American Medical Association 2019;**321**(18):1799-810 doi: 10.1001/jama.2019.4249[published Online First: Epub Date]|.

10. Royo-Bordonada MA, Fernández-Escobar C, Simón L, Sanz-Barbero B, Padilla J. Impact of an excise tax on the consumption of sugar-sweetened beverages in young people living in poorer neighbourhoods of Catalonia, Spain: A difference in differences study. BMC Public Health 2019;**19**(1) doi: 10.1186/s12889-019-7908-5[published Online First: Epub Date]|.

11. Alvarado M, Kostova D, Suhrcke M, et al. Trends in beverage prices following the introduction of a tax on sugar-sweetened beverages in Barbados. Preventive Medicine 2017;**105**:S23-S25 doi: 10.1016/j.ypmed.2017.07.013[published Online First: Epub Date]|.

For peer review only - http://bmjopen.bmj.com/site/about/guidelines.xhtml

12. Cawley J, Willage B, Frisvold D. Pass-through of a taxon sugar-sweetened beverages at the philadelphia international airport. JAMA - Journal of the American Medical Association 2018;**319**(3):305-06 doi: 10.1001/jama.2017.16903[published Online First: Epub Date]|.

13. Falbe J, Rojas N, Grummon AH, Madsen KA. Higher retail prices of sugar-sweetened beverages 3 months after implementation of an excise tax in Berkeley, California. American Journal of Public Health 2015;**105**(11):2194-201 doi: 10.2105/AJPH.2015.302881[published Online First: Epub Date]|.

14. Mytton OT, Eyles H, Ogilvie D. Evaluating the health impacts of food and beverage taxes. Curr Obes Rep 2014;**3**(4):432-39

15. Scarborough P, Adhikari V, Harrington RA, et al. Impact of the announcement and implementation of the UK soft drinks industry levy on sugar content, price, product size and number of available soft drinks in the UK, 2015-19: A controlled interrupted time series analysis. PLoS Medicine 2020;**17**(2) doi: 10.1371/JOURNAL.PMED.1003025[published Online First: Epub Date]|.

16. Stacey N, Mudara C, Ng SW, van Walbeek C, Hofman K, Edoka I. Sugar-based beverage taxes and beverage prices: Evidence from South Africa's Health Promotion Levy. Social Science and Medicine 2019;**238** doi: 10.1016/j.socscimed.2019.112465[published Online First: Epub Date]|.

17. Penney T, Adams J, Briggs A, et al. Theorising how the UK Soft Drinks Industry Levy could impact population level diet and health: Development of a multi-sectoral systems map. International Society for Behavioural Nutrition and Physical Activity. Victoria, Canada, 2017.

18. Quirmbach DD, Cornelsen L, Jebb SA, Marteau T, Smith R. Effect of increasing the price of sugar-sweetened beverages on alcoholic beverage purchases: an economic analysis of sales data. Journal of epidemiology and community health 2018;**72**(4):324-30 doi: 10.1136/jech-2017-209791[published Online First: Epub Date]|.

19. Scheelbeek PFD, Cornelsen L, Marteau TM, Jebb SA, Smith RD. Potential impact on prevalence of obesity in the UK of a 20% price increase in high sugar snacks: Modelling study. BMJ 2019;**366** doi: 10.1136/bmj.l4786[published Online First: Epub Date]|.

20. Smith RD, Cornelsen L, Quirmbach D, Jebb SA, Marteau TM. Are sweet snacks more sensitive to price increases than sugar-sweetened beverages: Analysis of British food purchase data. BMJ Open 2018;**8**(4) doi: 10.1136/bmjopen-2017-019788[published Online First: Epub Date]|.

21. HM Revenue & Customs. Soft Drinks Industry Levy. Secondary Soft Drinks Industry Levy 2016. https://www.gov.uk/government/publications/soft-drinks-industry-levy/soft-drinks-industry-levy.

22. Gauke D. Budget 2016. London: HM Treasury, 2016.

23. HM Revenue & Customs. Check if your drink is liable for the Soft Drinks Industry Levy. Secondary Check if your drink is liable for the Soft Drinks Industry Levy 2018. https://www.gov.uk/guidance/check-if-your-drink-is-liable-for-the-soft-drinks-industry-levy.

For peer review only - http://bmjopen.bmj.com/site/about/guidelines.xhtml

24. Bandy LK, Scarborough P, Harrington RA, Rayner M, Jebb SA. Reductions in sugar sales from soft drinks in the UK from 2015 to 2018. BMC Medicine 2020;**18**(1) doi: 10.1186/s12916-019-1477-4[published Online First: Epub Date]|.

25. Public Health England. Sugar Reduction: Report on Progress between 2015 and 2018. London: Public Health England, 2019.

26. British Soft Drinks Association. 2018 Annual Report. London: British Soft Drinks Association, 2019.

27. Pell D, Penney TL, Mytton O, et al. Anticipatory changes in British household purchases of soft drinks associated with the announcement of the Soft Drinks Industry Levy: A controlled interrupted time series analysis. PLoS Medicine 2020;**17**(11) doi: 10.1371/journal.pmed.1003269[published Online First: Epub Date]|.

28. White M, Cummins S, Rayner M, et al. Evaluation of the health impacts of the UK Treasury Soft Drinks Industry Levy (SDIL). NIHR Public Health Research Programme 2017

29. Webber D, O'Neill J. Average Household Income, UK: Financial Year Ending 2019. Secondary Average Household Income, UK: Financial Year Ending 2019. 2020. https://www.ons.gov.uk/peoplepopulationandcommunity/personalandhouseholdfinances/incomeandwealth/bulletins/householddisposableincomeandinequality/financialyearending2019provisional#:~:text=The%20distribution%20of%20people's%20household,year%20ending%20(FYE)%202019.

30. Lopez Bernal J, Cummins S, Gasparrini A. The use of controls in interrupted time series studies of public health interventions. International Journal of Epidemiology 2018;**47**(6):2082-93 doi: 10.1093/ije/dyy135[published Online First: Epub Date]|.

31. Zhang F, Wagner AK, Soumerai SB, Ross-Degnan D. Methods for estimating confidence intervals in interrupted time series analyses of health interventions. Journal of Clinical Epidemiology 2009;**62**(2):143-48 doi: 10.1016/j.jclinepi.2008.08.007[published Online First: Epub Date]|.

32. Pell D, Mytton O, Penney TL, et al. Changes in soft drinks purchased by British households associated with the UK soft drinks industry levy: controlled interrupted time series analysis. BMJ 2021;**372**:n254 doi: 10.1136/bmj.n254[published Online First: Epub Date]|.

33. Adams J. Rapid response to: Changes in soft drinks purchased by British households associated with the UK soft drinks industry levy: controlled interrupted time series analysis. Secondary Rapid response to: Changes in soft drinks purchased by British households associated with the UK soft drinks industry levy: controlled interrupted time series analysis 2022. https://www.bmj.com/content/372/bmj.n254/rr-0.

34. Knipe E. Families and Households in the UK: 2017. Secondary Families and Households in the UK: 2017. 2017. https://www.ons.gov.uk/peoplepopulationandcommunity/birthsdeathsandmarriages/families/bulletins/familiesandhouseholds/2017.

35. Briggs ADM, Mytton OT, Kehlbacher A, et al. Health impact assessment of the UK soft drinks industry levy: a comparative risk assessment modelling study. The Lancet Public

For peer review only - http://bmjopen.bmj.com/site/about/guidelines.xhtml

Health 2017;**2**(1):e15-e22 doi: 10.1016/S2468-2667(16)30037-8[published Online First: Epub Date]|.

36. Griffith R, O'Connell M, Smith K. Measuring the quality of people's diets: a comparison of intake and purchase data. Econometrics and IO of food and nutrition. Toulouse School of Economics 2012.

37. Law C, Cornelsen L, Adams J, et al. The impact of UK soft drinks industry levy on manufacturers' domestic turnover. Economics and Human Biology 2020;**37** doi: 10.1016/j.ehb.2020.100866[published Online First: Epub Date]|.

38. Law C, Cornelsen L, Adams J, et al. An analysis of the stock market reaction to the announcements of the UK Soft Drinks Industry Levy. Economics and Human Biology 2020;**38** doi: 10.1016/j.ehb.2019.100834[published Online First: Epub Date]|.

39. Pell D, Penney T, Hammond D, Vanderlee L, White M, Adams J. Support for, and perceived effectiveness of, the UK soft drinks industry levy among UK adults: Cross-sectional analysis of the International Food Policy Study. BMJ Open 2019;**9**(3) doi: 10.1136/bmjopen-2018-026698[published Online First: Epub Date]|.

40. Colchero MA, Popkin BM, Rivera JA, Ng SW. Beverage purchases from stores in Mexico under the excise tax on sugar sweetened beverages: Observational study. BMJ 2016;**352** doi: 10.1136/bmj.h6704[published Online First: Epub Date]|.

41. Males J, Van Aelst P. Did the Blue Planet set the Agenda for Plastic Pollution? An Explorative Study on the Influence of a Documentary on the Public, Media and Political Agendas. Environmental Communication 2020:1-15 doi: 10.1080/17524032.2020.1780458[published Online First: Epub Date]|.

42. O'Mahony A. The rise and fall: Bottled water category report 2020. *The Grocer* 2020.

43. World Health Organization. Use of non-sugar sweeteners: WHO guideline. Geneva: World Health Organization; 2023. Geneva: World Health Organization, 2023.

For peer review only

For peer review only - http://bmjopen.bmj.com/site/about/guidelines.xhtml

Figure legends

Figure 1. Schematic of overall analysis strategy. Solid lines=observed data; dashed lines=counterfactual estimated from previous observed data.

Figure 2. Observed and modelled volume (mls) of drinks liable to the Soft Drinks Industry Levy (SDIL), and weight of confectionery (g) purchased per household per week, March 2014 to March 2019 (weighted). Points are observed data for drinks/ confectionery; black lines (with shadows) show modelled data (and 95% confidence intervals); red lines indicate the counterfactuals had the announcement (red solid line) and implementation (red dashed line) not happened; the first dashed vertical line indicates the announcement of SDIL; the second dashed vertical line indicates the implementation of SDIL; the Y axis varies in scale between panels to maximise the resolution of figures; modelled purchases include averaged effects for seasonality and the impact of December and January (Christmas period), and, for confectionery, Easter; the control category of toiletries is shown in Figure 3.

Figure 3. Observed and modelled amount of sugar (g) in drinks liable to the Soft Drinks Industry Levy and confectionery purchased per household per week, March 2014 to March 2019 (weighted). Points are observed data for drinks/ confectionery and toiletries; black lines (with shadows) show modelled data (and 95% confidence intervals); red lines indicate the counterfactuals had the announcement (red solid line) and implementation (red dashed line) not happened; the first dashed vertical line indicates the announcement of SDIL; the second dashed vertical line indicates the implementation of SDIL; the Y axis varies in scale between panels to maximise the resolution of figures; modelled purchases include averaged effects for seasonality and the impact of December and January (Christmas period), and, for confectionery, Easter.

For peer review only

For peer review only - http://bmjopen.bmj.com/site/about/guidelines.xhtml

Table 1. Mean volume of, and amount of sugar in, purchased drinks and confectionery per household per week in relation to the UK soft drinks industry levy, March 2014 to March 2019 (weighted)

	Mean (SD) volume (mL) per household/week			Mean (SD) amount of sugar (g) per household/week		
	**Pre-announcement: Mar 2014-Mar 2016**	**Post-announcement: Mar 2016-Mar 2018**	**Post-implementation: Apr 2018-Mar 2019**	**Pre-announcement: Mar 2014-Mar 2016**	**Post-announcement: Mar 2016-Mar 2018**	**Post-implementation: Apr 2018-Mar 2019**
**All drinks**	7595 (295)	7547 (466)	7826 (540)	364 (17)	337(24)	307(19)
**Levy liable drinks (sugar/100 mL)**						
High tier (≥8 g)	880 (128)	680 (136)	363 (76)	98(14)	76(15)	40(9)
Low tier (≥5 g to <8 g	155 (32)	147 (37)	75 (32)	10(2)	10(2)	5(2)
No levy (<5 g):	1811 (169)	1876 (216)	2448 (321)	12(2)	12(3)	25(5)
>0 g to <5 g	785 (78)	989 (139)	768 (92)	12(2)	12(3)	25(5)
0 g	1027 (104)	1108 (132)	1459 (190)	0 (0)	0 (0)	0 (0)
Bottled water	591(72)	714(90)	786 (138)	0 (0)	0 (0)	0 (0)
**Levy exempt drinks**						
Alcoholic drinks	1874 (380)	1872 (456)	1948 (467)	.	.	.
Milk and milk based drinks:	3546 (137)	3540 (155)	3542 (148)	172 (7)	172(8)	170(7)
Fruit juices with no added sugar	516.6(29)	502(44)	520(47)	51(3)	49(4)	50(5)
Drinks sold as powders (g)	95(12)	88(11)	90(11)	21(3)	19(3)	18(3)
Confectionery (g)	308 (91)	303 (92)	318 (100)	173 (51)	170 (52)	178 (57)
Toiletries	123 (8)	120 (8)	121 (9)	.	.	.

Sugar from alcoholic drinks is not included here as many alcoholic drinks contain sugar but the product label does not provide the amount.

For peer review only - http://bmjopen.bmj.com/site/about/guidelines.xhtml

Table 2. Absolute and relative change in volume of drinks (mL) and confectionery (g) purchased per household per week in relation to the UK soft drinks industry Levy, March 2014 to March 2019 (weighted)

	**Analysis 1: pre-post announcement (Mar 2014-Mar 2018)**		**Analysis 2: pre-post implementation (Mar 2016-Mar 2019)**		**Analysis 3: pre-post announcement and implementation (Mar 2014-Mar 2019)**	
	**Absolute change (mL or g)**	**Relative change (%)**	**Absolute change (mL or g)**	**Relative change (%)**	**Absolute change (mL or g)**	**Relative change (%)**
**All drinks**	**11.8 (-103.7, 127.3)**	0.16 (-1.42, 1.74)	**187.8 (29.7, 345.9)**	**2.56 (0.40, 4.71)**	**188.8 (30.7, 346.9)**	**2.6 (0.4, 4.7)**
**Levy liable drinks (sugar/100 mL)**						
High tier (≥8 g)	**34.7 (8.06, 61.4)**	**7.27 (1.69, 12.9)**	**-171.6 (-208.1,-135.1)**	**-42.5 (-51.6, -33.5)**	**-140.8 (-177.3, -104.3)**	**-37.8 (-47.6, -28.0)**
Low tier (≥5 g to <8 g)*	**-65.7 (-77.5, -53.8)**	**-37.1 (-43.7, -30.4)**	**-71.8 (-87.8, -55.8)**	**-71.8 (-87.8, -55.8)**	**-170.5 (-186.5, -154.5)**	**-85.8 (-93.9, -77.8)**
No levy (<5 g):	**181.0 (118.4, 243.5)**	**11.1 (7.26, 14.9)**	**395.0 (309.4. 480.7)**	**19.8 (15.5, 24.1)**	**685.5 (599.8, 771.1)**	**40.2 (35.2, 45.2)**
>0 g to <5 g	**103.8 (75.2, 132.5)**	**16.7 (12.1, 21.3)**	**202.0 (162.7, 241.2)**	**25.0 (20.1, 29.9)**	**374.6 (335.4, 413.9)**	**59.0 (52.8, 65.1)**
0 g	**87.8 (41.1, 134.5)**	**8.66 (4.05, 13.3)**	**178.9 (115.6, 242.3)**	**14.7 (9.52, 20.0)**	**316.1 (252.7, 379.4)**	**29.4 (23.5, 35.3)**
Bottled water	30.3 (-62.0, 1.4)	4.24 (-8.7, 0.2)	**82.1 (-125.7, -38.4)**	**-10.5 (-16.1, -4.9)**	**-130.5 (-174.1, -88.8)**	**-15.7 (-20.9, -10.4)**
**Levy exempt drinks**						
Alcoholic drinks	-16.5 (-48.5, 15.4)	0.95 (-2.79, 0.89)	**-84.9 (-135.1, -34.7)**	**-4.81 (-7.66, -1.97)**	**-103.1 (-153.3, -53.0)**	**-5.8 (-8.60, -2.97)**
Milk and milk based drinks	**-185.5 (-249.7, -121.4)**	**-4.9 (-6.60, -3.20)**	**145.5 (64.4, 226.6)**	**4.21 (1.86, 6.56)**	**-132.8 (-213.9, -51.7)**	**-3.56 (-5.73, -1.38)**
No added sugar fruit juices	6.8 (-6.9, 20.5)	1.4 (-1.4, 4.3)	-6.2 (-24.8, 12.5)	-1.26 (-6.1, 2.5)	8.7 (-9.9, 27.3)	1.82 (-2.1, 5.7)
Drinks sold as powders (g)	**-6.9 (-10.0, -3.8)**	**-6.8 (-9.9, -3.8)**	**9.6 (5.3, 13.9)**	**11.2 (6.2, 16.2)**	0.9 (-3.3, 5.2)	1.0 (-3.5, 5.5)
Confectionery (g)	-10.1 (-53.9, 33.8)	-2.4 (-13.1, 8.2)	39.8 (-19.0, 98.6)	11.6 (-5.5, 28.8)	35.3 (94.1, -23.5)	10.2 (-6.8, 27.1)

Bold indicates significant difference at 95% confidence interval level.

For peer review only

For peer review only - http://bmjopen.bmj.com/site/about/guidelines.xhtml

Table 3. Absolute and relative change in sugar in drinks and confectionery (g) purchased per household (95% CI) per week in relation to the UK SDIL, March 2014- March 2019 (weighted)

	Analysis 1: pre-post announcement (Mar 2014-Mar 2018)		Analysis 2: pre-post implementation (Mar 2016-Mar 2019)		Analysis 3: pre-post announcement and implementation (Mar 2014-Mar 2019)	
	Absolute change (g)	Relative change (%)	Absolute change (g)	Relative change (%)	Absolute change (g)	Relative change (%)
**All drinks**	**4.6 (0.5, 8.6)**	**1.4 (0.2, 2.7)**	**-12.9 (-18.5, -7.4)**	**-4.3 (-6.1, -2.4)**	**-8.0 (-13.6, -2.4)**	**-2.7 (-4.5, -0.8)**
**Levy liable drinks (sugar/100 mL)**						
High tier (≥8 g)	**5.5 (3.8, 7.2)**	**10.8 (7.4, 14.1)**	**-21.2 (-23.8, -18.5)**	**-49.3 (-55.4, -43.1)**	**-16.2 (-18.8, -13.5)**	**-42.6 (-49.6, -35.6)**
Low tier (≥5 g to <8 g)	**-4.3 (-6.1, -2.6)**	**-37.5 (-52.5, -22.5)**	**-5.0 (-7.4, -2.6)**	**-75.8 (-112.7, -38.9)**	**-11.5 (-13.9, -9.1)**	**-87.8 (-106.4, -69.2)**
No levy (<5 g)†	**5.7 (3.9, 7.4)**	**72.6 (50.3, 94.9)**	**9.7 (7.3,12.1)**	**56.0 (41.9, 70.0)**	**19.2 (16.7, 21.6)**	**242.8 (211.9, 273.7)**
>0 g to <5 g sugar per 100 mL†	**5.7 (3.9, 7.4)**	**72.6 (50.3, 94.9)**	**9.7 (7.3,12.1)**	**56.0 (41.9, 70.0)**	**19.2 (16.7, 21.6)**	**242.8 (211.9, 273.7)**
**Levy exempt drinks**						
Milk and milk based drinks	**-3.9 (-6.5, -1.3)**	**-2.2 (-3.6, -0.7)**	**4.1 (0.5, 7.7)**	**2.4 (0.3, 4.6)**	-3.1 (-6.7, 0.5)	-1.8 (-3.8, 0.3)
No added sugar fruit juices	**2.6 (0.3, 4.8)**	**5.7 (0.7, 10.7)**	-1.7 (-4.8, 1.5)	-3.5 (-10.0, 3.0)	2.6 (-0.5, 5.7)	5.9 (-1.2, 13.1)
Drinks sold as powders (g)	0.3 (-1.6, 2.2)	1.6 (-7.5, 10.6)	-0.04 (-2.7, 2.6)	-0.2 (-13.9, 13.5)	1.1 (-1.6, 3.7)	5.7 (-8.8, 20.2)
Confectionery (g)	-6.6 (-32.0, 18.9)	-2.8 (-13.8, 8.14)	22.1 (-12.0, 56.1)	11.4 (-6.2, 29.1)	18.4 (-15.7, 52.4)	9.3 (-8.0, 26.7)

The counterfactual for low tier drinks crossed 0 mL shortly before the end of the study period thus predicting negative purchases; therefore, the non-counterfactual estimate at the end of the study period was compared with the final week during which the counterfactual was a positive number.
*Significant difference at 95% confidence interval level.

For peer review only - http://bmjopen.bmj.com/site/about/guidelines.xhtml
For peer review only

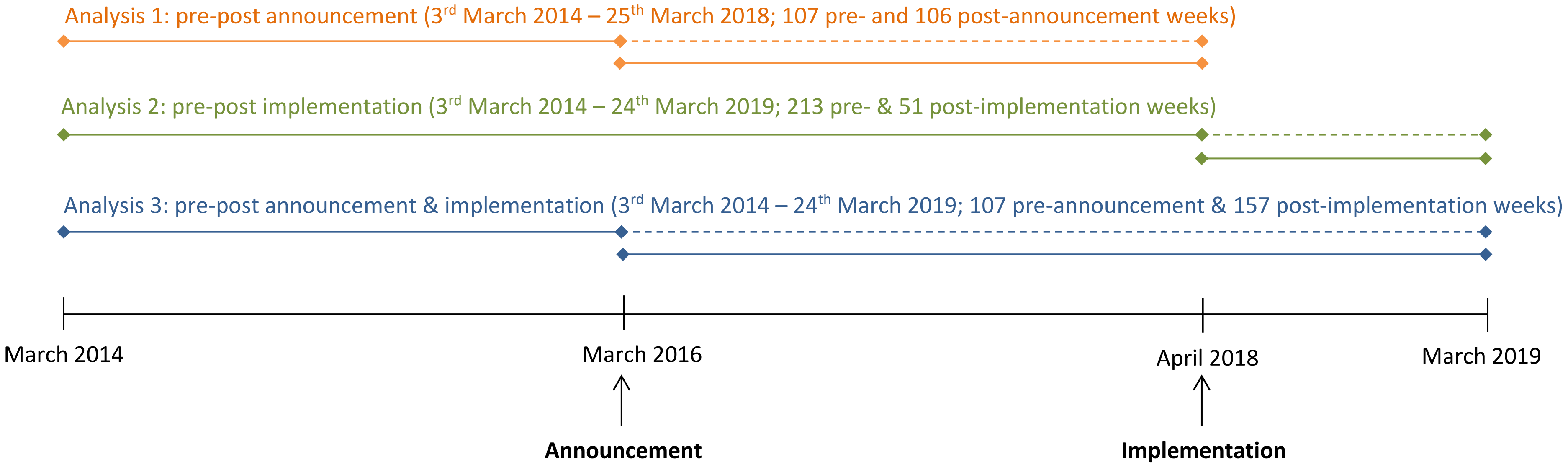

Figure 1: Schematic of overall analysis strategy

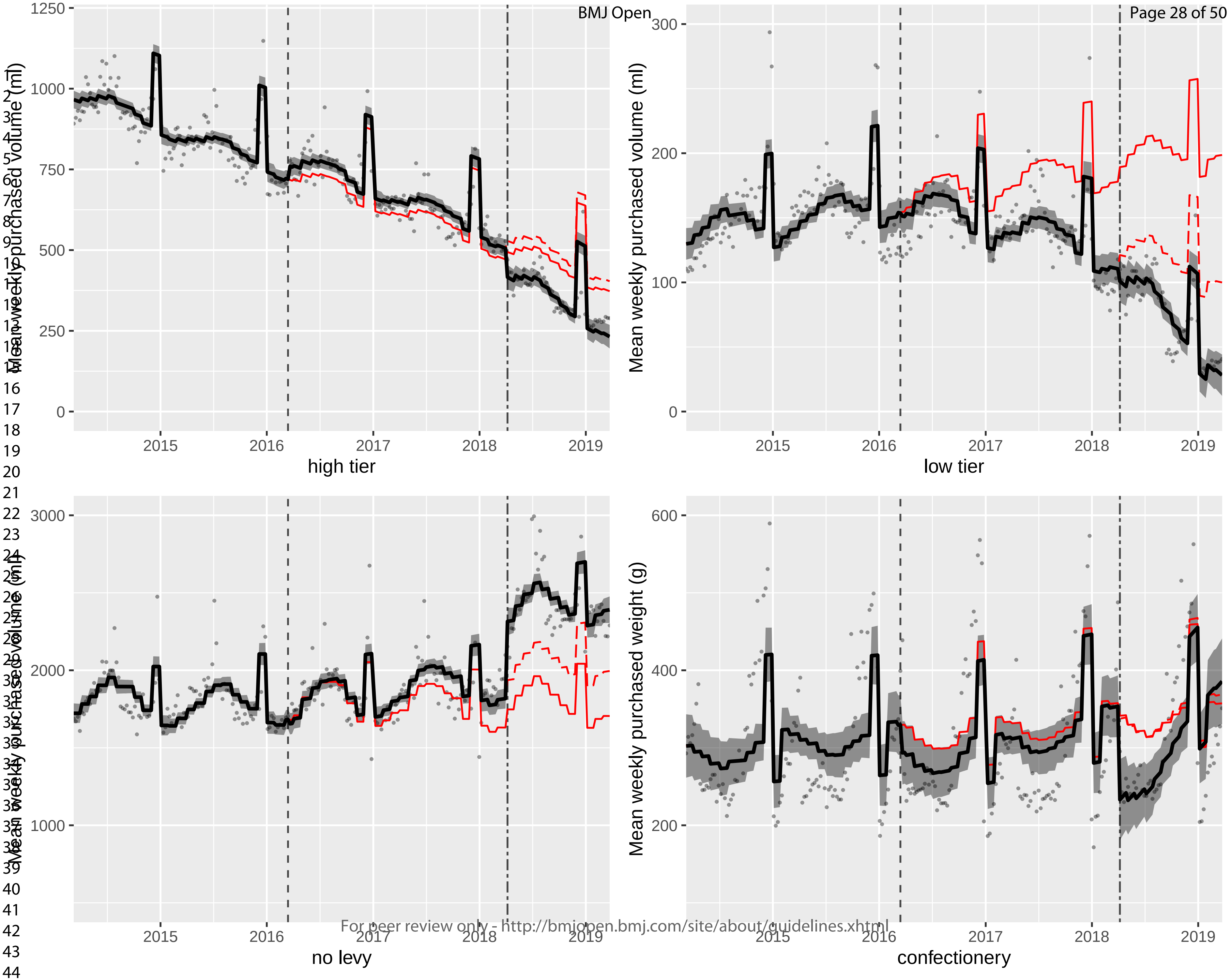
Mean weekly purchased volume (ml)
1250
1000
750
500
250
0
2015
2016
2017
2018
2019
high tier
300
200
100
low tier
3000
2000
1000
no levy
Mean weekly purchased weight (g)
600
400
confectionery

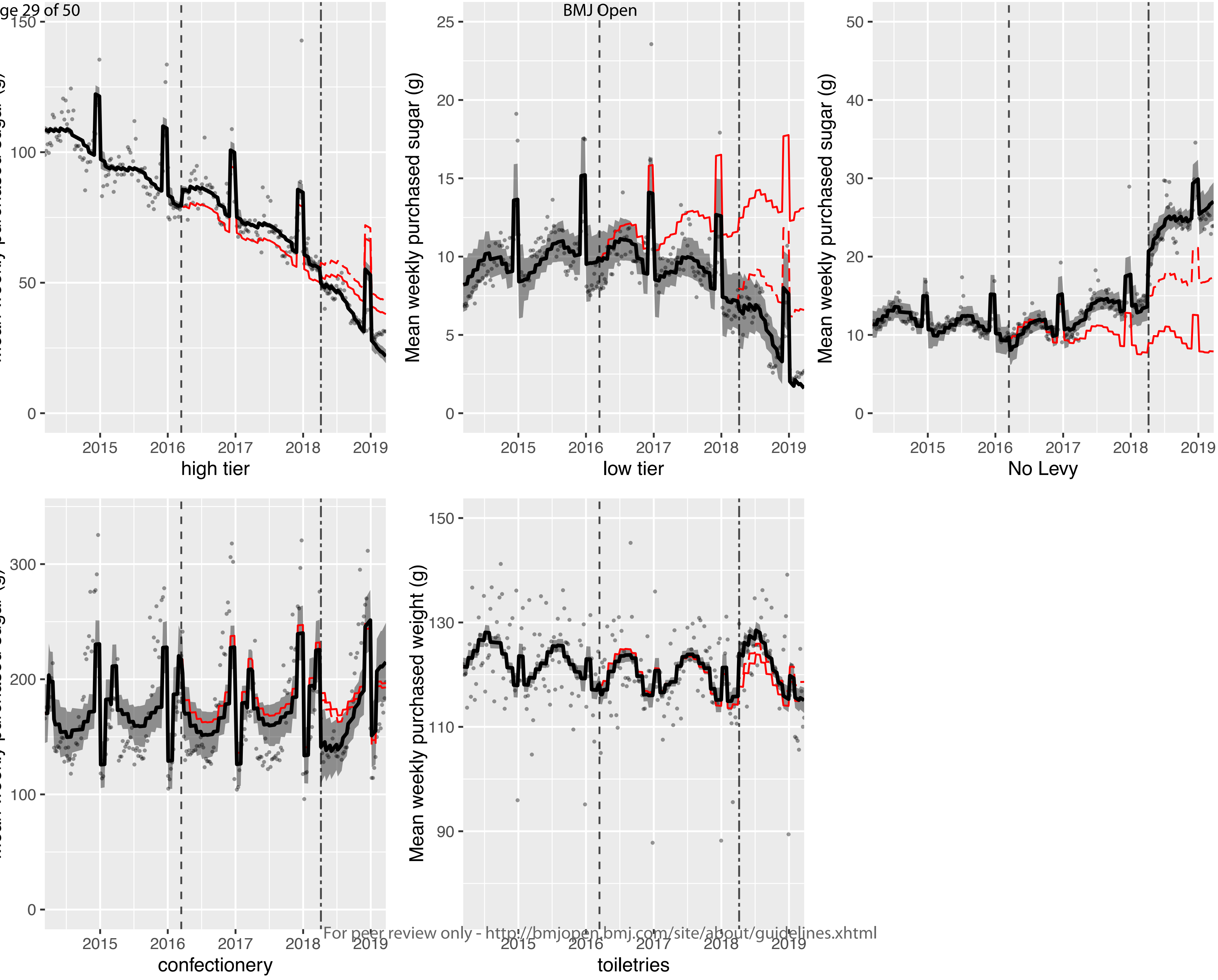
Mean weekly purchased sugar (g)
high tier
low tier
No Levy
confectionery
Mean weekly purchased weight (g)
toiletries
2015
2016
2017
2018
2019

Supplementary material A

STROBE Statement—checklist of items that should be included in reports of observational studies

	Item No	Recommendation	Section and Paragraph No
**Title and abstract**	1	(*a*) Indicate the study's design with a commonly used term in the title or the abstract	Title.
		(*b*) Provide in the abstract an informative and balanced summary of what was done and what was found	Abstract: main outcome measures; results; conclusions.
**Introduction**			
Background/rationale	2	Explain the scientific background and rationale for the investigation being reported	Introduction: paragraphs 1-3.
Objectives	3	State specific objectives, including any prespecified hypotheses	Introduction: paragraph 3-4.
**Methods**			
Study design	4	Present key elements of study design early in the paper	Methods: paragraph 1.
Setting	5	Describe the setting, locations, and relevant dates, including periods of recruitment, exposure, follow-up, and data collection	Methods: data source; overall analysis strategy; Figure 1.
Participants	6	(*a*) *Cohort study*—Give the eligibility criteria, and the sources and methods of selection of participants. Describe methods of follow-up *Case-control study*—Give the eligibility criteria, and the sources and methods of case ascertainment and control selection. Give the rationale for the choice of cases and controls *Cross-sectional study*—Give the eligibility criteria, and the sources and methods of selection of participants	Methods: data source, product categories – drinks, confectionery and toiletries; control group; outcome measures.
		(*b*) *Cohort study*—For matched studies, give matching criteria and number of exposed and unexposed *Case-control study*—For matched studies, give matching criteria and the number of controls per case	NA.
Variables	7	Clearly define all outcomes, exposures, predictors, potential confounders, and effect	Outcome measures; overall analysis strategy; primary analysis: category specific analyses; secondary analysis: all soft drinks categories combined, irrespective of levy

For peer review only

		modifiers. Give diagnostic criteria, if applicable	eligibility; sensitivity analysis: excluding small manufacturers; sensitivity analysis: ITS without a control category; supplementary material B.
Data sources/ measurement	8*	For each variable of interest, give sources of data and details of methods of assessment (measurement). Describe comparability of assessment methods if there is more than one group	Data source; overall analysis strategy; primary analysis: category specific analyses; secondary analysis: all soft drinks categories combined, irrespective of levy eligibility; sensitivity analysis: excluding small manufacturers; sensitivity analysis: ITS without a control category; supplementary material B.
Bias	9	Describe any efforts to address potential sources of bias	Product categories: drinks confectionery and toiletries; control group; sensitivity analysis: excluding small manufacturers; sensitivity analysis: ITS without a control category.
Study size	10	Explain how the study size was arrived at	Data source.
Quantitative variables	11	Explain how quantitative variables were handled in the analyses. If applicable, describe which groupings were chosen and why	Overall analysis strategy; primary analysis: category specific analyses; secondary analysis: all soft drinks categories combined, irrespective of levy eligibility; Box 1.
Statistical methods	12	(*a*) Describe all statistical methods, including those used to control for confounding	Control group; sensitivity analysis: ITS without a control category; Supplementary material A.
		(*b*) Describe any methods used to examine subgroups and interactions	Overall analysis strategy; primary analysis: category specific analyses; secondary analysis: all soft drinks categories combined, irrespective of levy eligibility.
		(*c*) Explain how missing data were addressed	Methods: data source
		(*d*) *Cohort study*—If applicable, explain how loss to follow-up was addressed<br>*Case-control study*—If applicable, explain how matching of cases and controls was addressed<br>*Cross-sectional study*—If applicable, describe analytical methods taking account of sampling strategy	Methods: data source.

Results

Participants	13*	(a) Report numbers of individuals at each stage of study—eg numbers potentially eligible, examined for eligibility, confirmed eligible, included in the study, completing follow-up, and analysed	Results: paragraph 1.
		(b) Give reasons for non-participation at each stage	Data source.
		(c) Consider use of a flow diagram	-
Descriptive data	14*	(a) Give characteristics of study participants (eg demographic, clinical,	Results: paragraph 1; Supplementary material C.

For peer review only

		social) and information on exposures and potential confounders	
		(b) Indicate number of participants with missing data for each variable of interest	Results: paragraph 1.
		(c) *Cohort study*—Summarise follow-up time (eg, average and total amount)	NA.
Outcome data	15*	*Cohort study*—Report numbers of outcome events or summary measures over time	NA.
		Case-control study—Report numbers in each exposure category, or summary measures of exposure	NA.
		Cross-sectional study—Report numbers of outcome events or summary measures	Results: paragraph 2; Table 1.
Main results	16	(*a*) Give unadjusted estimates and, if applicable, confounder-adjusted estimates and their precision (eg, 95% confidence interval). Make clear which confounders were adjusted for and why they were included	Primary analysis: category specific results; high tier drinks; low tier drinks; no levy drinks; levy exempt drinks and confectionery; Figures 2-3; Tables 2-3; supplementary material D.
		(*b*) Report category boundaries when continuous variables were categorized	Introduction - paragraph 3; product categories: drinks, confectionery and toiletries; Box 1.
		(*c*) If relevant, consider translating estimates of relative risk into absolute risk for a meaningful time period	Tables 2-3.
Other analyses	17	Report other analyses done—eg analyses of subgroups and interactions, and sensitivity analyses	Sensitivity analysis: excluding small manufacturers; sensitivity analysis: no control category; supplementary tables 4a-b; supplementary material G.
**Discussion**			
Key results	18	Summarise key results with reference to study objectives	Summary of main findings.
Limitations	19	Discuss limitations of the study, taking into account sources of potential bias or imprecision. Discuss both direction and magnitude of any potential bias	Key strengths and limitations.
Interpretation	20	Give a cautious overall interpretation of results considering objectives, limitations, multiplicity of analyses, results from similar studies, and other relevant evidence	Interpretation of findings - paragraphs 1-3.
Generalisability	21	Discuss the generalisability (external validity) of the study results	Interpretation of findings - paragraphs 4.
**Other information**			
Funding	22	Give the source of funding and the role of the funders for the present study and, if applicable, for the original study on which the present article is based	Funding.

*Give information separately for cases and controls in case-control studies and, if applicable, for exposed and unexposed groups in cohort and cross-sectional studies.

For peer review only

Note: An Explanation and Elaboration article discusses each checklist item and gives methodological background and published examples of transparent reporting. The STROBE checklist is best used in conjunction with this article (freely available on the Web sites of PLoS Medicine at http://www.plosmedicine.org/, Annals of Internal Medicine at http://www.annals.org/, and Epidemiology at http://www.epidem.com/). Information on the STROBE Initiative is available at www.strobe-statement.org.

For peer review only

Supplementary material B

The model specification is given by:

$Y_t = \beta_0 + \beta_1 T + \beta_2 A_t + \beta_3 A_t T + \beta_4 A_t Z + \beta_5 A_t TZ + \beta_6 I_t + \beta_7 I_t T + \beta_8 I_t Z + \beta_9 I_t TZ + e_t$

Y	*Average volume of (or purchased sugar in) drink or confectionery per household per week at week t (t=1,….,264)*
T	*Weeks since the start of the study; 1,…,264*
A_t	*0 if t prior to announcement, 1 if t on or after announcement*
I_t	*0 if t prior to implementation, 1 if t on or after implementation*
Z	*Control category (toiletries) = 0, drink or confectionery category = 1*
e_t	*N(0,σ^2) representing the residual variance of the model*

Dummy indicator variables determined to be statistically significant ($p<0.05$) were included for the intervention group as appropriate representing: interaction terms restart at 0 at the point of the interventions; the increase in purchases seen throughout December in the weeks before Christmas; the fall in purchases in the weeks immediately after Christmas; and the increase in confectionery purchases seen at Easter, for toiletries these were set to 0. To adjust for temperature-related trends in drink consumption the average UK monthly temperature was included in the intervention group with the average study period temperature used for toiletries.[5] Quadratic functions of announcement trends were included where they improved model fit - assessed using likelihood ratio tests. Stationary was examined using augmented Dickey-Fuller tests.[41] Autocorrelation between preceding time points was examined using autocorrelation and partial-autocorrelation plots. An appropriate autocorrelation structure was determined and then compared to alternative models using likelihood ratio tests. Visual inspection of the data suggested no additional benefit would be gained from including polynomial terms.

For peer review only

Supplementary material C

Supplementary Table 1: Demographic characteristics of Kantar Worldpanel take-home panel households from March 2014 – March 2018 (weighted)

	Level	Kantar Worldpanel (%)	UK population (%)
Children in household[1]	No	65.3	71.5
	Yes	34.6	28.5
Social grade of chief income earner[2]	AB: Higher and intermediate managerial	19.3	27
	C1: Junior managerial	34.1	28
	C2: Skilled manual workers	16.4	20
	D: Semi and unskilled-manual workers	12.4	15
	E: lowest grade workers	7.9	10
Total household income (£ per annum)[3]	0-9,999	6.4	..
	10,000-19,999	18.9	..
	20,000-29,999	17.2	..
	30,000-39,999	13.1	....
	40,000-49,999	9.0	..
	50,000-59,999	5.5	..
	60,000-69,999	2.9	..
	70,000+	4.1	..
	Refused to answer	14.4	..
	Mean (£)	..	35,697
	Median (£)	..	28,947
Highest qualification of chief income earner[4]	Higher than School leaving qualifications taken at ~18 years (e.g. A-Levels)	38.3	43.8
	School leaving qualifications taken at ~18 years (e.g. A-Levels)	12.3	22.4
	School leaving qualifications taken at ~16 years (e.g. GCSE)	20.6	18.7
	Other (including no qualifications and unknown)	16.2	15.1

[1]Average of households with dependent children from 2014-2018; [2]UK population figures from 2016; [3]No directly comparable figures available from ONS, mean and medians are averaged over 2014-2019; [4]UK population figures from 2014

For peer review only

Supplementary material D

Supplementary Figure 1a. Observed and modelled volume (ml) of drinks exempt from the Soft Drinks Industry Levy purchased per household per week, March 2014- March 2019 (weighted)

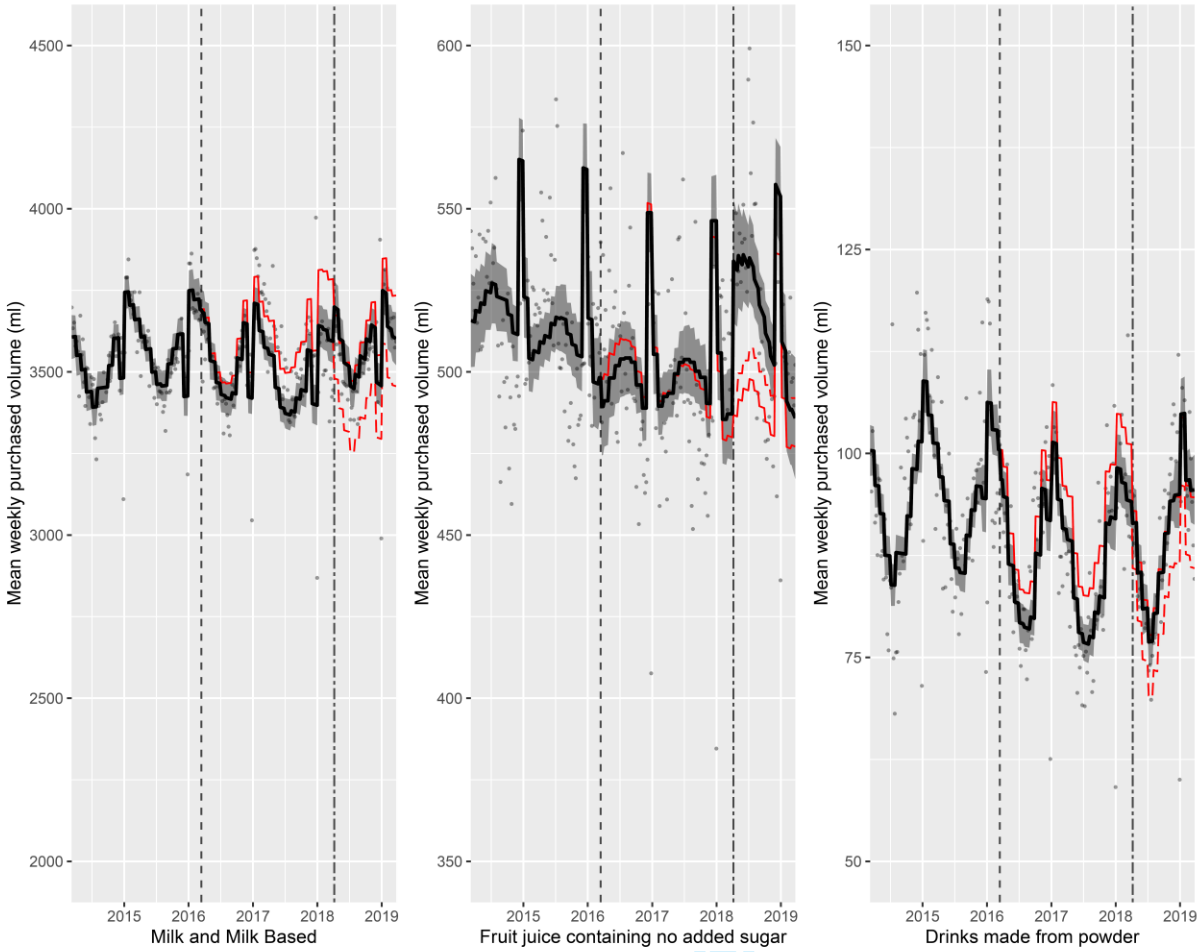

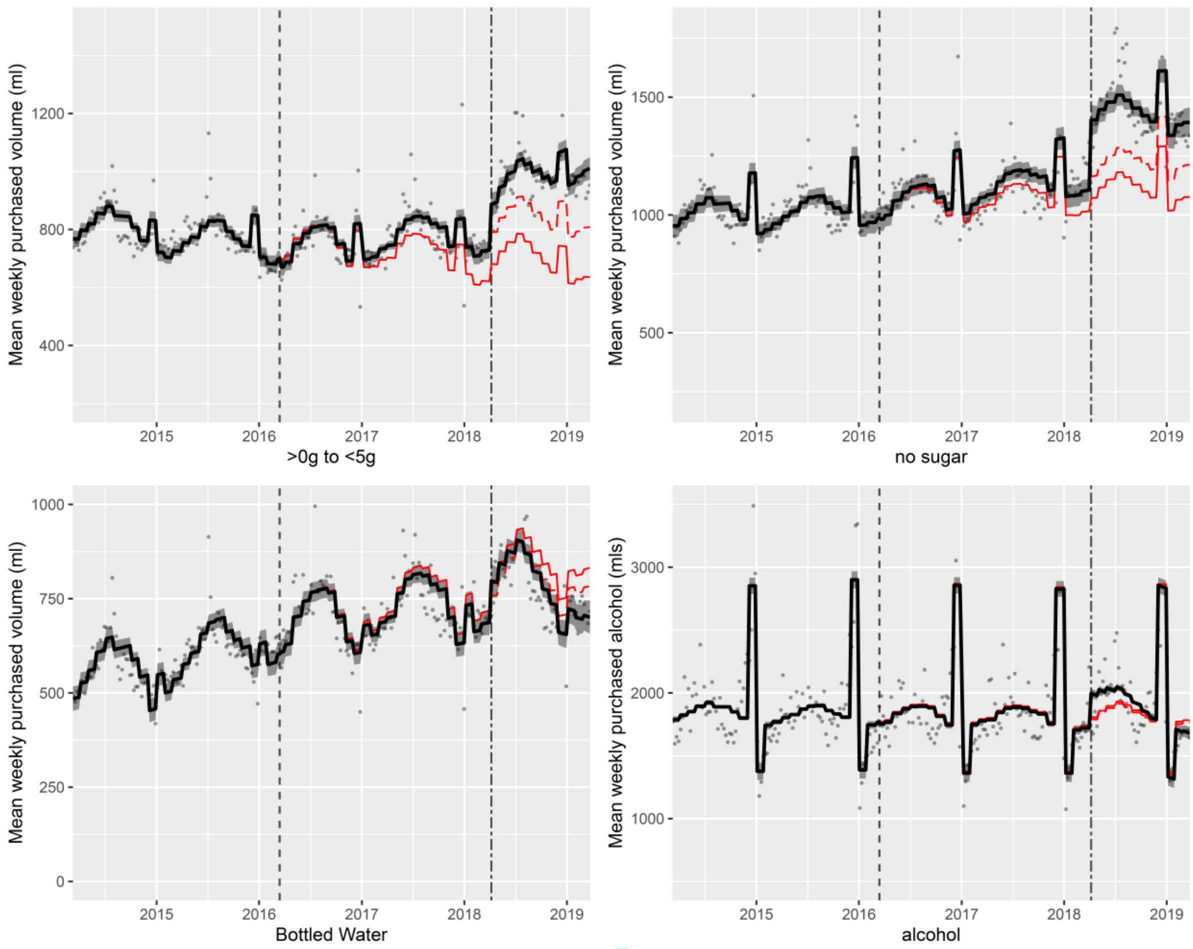

Notes. Points are observed data, black lines (with shadows) are modelled data (and 95% confidence intervals); red lines indicate counterfactuals had the announcement (red solid line) and implementation (red dashed line) not happened; the first dashed vertical line indicates the point of announcement; the second dashed vertical line indicates the point of implementation; The Y-axis varies in scale between panels to maximise the resolution of figures; modelled purchases include averaged effects for seasonality and the impact of December and January (Christmas period). The control category of toiletries is shown in Figure 3.

view only

Supplementary Figure 1b. Observed and modelled amount of sugar in drinks exempt from the Soft Drinks Industry Levy purchased per household per week, March 2014- March 2019 (weighted)

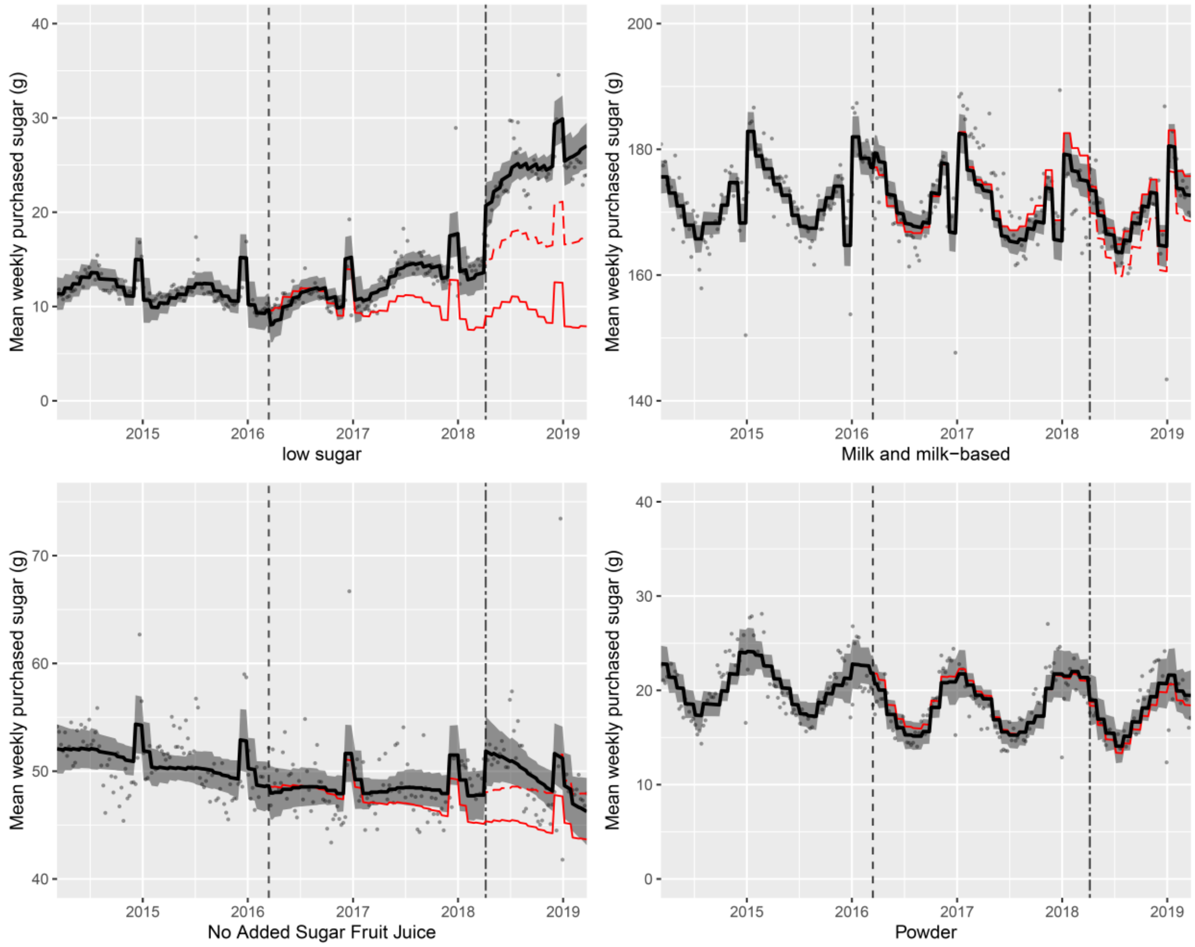

Notes. Points are observed data, black lines (with shadows) are modelled data (and 95% confidence intervals); red lines indicate counterfactuals had the announcement (red solid line) and implementation (red dashed line) not happened; the first dashed vertical line indicates the point of announcement; the second dashed vertical line indicates the point of implementation; The Y-axis varies in scale between panels to maximise the resolution of figures; modelled purchases include averaged effects for seasonality and the impact of December and January (Christmas period). The control category of toiletries is shown in Figure 3.

Supplementary Table 2a: Modelled level and trend changes in volume of drinks (ml) and confectionery (g) purchased per household per week (95% CI) in relation to the UK SDIL, March 2014 - March 2019 (weighted)

	Analysis 1: pre-post announcement (March 2014 – March 2018)		Analysis 2: pre-post implementation (March 2016 – March 2019)		Analysis 3: pre-post announcement & implementation (March 2014 – March 2019)	
	Level Change	Trend Change	Level Change	Trend Change	Level Change	Trend Change
Levy liable drinks						
High tier (≥8g sugar per 100ml)	44.0 (-7.7, 95.7)	-0.1 (-0.9, 0.7)	**-117.2 (-183.3, -51.1)**	1.0 (-0.9, 2.9)	-73.2 (-157.1, 10.7)	0.9 (-1.2, 3.0)
Low tier (≥5g - <8g sugar per 100ml)	-0.11 (-22.4, 22.1)	**-0.6 (-1.0, -0.3)**	-26.3 (-53.6, 1.04)	-0.81 (-1.6, 0.01)	-26.4 (-61.7, 8.8)	**-1.5 (-2.4, -0.6)**
No levy (<5g sugar per 100ml)	-28.2 (-149.4, 92.9)	**2.0 (0.04, 3.9)**	**372.0 (217.6, 526.4)**	**0.52 (-3.9, 4.9)**	**343.8 (147.5, 540.0)**	2.5 (-2.3, 7.3)
>0g to <5g sugar per 100ml	-27.1 (-82.6, 28.5)	**1.3 (0.4, 2.2)**	**88.1 (17.0, 159.3)**	**2.31 (0.3, 4.3)**	61.0 (-29.2, 151.4)	**3.6 (1.4, 5.8)**
0g sugar per 100ml	-7.37 (-93.9, 79.2)	0.9 (-0.6, 2.4)	**231.0 (125.2, 336.8)**	-0.99 (-4.3, 2.3)	**223.6 (87.0, 360.3)**	-0.08 (-3.7, 3.5)
Bottled water	6.86 (-53.4, 67.2)	-0.4 (-1.4, 0.6)	36.6 (-38.8, 112.0)	-2.24 (-4.5, 0.005)	43.5 (-53.1, 140.0)	**-2.6 (-5.1, -0.2)**
Levy exempt drinks						
Alcoholic drinks	-10.8 (-48.6, 27.0)	-0.07 (-0.5, 0.3)	**208.0 (137.9, 278.1)**	**-5.7 (-7.8, -3.6)**	**197.2 (117.6, 276.8)**	**-5.8 (-7.9, -3.7)**
Milk and milk based drinks[1]	-6.61 (-105.8, 92.6)	-1.7 (-4.0, 0.5)	**210.7 (98.7, 322.7)**	-1.2 (-5.3, 2.9)	**204.1 (54.4, 353.7)**	-2.9 (-7.6, 1.8)
No added sugar fruit juices	-8.72 (-34.3, 16.9)	0.1 (-0.3, 0.6)	30.5 (-0.9, 61.9)	-0.7 (-1.6, 0.3)	21.78 (-18.8, 62.3)	-0.5 (-1.6, 0.5)
Drinks sold as powders (g)	-1.79 (-7.8, 4.18)	-0.06 (-0.2, 0.03)	0.10 (-7.7, 7.9)	**0.3 (0.04, 0.5)**	-1.69 (-11.6, 8.17)	0.2 (-0.04, 0.4)
Confectionery (g)	-17.0 (-88.0, 54.0)	0.07 (-1.3, 1.5)	-77.9 (-163.9, 8.19)	2.4 (-0.6, 5.4)	**-98.9 (-206.4, -16.7)**	2.5 (-0.8, 5.8)

Notes. Bold indicates a significant difference at the 95% confidence interval level. [1]Milk comprises drinks in the following categories: semi-skimmed; specific low fat % milk (e.g. 1% fat milk); whole milk; buttermilk; modified milk; other milk; other non-cows milk; rice drink; soya milk. Skimmed milk is excluded from all analysis in this paper due to missing data.

For peer review only

Supplementary Table 2b: Modelled level and trend changes in sugar in drinks (ml) and confectionery (g) purchased per household per week (95% CI) in relation to the UK SDIL, March 2014 - March 2019 (weighted)

	Analysis 1: pre-post announcement (March 2014 – March 2018)		Analysis 2: pre-post implementation (March 2016 – March 2019)		Analysis 3: pre-post announcement & implementation (March 2014 – March 2019)	
	Level Change	Trend Change	Level Change	Trend Change	Level Change	Trend Change
Levy liable drinks						
High tier (≥8g sugar per 100ml)	**8.9 (6.8, 10.9)**	**-0.05 (-0.08, -0.03)**	**-11.5 (-15.3, -7.7)**	**-0.1 (-0.2, -0.02)**	-2.6 (-6.9, 1.6)	**-0.2 (-0.3, -0.07)**
Low tier (≥5g - <8g sugar per 100ml)	1.7 (-1.6, 5.1)	**-0.07 (-0.1, -0.02)**	**-6.2 (-10.6, -1.8)**	0.09 (-0.03, 0.2)	-4.5 (-10.0, 1.1)	0.02 (-0.1, 0.2)
No levy (<5g sugar per 100ml)	0.2 (-3.1, 3.5)	0.04 (-0.01, 0.09)	0.8 (-3.6, 5.3)	**0.2 (0.1, 0.4)**	1.0 (-4.5, 6.6)	**0.3 (0.2, 0.4)**
>0g to <5g sugar per 100ml	0.2 (-3.1, 3.5)	0.04 (-0.01, 0.09)	0.8 (-3.6, 5.3)	**0.2 (0.1, 0.4)**	1.0 (-4.5, 6.6)	**0.3 (0.2, 0.4)**
Levy exempt drinks						
Milk and milk based drinks[1]	4.1 (-0.1, 9.1)	**-0.09 (-0.2, -0.01)**	-2.0 (-8.6, 4.6)	**0.2 (0.01, 0.4)**	2.1 (-6.2, 10.4)	0.1 (-0.09, 0.3)
No added sugar fruit juices	1.0 (-3.4, 5.4)	0.001 (-0.06, 0.06)	-1.6 (-7.3, 4.1)	0.07 (-0.09, 0.2)	-0.6 (-7.8, 6.6)	0.07 (-0.1, 0.2)
Drinks sold as powders (g)	0.6 (-3.3, 4.5)	-0.02 (-0.08, 0.04)	-5.1 (-0.004, 10.2)	**0.2 (0.03, 0.3)**	-4.5 (-10.9, 1.9)	**0.2 (0.001, 0.3)**
Confectionery (g)	-8.6 (-49.7, 32.6)	0.03 (-0.8, 0.9)	-49.4 (-99.2, 0.4)	1.5 (-0.2, 3.2)	**-58.0 (-122.6, -6.7)**	1.6 (-0.4, 3.5)

Notes. Bold indicates a significant difference at the 95% confidence interval level. [1]Milk comprises drinks in the following categories: semi-skimmed; specific low fat % milk (e.g. 1% fat milk); whole milk; buttermilk; modified milk; other milk; other non-cows milk; rice drink; soya milk. Skimmed milk is excluded from all analysis in this paper due to missing data.

For peer review only

Supplementary material E

Secondary analysis: all drinks categories combined irrespective of levy eligibility

Supplementary Table 3a: Level and trend changes in volume of, and sugar in, all drinks (ml) and confectionery (g) purchased per household per week (95% CI) in relation to the UK SDIL, March 2014 - March 2019 (weighted)

	Analysis 1: pre-post announcement (March 2014 – March 2018)		Analysis 2: pre-post implementation (March 2016 – March 2019)		Analysis 3: pre-post announcement & implementation (March 2014 – March 2019)	
	Level Change	Trend Change	Level Change	Trend Change	Level Change	Trend Change
All drinks - volume						
Higher tier (≥8g sugar per 100ml)	22.8 (-37.5, 83.1)	0.5 (-0.5, 1.5)	-76.8 (-154.0 ,0.4)	-2.2 (-4.4, 0.01)	-54.0 (-152.0, 44.0)	-1.7 (-4.1, 0.7)
Lower tier (≥5g - <8g sugar per 100ml)	19.0 (-52.2, 90.2)	**-3.2 (-5.9, -0.5)**	27.9 (-47.8, 103.6)	-4.4 (-8.9, 0.02)	46.9 (-57.0, 150.8)	**-7.6 (-12.9, -2.4)**
Drinks containing <5g sugar per 100ml)	-152.1 (-348.3, 44.1)	2.6 (-0.7, 5.8)	**435.7 (187.7, 683.7)**	1.1 (-6.0, 8.3)	283.6 (-32.6, 599.8)	3.7 (-4.2, 11.5)
Levy liable drinks combined	15.2 (-72.9, 103.3)	0.6 (-0.8, 2.0)	-39.7 (-152.7, 73.4)	0.01 (-3.2, 3.2)	-24.5 (-167.8, 118.9)	0.6 (-2.9, 4.1)
All drinks excluding milk[1]	12.9 (-205.3, 231.0)	1.0 (-2.6, 4.5)	**347.6 (70.1, 625.0)**	-5.2 (-13.1, 2.7)	**360.5 (7.47, 713.4)**	-4.2(-12.9, 4.5)
All drinks combined	33.9 (-189.0, 256.7)	-0.2 (-3.8, 3.4)	**453.0 (170.1, 735.9)**	-5.1 (-13.2, 3.0)	**486.9 (126.7, 847.0)**	-5.4 (-14.2, 3.5)
All drinks – sugar						
Higher tier (≥8g sugar per 100ml)	4.1 (-2.6, 10.8)	0.04 (-0.07, 1.1)	**-13.4 (-22.2, -4.7)**	-0.1 (-0.4, 0.1)	-9.3 (-20.3, 1.7)	-0.08 (-0.3, 0.2)
Lower tier (≥5g - <8g sugar per 100ml)	**11.0 (5.6, 16.3)**	**-0.2 (-0.3, -0.1)**	-2.7 (-9.67, 4.3)	-0.01 (-0.2,0.2)	8.3 (-0.5, 17.1)	**-0.2 (-0.4, -0.01)**
Drinks containing <5g sugar per 100ml)	-6.3 (-12.2, -0.4)	**1.0 (0.9, 1.0)**	-0.7 (-8.30, 7.0)	**0.4 (0.2, 0.6)**	-7.0 (-16.6, 2.7)	**1.4 (1.1, 1.6)**
Levy liable drinks combined	5.0 (-1.8, 11.8)	-0.003 (-0.1, 0.1)	**-13.1 (-22.0, -4.2)**	0.02 (-0.2, 0.3)	-8.1(-19.3, 3.1)	0.02 (-0.3, 0.3)
All drinks excluding milk[1]	3.1 (-4.6, 10.8)	0.04 (-0.08, 0.2)	-9.3 (-19.2, 0.7)	-0.09 (-0.4, 0.2)	-6.2 (-18.8, 6.4)	-0.05 (-0.4, 0.3)
All drinks combined	5.3 (-2.6, 13.2)	-0.02 (-0.1, 0.1)	-5.7 (-15.9, 4.5)	-0.08 (-0.4, 0.2)	-0.4 (-13.2, 12.5)	-0.1 (-0.4, 0.2)

Notes. Bold indicates a significant difference at the 95% confidence interval level. The levy liable drinks category is a combination of high tier, low tier and no levy drinks. [1]Milk comprises drinks in the following categories: semi-skimmed; specific low fat % milk (e.g. 1% fat milk); whole milk; buttermilk; modified milk; other milk; other non-cows milk; rice drink; soya milk. Skimmed milk is excluded from all analysis in this paper due to missing data.

For peer review only

Supplementary Table 3b: Absolute and relative change in volume of, and sugar in, all drinks and confectionery (g) purchased per household (95% CI) per week in relation to the UK SDIL, March 2014- March 2019 (weighted)

	Analysis 1: pre-post announcement (March 2014 – March 2018)		Analysis 2: pre-post implementation (March 2016 – March 2019)		Analysis 3: pre-post announcement & implementation (March 2014 – March 2019)	
	Absolute change (ml or g)	Relative change (%)	Absolute change (ml or g)	Relative change (%)	Absolute change (ml or g)	Relative change (%)
All drinks - volume						
High tier (≥8g sugar per 100ml)	**76.6 (45.6, 107.7)**	**7.5 (4.5, 10.6)**	**-190.9 (-233.5, -148.3)**	**-19.4 (-23.7, -15.1)**	**-85.6 (-128.2, -43.0)**	**-9.8 (-14.6, -4.9)**
Low tier (≥5g - <8g sugar per 100ml)	316.7 (-241,7, -391.7)	**-28.0 (-21.3, -34.6)**	**-205.2 (-295.9, -114.5)**	**-26.2 (-37.8, -14.6)**	**-693.6 (-784.3, -602.9)**	**-54.5 (-61.7, -47.4)**
Drinks containing <5g sugar per 100ml)	**114.3 (12.4, 216.2)**	**2.2 (0.2, 4.1)**	**506.7 (367.3, 646.1)**	**9.0 (6.5, 11.4)**	**760.2 (620.8, 899.6)**	**14.1 (11.5, 16.6)**
Levy liable drinks combined	**76.5 (31.1, 121.8)**	**6.0 (2.5, 9.6)**	-42.7 (-104.9, 19.6)	-3.3(-8.1, 1.5)	**66.3 (4.1, 128.6)**	**5.6 (0.3, 10.8)**
All drinks excluding milk[1]	**116.1 (3.3, 229.0)**	**3.3 (0.09, 6.4)**	79.6 (-74.8, 234.1)	2.1 (-2.0, 6.1)	**250.1 (95.7, 404.5)**	**6.8 (2.6, 11.0)**
All drinks combined	11.8 (-103.7, 127.3)	0.2 (-1.4, 1.7)	**187.8 (29.7, 345.9)**	**2.6 (0.4, 4.7)**	**188.8 (30.7, 346.9)**	**2.6 (0.4, 4.7)**
All drinks – sugar						
High tier (≥8g sugar per 100ml)	**9.3 (5.9, 12.8)**	**7.6 (4.8, 10.4)**	**-22.9 (-27.8, -18.1)**	**-19.4 (-23.4, -15.3)**	**-9.99 (-14.8, -5.18)**	**-9.5 (-14.0, -4.9)**
Low tier (≥5g - <8g sugar per 100ml)	**-9.7 (-12.5, -6.9)**	**-18.6 (-23.9, -13.3)**	**-7.2 (-11.0, -3.4)**	**-19.4 (-29.7, -9.1)**	**-26.7 (-30.5, -22.9)**	**-47.1 (-53.9, -40.4)**
Drinks containing <5g sugar per 100ml)	**5.1 (2.1, 8.1)**	**3.6 (1.5, 5.8)**	**16.7 (12.5, 20.9)**	**11.2 (8.4, 14.0)**	**28.6 (24.4, 32.8)**	**20.9 (17.9, 23.9)**
Levy liable drinks combined	**6.1 (2.6, 9.6)**	**8.6 (3.6, 13.5)**	**-15.5 (-20.4, -10.7)**	**-22.7 (-29.8, -15.6)**	**-8.0 (-12.9, -3.2)**	**-13.2 (-21.1, -5.2)**
All drinks excluding milk[1]	**8.9 (5.0, 12.8)**	**6.5 (3.6, 9.4)**	**-17.2 (-22.7, -11.8)**	**-12.7 (-16.7, -8.67)**	**-4.5 (-10.0, 1.0)**	**-3.7 (-8.1, 0.8)**
All drinks combined	**4.6 (0.5, 8.6)**	**1.4 (0.2, 2.7)**	**-12.9 (-18.5, -7.4)**	**-4.3 (-6.1, -2.4)**	**-8.0 (-13.6, -2.4)**	**-2.7 (-4.5, -0.8)**

Notes. Bold indicates a significant difference at the 95% confidence interval level. The levy liable drinks category is a combination of high tier, low tier and no levy drinks. [1]Milk comprises drinks in the following categories: semi-skimmed; specific low fat % milk (e.g. 1% fat milk); whole milk; buttermilk; modified milk; other milk; other non-cows milk; rice drink; soya milk. Skimmed milk is excluded from all analysis in this paper due to missing data.

For peer review only

Supplementary material F

Sensitivity analysis: excluding small manufacturers

Supplementary table 4a: Modelled level and trend changes in volume of, and sugar in, drinks (ml) purchased per household per week (95% CI) in relation to the UK SDIL, March 2014 - March 2019; excluding small manufacturers (weighted)

	Analysis 1: pre-post announcement (March 2014 – March 2018)		Analysis 2: pre-post implementation (March 2016 – March 2019)		Analysis 3: pre-post announcement & implementation (March 2014 – March 2019)	
	Level Change	Trend Change	Level Change	Trend Change	Level Change	Trend Change
Volume						
High levy tier (≥8g sugar per 100ml)						
All Manufacturers	44.0 (-7.7, 95.7)	-0.1 (-0.9, 0.7)	**-117.2 (-183.3, -51.1)**	1.0 (-0.9, 2.9)	-73.2 (-157.1, 10.7)	0.9 (-1.2, 3.0)
Excluding Manufacturers with <1M Litres	41.5 (-9.7, 92.7)	-0.09 (-0.9, 0.7)	**-111.9 (-177.3, -46.6)**	-1.1 (-2.9, 0.8)	-70.8 (-153.4, 12.6)	-1.2 (-3.2, 0.9)
Excluding Manufacturers with <0.5M Litres	41.5 (-9.7, 92.7)	-0.09 (-0.9, 0.7)	**-111.9 (-177.3, -46.6)**	-1.1 (-2.9, 0.8)	-70.8 (-153.4, 12.6)	-1.2 (-3.2, 0.9)
Low levy tier (≥5g - <8g sugar per 100ml)						
All Manufacturers	-0.1 (-22.4, 22.1)	**-0.6 (-1.0, -0.3)**	-26.3 (-53.6, 1.0)	-0.8 (-1.6, 0.01)	-26.4 (-61.7, 8.8)	**-1.5 (-2.4, -0.6)**
Excluding Manufacturers with <1M Litres	-2.1 (-24.0, 19.9)_	**-0.7 (-1.0, -0.3)**	-20.9 (-47.5, 5.7)	**-0.9 (-1.8, -0.1)**	-23.0 (-57.5, 11.6)	**-1.6 (-2.5, -0.7)**
Excluding Manufacturers with <0.5M Litres	-2.1 (-24.0, 19.6)_	**-0.7 (-1.0, -0.3)**	-20.7 (-47.3, 5.9)	**-0.9 (-1.8, -0.1)**	-22.9 (-57.2, 11.5)	**-1.6 (-2.5, -0.7)**
Sugar						
High tier (≥8g sugar per 100ml)						
All Manufacturers	**8.9 (6.8, 10.9)**	**-0.05 (-0.08, -0.03)**	**-11.5 (-15.3, -7.7)**	**-0.1 (-0.2, -0.02)**	-2.6 (-6.9, 1.6)	**-0.2 (-0.3, -0.07)**
Excluding Manufacturers with <1M Litres	4.5 (-1.3, 10.2)	-0.03 (-0.1, 0.06)	**-13.5 (-20.8, -6.1)**	-0.07 (-0.3, 0.1)	-9.0 (-18.3, 0.4)	-0.1 (-0.3, 0.1)
Excluding Manufacturers with <0.5M Litres	4.5 (-1.3, 10.2)	-0.03 (-0.1, 0.06)	**-13.5 (-20.8, -6.1)**	-0.07 (-0.3, 0.1)	-9.0 (-18.3, 0.4)	-0.1 (-0.3, 0.1)
Low tier (≥5g - <8g sugar per 100ml)						
All Manufacturers	1.7 (-1.6, 5.1)	**-0.07 (-0.1, -0.02)**	**-6.2 (-10.6, -1.8)**	0.09 (-0.03, 0.2)	-4.5 (-10.0, 1.1)	0.02 (-0.1, 0.2)
Excluding Manufacturers with <1M Litres	-0.4 (-2.1, 1.3)	**-0.07 (-0.1, -0.04)**	**-2.5 (-4.6, -0.4)**	0.002 (-0.06, 0.06)	**-3.0 (-5.7, -0.2)**	-0.007 (-0.1, 0.0001)
Excluding Manufacturers with <0.5M Litres	-0.5 (-2.1, 1.2)	**-0.07 (-0.1, -0.04)**	**-2.5 (-4.7, -0.4)**	0.003 (-0.06, 0.06)	**-3.0 (-5.7, -0.2)**	-0.007 (-0.1, 0.0001)

For peer review only

Notes. Bold indicates a significant difference at the 95% confidence interval level.

Supplementary Table 4b: Modelled absolute and relative change in volume of, and sugar in, all drinks (ml) purchased per household (95% CI) per week in relation to the UK SDIL, March 2014- March 2019; excluding small manufacturers (weighted)

	Analysis 1: pre-post announcement (March 2014 – March 2018)		Analysis 2: pre-post implementation (March 2016 – March 2019)		Analysis 3: pre-post announcement & implementation (March 2014 – March 2019)	
	Absolute change (ml)	Relative change (%)	Absolute change (ml)	Relative change (%)	Absolute change (ml)	Relative change (%)
Volume						
High tier (≥8g sugar per 100ml)						
All Manufacturers	**34.7 (8.1, 61.4)**	**7.3 (1.7, 12.9)**	**-171.6 (-208.1, -135.1)**	**-42.5 (-51.6, -33.5)**	**-140.8 (-177.3, -104.3)**	**-37.8 (-47.6, -28.0)**
Excluding Manufacturers with <1M Litres	**35.3 (8.9, 61.6)**	**7.5 (1.9, 13.1)**	**-168.1 (-204.2, -131.9)**	**-42.6 (-51.7, -33.4)**	**-136.2 (-172.3, -100.1)**	**-37.6 (-47.5, -27.6)**
Excluding Manufacturers with <0.5M Litres	**35.3 (8.9, 61.6)**	**7.5 (1.9, 13.1)**	**-168.1 (-204.2, -131.9)**	**-42.6 (-51.7, -33.4)**	**-136.2 (-172.3, -100.1)**	**-37.6 (-47.5, -27.6)**
Low tier (≥5g - <8g sugar per 100ml)						
All Manufacturers	**-65.7 (-77.5, -53.8)**	**-37.1 (-43.7, -30.4)**	**-71.8 (-87.8, -55.8)**	**-71.8 (-87.8, -55.8)**	**-170.5 (-186.5, -154.5)**	**-85.8 (-93.9, -77.8)**
Excluding Manufacturers with <1M Litres	**-66.4 (-78.2, -54.6)**	**-37.6 (-44.3, -30.9)**	**-71.2 (-87.1, -55.3)**	**-72.5 (-88.7, -56.3)**	**-171.1 (-187.0, -155.2)**	**-86.4 (-94.4, -78.4)**
Excluding Manufacturers with <0.5M Litres	**-66.5 (-78.2, -54.7)**	**-37.6 (-44.2, -31.0)**	**-71.3 (-87.1, -55.5)**	**-72.4 (-88.5, -56.3)**	**-171.2 (-187.1, -155.4)**	**-86.3 (-94.3, -78.3)**
Sugar						
High tier (≥8g sugar per 100ml)						
All Manufacturers	**5.5 (3.8, 7.3)**	**10.8 (7.4, 14.1)**	**-21.2 (-23.8, -18.5)**	**-49.3 (-55.4, -43.1)**	**-16.2 (-18.8, -13.5)**	**-42.6 (-49.6, -35.6)**
Excluding Manufacturers with <1M Litres	**4.7 (1.7, 7.7)**	**9.3 (3.4, 15.1)**	**-19.3 (-23.4, -15.2)**	**-44.3 (-53.6, -35.0)**	**-14.6 (-18.7, -10.6)**	**-37.6 (-48.1, -27.1)**
Excluding Manufacturers with <0.5M Litres	**4.7 (1.7, 7.7)**	**9.3 (3.4, 15.1)**	**-19.3 (-23.4, -15.2)**	**-44.3 (-53.6, -35.0)**	**-14.6 (-18.7, -10.6)**	**-37.6 (-48.1, -27.1)**
Low tier (≥5g - <8g sugar per 100ml)						
All Manufacturers	**-4.3 (-6.1, -2.6)**	**-37.5 (-52.5,-22.5)**	**-5.0 (-7.4, -2.6)**	**-75.8 (-112.7, -38.9)**	**-11.5 (-13.9, -9.07)**	**-87.8 (-106.4, -69.2)**
Excluding Manufacturers with <1M Litres	**-4.7 (-5.5, -3.8)**	**-39.1 (-46.5, -31.7)**	**-4.8 (-6.0, -3.6)**	**-73.0 (-91.3, -54.7)**	**-11.8 (-13.0, -10.6)**	**-86.9 (-95.8, -78.1)**
Excluding Manufacturers with <0.5M Litres	**-4.7 (-5.6, -3.8)**	**-39.1 (-46.5, -31.8)**	**-4.8 (-6.0, -3.6)**	**-72.9 (-91.1, -54.6)**	**-11.8 (-13.0, -10.6)**	**-86.9 (-95.7, -78.0)**

Notes. Bold indicates a significant difference at the 95% confidence interval level.

For peer review only

Supplementary Figure 2. Observed and modelled volume (ml) and amount of sugar (g) in all drinks combined purchased per household per week, March 2014- March 2019 (weighted)

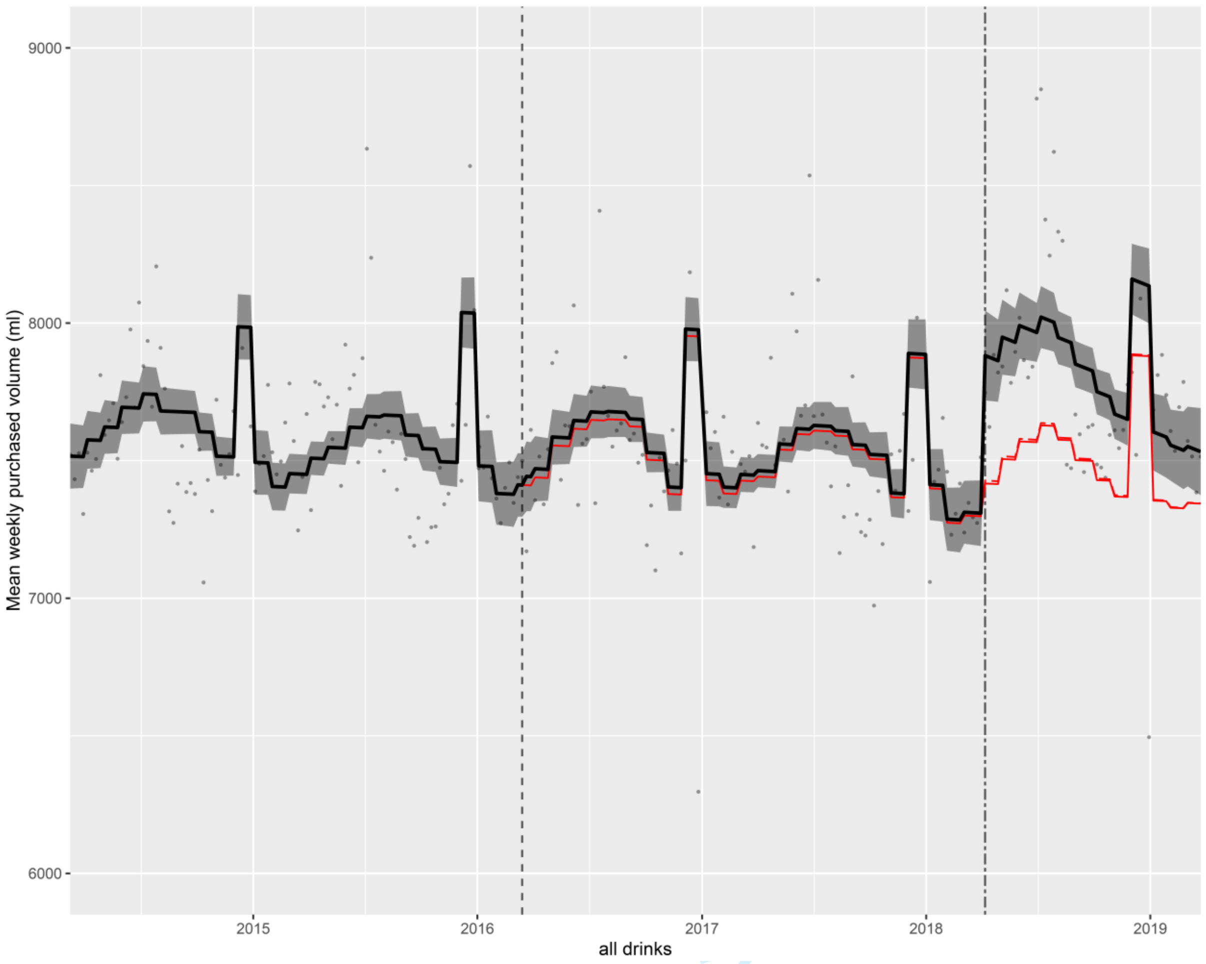

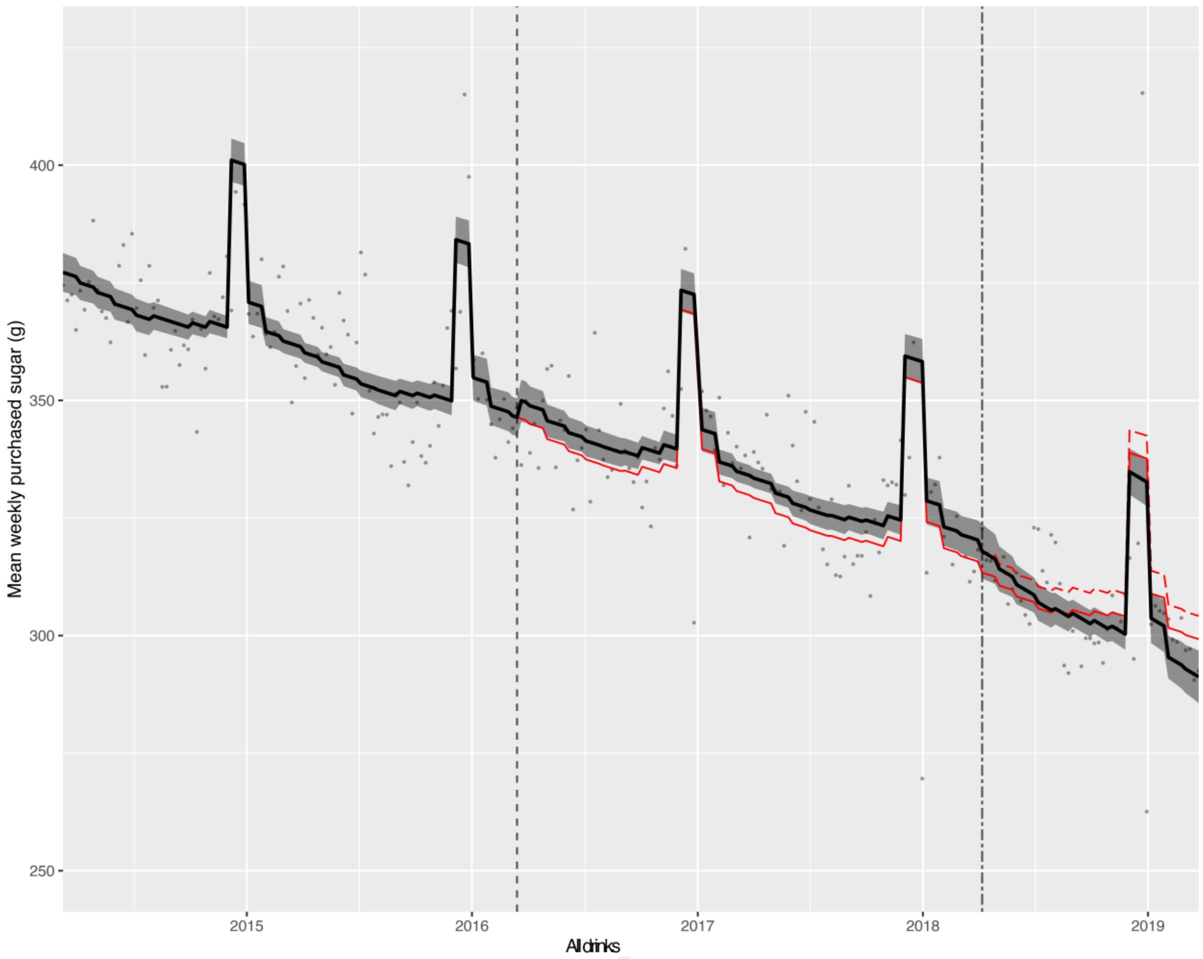

Notes. Points are observed data, black lines (with shadows) are modelled data (with 95% confidence intervals); red lines indicate the counterfactuals had the announcement (red solid line) and implementation (red dashed line) not happened; the first dashed vertical line indicates the point of announcement; the second dashed vertical line indicates the point of implementation; The Y-axis varies in scale between panels to maximise the resolution of figures; modelled purchases include averaged effects for seasonality and the impact of December and January (the Christmas period). The control category of toiletries is shown in Fig 3.

Supplementary material G

Removing the control category led to wider confidence intervals in a small number of cases such that absolute and relative changes in volume were not significantly different from the pre-implementation counterfactuals for no levy drinks and the pre-announcement and post implementation counterfactual for drinks containing 0g of sugar per 100ml. Significantly lower volumes of purchased volumes of powdered drinks were seen following the announcement in the controlled analysis unlike in the uncontrolled analysis. In the uncontrolled analysis absolute and relative differences in the amount of sugar in milk based drinks were significantly different from the pre-implementation counterfactual but not significantly different when examining the impact of the SDIL overall. Additionally the amount of sugar in confectionery was not significantly different from the pre-announcement counterfactual in the uncontrolled ITS analysis unlike in the controlled analysis.

For peer review only

Supplementary Table 5a: Level and trend changes in volume of drinks (ml) and confectionery (g) purchased per household per week (95% CI) in relation to the UK SDIL, March 2014 - March 2019 without toiletries as a control condition (weighted)

	Analysis 1: pre-post announcement (March 2014 – March 2018)		Analysis 2: pre-post implementation (March 2016 – March 2019)		Analysis 3: pre-post announcement & implementation (March 2014 – March 2019)	
	Level Change	Trend Change	Level Change	Trend Change	Level Change	Trend Change
Levy liable drinks						
High tier (≥8g sugar per 100ml)	42.3 (-9.4, 94.0)	-0.07 (-0.9, 0.8)	**-111.2 (-177.1, -45.3)**	-1.2 (-3.0, 0.7)	-68.9 (-152.7, 14.9)	-1.3 (-3.3, 0.8)
Low tier (≥5g - <8g sugar per 100ml)	-2.2 (-24.1, 19.8)	**-0.6 (-1.0, -0.2)**	-18.9 (-45.8, 8.0)	-1.0 (1.9, -0.2)	-21.1 (-55.75, 13.6)	**-1.7 (-2.6, -0.7)**
No levy (<5g sugar per 100ml)	-29.8 (-150.9, 91.3)	**2.0 (0.08, 4.0)**	**378.1 (223.7, 532.5)**	**0.3 (0.5, 2.0)**	**348.3 (152.0, 544.6)**	2.4 (-2.4, 7.2)
>0g to <5g sugar per 100ml	-27.9 (-78.3, 22.5)	**1.3 (0.5, 2.0)**	**98.0 (32.7, 163.3)**	**2.1 (0.3, 3.9)**	70.1 (-12.4, 152.6)	**3.4 (1.4, 5.4)**
0g sugar per 100ml	-2.0 (-75.5, 71.5)	0.8 (-0.4, 2.0)	**278.9 (185.6, 372.2)**	-1.7 (-4.4, 1.0)	**276.9 (158.2, 395.7)**	-0.9 (-3.9, 2.0)
Bottled water	9.9 (-49.1, 68.9)	-0.4 (-1.4, 0.6)	53.5 (-20.8, 127.8)	-2.6 (-4.7, 0.4)	63.4 (-31.5, 158.2)	**-2.9 (-5.3, -0.6)**
Levy exempt drinks						
Alcoholic drinks	-13.3 (-51.0, 24.4)	-0.03 (-0.4, 0.4)	**212.7 (142.5, 282.9)**	**-5.8 (-7.9, -3.8)**	**199.4 (119.8, 279.1)**	**-5.9 (-8.0, -3.8)**
Milk and milk based drinks[1]	26.0 (-35.7, 87.7)	**-1.1 (-2.0, -0.09)**	75.7 (-4.5, 155.9)	0.6 (-1.6, 2.8)	**101.7 (0.52, 202.9)**	-0.5 (-2.9, 2.0)
No added sugar fruit juices	-9.9 (-35.0, 15.2)	0.2 (-0.3, 0.6)	**40.9 (10.1, 71.7)**	-0.9 (-1.9, 0.02)	31.0 (-8.73, 70.7)	-0.8 (-1.8, 0.3)
Drinks sold as powders (g)	-3.4 (-9.01, 2.20)	-0.03 (-0.1, 0.06)	5.66 (-1.6, 12.9)	0.07 (-0.1, 0.3)	2.25 (-6.90, 11.4)	0.04 (-0.2, 0.3)
Confectionery (g)	-20.5 (-90.8, 49.8)	0.7 (-1.3, 1.5)	-70.6 (-155.8, 14.6)	**2.2 (0.7, 3.7)**	-91.1 (-201.5, 19.3)	2.3 (-2.2, 6.8)

Notes.Bold indicates a significant difference at the 95% confidence interval level. [1]Milk comprises drinks in the following categories: semi-skimmed; specific low fat % milk (e.g. 1% fat milk); whole milk; buttermilk; modified milk; other milk; other non-cows milk; rice drink; soya milk. Skimmed milk is excluded from all analysis in this paper due to missing data.

For peer review only

Supplementary Table 5b: Level and trend changes in sugar in drinks (ml) and confectionery (g) purchased per household per week (95% CI) in relation to the UK SDIL, March 2014 - March 2019 without toiletries as a control condition (weighted)

	Analysis 1: pre-post announcement (March 2014 – March 2018)		Analysis 2: pre-post implementation (March 2016 – March 2019)		Analysis 3: pre-post announcement & implementation (March 2014 – March 2019)	
	Level Change	Trend Change	Level Change	Trend Change	Level Change	Trend Change
Levy liable drinks						
High tier (≥8g sugar per 100ml)	4.8 (-1.0, 10.6)	-0.008 (-0.1, 0.09)	**-12.1 (-19.5, -4.8)**	-0.2 (-0.4, 0.06)	-7.3 (-16.6, 2.0)	-0.2 (-0.4, 0.07)
Low tier (≥5g - <8g sugar per 100ml)	-0.2 (-1.7, 1.2)	**-0.4 (-0.5, -0.4)**	-1.2 (-2.9, 0.6)	**-0.07 (-0.1, -0.01)**	-1.4 (-3.7, 0.9)	**-0.5 (-0.6, -0.5)**
No levy (<5g sugar per 100ml)	1.7 (-0.2, 3.3)	**0.07 (0.04, 0.1)**	**5.2 (3.2,7.2)**	**0.08 (0.02, 0.1)**	**6.9 (4.4, 9.5)**	**0.2 (0.09, 0.2)**
>0g to <5g sugar per 100ml	1.7 (-0.2, 3.3)	**0.07 (0.04, 0.1)**	**5.2 (3.2,7.2)**	**0.08 (0.02, 0.1)**	**6.9 (4.4, 9.5)**	**0.2 (0.09, 0.2)**
Levy exempt drinks						
Milk and milk based drinks[1]	2.4 (-0.6, 5.3)	**-0.06 (-0.1, -0.02)**	3.7 (-0.1, 7.5)	0.01 (-0.10, 0.1)	**6.1 (1.3, 10.9)**	-0.05 (-0.2, 0.06)
No added sugar fruit juices	-1.1 (-3.5, 1.4)	0.03 (-0.03, 0.07)	**3.3 (0.3, 6.3)**	**-0.10 (-0.2, -0.01)**	2.3 (-1.6, 6.1)	-0.07 (-0.2, 0.03)
Drinks sold as powders (g)	-1.0 (-3.1, 1.1)	0.01 (-0.02, 0.05)	0.4 (-2.2, 3.0)	-0.01 (-0.1, 0.07)	-0.6 (-3.9, 2.7)	0.001 (-0.09, 0.09)
Confectionery (g)	-11.7 (-51.5, 28.1)	0.05 (-0.8, 0.9)	-42.6 (-90.8, 5.6)	1.3 (-0.4, 3.0)	-54.3 (-116.8, 8.2)	1.3 (-0.5, 3.2)

Notes. Bold indicates a significant difference at the 95% confidence interval level. [1]Milk comprises drinks in the following categories: semi-skimmed; specific low fat % milk (e.g. 1% fat milk); whole milk; buttermilk; modified milk; other milk; other non-cows milk; rice drink; soya milk. Skimmed milk is excluded from all analysis in this paper due to missing data.

For peer review only

Supplementary Table 5c: Absolute and relative changes in volume of drinks (ml) and confectionery (g) purchased per household per week (95% CI) in relation to the UK SDIL, March 2014 - March 2019 without toiletries as a control condition (weighted)

	Analysis 1: pre-post announcement (March 2014 – March 2018)		Analysis 2: pre-post implementation (March 2016 – March 2019)		Analysis 3: pre-post announcement & implementation (March 2014 – March 2019)	
	Absolute change (ml or g)	Relative change (%)	Absolute change (ml or g)	Relative change (%)	Absolute change (ml or g)	Relative change (%)
Levy liable drinks						
High tier (≥8g sugar per 100ml)	34.4 (-4.5, 73.3)	7.3 (-1.0, 15.6)	**-171.6 (-223.1, -120.0)**	**-42.5 (-55.3, -29.7)**	**-140.9 (-192.5, -89.4)**	**-37.8 (-51.6, -24.0)**
Low tier (≥5g - <8g sugar per 100ml)	**-68.3 (-85.7,-50.9)**	**-38.2 (-47.9, -28.4)**	**-71.4 (-94.1, -48.7)**	**-71.5 (-94.2, -48.8)**	**-171.3 (-193.9, -148.6)**	**-85.8 (-97.1, -74.4)**
No levy (<5g sugar per 100ml)	**187.1 (95.7, 278.5)**	**11.5 (5.9, 17.1)**	**395.0 (273.9, 516.0)**	**19.8 (13.7, 25.9)**	**685.5 (564.4, 806.5)**	**40.2 (33.1, 47.3)**
>0g to <5g sugar per 100ml	**106.4 (68.7, 144.2)**	**17.2 (11.1, 23.3)**	**205.9 (155.5, 256.3)**	**25.6 (19.3, 31.8)**	**374.6 (326.0, 426.8)**	**59.3 (51.4, 67.2)**
0g sugar per 100ml	**80.7 (25.1, 136.4)**	**8.0 (2.5, 13.5)**	**191.9 (118.2, 265.5)**	**16.1 (9.9, 22.3)**	**312.0 (238.4, 385.6)**	**29.1 (22.3, 36.0)**
Bottled water	**-31.3 (-76.3, 13.7)**	**-4.4 (-10.6, 1.91)**	**-76.9 (-136.6, -17.2)**	**-9.89 (-17.6, -2.2)**	**-127.8 (-187.6, -68.1)**	**-15.4 (-22.6, -8.2)**
Levy exempt drinks						
Alcoholic drinks	-16.6 (-62.2, 28.9)	1.0 (-3.6, 1.7)	**-84.9 (-155.8, -14.0)**	**-4.81 (-8.8, -0.8)**	**-103.1 (-174.0, -32.2)**	**-5.8 (-9.8, -1.8)**
Milk and milk based drinks[1]	**-85.9 (-39.6, 132.2)**	**-2.3 (-3.6, -1.1)**	**106.4 (44.5, 168.3)**	**3.03 (1.3, 4.8)**	-32.8 (-94.6, 29.1)	-0.9 (-2.6, 0.8)
No added sugar fruit juices	5.9 (-13.9, 25.8)	1.2 (-2.9, 5.4)	-6.56 (-32.7, 19.6)	-1.33 (-6.6, 4.0)	6.95 (-19.2, 33.1)	1.5 (-4.0, 6.9)
Drinks sold as powders (g)	**-6.8 (-11.0, -2.57)**	**-6.8 (-10.9, -2.6)**	**9.3 (3.7, 14.9)**	**10.8 (4.3, 17.4)**	0.89 (-4.7, 6.5)	1.0 (-5.0, 6.9)
Confectionery (g)	-9.5 (-72.7, 53.7)	-2.3 (-17.7, 13.0)	40.6 (-42.1, 123.2)	11.9 (-12.3, 36.0)	36.3 (-46.4, 118.9)	10.5 (-13.4, 34.3)

Notes. Bold indicates a significant difference at the 95% confidence interval level. [1]Milk comprises drinks in the following categories: semi-skimmed; specific low fat % milk (e.g. 1% fat milk); whole milk; buttermilk; modified milk; other milk; other non-cows milk; rice drink; soya milk. Skimmed milk is excluded from all analysis in this paper due to missing data.

For peer review only

Supplementary Table 5d: Absolute and relative changes in sugar in drinks (ml) and confectionery (g) purchased per household per week (95% CI) in relation to the UK SDIL, March 2014 - March 2019 without toiletries as a control condition (weighted)

	Analysis 1: pre-post announcement (March 2014 – March 2018)		Analysis 2: pre-post implementation (March 2016 – March 2019)		Analysis 3: pre-post announcement & implementation (March 2014 – March 2019)	
	Level Change	Trend Change	Level Change	Trend Change	Level Change	Trend Change
Levy liable drinks						
High tier (≥8g sugar per 100ml)	3.9 (-0.4, 8.3)	7.6 (-0.78, 15.9)	**-19.6 (-25.3, -13.8)**	**-43.8 (-56.6, -30.9)**	**-16.1 (-21.8, -10.3)**	**-39.0 (-52.9, -25.0)**
Low tier (≥5g - <8g sugar per 100ml)	**-4.9 (-6.1, -3.8)**	**-40.3 (-49.6, -30.9)**	**-4.7 (-6.2, -3.2)**	**-70.9 (-93.3, -48.4)**	**-11.9 (-13.4, -10.4)**	**-86.0 (-96.8, -75.2)**
No levy (<5g sugar per 100ml)	**6.1 (4.9, 7.3)**	**77.7 (62.6, 92.8)**	**9.3 (7.8, 10.9)**	**52.5 (43.6, 61.4)**	**19.2 (17.6, 20.8)**	**240.9 (221.0, 260.8)**
>0g to <5g sugar per 100ml						
Levy exempt drinks						
Milk and milk based drinks[1]	**-4.2 (-6.4, -2.0)**	**-2.3 (-3.6, -1.1)**	**4.1 (1.1, 7.0)**	**2.4 (0.7, 4.2)**	**-3.2 (-6.5,-0.3)**	**-1.8 (-3.5, -0.1)**
No added sugar fruit juices	**2.6 (0.7, 4.6)**	**5.9 (1.5, 10.2)**	-1.8 (-4.3, 0.8)	-3.7 (-9.1, 1.6)	**2.6 (0.04, 5.2)**	**5.9 (0.08, 11.8)**
Drinks sold as powders (g)	0.4 (-1.2, 2.1)	2.1 (-5.9, 10.0)	-0.3 (-2.4, 1.9)	-1.3 (-12.5, 10.0)	0.9 (-1.3,3.0)	4.7 (-7.2, 16.7)
Confectionery (g)	-5.9 (-42.0, 30.1)	-2.6 (-18.1, 13.0)	23.4 (-24.0, 70.7)	12.1 (-12.4, 36.7)	20.2 (-27.1, 67.6)	10.3 (-13.9, 34.5)

Notes.[†]Trend[2], Bold indicates a significant difference at the 95% confidence interval level. [1]Milk comprises drinks in the following categories: semi-skimmed; specific low fat % milk (e.g. 1% fat milk); whole milk; buttermilk; modified milk; other milk; other non-cows milk; rice drink; soya milk. Skimmed milk is excluded from all analysis in this paper due to missing data.

For peer review only

Changes in soft drinks purchased by British households associated with the UK soft drinks industry levy: a controlled interrupted time series analysis

Journal:	*BMJ Open*
Manuscript ID	bmjopen-2023-077059.R2
Article Type:	Original research
Date Submitted by the Author:	09-Nov-2023

Complete List of Authors:	Rogers , Nina ; MRC Epidemiology Unit<br>Pell, David; MRC Epidemiology Unit<br>Mytton, Oliver; MRC Epidemiology Unit; University College London Great Ormond Street Institute of Child Health<br>Penney, Tarra; York University - Keele Campus<br>Briggs, Adam; University of Oxford, Nuffield Department of Population Health; University of Warwick Warwick Medical School, Institute for Health Policy and Clinical Practice<br>Cummins, Steven; London School of Hygiene & Tropical Medicine, Department of Social and Environmental Health Research<br>Penn-Jones, Catrin; MRC Epidemiology Unit<br>Rayner, Mike; University of Oxford, Nuffield Department of Population Health; NIHR Oxford Biomedical Research Centre<br>Rutter, Harry; University of Bath Department of Social and Policy Sciences<br>Scarborough, Peter; Centre on Population Approaches for Non-Communicable Disease Prevention, Nuffield Department of Population Health; NIHR Oxford Biomedical Research Centre<br>Sharp, Stephen; MRC Epidemiology Unit<br>Smith, Richard ; University of Exeter,<br>White, Martin; MRC Epidemiology Unit<br>Adams, J; MRC Epidemiology Unit

**Primary Subject Heading**:	Public health
Secondary Subject Heading:	Health policy
Keywords:	Obesity, PUBLIC HEALTH, NUTRITION & DIETETICS

SCHOLARONE™
Manuscripts

I, the Submitting Author has the right to grant and does grant on behalf of all authors of the Work (as defined in the below author licence), an exclusive licence and/or a non-exclusive licence for contributions from authors who are: i) UK Crown employees; ii) where BMJ has agreed a CC-BY licence shall apply, and/or iii) in accordance with the terms applicable for US Federal Government officers or employees acting as part of their official duties; on a worldwide, perpetual, irrevocable, royalty-free basis to BMJ Publishing Group Ltd ("BMJ") its licensees and where the relevant Journal is co-owned by BMJ to the co-owners of the Journal, to publish the Work in this journal and any other BMJ products and to exploit all rights, as set out in our licence.

The Submitting Author accepts and understands that any supply made under these terms is made by BMJ to the Submitting Author unless you are acting as an employee on behalf of your employer or a postgraduate student of an affiliated institution which is paying any applicable article publishing charge ("APC") for Open Access articles. Where the Submitting Author wishes to make the Work available on an Open Access basis (and intends to pay the relevant APC), the terms of reuse of such Open Access shall be governed by a Creative Commons licence – details of these licences and which Creative Commons licence will apply to this Work are set out in our licence referred to above.

Other than as permitted in any relevant BMJ Author's Self Archiving Policies, I confirm this Work has not been accepted for publication elsewhere, is not being considered for publication elsewhere and does not duplicate material already published. I confirm all authors consent to publication of this Work and authorise the granting of this licence.

For peer review only

Changes in soft drinks purchased by British households associated with the UK soft drinks industry levy: a controlled interrupted time series analysis

Nina T Rogers, research associate[1]

David Pell, research associate[1(when this work was conducted)]

Oliver Mytton, clinical lecturer[1(when this work was conducted), 9(currently)]

Tarra L Penney, assistant professor[1,2]

Adam Briggs, honorary associate clinical professor[3,4]

Steven Cummins, professor[5]

Catrin Jones, research associate[1]

Mike Rayner, professor[3,6]

Harry Rutter, professor[7]

Peter Scarborough, professor[3,6]

Stephen J Sharp, senior statistician[1]

Richard D Smith, professor[8]

Martin White, professor[1]

Jean Adams, professor [1]

[1]MRC Epidemiology Unit, University of Cambridge School of Clinical Medicine, Institute of Metabolic Science, Cambridge CB2 0QQ, UK

[2]Faculty of Health, School of Global Health, York University, Toronto, Canada

[3]Nuffield Department of Population Health, University of Oxford, Oxford, UK

[4]Warwick Medical School, University of Warwick, Division of Health Sciences, Coventry, UK

[5]Population Health Innovation Lab, Department of Public Health, Environment and Society, London School of Hygiene and Tropical Medicine, London, UK

[6]National Institute of Health Research Oxford Biomedical Research Centre, Oxford University Hospitals NHS Foundation Trust, Headington, Oxford, UK

[7]Department of Social and Policy Sciences, University of Bath, Bath, UK

[8]College of Medicine and Health, University of Exeter, Exeter, UK

[9]Great Ormond Street Institute of Child Health, University College London, London, UK

Correspondence to:
Jean Adams
jma79@medschl.cam.ac.uk
(@jeanmadams on Twitter)

For peer review only - http://bmjopen.bmj.com/site/about/guidelines.xhtml

Abstract

Objective To determine changes in household purchases of drinks one year after implementation of UK soft drinks industry levy (SDIL).
Design Controlled interrupted time series.
Participants Households reporting their purchasing to a market research company (average weekly n=22,091), March 2014 to March 2019.
Intervention A two tiered tax levied on soft drinks manufacturers, announced in March 2016 and implemented in April 2018. Drinks with ≥8g sugar/100mL (high tier) are taxed at £0.24/L, drinks with ≥5 to <8g sugar/100mL (low tier) are taxed at £0.18/L.
Main outcome measures Absolute and relative differences in the volume of, and amount of sugar in, soft drinks categories, all soft drinks combined, alcohol, and confectionery purchased per household per week one year after implementation.
Results In March 2019, compared with the counterfactual, purchased volume of high tier drinks decreased by 140.8mL (95% confidence interval 104.3-177.3mL) per household per week, equivalent to 37.8% (28.0%-47.6%), and sugar purchased in these drinks decreased by 16.2g (13.5-18.8g), or 42.6% (35.6-49.6%). Purchases of low tier drinks decreased by 170.5mL (154.5-186.5mL) or 85.8% (77.8-93.9%), with an 11.5g (9.1-13.9g) reduction in sugar in these drinks, equivalent to 87.8% (69.2-106.4%). When all soft drinks were combined irrespective of levy tier or eligibility, the volume of drinks purchased increased by 188.8mL (30.7-346.9mL) per household per week, or 2.6% (0.4-4.7%), but sugar decreased by 8.0g (2.4-13.6g), or 2.7% (0.8-4.5%). Purchases of confectionery and alcoholic drinks did not increase.
Conclusions Compared with trends before the SDIL was announced, one year after implementation, volume of all soft drinks purchased combined increased by 189mL, or 2.6% per household per week. The amount of sugar in those drinks was 8g, or 2.7%, lower per household per week. Further studies should determine whether and how apparently small effect sizes translate into health outcomes.
Study registration ISRCTN18042742.

Strengths and limitations of this study

- We used a large, nationally representative dataset, included a control category, and explored changes in two potential substitute categories (alcohol and confectionery).
- We only included purchases brought into homes.
- We did not assess changes in other categories beyond soft drinks, alcohol, and confectionery.
- The estimate of effect size in interrupted time series analyses is based on a modelled counterfactual that might be inaccurate.
- Attribution of effects in interrupted time series analyses is vulnerable to time varying confounding such as co-interventions.

For peer review only - http://bmjopen.bmj.com/site/about/guidelines.xhtml

INTRODUCTION

High consumption of sugar sweetened beverages (SSBs) is associated with increased risk of dental caries, obesity, type 2 diabetes, and cardiovascular disease.[1-3] The World Health Organization recommends the use of SSB taxes to reduce consumption.[4] A systematic review of studies published to June 2018 suggests that SSB taxes lead to decreases in the sales, purchasing and consumption of taxed drinks.[5] More recent findings support this conclusion.[6-10] Although price is one important mediator of these changes,[11-16] other potential mechanisms include reformulation of products to reduce sugar concentration, smaller portion sizes, and increases in the perception of SSBs being harmful to health associated with them being grouped with other taxed products such as alcohol and tobacco.[17] Furthermore, any public health benefits of reduced SSB consumption associated with SSB taxes might be negated by increased consumption of substitutes such as confectionery and alcohol.[18-20]

The UK soft drinks industry levy (SDIL) was one of the first taxes on SSBs explicitly designed to incentivise manufacturers of SSBs to reduce sugar content.[21 22] This is reflected in three design features. Firstly, the SDIL is levied on manufacturers, importers, and bottlers rather than on consumers. Secondly, the levy includes two tiers: £0.24/L for drinks containing ≥8 g total sugar per 100 mL, and £0.18/L for drinks containing ≥5 g and <8 g total sugar per 100 mL. Thirdly, the SDIL was intentionally announced in 2016, two years before implementation in 2018, to allow manufacturers time to adjust. The SDIL also provides exemptions (Box 1).[23]

Box 1. Glossary of terms

Soft drinks industry levy (SDIL)—a tiered tax on manufacturers of sugar sweetened beverages

Levy exempt drinks—drinks exempt from the SDIL irrespective of sugar content; that is, drinks containing >75% milk, drinks containing >1.2% alcohol, and drinks sold as alcohol replacements, drinks sold as powders, 100% fruit juices, and drinks sold by manufacturers selling less than one million litres of drinks not exempt for other reasons each year

High tier drinks—drinks that are not levy exempt and contain ≥8 g of sugar per 100 mL

Low tier drinks—drinks that are not levy exempt and contain ≥5 g to <8 g of sugar per 100 mL

No levy drinks—drinks that are not levy exempt but contain <5 g of sugar per 100 mL; we subdivided this category into drinks containing >0 g to <5 g of sugar per 100 mL, drinks containing 0 g of sugar per 100 mL. Bottled water was considered separately.

Levy liable drinks—drinks that are not levy exempt drinks; that is, the sum of high tier drinks, low tier drinks, and no levy drinks

Soft drinks—any drink not containing alcohol

Confectionery—products in the sugar confectionery and chocolate confectionery categories

For peer review only - http://bmjopen.bmj.com/site/about/guidelines.xhtml

Toiletries—products in the shampoo, hair conditioner, and liquid soap categories

Two before and after analyses have shown reductions of around 30% in sales weighted sugar concentration of levy eligible drinks in the UK from before the announcement of the SDIL on 16 March 2016 to after implementation on 6 April 2018.[24 25] However, background trends in purchases of sugary drinks are not stable, with decreases reported over several years.[26] This makes it difficult to attribute before and after decreases in sugary drinks purchases to the SDIL. An interrupted time series analysis found that the announcement and implementation of the SDIL were together associated with a 34 percentage point reduction in the proportion of levy liable drinks with >5 g total sugar per 100 mL, indicating substantial reformulation of the market.[15] Changes in prices across the UK soft drink market were also reported, although it was difficult to discern clear patterns in these, with some levied categories increasing and others decreasing in price. In a controlled interrupted time series analysis including data up to the point of SDIL implementation, we found that the SDIL announcement was associated with changes in both the volume of, and sugar purchased in, drinks in many categories.[27] Overall we found no change in total volume of purchases of all soft drinks combined, but a small increase in sugar purchased from soft drinks of 5.3g per household per week, or 1.7%.

In this paper, our aim was to determine whether household purchases of drinks and confectionery had changed one year after implementation of the SDIL.

METHODS

Here we extend our previous analyses[27] to study changes in the volume of, and amount of sugar in, household purchases of drinks in each levy tier, exempt drinks categories (including alcoholic drinks), and confectionery from two years before the announcement of the SDIL to one year after its implementation (March 2014 to March 2019). As before, we used controlled interrupted time series methods, with toiletries included as a control category.[27] We compared observed changes associated with the announcement and implementation of the SDIL to the counterfactual scenarios in which the announcement and implementation did not take place. Including a full two years of data before the announcement enables us to estimate pre-intervention trends and project these forward as counterfactual scenarios. The protocol is published elsewhere[28] and the study was registered. This study is reported in accordance with the strengthening the reporting of observational studies in epidemiology (STROBE) guideline (see Supplementary material A).

For peer review only - http://bmjopen.bmj.com/site/about/guidelines.xhtml
For peer review only

Data source

We used data from a panel of households reporting their purchasing on a weekly basis to a market research company (Kantar Worldpanel; KWP). Participating households are asked to record all food and drink purchases brought into the home (including those ordered online and delivered) through barcodes scanners and manual report. Purchasing information is uploaded weekly, where it is linked to nutritional data collected by KWP field workers on a rolling basis. Households record their personal characteristics and receive gift vouchers worth about £100 ($122; €112) annually—equivalent to 0.3% of median UK annual household income after tax in 2019 (£29 600).[29]

KWP samples households from across Great Britain (GB) using proprietary methods, aiming to achieve a sample that is demographically representative of GB households. Data excludes households that record fewer than six purchases weekly along with those whose adjusted weekly spend is lower than an undisclosed minimum. KWP applies proprietary weights to purchases to adjust for these exclusions and maintain the representativeness of the panel. We used these weights throughout.

The main data cleaning that occurred before analysis involved assigning products and product groups in the KWP dataset to SDIL relevant groups. This was done based on KWP assigned product groups, product names, and nutritional content. In previous work we found some evidence of error, but not bias, in the sugar concentration reported by KWP compared with information provided on manufacturers' websites.[27]

Product categories: drinks, confectionery, and toiletries

Purchased drinks that were levy liable were divided into high tier, low tier, or no levy based on sugar content (see box 1 for definitions). No levy drinks were additionally disaggregated, as described in box 1.

As the SDIL might have led to substitution to other drinks categories, we also examined purchasing of levy exempt drinks in several categories: milk based drinks (comprising milk, milk alternatives such as soya drinks, and yoghurt based juices and drinks), alcoholic drinks (comprising both alcoholic and alcohol replacement drinks), no added sugar fruit juices, and drinks sold as powder (eg, tea, coffee, hot chocolate). Other exempt categories (infant formulas and drinks sold for medical purposes) were excluded.

We also hypothesised that the SDIL might lead to substitution from sugary drinks to other high sugar categories. To investigate this, we used sugar and chocolate confectionery purchases (referred to as confectionery).

For peer review only

For peer review only - http://bmjopen.bmj.com/site/about/guidelines.xhtml

Control group

To control for background trends in household purchases we used purchases of shampoo, hair conditioner, and liquid soap (ie, toiletries). Toiletries meet the proposed criteria for a controlled interrupted time series: they are robust to seasonality and may have similar purchase volumes by households regardless of socioeconomic position or other potential confounders.[30]

Outcome measures

Most evaluations of SSB taxes focus on volume of drinks purchased. However, the SDIL's focus on reformulation makes the sugar purchased in drinks of additional public health interest. Thus, the outcome measures of interest were mean volume purchased per household per week in each of the drink categories and grams per household per week of confectionery; and mean sugar purchased per household per week from each of the drink categories and confectionery. Data were aggregated at the weekly level and analysed as a time series.

Overall analysis strategy

Previous evidence indicates that reformulation occurred after the announcement of the SDIL and price changes after implementation.[15] As such, we hypothesised the SDIL might act as two linked interventions: the announcement on 16 March 2016 and implementation on 6 April 2018.[17] Thus, our analysis strategy involved three separate comparisons that isolate the announcement and implementation of the SDIL and then examine the combined effect (Figure 1). In the first analysis, we isolated the announcement of the SDIL. Here we compared anticipatory effects on purchasing two years after the announcement to the counterfactual estimated from purchasing in the two years before the announcement. This replicates and updates our previous analysis[27] as we anticipate that the stabilising effect of including additional post-announcement data likely reduces error. In the second analysis, we isolated the implementation of the SDIL. Here we compared purchasing one year after implementation to the counterfactual estimated from purchasing in the four years before implementation. In the third analysis, we considered both the announcement and the implementation and we compared purchasing one year after implementation (ie three years after announcement) to the counterfactual estimated from purchasing in the two years before the announcement.

Throughout, we used the proprietary weights provided by KWP.

Primary analysis: category specific analyses

For peer review only - http://bmjopen.bmj.com/site/about/guidelines.xhtml
For peer review only

For each of the three analyses we developed separate controlled interrupted time series models for volume and sugar purchased from each levy liable and levy exempt drinks category and confectionery (Figure 1). Supplementary material B provides the full model specification.

We present absolute and relative differences between observed purchasing and counterfactual scenarios in the final week of each observation period, with standard errors used to calculate 95% confidence intervals for the relative difference obtained using the delta method.[31]

Secondary analysis: all soft drinks categories combined, irrespective of levy eligibility

Levy exempt drinks include drinks that might contain comparable amounts of sugar to levy liable products. To examine the extent to which the SDIL impacted on the purchased volume of, and amount of sugar in, soft drinks, regardless of SDIL liability, we carried out controlled interrupted time series analysis, combining purchases of all soft drinks irrespective of sugar content (ie, high tier, low tier, no levy, milk and milk based drinks, no added sugar fruit juice, and drinks sold as powders), levy liable drinks irrespective of sugar content (ie, high tier, low tier, and no levy drinks), and according to sugar content based on levy tiers irrespective of levy eligibility (ie, all soft drinks with ≥8 g of sugar per 100 mL, all soft drinks with ≥5 g to <8 g of sugar per 100 mL, and all soft drinks with <5 g of sugar per 100 mL).

Sensitivity analysis: excluding small manufacturers

The SDIL exempts drinks from manufacturers and producers who sell less than one million litres of levy liable drinks annually. As we were unable to obtain a list of exempt manufacturers, our main analyses include all manufacturers. We conducted sensitivity analyses to examine the effect of excluding manufacturers who we estimated to be small. The total purchase volume was summed by manufacturer by year across the five years in the KWP dataset, and a mean purchase volume per year for each manufacturer was calculated. In the first sensitivity analysis, we excluded manufacturers with a mean of less than one million litres purchased per year. Acknowledging KWP data excludes purchases not brought home, we repeated these analyses excluding manufacturers with mean annual purchased volumes of <0.5 million litres in KWP. We were unable to access accurate estimates of the proportion of all drinks purchases brought home. This value reflects an arbitrary, but we think conservative, estimate of the minimum proportion of drinks brought home.

Sensitivity analysis: interrupted time series without a control category

For peer review only - http://bmjopen.bmj.com/site/about/guidelines.xhtml

Toiletries were chosen as a control condition a priori to account for background trends in household purchases. It is, however, possible that a more appropriate control exists. As we only have access to data on purchasing of the categories described here (confectionery, drinks, toiletries), we were not able to examine alternative potential control categories. To examine the effect of the decision to use toiletries as the control category, we performed an additional sensitivity analysis with no control condition.

Changes to the protocol

We made several changes to the published protocol.[28] KWP provided additional data that allowed us to increase the precision of our estimates. Specifically, we were able to increase the pre-announcement study period from 104 to 107 weeks and reduce the unit of analysis from purchases every four weeks to purchases every week. We originally intended to include purchases not brought home. We excluded these purchases, however, as these data were not available before mid-2015, meaning that robust pre-announcement trends could not be estimated. Although we originally intended to combine all no levy drinks, we present these disaggregated into those with >0 g and <5 g of sugar per 100 mL and 0 g of sugar per 100 mL, as well as bottled water, as trends for these different categories are noticeably different. Our original intention to explore potential disparities across socioeconomic groups will be pursued in future work.

Patient and public involvement

The steering group for the wider SDIL evaluation includes two lay members and meets twice a year. Patients and the public were not involved in developing the research question, the outcome measures, the design, or the conduct of the work reported here. The steering group has regularly contributed ideas for routes to dissemination.

Correction of Pell et al (2021)

This paper is a corrected version of Pell et al (2021),[32], now retracted, which was originally published in the *BMJ*. The analysis presented in the original Pell et al (2021) paper included an incorrect weighting variable. This variable was incorrectly calculated as the inverse of what it should have been. The variable was also redundant to the analysis as it replicated a component of a second weighting variable also included (the "proprietary weights provided by KWP" mentioned above). The current corrected version replicates the original analysis without this redundant and incorrectly calculated weighting variable. The second, correct, weighting variable (the "proprietary weights provided by KWP" mentioned above) remains included. The authors identified the error themselves and alerted the journal and readers.[33]

For peer review only - http://bmjopen.bmj.com/site/about/guidelines.xhtml

RESULTS

About 31 million purchases of drinks, confectionery, and toiletries from March 2014 to March 2019 were included from a mean of 22 091 households each week. The characteristics of included households remained consistent over the study period, and after weighting they largely reflected households in 2014-19 in the UK (see Supplementary table 1 in Supplementary material C).

Table 1 summarises households' weekly purchased volumes of, and amounts of sugar in, drinks and other categories over the study period. Substantial reductions in volume of, and sugar in, purchases of SDIL liable drinks were observed in the high and low tiers over time. These reductions were accompanied by a smaller increase in volume of no levy drinks purchased, but proportionally much greater increases in sugar purchased in these drinks.

Primary analysis: category specific results

Results of the controlled interrupted time series analyses of purchased volume of, and sugar in, levy liable drinks and confectionery are shown in Figure 2 (volume) and Figure 3 (sugar). Absolute and relative changes are summarised in Tables 2 and 3. Supplementary tables 2a and b in Supplementary material D show level and trend changes from these models. Supplementary figures 1a and b in Supplementary material D show similar figures and data for subcategories of no levy drinks, bottled water and exempt categories.

High tier drinks

The trend in purchased volume of, and sugar in, high tier drinks continued downwards throughout the study period. The announcement of the SDIL was associated with an increase in purchased volume of (34.7ml (95% confidence intervals 8.1 to 61.4ml, or 7.3% (1.7 to 12.9%)), and sugar in (5.5g (3.8 to 7.2), or 10.8% (7.4 to 14.1%)), these drinks. In contrast, the implementation of the SDIL was associated with a reduction in purchased volume of, and sugar in, these drinks. The volume of high tier drinks purchased was 171.6 mL (135.1 to 208.1mL) per household per week, or 42.5% (33.5% to 51.6%), lower in March 2019 compared with the counterfactual estimated from pre-implementation trends. The reductions associated with implementation outweighed the increases associated with announcement, such that the intervention as a whole was associated with a decrease in purchased volume of 140.8ml (104.3 to 177.3ml) per household per week or 37.8% (28.0 to 47.6%) and sugar of 16.2 g (13.5 to 18.8g) per household per week or 42.6% (35.6% to 49.6%) from these drinks.

Low tier drinks

For peer review only - http://bmjopen.bmj.com/site/about/guidelines.xhtml

Purchased volume of, and sugar in, low tier drinks gradually increased before the announcement of SDIL. The announcement was associated with a reversal of this trend. There were reductions in purchased volume of, and sugar in, low tier drinks associated with announcement, implementation and the whole intervention. Compared with the counterfactual estimated from pre-announcement trends, in March 2019 the volume of purchased low tier drinks per household per week decreased by 170.5 mL (154.5 to 186.5 mL), or 85.8% (77.8 to 93.9%); and sugar purchased in these drinks decreased by 11.5 g (9.1 to 13.9g) per household per week, or 87.8% (69.2 to 106.4%).

No levy drinks

Before the announcement of the SDIL there was a gradual upward trend in volume of purchased no levy drinks but a gradual downward trend in purchased sugar. Announcement, implementation and the whole intervention were associated with increases in volume of no levy drinks purchased as well as sugar purchased from those drinks. Overall, purchased volume of no levy drinks in March 2019 was 685.5 mL (599.8 to 771.1mL) higher, equivalent to 40.2% (35.2% to 45.2%) increase compared with the counterfactual of pre-announcement trends. Equivalent figures for sugar purchased from no levy drinks were a 19.2g (16.7 to 21.6g) per household per week, equivalent to 242.8% (211.9 to 273.7%), increase.

The implementation and the announcement of the SDIL were associated with increases in volume of purchased drinks with no sugar and with >0 to <5 g total sugar per 100 mL, and increases of sugar in drinks with >0 to<5g sugar per 100ml.

Bottled water

The implementation, but not the announcement, of the SDIL were associated with significant decreases in bottled water purchased which led to an overall decrease in volume of bottled water purchased as a result of the whole intervention of 130.5ml (88.8 to 174.1ml) per household per week, or 15.7% (10.4 to 20.9%).

Levy exempt drinks and confectionery

Overall, the combined announcement and implementation of the SDIL were associated with decreases in purchased volume of alcoholic and milk and milk-based drinks, but no change in sugar purchased from levy exempt categories or from confectionery. Compared with the counterfactual of pre-announcement trends, in March 2019 volume of alcoholic drinks purchased decreased by 103.1ml (53.0 to 153.3ml) per household per week, equivalent to a

For peer review only - http://bmjopen.bmj.com/site/about/guidelines.xhtml

5.8% (3.0 to 8.6%) reduction; and volume of milk and milk based drinks purchased decreased by 132.8ml (51.7 to 213.9ml), equivalent to a 3.6% (1.4 to 5.7%) reduction.

Secondary analysis: all soft drinks categories combined

Supplementary table 3a in Supplementary material E and Supplementary figure 2a and 2b in Supplementary material F summarise the results of the controlled interrupted time series analyses of the associated effects of the SDIL on purchased volume of, and sugar from, all soft drinks categories combined, irrespective of levy eligibility. Supplementary table 3b in Supplementary material E summarises absolute and relative changes in volume of, and sugar in, all soft drinks and confectionery purchased. Summary figures are also provided in Table 2 and Table 3.

Overall, compared with the counterfactual estimated from pre-announcement trends, a small increase was observed in volume of all soft drinks purchased in March 2019 of 188.8ml (30.7 to 346.9ml) per household per week, equivalent to a 2.6% (0.4 to 4.7%) increase. A reduction was, however, found in sugar purchased in all soft drinks (including exempt drinks) combined of 8.0g per household per week (2.4 to 13.6g), equivalent to 2.7% (0.8 to 4.5%).

Sensitivity analyses

Excluding manufacturers of levy liable products with less than one million and less than 500 000 litres of purchased drinks annually in our dataset was associated with small changes in the magnitude of estimated coefficients, but with no change in the direction or statistical significance of absolute or relative changes in volume of, or sugar in, drinks (Supplementary tables 4a to b in Supplementary material F).

In general, removing the control category led to minor changes in effect estimates but wider confidence intervals (see Supplementary tables 5a to d in Supplementary material G).

DISCUSSION

Taking account of pre-existing pre-announcement trends, this study found that one year after implementation of the SDIL, sugar purchased from all soft drinks combined that were taken home decreased by 8.0 g per household per week (or 2.7%), whilst volume increased by 188.8 mL per household per week (or 2.6%). Assuming a mean UK household size of 2.4 people,[34] this is equivalent to a reduction in sugar from SSBs of 3.3 g per person per week and an increase in volume of 79 mL per person per week, or equivalent to the replacement of 66 mL of a drink with 5 g sugar per 100 mL per person per week with 145 mL of a sugar-free alternative. A modelling study conducted before implementation of the SDIL found that if the levy achieved reformulation it could be expected to lead to a decrease in sugar consumption

For peer review only - http://bmjopen.bmj.com/site/about/guidelines.xhtml

For peer review only

from SSBs (from all sources, not just for consumption at home) of 7-38 g per person per week and that this would be associated with a reduction in the number of obese individuals in the UK of 0.2-0.9% and a reduction in incidence cases of type 2 diabetes of -2.0 to 31.1 per 1000 person years.[35] The reduction in sugar from SSBs we report one year after implementation of the SDIL is around half of these lower effect estimates.

Strengths and weaknesses of this study

In this study we used a large, nationally representative dataset, included a control category, and explored changes in two potential substitute categories (alcohol and confectionery).

We only included purchases brought into homes. Although KWP also collects data on other purchases, this smaller panel was established in mid-2015 and so was unsuitable for our analyses because robust pre-announcement trends could not be estimated. KWP data are collected at the household level and do not take account of waste or differential sharing within households. Nevertheless, the data provide a reasonable estimate of consumption.[36] We did not assess changes in other categories beyond soft drinks, alcohol, and confectionery.

The estimate of effect size in interrupted time series analyses is based on a modelled counterfactual that might be inaccurate. For example, the strong downward trend in higher tier drinks before the announcement of SDIL might not have continued. Attribution of effects in interrupted time series analyses is vulnerable to time varying confounding including co-interventions. The SDIL is part of a wider sugar reduction strategy, although this has been found to have achieved minimal changes beyond those attributable to the SDIL.[25]

The personal characteristics of the panel remained similar over the study period, and proprietary weightings were used to account for non-consumers and to adjust for variations in panel composition. Households participating in KWP are slightly more likely to be from lower social grades and to have no qualifications compared with UK households generally. This might reflect the relative value placed on the small rewards for participation by different households and could limit the generalisability of our findings. If households from lower socioeconomic backgrounds are more likely to change purchasing as a result of the SDIL, then we could have marginally overestimated the effect of the SDIL. However, while we previously found that the price of soft drinks in the UK did change after implementation of the SDIL, no clear pattern was found, with the price of some groups of drinks increasing and others decreasing.[15] We previously found no systematic differences between the sugar content of drinks reported in KWP data and contemporaneous values listed on supermarket websites.[27]

For peer review only - http://bmjopen.bmj.com/site/about/guidelines.xhtml

Comparison with other work

Our finding that the SDIL was associated with a reduction in purchased sugar from all soft drinks combined is consistent with previous analyses that focused on the SDIL.[24 25] Although our estimate of the reduction in sugar consumption from all soft drinks combined associated with the levy (2.7%) is less than that estimated by others (29%)[25] this previous estimate did not take account of pre-existing trends which we have demonstrated were on a steep downward trajectory for high tier drinks.

We found that the reduction in purchased sugar from all soft drinks combined alongside a 2.6% increase in volume of all soft drinks purchased. This is consistent with previously reported reductions in the sugar concentration of drinks associated with the SDIL.[15] However, the estimated effect size is below the range of reformulation scenarios modelled before implementation (ie, a reduction of 17 to 90 g of sugar per household per week).[35] This difference may be, at least partly, attributable to our focus on drinks taken home versus the modelling study's focus on all drinks. Furthermore, the modelling was based on pre-implementation best and worst case scenarios of changes in formulation, price and SSB market share whilst our analysis was based on observed data.

Evaluations of other SSB taxes have revealed a consistent trend of reductions in purchasing of taxed drinks and no change in purchasing of untaxed drinks.[5] We found similar with both volume of, and sugar in, high and low tier drinks decreasing overall. However, these reductions in volume of taxed drinks were more than offset by increases in volume of no levy drinks purchased. Despite some increases in sugar purchased in no levy drinks, these did not offset decreases in sugar purchased from high and low tier drinks. The SDIL is relatively unique in being explicitly designed to encourage reformulation and there is evidence that substantial reformulation occurred.[15] We are not able to determine from our findings whether the changes we report are due to changes in consumer preference, formulation, or both.

Meaning of the study and implications for policymakers

Our main findings are that the SDIL was associated with a reduction in purchased sugar from all soft drinks combined with evidence of an increase in the total volume of all soft drinks purchased. Given the reformulation associated with the SDIL already documented,[15] it is probable that the changes we report were driven by reductions in the sugar concentration of available drinks, alongside consumers switching to and, indeed increasing consumption of, lower sugar alternatives. Despite the overall reduction we found in sugar purchased in soft drinks, the average amount of sugar purchased in drinks in the no levy group paradoxically

For peer review only

For peer review only - http://bmjopen.bmj.com/site/about/guidelines.xhtml

increased after implementation of the SDIL, with many drinks that previously had sugar concentrations above the levy threshold now having them just below the threshold. This seems to reflect manufacturers reformulating to target thresholds. Lowering the threshold sugar concentration at which drinks become eligible for the SDIL even further could potentially lead to greater overall reductions in sugar concentrations and sugar purchased in soft drinks, as could extension of the SDIL to milk based drinks and other currently exempt categories that sometimes contain high levels of sugar.

The SDIL has also been found to have had no long term negative effects on the share value or turnover of UK soft drinks companies,[37 38] suggesting that, contrary to industry predictions, public health can gain without negatively affecting the soft drinks sector.

We note a marked pre-implementation decline in purchasing of high levy tier drinks. It is possible that this was, at least in part, driven by concern from industry about a possible SSB tax, leading to some pre-announcement reformulation; alongside growing consumer awareness of, and concerns about, the health impacts of SSBs.[39] Although it is uncertain if this trend would have continued in the absence of the SDIL, it is likely to be beneficial for health.

Reassuringly, we did not observe any increase in purchasing of potentially harmful substitutes (ie, alcohol and confectionery) associated with the SDIL, which could have partially or wholly offset any public health gains from the SDIL. However, we did not study the SDIL's effect on purchases of other food groups or on overall diet.

In contrast with previous findings from Mexico and Barbados,[6 40] we did not observe an increase in purchased bottled water associated with the SDIL. Indeed purchases of bottled water decreased significantly during the study period (by 130.5 mL per household per week, or 15.7%). Although we cannot rule out an effect of the SDIL on bottled water purchases, we cannot think of a plausible pathway through which it achieved reductions in purchased bottled water. Instead, this reduction might be due to coincident increases in concern about single use plastic that have been attributed, in the UK, to the broadcast of the nature documentary series *Blue Planet 2* in October-December 2017.[41] It is not clear if a similar "Blue Planet effect" has occurred in other countries. Unlike for many other soft drinks, a like-for-like substitution is available for bottled water in countries such as the UK—that is, filling reusable water bottles with tap water. Several UK retailers have reported substantial growth in sales of reusable water bottles since 2018.[42] Given that tap water is freely available, it is difficult to study changes in its consumption directly.

For peer review only - http://bmjopen.bmj.com/site/about/guidelines.xhtml

Unanswered questions and future research

Future work should seek to understand the longer term effects of the SDIL on purchasing and consumption of soft drinks as well as total diet, and health outcomes. Differential effects of the SDIL on all these outcomes across population groups (eg, by socioeconomic position and in households with vs without children) should also be explored to determine whether the SDIL contributes to narrowing inequalities in health. The changes in purchasing we report here could be used as an input to health impact modelling to estimate the effect of changes on population prevalence of obesity, diabetes, and other chronic conditions to determine how apparently small changes in consumption at the household level translate into health benefits. It is likely that the reformulation that has occurred in response to the SDIL[15] reflects substantial increases in the use of artificial sweeteners in the UK soft drinks market. Given public mistrust of artificial sweeteners[39] and the recent advice from WHO that artificial sweeteners should not be used to reduce the risk of non-communicable diseases,[43] the effect of the SDIL on consumption of these should also be explored.

CONCLUSION

One year after implementation of the SDIL, purchased sugar in all soft drinks combined decreased by around 8 g per household per week (or 2.7%) with an increase in the volume of purchased soft drinks of 189 mL per household per week (or 2.6%). Further studies are required to determine whether and how these apparently small effect sizes translate into health outcomes.

For peer review only

For peer review only - http://bmjopen.bmj.com/site/about/guidelines.xhtml

Contributors

DP, MW, SC, MR, RS, HR, JA, PS, OM, AB, RDS and TLP conceived the study and defined the analytical strategy. DP, NR, JA, OM, TLP, and SJS performed statistical analyses. DP, NR, JA, OM, TLP, CJ and SJS provided preliminary interpretation of findings. DP, NR, JA, OM, and TLP drafted the manuscript. All authors critically interpreted the results, revised the manuscript, provided relevant intellectual input, and read and approved the final manuscript. NR and JA had primary responsibility for the final content. JA will act as guarantor. The corresponding author attests that all listed authors meet authorship criteria and that no others meeting the criteria have been omitted.

Funding

NR, OM, MW, and JA were funded by the Centre for Diet and Activity Research (CEDAR), a UK Clinical Research Collaboration (UKCRC) Public Health Research Centre of Excellence Funding from the British Heart Foundation, Cancer Research UK, Economic and Social Research Council, Medical Research Council, National Institute for Health Research (NIHR), and Wellcome Trust, under the auspices of the UKCRC. This project was funded by the NIHR Public Health Research programme (grant numbers 16/49/01 and 16/130/01), and these grants supported purchase of the data used in this study. The work was also supported by the Medical Research Council (grant numbers MC_UU_12015/6 and MC_UU_00006/7). The views expressed are those of the authors and not necessarily those of the National Health Service, the NIHR, or the Department of Health and Social Care, UK. The funders had no role in study design, data collection and analysis, decision to publish, or preparation of the manuscript. NR and JA had full access to all the data in the study. All authors had final responsibility for the decision to submit for publication. The lead author affirms that the manuscript is an honest, accurate, and transparent account of the study being reported; that no important aspects of the study have been omitted; and that any discrepancies from the study as planned (and, if relevant, registered) have been explained.

Competing interests

All authors have completed the ICMJE uniform disclosure form at www.icmje.org/coi_disclosure.pdf (available on request from the corresponding author) and declare: MW was director of the National Institute for Health Research Public Health Research Funding programme when this work was conducted, and OM was on secondment at the UK Department of Health and Social Care when this work was conducted and previously worked with Public Health England; no support from any organisation for the submitted work other than that described above; no financial relationships with any organisations that might have an interest in the submitted work in the previous three years; and no other relationships or activities that could appear to have influenced the submitted work.

Ethical approval

Not required for secondary data analysis of anonymised data.

Data availability statement

The statistical code for the analyses are available from https://github.com/MRC-Epid/SDILEvaluation. Kantar Worldpanel data are not publicly available but can be purchased from Kantar Worldpanel (http://www.kantarworldpanel.com). The authors are not legally permitted to share the data used for this study but interested parties can contact Kantar WorldPanel representative Sean Cannon (Sean.Cannon@kantar.com) to inquire about accessing this proprietary data.

Open Access

For peer review only - http://bmjopen.bmj.com/site/about/guidelines.xhtml

This is an Open Access article distributed in accordance with the terms of the Creative Commons Attribution (CC BY 4.0) license, which permits others to distribute, remix, adapt and build upon this work, for commercial use, provided the original work is properly cited. See: http://creativecommons.org/licenses/by/4.0/.

For peer review only

For peer review only - http://bmjopen.bmj.com/site/about/guidelines.xhtml

References

1. Imamura F, O'Connor L, Ye Z, et al. Consumption of sugar sweetened beverages, artificially sweetened beverages, and fruit juice and incidence of type 2 diabetes: Systematic review, meta-analysis, and estimation of population attributable fraction. BMJ 2015;**351** doi: 10.1136/bmj.h3576.

2. Moynihan PJ, Kelly SAM. Effect on caries of restricting sugars intake: Systematic review to inform WHO guidelines. Journal of Dental Research 2014;**93**(1):8-18 doi: 10.1177/0022034513508954.

3. Xi B, Huang Y, Reilly KH, et al. Sugar-sweetened beverages and risk of hypertension and CVD: A dose-response meta-analysis. British Journal of Nutrition 2015;**113**(5):709-17 doi: 10.1017/S0007114514004383.

4. World Health Organization. Best buys' and Other Recommended Interventions for the Prevention and Control of Noncommunicable Diseases. Geneva: WHO, 2017.

5. Teng AM, Jones AC, Mizdrak A, Signal L, Genç M, Wilson N. Impact of sugar-sweetened beverage taxes on purchases and dietary intake: Systematic review and meta-analysis. Obesity Reviews 2019;**20**(9):1187-204 doi: 10.1111/obr.12868.

6. Alvarado M, Unwin N, Sharp SJ, et al. Assessing the impact of the Barbados sugar-sweetened beverage tax on beverage sales: An observational study. International Journal of Behavioral Nutrition and Physical Activity 2019;**16**(1) doi: 10.1186/s12966-019-0776-7.

7. Cawley J, Frisvold D, Hill A, Jones D. The impact of the Philadelphia beverage tax on purchases and consumption by adults and children. Journal of Health Economics 2019;**67** doi: 10.1016/j.jhealeco.2019.102225.

8. Lee MM, Falbe J, Schillinger D, Basu S, McCulloch CE, Madsen KA. Sugar-sweetened beverage consumption 3 years after the Berkeley, California, sugar-sweetened beverage tax. American Journal of Public Health 2019;**109**(4):637-39 doi: 10.2105/AJPH.2019.304971.

9. Roberto CA, Lawman HG, Levasseur MT, et al. Association of a Beverage Tax on Sugar-Sweetened and Artificially Sweetened Beverages with Changes in Beverage Prices and Sales at Chain Retailers in a Large Urban Setting. JAMA - Journal of the American Medical Association 2019;**321**(18):1799-810 doi: 10.1001/jama.2019.4249.

10. Royo-Bordonada MA, Fernández-Escobar C, Simón L, Sanz-Barbero B, Padilla J. Impact of an excise tax on the consumption of sugar-sweetened beverages in young people living in poorer neighbourhoods of Catalonia, Spain: A difference in differences study. BMC Public Health 2019;**19**(1) doi: 10.1186/s12889-019-7908-5.

11. Alvarado M, Kostova D, Suhrcke M, et al. Trends in beverage prices following the introduction of a tax on sugar-sweetened beverages in Barbados. Preventive Medicine 2017;**105**:S23-S25 doi: 10.1016/j.ypmed.2017.07.013.

12. Cawley J, Willage B, Frisvold D. Pass-through of a taxon sugar-sweetened beverages at the philadelphia international airport. JAMA - Journal of the American Medical Association 2018;**319**(3):305-06 doi: 10.1001/jama.2017.16903.

For peer review only - http://bmjopen.bmj.com/site/about/guidelines.xhtml

13. Falbe J, Rojas N, Grummon AH, Madsen KA. Higher retail prices of sugar-sweetened beverages 3 months after implementation of an excise tax in Berkeley, California. American Journal of Public Health 2015;**105**(11):2194-201 doi: 10.2105/AJPH.2015.302881.

14. Mytton OT, Eyles H, Ogilvie D. Evaluating the health impacts of food and beverage taxes. Curr Obes Rep 2014;**3**(4):432-39.

15. Scarborough P, Adhikari V, Harrington RA, et al. Impact of the announcement and implementation of the UK soft drinks industry levy on sugar content, price, product size and number of available soft drinks in the UK, 2015-19: A controlled interrupted time series analysis. PLoS Medicine 2020;**17**(2) doi: 10.1371/JOURNAL.PMED.1003025.

16. Stacey N, Mudara C, Ng SW, van Walbeek C, Hofman K, Edoka I. Sugar-based beverage taxes and beverage prices: Evidence from South Africa's Health Promotion Levy. Social Science and Medicine 2019;**238** doi: 10.1016/j.socscimed.2019.112465.

17. Penney T, Adams J, Briggs A, et al. Theorising how the UK Soft Drinks Industry Levy could impact population level diet and health: Development of a multi-sectoral systems map. International Society for Behavioural Nutrition and Physical Activity. Victoria, Canada, 2017.

18. Quirmbach DD, Cornelsen L, Jebb SA, Marteau T, Smith R. Effect of increasing the price of sugar-sweetened beverages on alcoholic beverage purchases: an economic analysis of sales data. Journal of Epidemiology and Community Health 2018;**72**(4):324-30 doi: 10.1136/jech-2017-209791.

19. Scheelbeek PFD, Cornelsen L, Marteau TM, Jebb SA, Smith RD. Potential impact on prevalence of obesity in the UK of a 20% price increase in high sugar snacks: Modelling study. BMJ 2019;**366** doi: 10.1136/bmj.l4786.

20. Smith RD, Cornelsen L, Quirmbach D, Jebb SA, Marteau TM. Are sweet snacks more sensitive to price increases than sugar-sweetened beverages: Analysis of British food purchase data. BMJ Open 2018;**8**(4) doi: 10.1136/bmjopen-2017-019788.

21. HM Revenue & Customs. Soft Drinks Industry Levy. Secondary Soft Drinks Industry Levy 2016. https://www.gov.uk/government/publications/soft-drinks-industry-levy/soft-drinks-industry-levy.

22. Gauke D. Budget 2016. London: HM Treasury, 2016.

23. HM Revenue & Customs. Check if your drink is liable for the Soft Drinks Industry Levy. Secondary Check if your drink is liable for the Soft Drinks Industry Levy 2018. https://www.gov.uk/guidance/check-if-your-drink-is-liable-for-the-soft-drinks-industry-levy.

24. Bandy LK, Scarborough P, Harrington RA, Rayner M, Jebb SA. Reductions in sugar sales from soft drinks in the UK from 2015 to 2018. BMC Medicine 2020;**18**(1) doi: 10.1186/s12916-019-1477-4.

25. Public Health England. Sugar Reduction: Report on Progress between 2015 and 2018. London: Public Health England, 2019.

For peer review only - http://bmjopen.bmj.com/site/about/guidelines.xhtml

26. British Soft Drinks Association. 2018 Annual Report. London: British Soft Drinks Association, 2019.

27. Pell D, Penney TL, Mytton O, et al. Anticipatory changes in British household purchases of soft drinks associated with the announcement of the Soft Drinks Industry Levy: A controlled interrupted time series analysis. PLoS Medicine 2020;**17**(11) doi: 10.1371/journal.pmed.1003269.

28. White M, Cummins S, Rayner M, et al. Evaluation of the health impacts of the UK Treasury Soft Drinks Industry Levy (SDIL). NIHR Public Health Research Programme 2017

29. Webber D, O'Neill J. Average Household Income, UK: Financial Year Ending 2019. Secondary Average Household Income, UK: Financial Year Ending 2019. 2020. https://www.ons.gov.uk/peoplepopulationandcommunity/personalandhouseholdfinances/incomeandwealth/bulletins/householddisposableincomeandinequality/financialyearending2019provisional#:~:text=The%20distribution%20of%20people's%20household,year%20ending%20(FYE)%202019.

30. Lopez Bernal J, Cummins S, Gasparrini A. The use of controls in interrupted time series studies of public health interventions. International Journal of Epidemiology 2018;**47**(6):2082-93 doi: 10.1093/ije/dyy135.

31. Zhang F, Wagner AK, Soumerai SB, Ross-Degnan D. Methods for estimating confidence intervals in interrupted time series analyses of health interventions. Journal of Clinical Epidemiology 2009;**62**(2):143-48 doi: 10.1016/j.jclinepi.2008.08.007.

32. Pell D, Mytton O, Penney TL, et al. Changes in soft drinks purchased by British households associated with the UK soft drinks industry levy: controlled interrupted time series analysis. BMJ 2021;**372**:n254 doi: 10.1136/bmj.n254 [RETRACTED].

33. Adams J. Rapid response to: Changes in soft drinks purchased by British households associated with the UK soft drinks industry levy: controlled interrupted time series analysis. Secondary Rapid response to: Changes in soft drinks purchased by British households associated with the UK soft drinks industry levy: controlled interrupted time series analysis 2022. https://www.bmj.com/content/372/bmj.n254/rr-0.

34. Knipe E. Families and Households in the UK: 2017. Secondary Families and Households in the UK: 2017. 2017. https://www.ons.gov.uk/peoplepopulationandcommunity/birthsdeathsandmarriages/families/bulletins/familiesandhouseholds/2017.

35. Briggs ADM, Mytton OT, Kehlbacher A, et al. Health impact assessment of the UK soft drinks industry levy: a comparative risk assessment modelling study. The Lancet Public Health 2017;**2**(1):e15-e22 doi: 10.1016/S2468-2667(16)30037-8.

36. Griffith R, O’Connell M, Smith K. Measuring the quality of people’s diets: a comparison of intake and purchase data. Econometrics and IO of food and nutrition. Toulouse School of Economics 2012.

37. Law C, Cornelsen L, Adams J, et al. The impact of UK soft drinks industry levy on manufacturers’ domestic turnover. Economics and Human Biology 2020;**37** doi: 10.1016/j.ehb.2020.100866.

For peer review only - http://bmjopen.bmj.com/site/about/guidelines.xhtml

38. Law C, Cornelsen L, Adams J, et al. An analysis of the stock market reaction to the announcements of the UK Soft Drinks Industry Levy. Economics and Human Biology 2020;**38** doi: 10.1016/j.ehb.2019.100834.

39. Pell D, Penney T, Hammond D, Vanderlee L, White M, Adams J. Support for, and perceived effectiveness of, the UK soft drinks industry levy among UK adults: Cross-sectional analysis of the International Food Policy Study. BMJ Open 2019;**9**(3) doi: 10.1136/bmjopen-2018-026698.

40. Colchero MA, Popkin BM, Rivera JA, Ng SW. Beverage purchases from stores in Mexico under the excise tax on sugar sweetened beverages: Observational study. BMJ 2016;**352** doi: 10.1136/bmj.h6704.

41. Males J, Van Aelst P. Did the Blue Planet set the Agenda for Plastic Pollution? An Explorative Study on the Influence of a Documentary on the Public, Media and Political Agendas. Environmental Communication 2020:1-15 doi: 10.1080/17524032.2020.1780458.

42. O'Mahony A. The rise and fall: Bottled water category report 2020. *The Grocer* 2020.

43. World Health Organization. Use of non-sugar sweeteners: WHO guideline. Geneva: World Health Organization; 2023. Geneva: World Health Organization, 2023.

For peer review only

For peer review only - http://bmjopen.bmj.com/site/about/guidelines.xhtml

Figure legends

Figure 1. Schematic of overall analysis strategy. Solid lines=observed data; dashed lines=counterfactual estimated from previous observed data.

Figure 2. Observed and modelled volume (mLs) of drinks liable to the Soft Drinks Industry Levy (SDIL), and weight of confectionery (g) purchased per household per week, March 2014 to March 2019 (weighted). Points are observed data for drinks/ confectionery; black lines (with shadows) show modelled data (and 95% confidence intervals); red lines indicate the counterfactuals had the announcement (red solid line) and implementation (red dashed line) not happened; the first dashed vertical line indicates the announcement of SDIL; the second dashed vertical line indicates the implementation of SDIL; the Y axis varies in scale between panels to maximise the resolution of figures; modelled purchases include averaged effects for seasonality and the impact of December and January (Christmas period), and, for confectionery, Easter; the control category of toiletries is shown in Figure 3.

Figure 3. Observed and modelled amount of sugar (g) in drinks liable to the Soft Drinks Industry Levy and confectionery purchased per household per week, March 2014 to March 2019 (weighted). Points are observed data for drinks/ confectionery and toiletries; black lines (with shadows) show modelled data (and 95% confidence intervals); red lines indicate the counterfactuals had the announcement (red solid line) and implementation (red dashed line) not happened; the first dashed vertical line indicates the announcement of SDIL; the second dashed vertical line indicates the implementation of SDIL; the Y axis varies in scale between panels to maximise the resolution of figures; modelled purchases include averaged effects for seasonality and the impact of December and January (Christmas period), and, for confectionery, Easter.

For peer review only

For peer review only - http://bmjopen.bmj.com/site/about/guidelines.xhtml

Table 1. Mean volume of, and amount of sugar in, purchased drinks and confectionery per household per week in relation to the UK soft drinks industry levy, March 2014 to March 2019 (weighted)

	**Mean (SD) volume (mL) per household/week**			**Mean (SD) amount of sugar (g) per household/week**		
	**Pre-announcement: Mar 2014-Mar 2016**	**Post-announcement: Mar 2016-Mar 2018**	**Post- implementation: Apr 2018-Mar 2019**	**Pre-announcement: Mar 2014-Mar 2016**	**Post- announcement: Mar 2016-Mar 2018**	**Post- implementation: Apr 2018-Mar 2019**
**Levy liable drinks (sugar/100 mL)**						
High tier (≥8 g)	880 (128)	680 (136)	363 (76)	98(14)	76(15)	40(9)
Low tier (≥5 g to <8 g	155 (32)	147 (37)	75 (32)	10(2)	10(2)	5(2)
No levy (<5 g)	1811 (169)	1876 (216)	2448 (321)	12(2)	12(3)	25(5)
>0 g to <5 g	785 (78)	768 (92)	989 (139)	12(2)	12(3)	25(5)
0 g	1027 (104)	1108 (132)	1459 (190)	0 (0)	0 (0)	0 (0)
Bottled water	591 (72)	714 (90)	786 (138)	0 (0)	0 (0)	0 (0)
**Levy exempt drinks**						
Alcoholic drinks*	1874 (380)	1872 (456)	1948 (467)	.	.	.
Milk and milk based drinks**	3546 (137)	3540 (155)	3542 (148)	172 (7)	172(8)	170(7)
Fruit juices with no added sugar	516 (29)	502 (44)	520 (47)	51(3)	49(4)	50(5)
Drinks sold as powders (g)	95 (12)	88 (11)	90 (11)	21(3)	19(3)	18(3)
**Other categories**						
Confectionery (g)	308 (91)	303 (92)	318 (100)	173 (51)	170 (52)	178 (57)
Toiletries	123 (8)	120 (8)	121 (9)	.	.	.
**All soft drinks combined (ie excluding alcohol)**	7595 (295)	7547 (466)	7826 (540)	364 (17)	337(24)	307(19)

*Sugar from alcoholic drinks is not included here as many alcoholic drinks contain sugar but the product label does not provide the amount.
**Milk comprises drinks in the following categories: semi-skimmed; specific low fat % milk (e.g. 1% fat milk); whole milk; buttermilk; modified milk; other milk; other non-cows milk; rice drink; soya milk. Skimmed milk is excluded from all analysis in this paper due to missing data.

For peer review only - http://bmjopen.bmj.com/site/about/guidelines.xhtml

For peer review only

Table 2. Absolute and relative change in volume of drinks (mL) and confectionery (g) purchased per household per week in relation to the UK soft drinks industry Levy, March 2014 to March 2019 (weighted)

	**Analysis 1: pre-post announcement (Mar 2014-Mar 2018)**		**Analysis 2: pre-post implementation (Mar 2016-Mar 2019)**		**Analysis 3: pre-post announcement and implementation (Mar 2014-Mar 2019)**	
	**Absolute change (mL or g)**	**Relative change (%)**	**Absolute change (mL or g)**	**Relative change (%)**	**Absolute change (mL or g)**	**Relative change (%)**
**Levy liable drinks (sugar/100 mL)**						
High tier (≥8 g)	**34.7 (8.06, 61.4)**	**7.27 (1.69, 12.9)**	**-171.6 (-208.1,-135.1)**	**-42.5 (-51.6, -33.5)**	**-140.8 (-177.3, -104.3)**	**-37.8 (-47.6, -28.0)**
Low tier (≥5 g to <8 g)	**-65.7 (-77.5, -53.8)**	**-37.1 (-43.7, -30.4)**	**-71.8 (-87.8, -55.8)**	**-71.8 (-87.8, -55.8)**	**-170.5 (-186.5, -154.5)**	**-85.8 (-93.9, -77.8)**
No levy (<5 g):	**181.0 (118.4, 243.5)**	**11.1 (7.26, 14.9)**	**395.0 (309.4. 480.7)**	**19.8 (15.5, 24.1)**	**685.5 (599.8, 771.1)**	**40.2 (35.2, 45.2)**
>0 g to <5 g	**103.8 (75.2, 132.5)**	**16.7 (12.1, 21.3)**	**202.0 (162.7, 241.2)**	**25.0 (20.1, 29.9)**	**374.6 (335.4, 413.9)**	**59.0 (52.8, 65.1)**
0 g	**87.8 (41.1, 134.5)**	**8.66 (4.05, 13.3)**	**178.9 (115.6, 242.3)**	**14.7 (9.52, 20.0)**	**316.1 (252.7, 379.4)**	**29.4 (23.5, 35.3)**
Bottled water	30.3 (-62.0, 1.4)	4.24 (-8.7, 0.2)	**82.1 (-125.7, -38.4)**	**-10.5 (-16.1, -4.9)**	**-130.5 (-174.1, -88.8)**	**-15.7 (-20.9, -10.4)**
**Levy exempt drinks**						
Alcoholic drinks	-16.5 (-48.5, 15.4)	0.95 (-2.79, 0.89)	**-84.9 (-135.1, -34.7)**	**-4.81 (-7.66, -1.97)**	**-103.1 (-153.3, -53.0)**	**-5.8 (-8.60, -2.97)**
Milk and milk based drinks*	**-185.5 (-249.7, -121.4)**	**-4.9 (-6.60, -3.20)**	**145.5 (64.4, 226.6)**	**4.21 (1.86, 6.56)**	**-132.8 (-213.9, -51.7)**	**-3.56 (-5.73, -1.38)**
No added sugar fruit juices	6.8 (-6.9, 20.5)	1.4 (-1.4, 4.3)	-6.2 (-24.8, 12.5)	-1.26 (-6.1, 2.5)	8.7 (-9.9, 27.3)	1.82 (-2.1, 5.7)
Drinks sold as powders (g)	**-6.9 (-10.0, -3.8)**	**-6.8 (-9.9, -3.8)**	**9.6 (5.3, 13.9)**	**11.2 (6.2, 16.2)**	0.9 (-3.3, 5.2)	1.0 (-3.5, 5.5)
**Other categories**						
Confectionery (g)	-10.1 (-53.9, 33.8)	-2.4 (-13.1, 8.2)	39.8 (-19.0, 98.6)	11.6 (-5.5, 28.8)	35.3 (94.1, -23.5)	10.2 (-6.8, 27.1)
**All soft drinks combined (ie excluding alcohol)**	11.8 (-103.7, 127.3)	0.16 (-1.42, 1.74)	**187.8 (29.7, 345.9)**	**2.56 (0.40, 4.71)**	**188.8 (30.7, 346.9)**	**2.6 (0.4, 4.7)**

Bold indicates significant difference at 95% confidence interval level.
*Milk comprises drinks in the following categories: semi-skimmed; specific low fat % milk (e.g. 1% fat milk); whole milk; buttermilk; modified milk; other milk; other non-cows milk; rice drink; soya milk. Skimmed milk is excluded from all analysis in this paper due to missing data.

For peer review only

For peer review only - http://bmjopen.bmj.com/site/about/guidelines.xhtml

Table 3. Absolute and relative change in sugar in drinks and confectionery (g) purchased per household (95% CI) per week in relation to the UK SDIL, March 2014- March 2019 (weighted)

	**Analysis 1: pre-post announcement (Mar 2014-Mar 2018)**		**Analysis 2: pre-post implementation (Mar 2016-Mar 2019)**		**Analysis 3: pre-post announcement and implementation (Mar 2014-Mar 2019)**	
	**Absolute change (g)**	**Relative change (%)**	**Absolute change (g)**	**Relative change (%)**	**Absolute change (g)**	**Relative change (%)**
**All drinks**						
**Levy liable drinks (sugar/100 mL)**						
High tier (≥8 g)	**5.5 (3.8, 7.2)**	**10.8 (7.4, 14.1)**	**-21.2 (-23.8, -18.5)**	**-49.3 (-55.4, -43.1)**	**-16.2 (-18.8, -13.5)**	**-42.6 (-49.6, -35.6)**
Low tier (≥5 g to <8 g)*	**-4.3 (-6.1, -2.6)**	**-37.5 (-52.5, -22.5)**	**-5.0 (-7.4, -2.6)**	**-75.8 (-112.7, -38.9)**	**-11.5 (-13.9, -9.1)**	**-87.8 (-106.4, -69.2)**
No levy (<5 g)**	**5.7 (3.9, 7.4)**	**72.6 (50.3, 94.9)**	**9.7 (7.3,12.1)**	**56.0 (41.9, 70.0)**	**19.2 (16.7, 21.6)**	**242.8 (211.9, 273.7)**
>0 g to <5 g	**5.7 (3.9, 7.4)**	**72.6 (50.3, 94.9)**	**9.7 (7.3,12.1)**	**56.0 (41.9, 70.0)**	**19.2 (16.7, 21.6)**	**242.8 (211.9, 273.7)**
**Levy exempt drinks**						
Milk and milk based drinks†	**-3.9 (-6.5, -1.3)**	**-2.2 (-3.6, -0.7)**	**4.1 (0.5, 7.7)**	**2.4 (0.3, 4.6)**	-3.1 (-6.7, 0.5)	-1.8 (-3.8, 0.3)
No added sugar fruit juices	**2.6 (0.3, 4.8)**	**5.7 (0.7, 10.7)**	-1.7 (-4.8, 1.5)	-3.5 (-10.0, 3.0)	2.6 (-0.5, 5.7)	5.9 (-1.2, 13.1)
Drinks sold as powders	0.3 (-1.6, 2.2)	1.6 (-7.5, 10.6)	-0.04 (-2.7, 2.6)	-0.2 (-13.9, 13.5)	1.1 (-1.6, 3.7)	5.7 (-8.8, 20.2)
**Other categories**						
Confectionery	-6.6 (-32.0, 18.9)	-2.8 (-13.8, 8.14)	22.1 (-12.0, 56.1)	11.4 (-6.2, 29.1)	18.4 (-15.7, 52.4)	9.3 (-8.0, 26.7)
**All soft drinks combined (ie excluding alcohol)**	**4.6 (0.5, 8.6)**	**1.4 (0.2, 2.7)**	**-12.9 (-18.5, -7.4)**	**-4.3 (-6.1, -2.4)**	**-8.0 (-13.6, -2.4)**	**-2.7 (-4.5, -0.8)**

Bold indicates significant difference at 95% confidence interval level.
*The counterfactual for low tier drinks crossed 0 mL shortly before the end of the study period thus predicting negative purchases; therefore, the non-counterfactual estimate at the end of the study period was compared with the final week during which the counterfactual was a positive number.
**We do not report change in sugar purchased from drinks with 0g sugar/100ml or bottled water as these contain no sugar; the figures for the combined No levy line and the >0g to <5g of sugar/100ml line are the same as the only drinks in the No levy category containing sugar are those with >0g to <5g
† Milk comprises drinks in the following categories: semi-skimmed; specific low fat % milk (e.g. 1% fat milk); whole milk; buttermilk; modified milk; other milk; other non-cows milk; rice drink; soya milk. Skimmed milk is excluded from all analysis in this paper due to missing data.

For peer review only - http://bmjopen.bmj.com/site/about/guidelines.xhtml

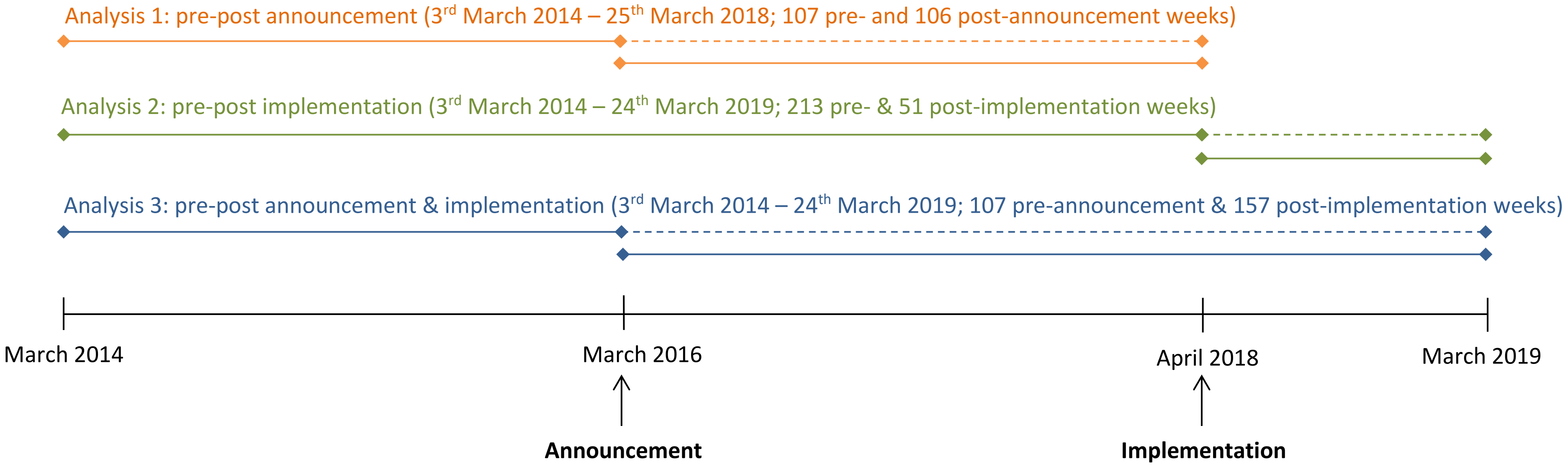

Figure 1: Schematic of overall analysis strategy

review only

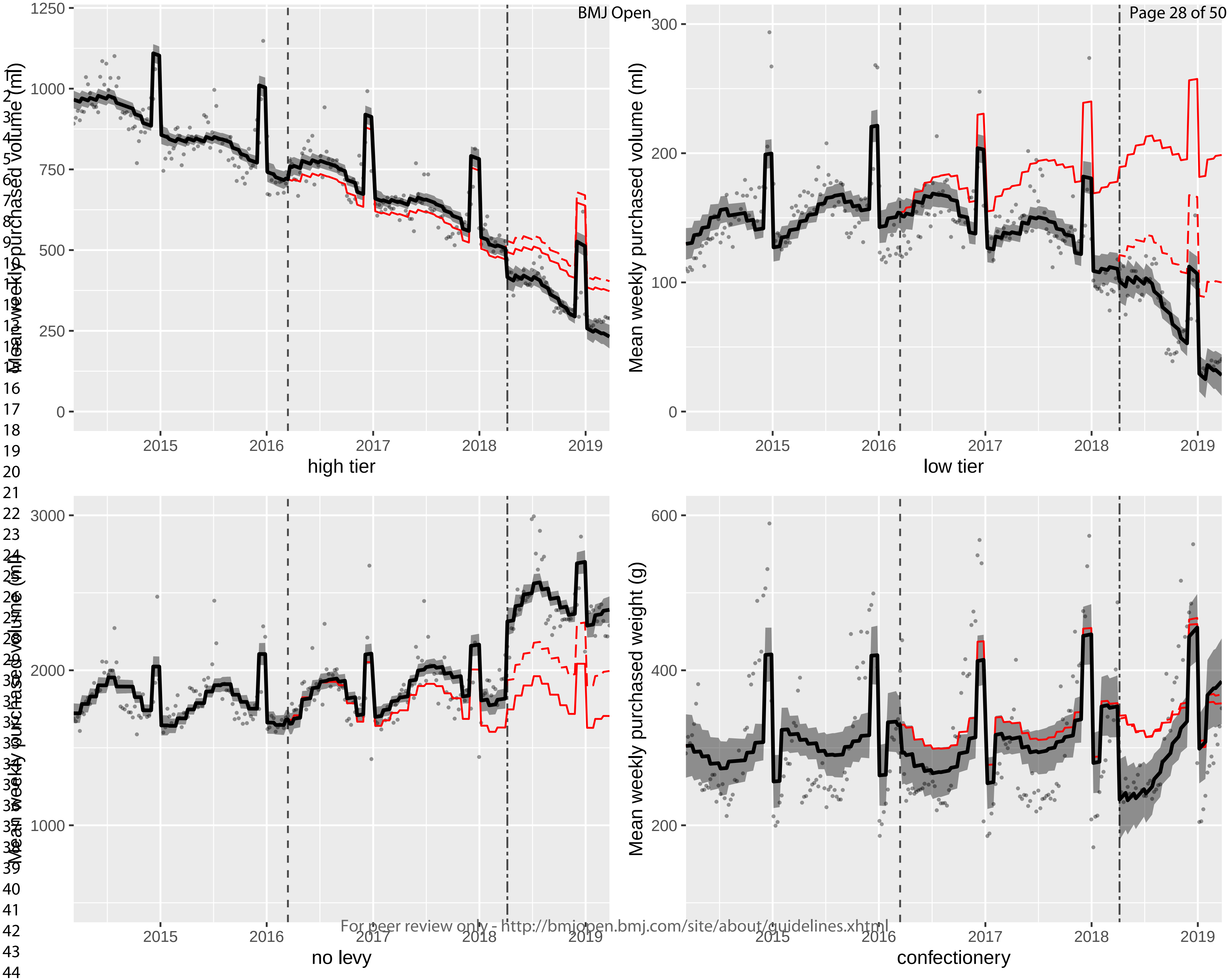
Mean weekly purchased volume (ml)
1250
1000
750
500
250
0
2015
2016
2017
2018
2019
high tier
Mean weekly purchased volume (ml)
300
200
100
0
low tier
Mean weekly purchased volume (ml)
3000
2000
1000
no levy
Mean weekly purchased weight (g)
600
400
200
confectionery

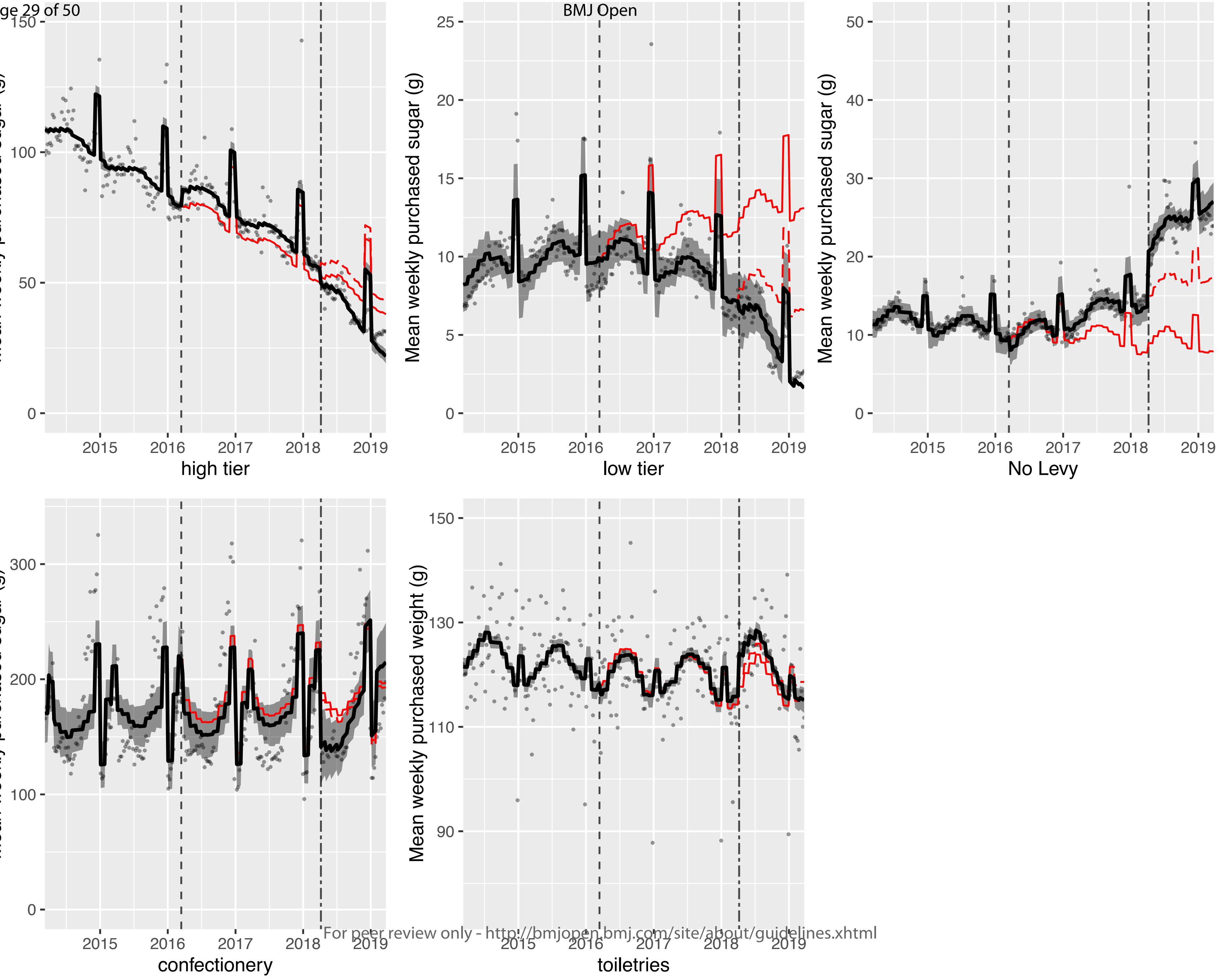
Mean weekly purchased sugar (g)
high tier
low tier
No Levy
confectionery
Mean weekly purchased weight (g)
toiletries
2015
2016
2017
2018
2019

Supplementary material A

STROBE Statement—checklist of items that should be included in reports of observational studies

	Item No	Recommendation	Section and Paragraph No
**Title and abstract**	1	(*a*) Indicate the study's design with a commonly used term in the title or the abstract	Title.
		(*b*) Provide in the abstract an informative and balanced summary of what was done and what was found	Abstract: main outcome measures; results; conclusions.
**Introduction**			
Background/rationale	2	Explain the scientific background and rationale for the investigation being reported	Introduction: paragraphs 1-3.
Objectives	3	State specific objectives, including any prespecified hypotheses	Introduction: paragraph 3-4.
**Methods**			
Study design	4	Present key elements of study design early in the paper	Methods: paragraph 1.
Setting	5	Describe the setting, locations, and relevant dates, including periods of recruitment, exposure, follow-up, and data collection	Methods: data source; overall analysis strategy; Figure 1.
Participants	6	(*a*) *Cohort study*—Give the eligibility criteria, and the sources and methods of selection of participants. Describe methods of follow-up<br>*Case-control study*—Give the eligibility criteria, and the sources and methods of case ascertainment and control selection. Give the rationale for the choice of cases and controls<br>*Cross-sectional study*—Give the eligibility criteria, and the sources and methods of selection of participants	Methods: data source, product categories – drinks, confectionery and toiletries; control group; outcome measures.
		(*b*) *Cohort study*—For matched studies, give matching criteria and number of exposed and unexposed<br>*Case-control study*—For matched studies, give matching criteria and the number of controls per case	NA.
Variables	7	Clearly define all outcomes, exposures, predictors, potential confounders, and effect	Outcome measures; overall analysis strategy; primary analysis: category specific analyses; secondary analysis: all soft drinks categories combined, irrespective of levy

For peer review only

		modifiers. Give diagnostic criteria, if applicable	eligibility; sensitivity analysis: excluding small manufacturers; sensitivity analysis: ITS without a control category; supplementary material B.
Data sources/ measurement	8*	For each variable of interest, give sources of data and details of methods of assessment (measurement). Describe comparability of assessment methods if there is more than one group	Data source; overall analysis strategy; primary analysis: category specific analyses; secondary analysis: all soft drinks categories combined, irrespective of levy eligibility; sensitivity analysis: excluding small manufacturers; sensitivity analysis: ITS without a control category; supplementary material B.
Bias	9	Describe any efforts to address potential sources of bias	Product categories: drinks confectionery and toiletries; control group; sensitivity analysis: excluding small manufacturers; sensitivity analysis: ITS without a control category.
Study size	10	Explain how the study size was arrived at	Data source.
Quantitative variables	11	Explain how quantitative variables were handled in the analyses. If applicable, describe which groupings were chosen and why	Overall analysis strategy; primary analysis: category specific analyses; secondary analysis: all soft drinks categories combined, irrespective of levy eligibility; Box 1.
Statistical methods	12	(*a*) Describe all statistical methods, including those used to control for confounding	Control group; sensitivity analysis: ITS without a control category; Supplementary material A.
		(*b*) Describe any methods used to examine subgroups and interactions	Overall analysis strategy; primary analysis: category specific analyses; secondary analysis: all soft drinks categories combined, irrespective of levy eligibility.
		(*c*) Explain how missing data were addressed	Methods: data source
		(*d*) *Cohort study*—If applicable, explain how loss to follow-up was addressed *Case-control study*—If applicable, explain how matching of cases and controls was addressed *Cross-sectional study*—If applicable, describe analytical methods taking account of sampling strategy	Methods: data source.

Results

Participants	13*	(a) Report numbers of individuals at each stage of study—eg numbers potentially eligible, examined for eligibility, confirmed eligible, included in the study, completing follow-up, and analysed	Results: paragraph 1.
		(b) Give reasons for non-participation at each stage	Data source.
		(c) Consider use of a flow diagram	-
Descriptive data	14*	(a) Give characteristics of study participants (eg demographic, clinical,	Results: paragraph 1; Supplementary material C.

For peer review only

		social) and information on exposures and potential confounders	
		(b) Indicate number of participants with missing data for each variable of interest	Results: paragraph 1.
		(c) *Cohort study*—Summarise follow-up time (eg, average and total amount)	NA.
Outcome data	15*	*Cohort study*—Report numbers of outcome events or summary measures over time	NA.
		Case-control study—Report numbers in each exposure category, or summary measures of exposure	NA.
		Cross-sectional study—Report numbers of outcome events or summary measures	Results: paragraph 2; Table 1.
Main results	16	(*a*) Give unadjusted estimates and, if applicable, confounder-adjusted estimates and their precision (eg, 95% confidence interval). Make clear which confounders were adjusted for and why they were included	Primary analysis: category specific results; high tier drinks; low tier drinks; no levy drinks; levy exempt drinks and confectionery; Figures 2-3; Tables 2-3; supplementary material D.
		(*b*) Report category boundaries when continuous variables were categorized	Introduction - paragraph 3; product categories: drinks, confectionery and toiletries; Box 1.
		(*c*) If relevant, consider translating estimates of relative risk into absolute risk for a meaningful time period	Tables 2-3.
Other analyses	17	Report other analyses done—eg analyses of subgroups and interactions, and sensitivity analyses	Sensitivity analysis: excluding small manufacturers; sensitivity analysis: no control category; supplementary tables 4a-b; supplementary material G.
**Discussion**			
Key results	18	Summarise key results with reference to study objectives	Summary of main findings.
Limitations	19	Discuss limitations of the study, taking into account sources of potential bias or imprecision. Discuss both direction and magnitude of any potential bias	Key strengths and limitations.
Interpretation	20	Give a cautious overall interpretation of results considering objectives, limitations, multiplicity of analyses, results from similar studies, and other relevant evidence	Interpretation of findings - paragraphs 1-3.
Generalisability	21	Discuss the generalisability (external validity) of the study results	Interpretation of findings - paragraphs 4.
**Other information**			
Funding	22	Give the source of funding and the role of the funders for the present study and, if applicable, for the original study on which the present article is based	Funding.

*Give information separately for cases and controls in case-control studies and, if applicable, for exposed and unexposed groups in cohort and cross-sectional studies.

For peer review only

Note: An Explanation and Elaboration article discusses each checklist item and gives methodological background and published examples of transparent reporting. The STROBE checklist is best used in conjunction with this article (freely available on the Web sites of PLoS Medicine at http://www.plosmedicine.org/, Annals of Internal Medicine at http://www.annals.org/, and Epidemiology at http://www.epidem.com/). Information on the STROBE Initiative is available at www.strobe-statement.org.

For peer review only

Supplementary material B

The model specification is given by:

$Y_t = \beta_0 + \beta_1 T + \beta_2 A_t + \beta_3 A_t\, T + \beta_4 A_t\, Z + \beta_5 A_t\, TZ + \beta_6 I_t + \beta_7 I_t\, T + \beta_8 I_t\, Z + \beta_9 I_t\, TZ + e_t$

Y	*Average volume of (or purchased sugar in) drink or confectionery per household per week at week t (t=1,….,264)*
T	*Weeks since the start of the study; 1,…,264*
A_t	*0 if t prior to announcement, 1 if t on or after announcement*
I_t	*0 if t prior to implementation, 1 if t on or after implementation*
Z	*Control category (toiletries) = 0, drink or confectionery category = 1*
e_t	$N(0,\sigma^2)$ *representing the residual variance of the model*

Dummy indicator variables determined to be statistically significant ($p<0.05$) were included for the intervention group as appropriate representing: interaction terms restart at 0 at the point of the interventions; the increase in purchases seen throughout December in the weeks before Christmas; the fall in purchases in the weeks immediately after Christmas; and the increase in confectionery purchases seen at Easter, for toiletries these were set to 0. To adjust for temperature-related trends in drink consumption the average UK monthly temperature was included in the intervention group with the average study period temperature used for toiletries.[5] Quadratic functions of announcement trends were included where they improved model fit - assessed using likelihood ratio tests. Stationary was examined using augmented Dickey-Fuller tests.[41] Autocorrelation between preceding time points was examined using autocorrelation and partial-autocorrelation plots. An appropriate autocorrelation structure was determined and then compared to alternative models using likelihood ratio tests. Visual inspection of the data suggested no additional benefit would be gained from including polynomial terms.

For peer review only

Supplementary material C

Supplementary Table 1: Demographic characteristics of Kantar Worldpanel take-home panel households from March 2014 – March 2018 (weighted)

	Level	Kantar Worldpanel (%)	UK population (%)
Children in household[1]	No	65.3	71.5
	Yes	34.6	28.5
Social grade of chief income earner[2]	AB: Higher and intermediate managerial	19.3	27
	C1: Junior managerial	34.1	28
	C2: Skilled manual workers	16.4	20
	D: Semi and unskilled-manual workers	12.4	15
	E: lowest grade workers	7.9	10
Total household income (£ per annum)[3]	0-9,999	6.4	..
	10,000-19,999	18.9	..
	20,000-29,999	17.2	..
	30,000-39,999	13.1	....
	40,000-49,999	9.0	..
	50,000-59,999	5.5	..
	60,000-69,999	2.9	..
	70,000+	4.1	..
	Refused to answer	14.4	..
	Mean (£)	..	35,697
	Median (£)	..	28,947
Highest qualification of chief income earner[4]	Higher than School leaving qualifications taken at ~18 years (e.g. A-Levels)	38.3	43.8
	School leaving qualifications taken at ~18 years (e.g. A-Levels)	12.3	22.4
	School leaving qualifications taken at ~16 years (e.g. GCSE)	20.6	18.7
	Other (including no qualifications and unknown)	16.2	15.1

[1]Average of households with dependent children from 2014-2018; [2]UK population figures from 2016; [3]No directly comparable figures available from ONS, mean and medians are averaged over 2014-2019; [4]UK population figures from 2014

For peer review only

Supplementary material D

Supplementary Figure 1a. Observed and modelled volume (ml) of drinks exempt from the Soft Drinks Industry Levy, subcategories of low levy drinks and bottled water purchased per household per week, March 2014- March 2019 (weighted)

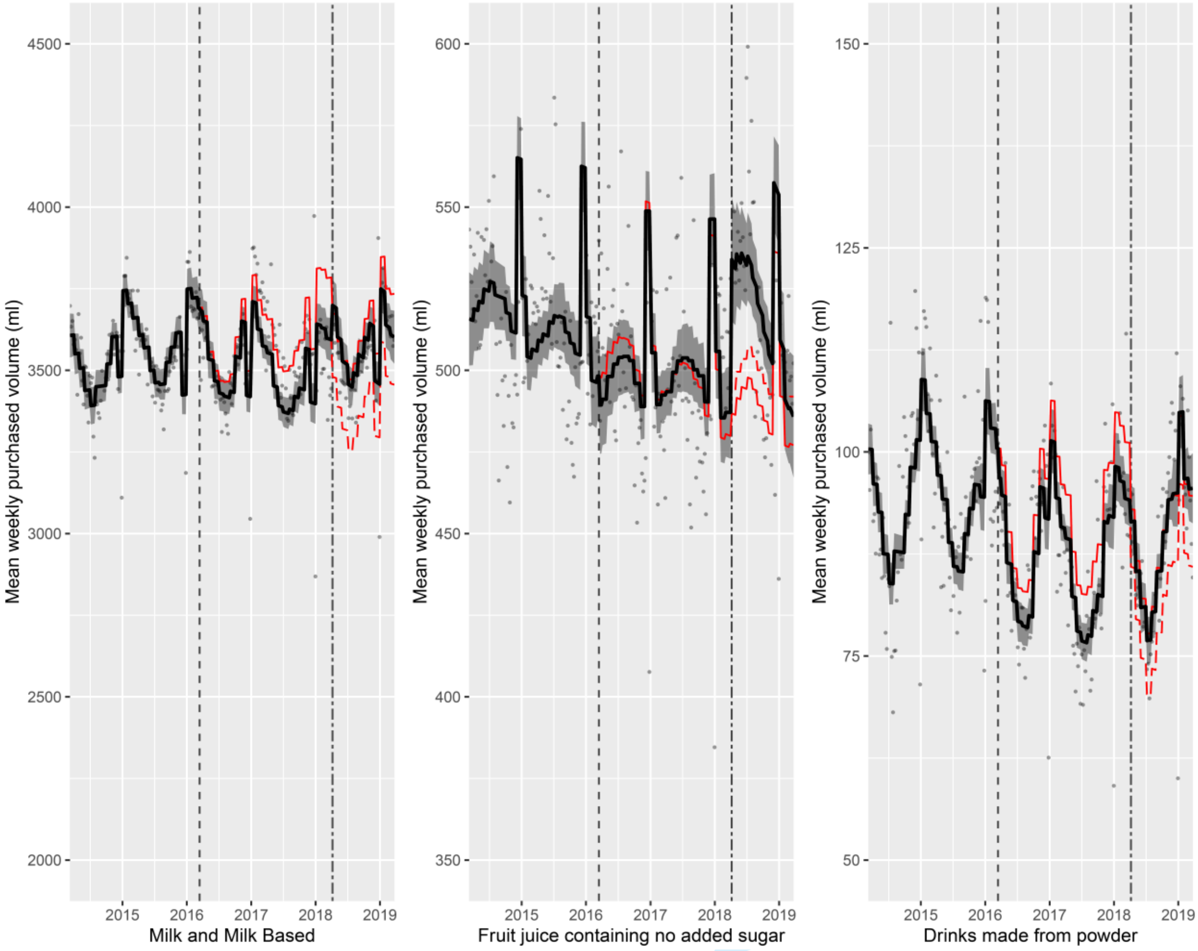

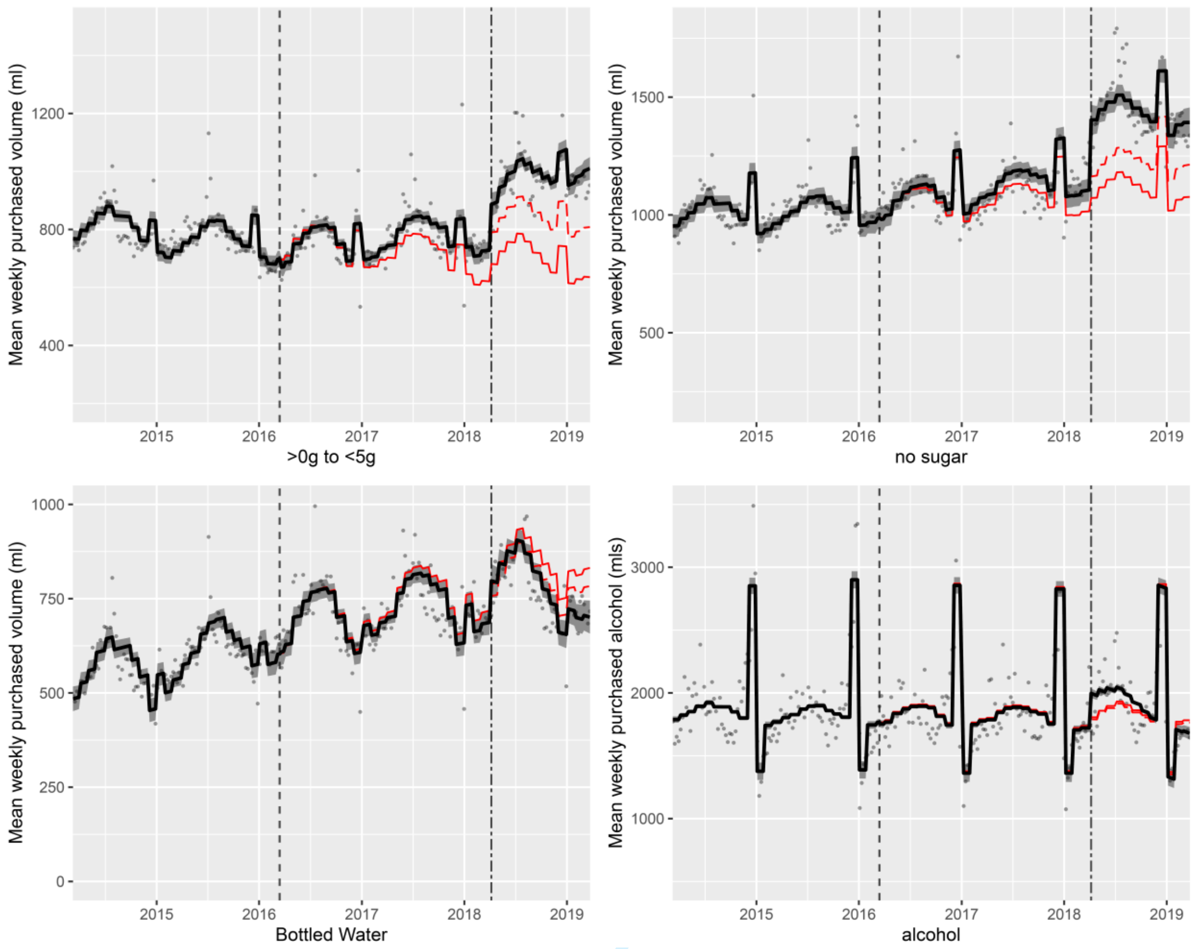

Notes. Points are observed data, black lines (with shadows) are modelled data (and 95% confidence intervals); red lines indicate counterfactuals had the announcement (red solid line) and implementation (red dashed line) not happened; the first dashed vertical line indicates the point of announcement; the second dashed vertical line indicates the point of implementation; The Y-axis varies in scale between panels to maximise the resolution of figures; modelled purchases include averaged effects for seasonality and the impact of December and January (Christmas period). The control category of toiletries is shown in Figure 3.

view only

Supplementary Figure 1b. Observed and modelled amount of sugar in no levy drinks containing sugar (low sugar) and drinks exempt from the Soft Drinks Industry Levy purchased per household per week, March 2014- March 2019 (weighted)

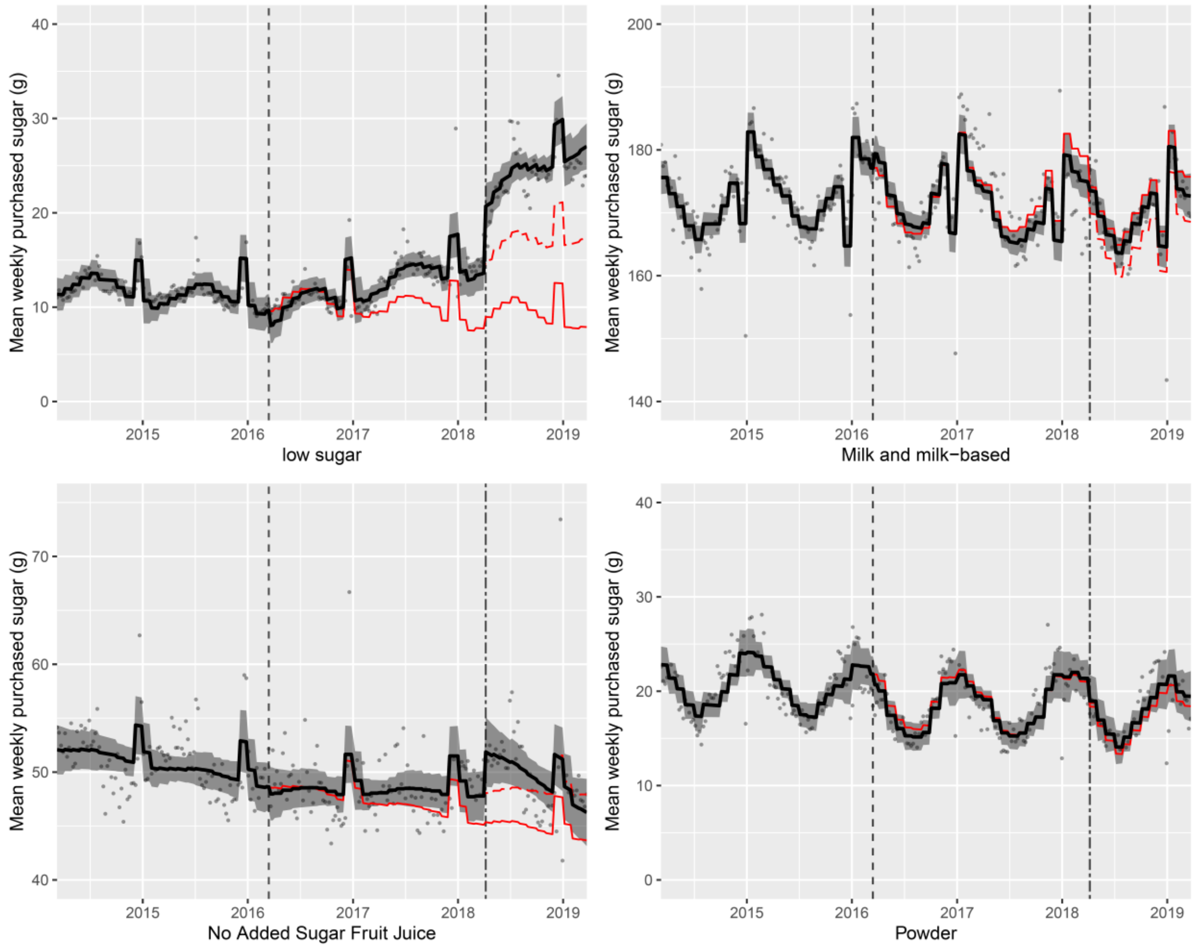

Notes. Points are observed data, black lines (with shadows) are modelled data (and 95% confidence intervals); red lines indicate counterfactuals had the announcement (red solid line) and implementation (red dashed line) not happened; the first dashed vertical line indicates the point of announcement; the second dashed vertical line indicates the point of implementation; The Y-axis varies in scale between panels to maximise the resolution of figures; modelled purchases include averaged effects for seasonality and the impact of December and January (Christmas period). The control category of toiletries is shown in Figure 3.

Supplementary Table 2a: Modelled level and trend changes in volume of drinks (ml) and confectionery (g) purchased per household per week (95% CI) in relation to the UK SDIL, March 2014 - March 2019 (weighted)

	Analysis 1: pre-post announcement (March 2014 – March 2018)		Analysis 2: pre-post implementation (March 2016 – March 2019)		Analysis 3: pre-post announcement & implementation (March 2014 – March 2019)	
	Level Change	Trend Change	Level Change	Trend Change	Level Change	Trend Change
Levy liable drinks						
High tier (≥8g sugar per 100ml)	44.0 (-7.7, 95.7)	-0.1 (-0.9, 0.7)	**-117.2 (-183.3, -51.1)**	1.0 (-0.9, 2.9)	-73.2 (-157.1, 10.7)	0.9 (-1.2, 3.0)
Low tier (≥5g - <8g sugar per 100ml)	-0.11 (-22.4, 22.1)	**-0.6 (-1.0, -0.3)**	-26.3 (-53.6, 1.04)	-0.81 (-1.6, 0.01)	-26.4 (-61.7, 8.8)	**-1.5 (-2.4, -0.6)**
No levy (<5g sugar per 100ml)	-28.2 (-149.4, 92.9)	**2.0 (0.04, 3.9)**	**372.0 (217.6, 526.4)**	**0.52 (-3.9, 4.9)**	**343.8 (147.5, 540.0)**	2.5 (-2.3, 7.3)
>0g to <5g sugar per 100ml	-27.1 (-82.6, 28.5)	**1.3 (0.4, 2.2)**	**88.1 (17.0, 159.3)**	**2.31 (0.3, 4.3)**	61.0 (-29.2, 151.4)	**3.6 (1.4, 5.8)**
0g sugar per 100ml	-7.37 (-93.9, 79.2)	0.9 (-0.6, 2.4)	**231.0 (125.2, 336.8)**	-0.99 (-4.3, 2.3)	**223.6 (87.0, 360.3)**	-0.08 (-3.7, 3.5)
Bottled water	6.86 (-53.4, 67.2)	-0.4 (-1.4, 0.6)	36.6 (-38.8, 112.0)	-2.24 (-4.5, 0.005)	43.5 (-53.1, 140.0)	**-2.6 (-5.1, -0.2)**
Levy exempt drinks						
Alcoholic drinks	-10.8 (-48.6, 27.0)	-0.07 (-0.5, 0.3)	**208.0 (137.9, 278.1)**	**-5.7 (-7.8, -3.6)**	**197.2 (117.6, 276.8)**	**-5.8 (-7.9, -3.7)**
Milk and milk based drinks*	-6.61 (-105.8, 92.6)	-1.7 (-4.0, 0.5)	**210.7 (98.7, 322.7)**	-1.2 (-5.3, 2.9)	**204.1 (54.4, 353.7)**	-2.9 (-7.6, 1.8)
No added sugar fruit juices	-8.72 (-34.3, 16.9)	0.1 (-0.3, 0.6)	30.5 (-0.9, 61.9)	-0.7 (-1.6, 0.3)	21.78 (-18.8, 62.3)	-0.5 (-1.6, 0.5)
Drinks sold as powders (g)	-1.79 (-7.8, 4.18)	-0.06 (-0.2, 0.03)	0.10 (-7.7, 7.9)	**0.3 (0.04, 0.5)**	-1.69 (-11.6, 8.17)	0.2 (-0.04, 0.4)
Other categories						
Confectionery (g)	-17.0 (-88.0, 54.0)	0.07 (-1.3, 1.5)	-77.9 (-163.9, 8.19)	2.4 (-0.6, 5.4)	**-98.9 (-206.4, -16.7)**	2.5 (-0.8, 5.8)

Bold indicates a significant difference at the 95% confidence interval level.
*Milk comprises drinks in the following categories: semi-skimmed; specific low fat % milk (e.g. 1% fat milk); whole milk; buttermilk; modified milk; other milk; other non-cows milk; rice drink; soya milk. Skimmed milk is excluded from all analysis in this paper due to missing data.

For peer review only

Supplementary Table 2b: Modelled level and trend changes in sugar in drinks and confectionery (g) purchased per household per week (95% CI) in relation to the UK SDIL, March 2014 - March 2019 (weighted)

	Analysis 1: pre-post announcement (March 2014 – March 2018)		Analysis 2: pre-post implementation (March 2016 – March 2019)		Analysis 3: pre-post announcement & implementation (March 2014 – March 2019)	
	Level Change	Trend Change	Level Change	Trend Change	Level Change	Trend Change
Levy liable drinks						
High tier (≥8g sugar per 100ml)	**8.9 (6.8, 10.9)**	**-0.05 (-0.08, -0.03)**	**-11.5 (-15.3, -7.7)**	**-0.1 (-0.2, -0.02)**	-2.6 (-6.9, 1.6)	**-0.2 (-0.3, -0.07)**
Low tier (≥5g - <8g sugar per 100ml)	1.7 (-1.6, 5.1)	**-0.07 (-0.1, -0.02)**	**-6.2 (-10.6, -1.8)**	0.09 (-0.03, 0.2)	-4.5 (-10.0, 1.1)	0.02 (-0.1, 0.2)
No levy (<5g sugar per 100ml)	0.2 (-3.1, 3.5)	0.04 (-0.01, 0.09)	0.8 (-3.6, 5.3)	**0.2 (0.1, 0.4)**	1.0 (-4.5, 6.6)	**0.3 (0.2, 0.4)**
>0g to <5g sugar per 100ml	0.2 (-3.1, 3.5)	0.04 (-0.01, 0.09)	0.8 (-3.6, 5.3)	**0.2 (0.1, 0.4)**	1.0 (-4.5, 6.6)	**0.3 (0.2, 0.4)**
Levy exempt drinks						
Milk and milk based drinks*	4.1 (-0.1, 9.1)	**-0.09 (-0.2, -0.01)**	-2.0 (-8.6, 4.6)	**0.2 (0.01, 0.4)**	2.1 (-6.2, 10.4)	0.1 (-0.09, 0.3)
No added sugar fruit juices	1.0 (-3.4, 5.4)	0.001 (-0.06, 0.06)	-1.6 (-7.3, 4.1)	0.07 (-0.09, 0.2)	-0.6 (-7.8, 6.6)	0.07 (-0.1, 0.2)
Drinks sold as powders (g	0.6 (-3.3, 4.5)	-0.02 (-0.08, 0.04)	-5.1 (-0.004, 10.2)	**0.2 (0.03, 0.3)**	-4.5 (-10.9, 1.9)	**0.2 (0.001, 0.3)**
Other categories						
Confectionery (g)	-8.6 (-49.7, 32.6)	0.03 (-0.8, 0.9)	-49.4 (-99.2, 0.4)	1.5 (-0.2, 3.2)	**-58.0 (-122.6, -6.7)**	1.6 (-0.4, 3.5)

Bold indicates a significant difference at the 95% confidence interval level.
*Milk comprises drinks in the following categories: semi-skimmed; specific low fat % milk (e.g. 1% fat milk); whole milk; buttermilk; modified milk; other milk; other non-cows milk; rice drink; soya milk. Skimmed milk is excluded from all analysis in this paper due to missing data.

For peer review only

Supplementary material E

Secondary analysis: all drinks categories combined irrespective of levy eligibility

Supplementary Table 3a: Level and trend changes in volume of, and sugar in, all soft drinks (ml) and confectionery (g) purchased per household per week (95% CI) in relation to the UK SDIL, March 2014 - March 2019 (weighted)

	Analysis 1: pre-post announcement (March 2014 – March 2018)		Analysis 2: pre-post implementation (March 2016 – March 2019)		Analysis 3: pre-post announcement & implementation (March 2014 – March 2019)	
	Level Change	Trend Change	Level Change	Trend Change	Level Change	Trend Change
All soft drinks - volume						
Higher tier (≥8g sugar per 100ml)	22.8 (-37.5, 83.1)	0.5 (-0.5, 1.5)	-76.8 (-154.0 ,0.4)	-2.2 (-4.4, 0.01)	-54.0 (-152.0, 44.0)	-1.7 (-4.1, 0.7)
Lower tier (≥5g - <8g sugar per 100ml)	19.0 (-52.2, 90.2)	**-3.2 (-5.9, -0.5)**	27.9 (-47.8, 103.6)	-4.4 (-8.9, 0.02)	46.9 (-57.0, 150.8)	**-7.6 (-12.9, -2.4)**
Drinks containing <5g sugar per 100ml)	-152.1 (-348.3, 44.1)	2.6 (-0.7, 5.8)	**435.7 (187.7, 683.7)**	1.1 (-6.0, 8.3)	283.6 (-32.6, 599.8)	3.7 (-4.2, 11.5)
Levy liable drinks combined	15.2 (-72.9, 103.3)	0.6 (-0.8, 2.0)	-39.7 (-152.7, 73.4)	0.01 (-3.2, 3.2)	-24.5 (-167.8, 118.9)	0.6 (-2.9, 4.1)
All soft drinks excluding milk*	12.9 (-205.3, 231.0)	1.0 (-2.6, 4.5)	**347.6 (70.1, 625.0)**	-5.2 (-13.1, 2.7)	**360.5 (7.47, 713.4)**	-4.2(-12.9, 4.5)
All soft drinks combined	33.9 (-189.0, 256.7)	-0.2 (-3.8, 3.4)	**453.0 (170.1, 735.9)**	-5.1 (-13.2, 3.0)	**486.9 (126.7, 847.0)**	-5.4 (-14.2, 3.5)
All soft drinks – sugar						
Higher tier (≥8g sugar per 100ml)	4.1 (-2.6, 10.8)	0.04 (-0.07, 1.1)	**-13.4 (-22.2, -4.7)**	-0.1 (-0.4, 0.1)	-9.3 (-20.3, 1.7)	-0.08 (-0.3, 0.2)
Lower tier (≥5g - <8g sugar per 100ml)	**11.0 (5.6, 16.3)**	**-0.2 (-0.3, -0.1)**	-2.7 (-9.67, 4.3)	-0.01 (-0.2,0.2)	8.3 (-0.5, 17.1)	**-0.2 (-0.4, -0.01)**
Drinks containing <5g sugar per 100ml)	-6.3 (-12.2, -0.4)	**1.0 (0.9, 1.0)**	-0.7 (-8.30, 7.0)	**0.4 (0.2, 0.6)**	-7.0 (-16.6, 2.7)	**1.4 (1.1, 1.6)**
Levy liable drinks combined	5.0 (-1.8, 11.8)	-0.003 (-0.1, 0.1)	**-13.1 (-22.0, -4.2)**	0.02 (-0.2, 0.3)	-8.1(-19.3, 3.1)	0.02 (-0.3, 0.3)
All soft drinks excluding milk*	3.1 (-4.6, 10.8)	0.04 (-0.08, 0.2)	-9.3 (-19.2, 0.7)	-0.09 (-0.4, 0.2)	-6.2 (-18.8, 6.4)	-0.05 (-0.4, 0.3)
All soft drinks combined	5.3 (-2.6, 13.2)	-0.02 (-0.1, 0.1)	-5.7 (-15.9, 4.5)	-0.08 (-0.4, 0.2)	-0.4 (-13.2, 12.5)	-0.1 (-0.4, 0.2)

Bold indicates a significant difference at the 95% confidence interval level. The levy liable drinks category is a combination of high tier, low tier and no levy drinks.

*Milk comprises drinks in the following categories: semi-skimmed; specific low fat % milk (e.g. 1% fat milk); whole milk; buttermilk; modified milk; other milk; other non-cows milk; rice drink; soya milk. Skimmed milk is excluded from all analysis in this paper due to missing data.

For peer review only

Supplementary Table 3b: Absolute and relative change in volume of, and sugar in, all soft drinks and confectionery (g) purchased per household (95% CI) per week in relation to the UK SDIL, March 2014- March 2019 (weighted)

	Analysis 1: pre-post announcement (March 2014 – March 2018)		Analysis 2: pre-post implementation (March 2016 – March 2019)		Analysis 3: pre-post announcement & implementation (March 2014 – March 2019)	
	Absolute change (ml or g)	Relative change (%)	Absolute change (ml or g)	Relative change (%)	Absolute change (ml or g)	Relative change (%)
All soft drinks - volume						
High tier (≥8g sugar per 100ml)	**76.6 (45.6, 107.7)**	**7.5 (4.5, 10.6)**	**-190.9 (-233.5, -148.3)**	**-19.4 (-23.7, -15.1)**	**-85.6 (-128.2, -43.0)**	**-9.8 (-14.6, -4.9)**
Low tier (≥5g - <8g sugar per 100ml)	316.7 (-241,7, -391.7)	**-28.0 (-21.3, -34.6)**	**-205.2 (-295.9, -114.5)**	**-26.2 (-37.8, -14.6)**	**-693.6 (-784.3, -602.9)**	**-54.5 (-61.7, -47.4)**
Drinks containing <5g sugar per 100ml)	**114.3 (12.4, 216.2)**	**2.2 (0.2, 4.1)**	**506.7 (367.3, 646.1)**	**9.0 (6.5, 11.4)**	**760.2 (620.8, 899.6)**	**14.1 (11.5, 16.6)**
Levy liable drinks combined	**76.5 (31.1, 121.8)**	**6.0 (2.5, 9.6)**	-42.7 (-104.9, 19.6)	-3.3(-8.1, 1.5)	**66.3 (4.1, 128.6)**	**5.6 (0.3, 10.8)**
All soft drinks excluding milk*	**116.1 (3.3, 229.0)**	**3.3 (0.09, 6.4)**	79.6 (-74.8, 234.1)	2.1 (-2.0, 6.1)	**250.1 (95.7, 404.5)**	**6.8 (2.6, 11.0)**
All soft drinks combined	11.8 (-103.7, 127.3)	0.2 (-1.4, 1.7)	**187.8 (29.7, 345.9)**	**2.6 (0.4, 4.7)**	**188.8 (30.7, 346.9)**	**2.6 (0.4, 4.7)**
All soft drinks – sugar						
High tier (≥8g sugar per 100ml)	**9.3 (5.9, 12.8)**	**7.6 (4.8, 10.4)**	**-22.9 (-27.8, -18.1)**	**-19.4 (-23.4, -15.3)**	**-9.99 (-14.8, -5.18)**	**-9.5 (-14.0, -4.9)**
Low tier (≥5g - <8g sugar per 100ml)	**-9.7 (-12.5, -6.9)**	**-18.6 (-23.9, -13.3)**	**-7.2 (-11.0, -3.4)**	**-19.4 (-29.7, -9.1)**	**-26.7 (-30.5, -22.9)**	**-47.1 (-53.9, -40.4)**
Drinks containing <5g sugar per 100ml)	**5.1 (2.1, 8.1)**	**3.6 (1.5, 5.8)**	**16.7 (12.5, 20.9)**	**11.2 (8.4, 14.0)**	**28.6 (24.4, 32.8)**	**20.9 (17.9, 23.9)**
Levy liable drinks combined	**6.1 (2.6, 9.6)**	**8.6 (3.6, 13.5)**	**-15.5 (-20.4, -10.7)**	**-22.7 (-29.8, -15.6)**	**-8.0 (-12.9, -3.2)**	**-13.2 (-21.1, -5.2)**
All soft drinks excluding milk*	**8.9 (5.0, 12.8)**	**6.5 (3.6, 9.4)**	**-17.2 (-22.7, -11.8)**	**-12.7 (-16.7, -8.67)**	**-4.5 (-10.0, 1.0)**	**-3.7 (-8.1, 0.8)**
All soft drinks combined	**4.6 (0.5, 8.6)**	**1.4 (0.2, 2.7)**	**-12.9 (-18.5, -7.4)**	**-4.3 (-6.1, -2.4)**	**-8.0 (-13.6, -2.4)**	**-2.7 (-4.5, -0.8)**

Bold indicates a significant difference at the 95% confidence interval level. The levy liable drinks category is a combination of high tier, low tier and no levy drinks.

*Milk comprises drinks in the following categories: semi-skimmed; specific low fat % milk (e.g. 1% fat milk); whole milk; buttermilk; modified milk; other milk; other non-cows milk; rice drink; soya milk. Skimmed milk is excluded from all analysis in this paper due to missing data.

For peer review only

Supplementary material F
Sensitivity analysis: excluding small manufacturers

Supplementary table 4a: Modelled level and trend changes in volume of, and sugar in, drinks (ml) purchased per household per week (95% CI) in relation to the UK SDIL, March 2014 - March 2019; excluding small manufacturers (weighted)

	Analysis 1: pre-post announcement (March 2014 – March 2018)		Analysis 2: pre-post implementation (March 2016 – March 2019)		Analysis 3: pre-post announcement & implementation (March 2014 – March 2019)	
	Level Change	Trend Change	Level Change	Trend Change	Level Change	Trend Change
Volume						
High levy tier (≥8g sugar per 100ml)						
All Manufacturers	44.0 (-7.7, 95.7)	-0.1 (-0.9, 0.7)	**-117.2 (-183.3, -51.1)**	1.0 (-0.9, 2.9)	-73.2 (-157.1, 10.7)	0.9 (-1.2, 3.0)
Excluding Manufacturers with <1M Litres	41.5 (-9.7, 92.7)	-0.09 (-0.9, 0.7)	**-111.9 (-177.3, -46.6)**	-1.1 (-2.9, 0.8)	-70.8 (-153.4, 12.6)	-1.2 (-3.2, 0.9)
Excluding Manufacturers with <0.5M Litres	41.5 (-9.7, 92.7)	-0.09 (-0.9, 0.7)	**-111.9 (-177.3, -46.6)**	-1.1 (-2.9, 0.8)	-70.8 (-153.4, 12.6)	-1.2 (-3.2, 0.9)
Low levy tier (≥5g - <8g sugar per 100ml)						
All Manufacturers	-0.1 (-22.4, 22.1)	**-0.6 (-1.0, -0.3)**	-26.3 (-53.6, 1.0)	-0.8 (-1.6, 0.01)	-26.4 (-61.7, 8.8)	**-1.5 (-2.4, -0.6)**
Excluding Manufacturers with <1M Litres	-2.1 (-24.0, 19.9)_	**-0.7 (-1.0, -0.3)**	-20.9 (-47.5, 5.7)	**-0.9 (-1.8, -0.1)**	-23.0 (-57.5, 11.6)	**-1.6 (-2.5, -0.7)**
Excluding Manufacturers with <0.5M Litres	-2.1 (-24.0, 19.6)_	**-0.7 (-1.0, -0.3)**	-20.7 (-47.3, 5.9)	**-0.9 (-1.8, -0.1)**	-22.9 (-57.2, 11.5)	**-1.6 (-2.5, -0.7)**
Sugar						
High tier (≥8g sugar per 100ml)						
All Manufacturers	**8.9 (6.8, 10.9)**	**-0.05 (-0.08, -0.03)**	**-11.5 (-15.3, -7.7)**	**-0.1 (-0.2, -0.02)**	-2.6 (-6.9, 1.6)	**-0.2 (-0.3, -0.07)**
Excluding Manufacturers with <1M Litres	4.5 (-1.3, 10.2)	-0.03 (-0.1, 0.06)	**-13.5 (-20.8, -6.1)**	-0.07 (-0.3, 0.1)	-9.0 (-18.3, 0.4)	-0.1 (-0.3, 0.1)
Excluding Manufacturers with <0.5M Litres	4.5 (-1.3, 10.2)	-0.03 (-0.1, 0.06)	**-13.5 (-20.8, -6.1)**	-0.07 (-0.3, 0.1)	-9.0 (-18.3, 0.4)	-0.1 (-0.3, 0.1)
Low tier (≥5g - <8g sugar per 100ml)						
All Manufacturers	1.7 (-1.6, 5.1)	**-0.07 (-0.1, -0.02)**	**-6.2 (-10.6, -1.8)**	0.09 (-0.03, 0.2)	-4.5 (-10.0, 1.1)	0.02 (-0.1, 0.2)
Excluding Manufacturers with <1M Litres	-0.4 (-2.1, 1.3)	**-0.07 (-0.1, -0.04)**	**-2.5 (-4.6, -0.4)**	0.002 (-0.06, 0.06)	**-3.0 (-5.7, -0.2)**	-0.007 (-0.1, 0.0001)
Excluding Manufacturers with <0.5M Litres	-0.5 (-2.1, 1.2)	**-0.07 (-0.1, -0.04)**	**-2.5 (-4.7, -0.4)**	0.003 (-0.06, 0.06)	**-3.0 (-5.7, -0.2)**	-0.007 (-0.1, 0.0001)

Bold indicates a significant difference at the 95% confidence interval level.

For peer review only

Supplementary Table 4b: Modelled absolute and relative change in volume of, and sugar in, all drinks (ml) purchased per household (95% CI) per week in relation to the UK SDIL, March 2014- March 2019; excluding small manufacturers (weighted)

	Analysis 1: pre-post announcement (March 2014 – March 2018)		Analysis 2: pre-post implementation (March 2016 – March 2019)		Analysis 3: pre-post announcement & implementation (March 2014 – March 2019)	
	Absolute change (ml)	Relative change (%)	Absolute change (ml)	Relative change (%)	Absolute change (ml)	Relative change (%)
Volume						
High tier (≥8g sugar per 100ml)						
All Manufacturers	**34.7 (8.1, 61.4)**	**7.3 (1.7, 12.9)**	**-171.6 (-208.1, -135.1)**	**-42.5 (-51.6, -33.5)**	**-140.8 (-177.3, -104.3)**	**-37.8 (-47.6, -28.0)**
Excluding Manufacturers with <1M Litres	**35.3 (8.9, 61.6)**	**7.5 (1.9, 13.1)**	**-168.1 (-204.2, -131.9)**	**-42.6 (-51.7, -33.4)**	**-136.2 (-172.3, -100.1)**	**-37.6 (-47.5, -27.6)**
Excluding Manufacturers with <0.5M Litres	**35.3 (8.9, 61.6)**	**7.5 (1.9, 13.1)**	**-168.1 (-204.2, -131.9)**	**-42.6 (-51.7, -33.4)**	**-136.2 (-172.3, -100.1)**	**-37.6 (-47.5, -27.6)**
Low tier (≥5g - <8g sugar per 100ml)						
All Manufacturers	**-65.7 (-77.5, -53.8)**	**-37.1 (-43.7, -30.4)**	**-71.8 (-87.8, -55.8)**	**-71.8 (-87.8, -55.8)**	**-170.5 (-186.5, -154.5)**	**-85.8 (-93.9, -77.8)**
Excluding Manufacturers with <1M Litres	**-66.4 (-78.2, -54.6)**	**-37.6 (-44.3, -30.9)**	**-71.2 (-87.1, -55.3)**	**-72.5 (-88.7, -56.3)**	**-171.1 (-187.0, -155.2)**	**-86.4 (-94.4, -78.4)**
Excluding Manufacturers with <0.5M Litres	**-66.5 (-78.2, -54.7)**	**-37.6 (-44.2, -31.0)**	**-71.3 (-87.1, -55.5)**	**-72.4 (-88.5, -56.3)**	**-171.2 (-187.1, -155.4)**	**-86.3 (-94.3, -78.3)**
Sugar						
High tier (≥8g sugar per 100ml)						
All Manufacturers	**5.5 (3.8, 7.3)**	**10.8 (7.4, 14.1)**	**-21.2 (-23.8, -18.5)**	**-49.3 (-55.4, -43.1)**	**-16.2 (-18.8, -13.5)**	**-42.6 (-49.6, -35.6)**
Excluding Manufacturers with <1M Litres	**4.7 (1.7, 7.7)**	**9.3 (3.4, 15.1)**	**-19.3 (-23.4, -15.2)**	**-44.3 (-53.6, -35.0)**	**-14.6 (-18.7, -10.6)**	**-37.6 (-48.1, -27.1)**
Excluding Manufacturers with <0.5M Litres	**4.7 (1.7, 7.7)**	**9.3 (3.4, 15.1)**	**-19.3 (-23.4, -15.2)**	**-44.3 (-53.6, -35.0)**	**-14.6 (-18.7, -10.6)**	**-37.6 (-48.1, -27.1)**
Low tier (≥5g - <8g sugar per 100ml)						
All Manufacturers	**-4.3 (-6.1, -2.6)**	**-37.5 (-52.5,-22.5)**	**-5.0 (-7.4, -2.6)**	**-75.8 (-112.7, -38.9)**	**-11.5 (-13.9, -9.07)**	**-87.8 (-106.4, -69.2)**
Excluding Manufacturers with <1M Litres	**-4.7 (-5.5, -3.8)**	**-39.1 (-46.5, -31.7)**	**-4.8 (-6.0, -3.6)**	**-73.0 (-91.3, -54.7)**	**-11.8 (-13.0, -10.6)**	**-86.9 (-95.8, -78.1)**
Excluding Manufacturers with <0.5M Litres	**-4.7 (-5.6, -3.8)**	**-39.1 (-46.5, -31.8)**	**-4.8 (-6.0, -3.6)**	**-72.9 (-91.1, -54.6)**	**-11.8 (-13.0, -10.6)**	**-86.9 (-95.7, -78.0)**

Bold indicates a significant difference at the 95% confidence interval level.

For peer review only

Supplementary Figure 2a. Observed and modelled volume (ml) in all soft drinks combined purchased per household per week, March 2014- March 2019 (weighted)

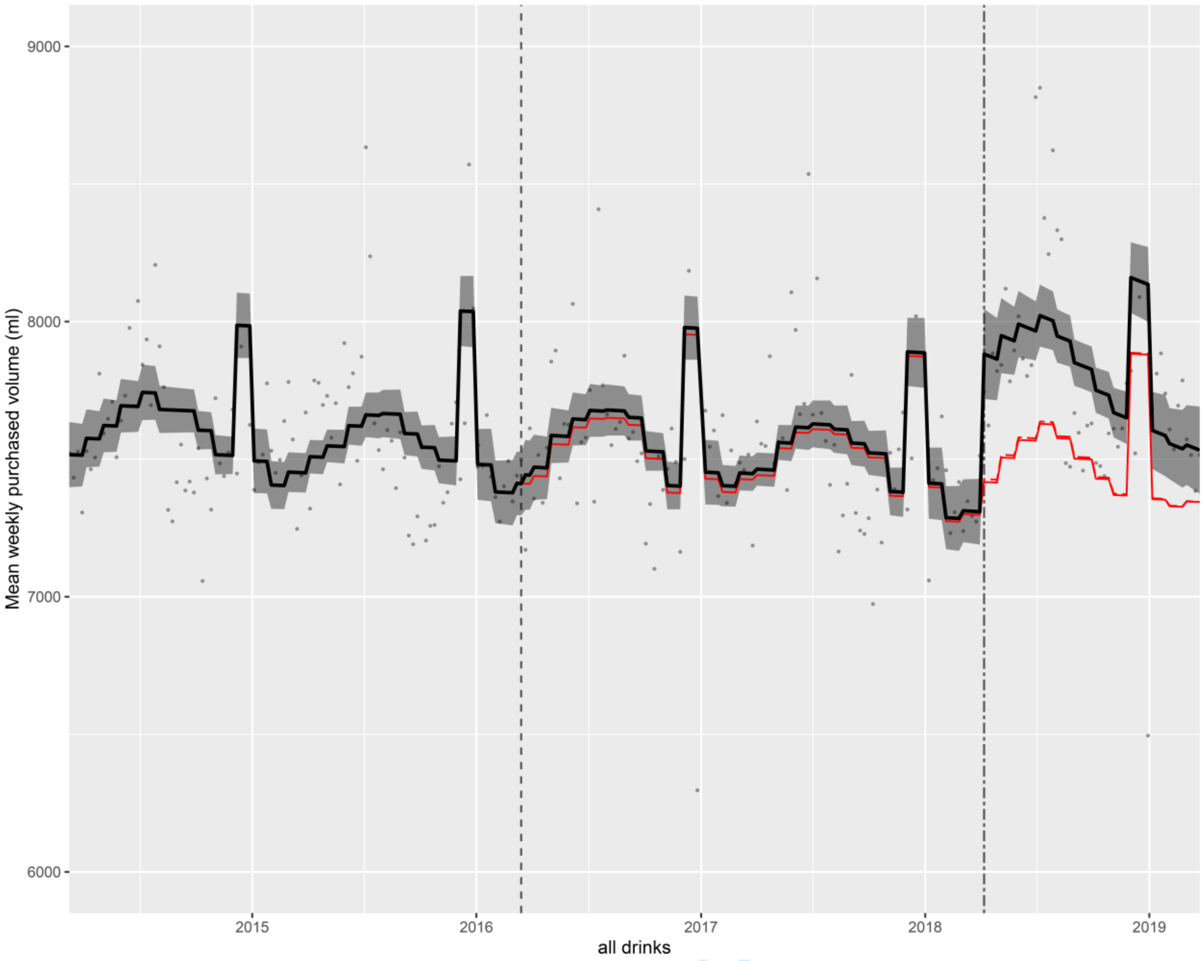

Notes. Points are observed data, black lines (with shadows) are modelled data (with 95% confidence intervals); red lines indicate the counterfactuals had the announcement (red solid line) and implementation (red dashed line) not happened; the first dashed vertical line indicates the point of announcement; the second dashed vertical line indicates the point of implementation; modelled purchases include averaged effects for seasonality and the impact of December and January (the Christmas period). The control category of toiletries is shown in Fig 3.

Supplementary Figure 2b. Observed and modelled amount of sugar (g) in all soft drinks combined purchased per household per week, March 2014- March 2019 (weighted)

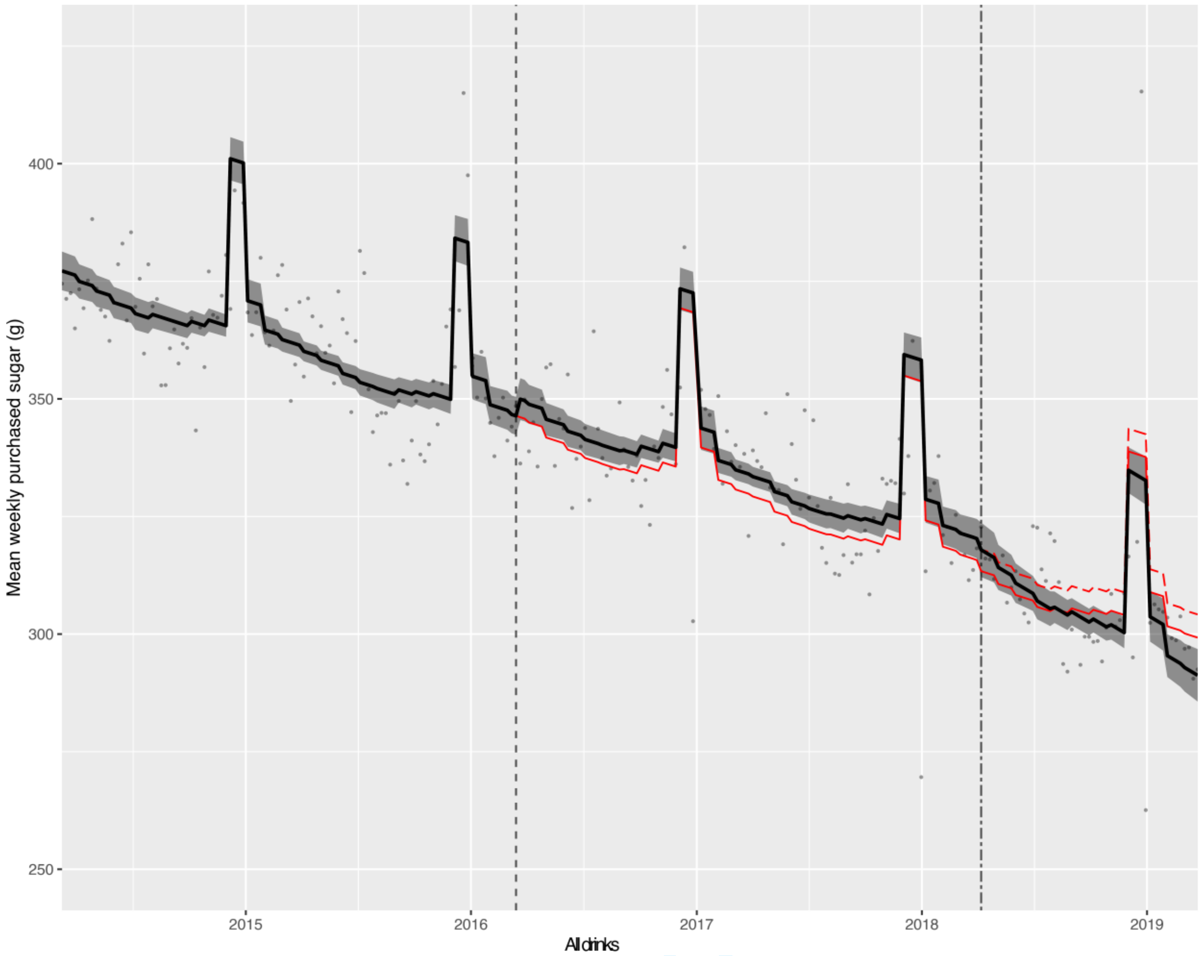

Notes. Points are observed data, black lines (with shadows) are modelled data (with 95% confidence intervals); red lines indicate the counterfactuals had the announcement (red solid line) and implementation (red dashed line) not happened; the first dashed vertical line indicates the point of announcement; the second dashed vertical line indicates the point of implementation; modelled purchases include averaged effects for seasonality and the impact of December and January (the Christmas period). The control category of toiletries is shown in Fig 3.

Supplementary material G

Removing the control category led to wider confidence intervals in a small number of cases such that absolute and relative changes in volume were not significantly different from the pre-implementation counterfactuals for no levy drinks and the pre-announcement and post implementation counterfactual for drinks containing 0g of sugar per 100ml. Significantly lower volumes of purchased volumes of powdered drinks were seen following the announcement in the controlled analysis unlike in the uncontrolled analysis. In the uncontrolled analysis absolute and relative differences in the amount of sugar in milk based drinks were significantly different from the pre-implementation counterfactual but not significantly different when examining the impact of the SDIL overall. Additionally the amount of sugar in confectionery was not significantly different from the pre-announcement counterfactual in the uncontrolled ITS analysis unlike in the controlled analysis.

For peer review only

Supplementary Table 5a: Level and trend changes in volume of drinks (ml) and confectionery (g) purchased per household per week (95% CI) in relation to the UK SDIL, March 2014 - March 2019 without toiletries as a control condition (weighted)

	Analysis 1: pre-post announcement (March 2014 – March 2018)		Analysis 2: pre-post implementation (March 2016 – March 2019)		Analysis 3: pre-post announcement & implementation (March 2014 – March 2019)	
	Level Change	Trend Change	Level Change	Trend Change	Level Change	Trend Change
Levy liable drinks						
High tier (≥8g sugar per 100ml)	42.3 (-9.4, 94.0)	-0.07 (-0.9, 0.8)	**-111.2 (-177.1, -45.3)**	-1.2 (-3.0, 0.7)	-68.9 (-152.7, 14.9)	-1.3 (-3.3, 0.8)
Low tier (≥5g - <8g sugar per 100ml)	-2.2 (-24.1, 19.8)	**-0.6 (-1.0, -0.2)**	-18.9 (-45.8, 8.0)	-1.0 (1.9, -0.2)	-21.1 (-55.75, 13.6)	**-1.7 (-2.6, -0.7)**
No levy (<5g sugar per 100ml)	-29.8 (-150.9, 91.3)	**2.0 (0.08, 4.0)**	**378.1 (223.7, 532.5)**	**0.3 (0.5, 2.0)**	**348.3 (152.0, 544.6)**	2.4 (-2.4, 7.2)
>0g to <5g sugar per 100ml	-27.9 (-78.3, 22.5)	**1.3 (0.5, 2.0)**	**98.0 (32.7, 163.3)**	**2.1 (0.3, 3.9)**	70.1 (-12.4, 152.6)	**3.4 (1.4, 5.4)**
0g sugar per 100ml	-2.0 (-75.5, 71.5)	0.8 (-0.4, 2.0)	**278.9 (185.6, 372.2)**	-1.7 (-4.4, 1.0)	**276.9 (158.2, 395.7)**	-0.9 (-3.9, 2.0)
Bottled water	9.9 (-49.1, 68.9)	-0.4 (-1.4, 0.6)	53.5 (-20.8, 127.8)	-2.6 (-4.7, 0.4)	63.4 (-31.5, 158.2)	**-2.9 (-5.3, -0.6)**
Levy exempt drinks						
Alcoholic drinks	-13.3 (-51.0, 24.4)	-0.03 (-0.4, 0.4)	**212.7 (142.5, 282.9)**	**-5.8 (-7.9, -3.8)**	**199.4 (119.8, 279.1)**	**-5.9 (-8.0, -3.8)**
Milk and milk based drinks*	26.0 (-35.7, 87.7)	**-1.1 (-2.0, -0.09)**	75.7 (-4.5, 155.9)	0.6 (-1.6, 2.8)	**101.7 (0.52, 202.9)**	-0.5 (-2.9, 2.0)
No added sugar fruit juices	-9.9 (-35.0, 15.2)	0.2 (-0.3, 0.6)	**40.9 (10.1, 71.7)**	-0.9 (-1.9, 0.02)	31.0 (-8.73, 70.7)	-0.8 (-1.8, 0.3)
Drinks sold as powders (g)	-3.4 (-9.01, 2.20)	-0.03 (-0.1, 0.06)	5.66 (-1.6, 12.9)	0.07 (-0.1, 0.3)	2.25 (-6.90, 11.4)	0.04 (-0.2, 0.3)
Other categories						
Confectionery (g)	-20.5 (-90.8, 49.8)	0.7 (-1.3, 1.5)	-70.6 (-155.8, 14.6)	**2.2 (0.7, 3.7)**	-91.1 (-201.5, 19.3)	2.3 (-2.2, 6.8)

Bold indicates a significant difference at the 95% confidence interval level.
*Milk comprises drinks in the following categories: semi-skimmed; specific low fat % milk (e.g. 1% fat milk); whole milk; buttermilk; modified milk; other milk; other non-cows milk; rice drink; soya milk. Skimmed milk is excluded from all analysis in this paper due to missing data.

For peer review only

Supplementary Table 5b: Level and trend changes in sugar in drinks and confectionery (g) purchased per household per week (95% CI) in relation to the UK SDIL, March 2014 - March 2019 without toiletries as a control condition (weighted)

	Analysis 1: pre-post announcement (March 2014 – March 2018)		Analysis 2: pre-post implementation (March 2016 – March 2019)		Analysis 3: pre-post announcement & implementation (March 2014 – March 2019)	
	Level Change	Trend Change	Level Change	Trend Change	Level Change	Trend Change
Levy liable drinks						
High tier (≥8g sugar per 100ml)	4.8 (-1.0, 10.6)	-0.008 (-0.1, 0.09)	**-12.1 (-19.5, -4.8)**	-0.2 (-0.4, 0.06)	-7.3 (-16.6, 2.0)	-0.2 (-0.4, 0.07)
Low tier (≥5g - <8g sugar per 100ml)	-0.2 (-1.7, 1.2)	**-0.4 (-0.5, -0.4)**	-1.2 (-2.9, 0.6)	**-0.07 (-0.1, -0.01)**	-1.4 (-3.7, 0.9)	**-0.5 (-0.6, -0.5)**
No levy (<5g sugar per 100ml)	1.7 (-0.2, 3.3)	**0.07 (0.04, 0.1)**	**5.2 (3.2,7.2)**	**0.08 (0.02, 0.1)**	**6.9 (4.4, 9.5)**	**0.2 (0.09, 0.2)**
>0g to <5g sugar per 100ml	1.7 (-0.2, 3.3)	**0.07 (0.04, 0.1)**	**5.2 (3.2,7.2)**	**0.08 (0.02, 0.1)**	**6.9 (4.4, 9.5)**	**0.2 (0.09, 0.2)**
Levy exempt drinks						
Milk and milk based drinks*	2.4 (-0.6, 5.3)	**-0.06 (-0.1, -0.02)**	3.7 (-0.1, 7.5)	0.01 (-0.10, 0.1)	**6.1 (1.3, 10.9)**	-0.05 (-0.2, 0.06)
No added sugar fruit juices	-1.1 (-3.5, 1.4)	0.03 (-0.03, 0.07)	**3.3 (0.3, 6.3)**	**-0.10 (-0.2, -0.01)**	2.3 (-1.6, 6.1)	-0.07 (-0.2, 0.03)
Drinks sold as powders	-1.0 (-3.1, 1.1)	0.01 (-0.02, 0.05)	0.4 (-2.2, 3.0)	-0.01 (-0.1, 0.07)	-0.6 (-3.9, 2.7)	0.001 (-0.09, 0.09)
Other categories						
Confectionery	-11.7 (-51.5, 28.1)	0.05 (-0.8, 0.9)	-42.6 (-90.8, 5.6)	1.3 (-0.4, 3.0)	-54.3 (-116.8, 8.2)	1.3 (-0.5, 3.2)

Bold indicates a significant difference at the 95% confidence interval level.
*Milk comprises drinks in the following categories: semi-skimmed; specific low fat % milk (e.g. 1% fat milk); whole milk; buttermilk; modified milk; other milk; other non-cows milk; rice drink; soya milk. Skimmed milk is excluded from all analysis in this paper due to missing data.

For peer review only

Supplementary Table 5c: Absolute and relative changes in volume of drinks (ml) and confectionery (g) purchased per household per week (95% CI) in relation to the UK SDIL, March 2014 - March 2019 without toiletries as a control condition (weighted)

	Analysis 1: pre-post announcement (March 2014 – March 2018)		Analysis 2: pre-post implementation (March 2016 – March 2019)		Analysis 3: pre-post announcement & implementation (March 2014 – March 2019)	
	Absolute change (ml or g)	Relative change (%)	Absolute change (ml or g)	Relative change (%)	Absolute change (ml or g)	Relative change (%)
Levy liable drinks						
High tier (≥8g sugar per 100ml)	34.4 (-4.5, 73.3)	7.3 (-1.0, 15.6)	**-171.6 (-223.1, -120.0)**	**-42.5 (-55.3, -29.7)**	**-140.9 (-192.5, -89.4)**	**-37.8 (-51.6, -24.0)**
Low tier (≥5g - <8g sugar per 100ml)	**-68.3 (-85.7,-50.9)**	**-38.2 (-47.9, -28.4)**	**-71.4 (-94.1, -48.7)**	**-71.5 (-94.2, -48.8)**	**-171.3 (-193.9, -148.6)**	**-85.8 (-97.1, -74.4)**
No levy (<5g sugar per 100ml)	**187.1 (95.7, 278.5)**	**11.5 (5.9, 17.1)**	**395.0 (273.9, 516.0)**	**19.8 (13.7, 25.9)**	**685.5 (564.4, 806.5)**	**40.2 (33.1, 47.3)**
>0g to <5g sugar per 100ml	**106.4 (68.7, 144.2)**	**17.2 (11.1, 23.3)**	**205.9 (155.5, 256.3)**	**25.6 (19.3, 31.8)**	**374.6 (326.0, 426.8)**	**59.3 (51.4, 67.2)**
0g sugar per 100ml	**80.7 (25.1, 136.4)**	**8.0 (2.5, 13.5)**	**191.9 (118.2, 265.5)**	**16.1 (9.9, 22.3)**	**312.0 (238.4, 385.6)**	**29.1 (22.3, 36.0)**
Bottled water	**-31.3 (-76.3, 13.7)**	**-4.4 (-10.6, 1.91)**	**-76.9 (-136.6, -17.2)**	**-9.89 (-17.6, -2.2)**	**-127.8 (-187.6, -68.1)**	**-15.4 (-22.6, -8.2)**
Levy exempt drinks						
Alcoholic drinks	-16.6 (-62.2, 28.9)	1.0 (-3.6, 1.7)	**-84.9 (-155.8, -14.0)**	**-4.81 (-8.8, -0.8)**	**-103.1 (-174.0, -32.2)**	**-5.8 (-9.8, -1.8)**
Milk and milk based drinks*	**-85.9 (-39.6, 132.2)**	**-2.3 (-3.6, -1.1)**	**106.4 (44.5, 168.3)**	**3.03 (1.3, 4.8)**	-32.8 (-94.6, 29.1)	-0.9 (-2.6, 0.8)
No added sugar fruit juices	5.9 (-13.9, 25.8)	1.2 (-2.9, 5.4)	-6.56 (-32.7, 19.6)	-1.33 (-6.6, 4.0)	6.95 (-19.2, 33.1)	1.5 (-4.0, 6.9)
Drinks sold as powders (g)	**-6.8 (-11.0, -2.57)**	**-6.8 (-10.9, -2.6)**	**9.3 (3.7, 14.9)**	**10.8 (4.3, 17.4)**	0.89 (-4.7, 6.5)	1.0 (-5.0, 6.9)
Other categories						
Confectionery (g)	-9.5 (-72.7, 53.7)	-2.3 (-17.7, 13.0)	40.6 (-42.1, 123.2)	11.9 (-12.3, 36.0)	36.3 (-46.4, 118.9)	10.5 (-13.4, 34.3)

Bold indicates a significant difference at the 95% confidence interval level.

*Milk comprises drinks in the following categories: semi-skimmed; specific low fat % milk (e.g. 1% fat milk); whole milk; buttermilk; modified milk; other milk; other non-cows milk; rice drink; soya milk. Skimmed milk is excluded from all analysis in this paper due to missing data.

Supplementary Table 5d: Absolute and relative changes in sugar in drinks and confectionery (g) purchased per household per week (95% CI) in relation to the UK SDIL, March 2014 - March 2019 without toiletries as a control condition (weighted)

	Analysis 1: pre-post announcement (March 2014 – March 2018)		Analysis 2: pre-post implementation (March 2016 – March 2019)		Analysis 3: pre-post announcement & implementation (March 2014 – March 2019)	
	Level Change	Trend Change	Level Change	Trend Change	Level Change	Trend Change
Levy liable drinks						
High tier (≥8g sugar per 100ml)	3.9 (-0.4, 8.3)	7.6 (-0.78, 15.9)	**-19.6 (-25.3, -13.8)**	**-43.8 (-56.6, -30.9)**	**-16.1 (-21.8, -10.3)**	**-39.0 (-52.9, -25.0)**
Low tier (≥5g - <8g sugar per 100ml)	**-4.9 (-6.1, -3.8)**	**-40.3 (-49.6, -30.9)**	**-4.7 (-6.2, -3.2)**	**-70.9 (-93.3, -48.4)**	**-11.9 (-13.4, -10.4)**	**-86.0 (-96.8, -75.2)**
No levy (<5g sugar per 100ml)	**6.1 (4.9, 7.3)**	**77.7 (62.6, 92.8)**	**9.3 (7.8, 10.9)**	**52.5 (43.6, 61.4)**	**19.2 (17.6, 20.8)**	**240.9 (221.0, 260.8)**
>0g to <5g sugar per 100ml						
Levy exempt drinks						
Milk and milk based drinks*	**-4.2 (-6.4, -2.0)**	**-2.3 (-3.6, -1.1)**	**4.1 (1.1, 7.0)**	**2.4 (0.7, 4.2)**	**-3.2 (-6.5,-0.3)**	**-1.8 (-3.5, -0.1)**
No added sugar fruit juices	**2.6 (0.7, 4.6)**	**5.9 (1.5, 10.2)**	-1.8 (-4.3, 0.8)	-3.7 (-9.1, 1.6)	**2.6 (0.04, 5.2)**	**5.9 (0.08, 11.8)**
Drinks sold as powders (g)	0.4 (-1.2, 2.1)	2.1 (-5.9, 10.0)	-0.3 (-2.4, 1.9)	-1.3 (-12.5, 10.0)	0.9 (-1.3,3.0)	4.7 (-7.2, 16.7)
Other categories						
Confectionery (g)	-5.9 (-42.0, 30.1)	-2.6 (-18.1, 13.0)	23.4 (-24.0, 70.7)	12.1 (-12.4, 36.7)	20.2 (-27.1, 67.6)	10.3 (-13.9, 34.5)

Bold indicates a significant difference at the 95% confidence interval level.
*Milk comprises drinks in the following categories: semi-skimmed; specific low fat % milk (e.g. 1% fat milk); whole milk; buttermilk; modified milk; other milk; other non-cows milk; rice drink; soya milk. Skimmed milk is excluded from all analysis in this paper due to missing data.

For peer review only
